# The eukaryotic translation initiation regulator CDC123 defines a divergent clade of ATP-grasp enzymes with a predicted role in novel protein modifications

**DOI:** 10.1186/s13062-015-0053-x

**Published:** 2015-05-15

**Authors:** A Maxwell Burroughs, Dapeng Zhang, L Aravind

**Affiliations:** National Center for Biotechnology Information, National Library of Medicine, Bethesda, MD 20894 USA

## Abstract

**Abstract:**

Deciphering the origin of uniquely eukaryotic features of sub-cellular systems, such as the translation apparatus, is critical in reconstructing eukaryogenesis. One such feature is the highly conserved, but poorly understood, eukaryotic protein CDC123, which regulates the abundance of the eukaryotic translation initiation eIF2 complex and binds one of its components eIF2γ. We show that the eukaryotic protein CDC123 defines a novel clade of ATP-grasp enzymes distinguished from all other members of the superfamily by a RAGNYA domain with two conserved lysines (henceforth the R2K clade). Combining the available biochemical and genetic data on CDC123 with the inferred enzymatic function, we propose that the eukaryotic CDC123 proteins are likely to function as ATP-dependent protein-peptide ligases which modify proteins by ribosome-independent addition of an oligopeptide tag. We also show that the CDC123 family emerged first in bacteria where it appears to have diversified along with the two other families of the R2K clade. The bacterial CDC123 family members are of two distinct types, one found as part of type VI secretion systems which deliver polymorphic toxins and the other functioning as potential effectors delivered to amoeboid eukaryotic hosts. Representatives of the latter type have also been independently transferred to phylogenetically unrelated amoeboid eukaryotes and their nucleo-cytoplasmic large DNA viruses. Similarly, the two other prokaryotic R2K clade families are also proposed to participate in biological conflicts between bacteriophages and their hosts. These findings add further evidence to the recently proposed hypothesis that the horizontal transfer of enzymatic effectors from the bacterial endosymbionts of the stem eukaryotes played a fundamental role in the emergence of the characteristically eukaryotic regulatory systems and sub-cellular structures.

**Reviewers:**

This article was reviewed by Michael Galperin and Sandor Pongor.

**Electronic supplementary material:**

The online version of this article (doi:10.1186/s13062-015-0053-x) contains supplementary material, which is available to authorized users.

## Findings

The origin of eukaryotes was marked by the emergence of entirely new subcellular systems as well as novel components in preexisting systems. Deciphering the evolutionary history and the ultimate provenance of these systems and components, which were long considered to be quintessential features of eukaryotes, has considerably advanced in the past decade as a result of the growing genomic data and concomitant comparative genomics analysis [1-4]. In this regard, we have had a long-standing interest in understanding the origins of eukaryotic innovations relating to ribosome biogenesis and the translation machinery [5,6]. In several cases, we have been able to identify prokaryotic homologs of what previously seemed to be purely eukaryote-specific components in these systems. Recognition of these prokaryotic versions has helped clarify the precise evolutionary trajectories by which these components were recruited to the eukaryotic ribosome biogenesis/translation apparatus. Moreover, these studies have also often helped predict the potential biochemical roles of several poorly understood components in these systems by exploiting the contextual information available in prokaryotic genomes [5,6].

In this study we present an investigation of the conserved eukaryotic regulator of translation initiation CDC123 and it homologs. CDC123 was first identified over 30 years ago in a screen for temperature-sensitive mutations that blocked cell proliferation in rat fibroblast cells [7]. This was attributed to a cell-cycle related function arising from its apparent functional interaction with checkpoint proteins chf1/chf2 [8], which are active in triggering mitosis entry [9]. Conditional mutants in the *Saccharomyces cerevisiae* cognate were shown to result in increased heat sensitivity, whereas CDC123 null mutants were inviable [8]. Further investigation of these phenotypes pointed to a role in translation as it was observed that CDC123 specifically regulates the abundance of the eukaryotic translation initiation eIF2 complex [8,10], and binds one of its components yeast GCD11 or its human ortholog eIF2γ [11,12] in the cytoplasm. To date its orthologs have only been reported from eukaryotes, where it is widely distributed across all major lineages of the eukaryotic tree. This phyletic pattern, together with its essentiality in yeast, suggest that CDC123 might indeed be a conserved regulator of translation. However, despite over three decades of research on CDC123, its precise role in translation or cell-cycle regulation remains unclear. Given these observations and the mounting evidence suggesting possible links between CDC123 and a variety of human disease states including breast cancer [13], type II diabetes [14], and COPD [15], we sought to apply state-of-the-art methods in comparative sequence and genome analysis to better understand the biochemical roles of CDC123.

We show that CDC123 defines a novel, highly-derived clade of the ATP-grasp superfamily of enzymes [16,17]. We define the conserved sequence and structure features of this clade of ATP-grasp proteins and predict they are likely to catalyze protein modifications by the formation of amide/peptide-like linkages in an ATP-dependent manner. In addition, we identify the first bacterial homologs of CDC123 where they are often found as part of the type VI secretion systems (T6SS) that deliver polymorphic toxins [18,19]. Further, we show that this clade of ATP-grasp domains additionally includes two previously unknown, related prokaryotic families with potential roles in distinct biological conflict systems [20-22]. Finally, we present evidence that the eukaryotic CDC123s might have been derived from an ancestral bacterial conflict system in the stem eukaryote and recruited for a role in protein modifications, including in context of translation initiation.

### CDC123 contains an ATP-grasp module and has several distinct bacterial homologs

To better characterize CDC123, we initiated iterative sequence profile searches with CDC123 orthologs known from prior studies as queries using the PSI-BLAST and JACKHMMER programs (see Methods). Beyond the previously-identified homologs in animals, plants, fungi, and stramenopiles [8], we detected eukaryotic orthologs spanning all other major branches of the eukaryotic tree. For example, a search initiated with the yeast CDC123 recovered orthologs from the apicomplexans, kinetoplastids, parabasalids and diplomonads within 2 iterations with PSI-BLAST (Additional file 1). Concomitantly, these searches also recovered sequences from diverse bacterial and viral lineages. For example, the above search recovered sequences from the γ-proteobacteria *Erwinia chrysanthemi* (gi: 654084322, iteration: 1; e-value 6e-6) and *Legionella pneumophila* (gi: 652968979; iteration: 2; e-value: 2e-08), the planctomycete *Zavarzinella formosa* (gi: 521962559, iteration: 2; e-value: 8e-09), and the nucleocytoplasmic large DNA virus (NCLDV) [23] Megavirus Iba (gi: 448825053; iteration: 2; e-value 1e-11).

Reverse searches initiated with these bacterial sequences recovered their eukaryotic counterparts in initial iterations, then recovered several prokaryotic sequences either unannotated or annotated as containing the “Domain of Unknown Function”, DUF4343 [24], before finally recovering sequences containing known ATP-grasp domains, typically those most-closely related to RimK and RimK-like ATP-grasp families [16]. For example, a search initiated with the bacterial CDC123 homologue from *Lentisphaera araneosa* (gi: 494490064) recovers a sequence annotated as containing the DUF4343 domain from bacterium *Deinococcus pimensis* (gi:653301678; iteration: 4; e-value: 4e-3), a sequence from the bacterium *Pseudomonas aeruginosa* with no previously identified domain (gi: 489255144; iteration: 6; e-value: 4e-05), and a RimK-like ATP-grasp fold [25] domain from *Herpetosiphon aurantiacus* (gi: 501142781; iteration: 8; e-value: 2e-04). We further confirmed these results using an HMM-(Hidden Markov Model) based method for detecting distant homology. HMMs constructed with the CDC123 sequences as seeds were searched against a library of pre-constructed HMMs based on Pfam domain definitions [26] and solved PDB (Protein Data Bank [27]) structures with the HHpred program. The strongest relationship detected in these searches was consistently with the Pfam DUF4343 domain, followed by detection of other ATP-grasp families and structures including the Pfam DUF3182 domain, a heretofore unrecognized member of the ATP-grasp fold sharing conserved features and general sequence affinity with a clade of ATP-grasp enzymes including the carbamoyl phosphate synthases and BtrJ-like butirosin biosynthesis enzymes (Additional File 1). For example, a HMM constructed using the yeast CDC123 sequence as a seed detected a significant relationship with the DUF4343 Pfam domain (p-value: 5.7e-10), the RimK-like ATP-grasp domain (p-value: 9.8e-07), and the RimK structure from *Thermus thermophilus* (PDB: 3VPD; p-value: 6.7E-06). However, in terms of reciprocal recovery in sequence-similarity searches and sequence similarity- and length-based clustering with the BLASTCLUST program (see Methods), none of CDC123 and its newly identified homologs overlapped with any previously known ATP-grasp families [28,16]. Together, these results strongly suggest that these sequences define a previously-unrecognized clade of ATP-grasp-like proteins, which includes the CDC123, DUF4343-containing proteins, and several additional unannotated prokaryotic proteins.

### Distinctive features of the novel ATP-grasp clade and identification of three distinct families within it

The catalytic module of the ATP-grasp superfamily is constructed from two distinct domains: the N-terminal RAGNYA domain and the C-terminal protein kinase/PIPK-like domain [29-31]. In addition to this catalytic module, most members of the ATP-grasp superfamily are fused at the N-terminus to the pre-ATP-grasp domain [16]. The position of the catalytic residues are typically conserved across the superfamily and include: 1) a positively-charged residue, typically a lysine, found in the linker region connecting the pre-ATP-grasp domain with the RAGNYA domain, 2) an additional positively-charged residue, again typically a lysine, found near the C-terminal end of the second strand of the RAGYNA domain, 3) an acidic residue, typically an aspartate, located in the central region of the fourth strand of the protein kinase-like domain, and 4) a conserved motif typically of the form ExN (where ‘x’ is any residue) located at the C-terminus of the fifth and final conserved strand of the protein kinase domain [16]. Additionally, a large, monophyletic clade of ATP-grasp superfamilies, including most peptide/amide bond-forming ligases members, contain a conserved arginine residue in the first strand of the protein kinase-like domain [16] (Additional File 1).

**Figure 1:**
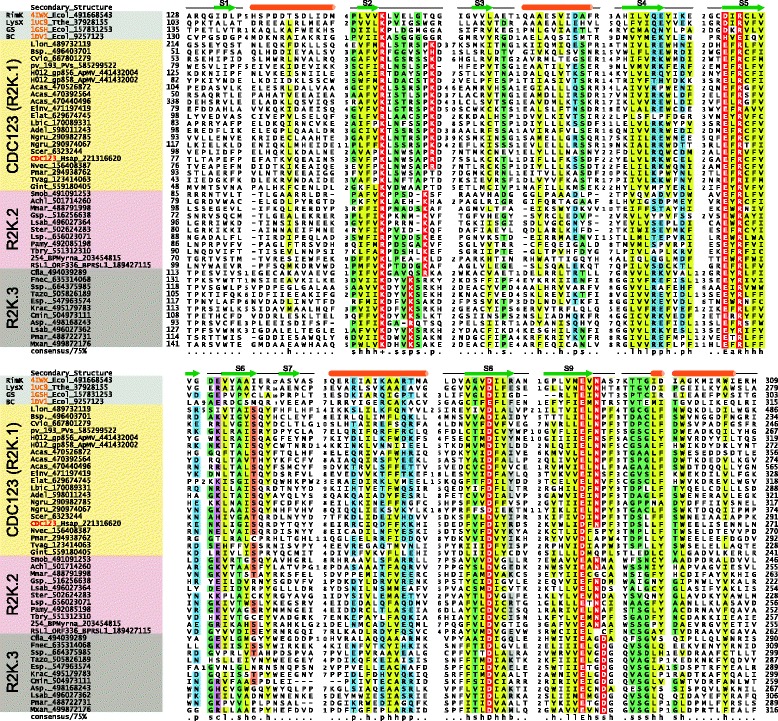
**Multiple sequence alignment of three families of R2K ATP-grasp modules with known ATP-grasp structures.** Proteins are labeled with their species abbreviations and GenBank index numbers along with gene names for human and viral homologs. PDB identifiers, colored in orange, are given in lieu of gene names where applicable. Secondary structures are depicted above alignment with loop regions shown as lines, β-strands (S1-S9) shown as green arrows and α-helices shown as orange cylinders. The coloring of the alignment is based on 75% consensus shown below the alignment, using the following scheme: h, hydrophobic (shaded in yellow); s, small (shaded in light green); l, aliphatic (shaded in yellow); p, polar (shaded in light blue); +, positively charged; b, big (shaded in gray); a, aromatic (shaded in yellow); c, charged (shaded in purple). Predicted catalytic residues are colored in white and shaded in red. Species abbreviations: Acas, *Acanthamoeba castellanii*; Achl, *Arthrobacter chlorophenolicus*; Adel, *Auricularia delicata*; ApMV, Acanthamoeba polyphaga moumouvirus; Asp., *Acaryochloris sp*.; BPMyrna, Mycobacterium phage Myrna; BPRSL1, Ralstonia phage RSL1; Bsp., *Brenneria sp*.; Cfla, *Chthoniobacter flavus*; Cmin, *Chamaesiphon minutus*; Einv, *Entamoeba invadens*; Elat, *Eutypa lata*; Esp., *Eggerthella sp*.; Fnec, *Fusobacterium necrophorum*; Gint, *Giardia intestinalis*; Gsp., *Geitlerinema sp*.; Hsap, *Homo sapiens*; Krac, *Ktedonobacter racemifer*; Lbic, *Laccaria bicolor*; Llon, *Legionella longbeachae*; Lsab, *Lachnoanaerobaculum saburreum*; Lsp., *Labrenzia sp*.; Mmar, *Microscilla marina*; Mxan, *Myxococcus xanthus*; Ngru, *Naegleria gruberi*; Nvec, *Nematostella vectensis*; PVs, *Pithovirus sibericum*; Pamy, *Pseudomonas amygdali*; Pmar, *Perkinsus marinus*; Pmar, *Planctomyces maris*; Scer, Saccharomyces cerevisiae; Smob, *Streptomyces mobaraensis*; Ssp., Streptomyces sp.; Ster, *Sebaldella termitidis*; Tazo, *Treponema azotonutricium*; Tbry, *Treponema bryantii*; Tvag, *Trichomonas vaginalis*. Other abbreviations: GS, glutathione synthase; BC, biotin carboxylase.

Comparison of the features of the newly-identified clade to the above-described ATP-grasp template revealed considerable concordance (for example: K104, D233, and D246xN248 correspond to features 2-4 listed above in the human CDC123 protein). However, striking differences were observed: 1) In other ATP-grasp families the loop between strands 2 and 3 of the RAGNYA domain is well-conserved in terms of length (usually 9 amino acids) and harbors a conserved ssxGbGl motif (where ‘s’ is any *s*mall residue, ‘b’ is any *b*ig residue, and ‘l’ is any a*l*iphatic residue) [16]. However, in this novel clade this loop displays considerable length diversity and lacks the above sequence motif. 2) The lysine typically observed in the linker region between the pre-ATP-grasp domain and the RAGNYA domain is consistently absent in all members of this newly detected clade (Figure 1). Instead, they display a distinct conserved lysine/arginine in the above-stated loop, just downstream of the absolutely conserved lysine in strand 2 (Figure 1). This loop region is spatially positioned in close proximity to the active site [28]. Hence, we predict the conserved lysine/arginine from this loop likely acts as a secondarily-acquired, compensatory residue that functions in lieu of the conserved lysine from the pre-ATP-grasp-RAGNYA linker region. Indeed, these shared features strongly support the monophyly of this clade of ATP-grasp enzymes and we propose naming this novel clade the R2K ATP-grasp clade, for *R*AGNYA-containing *2* lysines (*K*).

To further understand the relationships within the R2K clade, we clustered its representatives using sequence similarity- and length-based scoring parameters with the BLASTCLUST program (Additional file 1). The results identified three distinct families: 1) the CDC123 or R2K.1 family consisting of the pan-eukaryotic CDC123-like proteins, close homologs in certain NCDLVs infecting microbial eukaryotes, and bacterial versions from α-, δ-, and γ-proteobacteria, planctomycetes, lentisphaerae, and firmicutes; 2) the R2K.2 family sporadically present across many bacteria and a few bacteriophages, typically annotated as matching the Pfam DUF4343 model; 3) the R2K.3 family with a similar phyletically wide, yet sporadic, distribution in bacteria with rare archaeal representatives. The R2K.3 family is often misannotated as a “membrane protein”, typified by the sce1853 protein in *Sorangium cellulosum*. Each of the families is clearly distinguished from the other by the spacing of the second conserved lysine with respect to the absolutely conserved lysine in strand 2 of the RAGNYA domain (Figure 1). A subset of the families or members within each family might show certain peculiarities: the eukaryotic versions of the CDC123 family are often characterized by large, variable, low complexity inserts within the catalytic module predicted to be structurally disordered. The pre-ATP-grasp domain is well-conserved in the R2K.3 family but is rapidly diverging in the CDC123 and the R2K.2 families. The R2K.3 family is further distinguished by an unusual constellation of conserved residues in the final strand of the protein kinase/PIPK-like domain of the ATP-grasp module, where it contains an ExGD motif instead of the standard ExN motif (Figure 1). While the N residue is, on occasion, substituted for distinct polar residues, the migration of the residue one position downstream has not, to our knowledge, previously been observed in the ATP-grasp superfamily.

### Evolutionary history of the R2K clade ATP-grasp enzymes

**Figure 2:**
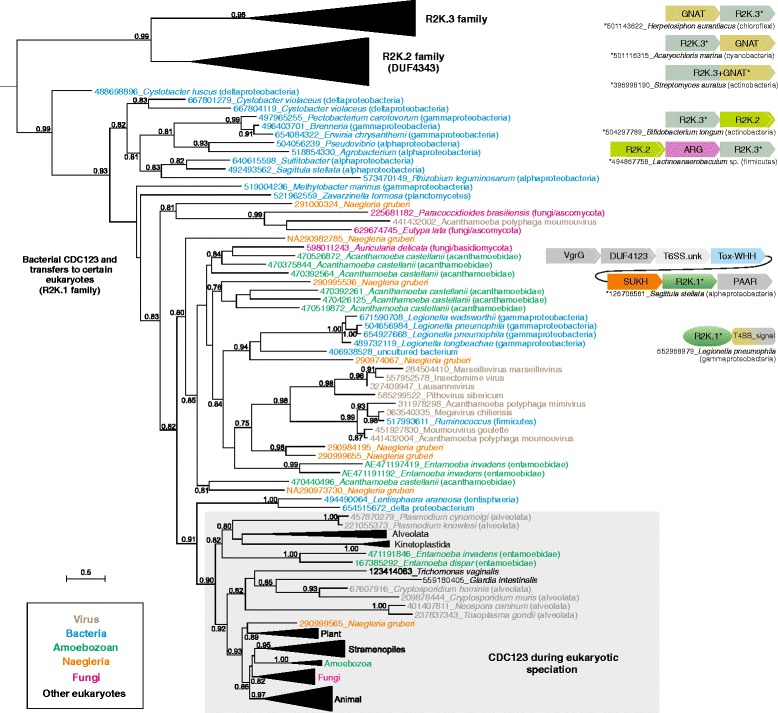
**Evolutionary relationship of three families of the R2K ATP-grasp module shown to the left and conserved contextual associations including operonic organizations and domain architectures are provided on the right.** Tree nodes supported by bootstrap >75% are shown. Proteins are denoted by their GenBank index numbers and their complete species names and colored according to their lineages: bacterial in blue, viral in green, amoeboazoan in orange, *Naegleria* in purple, fungal in red. Conserved gene neighborhoods are depicted as boxed, labeled arrows with the arrowhead pointing to the C-terminus of the protein. Genes known to be part of the T6SS are shaded in gray, including the “T6SS.unk” gene containing a domain of unknown function in the secretion system. Conserved domain architectures are depicted as adjoining, labeled shapes.

Despite their distinctive features, the fusion to the pre-ATP-grasp domain points to the R2K clade being deeply nested within the previously defined tree of ATP-grasp-like modules [16] (Additional File 1). Moreover, the presence of the conserved arginine residue in the first strand of the protein kinase/PIPK-like domain of the ATP-grasp module (part of the conserved ExR motif in S5 of Figure 1) suggests that the R2K clade specifically belongs to a larger assemblage within the superfamily that is almost entirely comprised of ligases catalyzing peptide-like linkages [16]. This assemblage includes the ATP-grasp enzymes catalyzing the formation of such bonds in cofactors (e.g. glutathione), antibiotics [32,33], peptidoglycan [34,35], siderophores [36], the biosynthesis of lysine (LysX), and catalyzing polyglutamyl and polyglycinyl modification of cofactors and proteins like ribosomal protein S6 and tubulin [37,38]. The majority of these families appear to have first radiated in the bacteria [16]. Similarly, all three families of the R2K clade have a bacterial presence, with the eukaryotic CDC123s nested within the bacterial diversification of this clade in a phylogenetic tree (Figure 2). These observations suggest the R2K clade first emerged in bacteria followed by initial diversification into three distinct families. Additionally, the phyletic patterns of bacterial versions and their relationships in the phylogenetic tree (Figure 2, Additional File 1) strongly suggest horizontal gene transfer (HGT) as the key theme in their evolution.

Two distinct versions of the CDC123 (R2K.1) family are found in eukaryotes. The phyletic patterns suggest that the classical CDC123 orthologs, typified by relatively short average branch terminal lengths (Figure 2), were likely to have been present in the Last Eukaryotic Common Ancestor (LECA), suggesting that a HGT event from a bacterial source transferred these to the stem of the eukaryotic lineage. A second set of more rapidly-evolving CDC123 family members are found primarily in phylogenetically distant amoeboid organisms like *Entamoeba*, *Acanthamoeba*, and *Naegleria*, often in multiple copies (Figure 2). These group with cognates from facultative bacterial symbionts of amoebae, namely *Legionella* and giant NCLDVs that infect amoeboid organisms [39] (Figure 2). The complex interplay between *Legionella* and eukaryotic hosts [40,41] has previously been proposed to have been a conduit for HGT of multiple domains [42,43]. Similarly, transfers between symbionts and viruses sharing the same host cell have also been documented [44,45,39]. Thus, the distinctive members of the R2K.1 shared by amoeboid eukaryotes and their symbionts and viruses were likely disseminated via HGT associated with these interactions.

### Functional inferences for R2K families based on genome contextual information and prior experimental results

Based on the conservation of most key catalytic residues or their compensation with spatially-equivalent residues from elsewhere in the sequence we propose that most members of the R2K clade are likely to be active enzymes, although in some lineages this activity may have been lost, most notably in the eukaryotic apicomplexan clade (Figure 1, Additional File 1). Furthermore, based on the nesting of the R2K clade within the ATP-grasp assemblage, which primarily catalyzes the formation of peptide-like linkages [16] (Additional file 1), we propose that members of this clade are likely to catalyze similar reactions. Yeast strains overexpressing CDC123 displayed a second, slightly larger isoform of CDC123 at low levels [46]. This isoform was suggested to result from an unknown modification to CDC123 and was linked to its proteasomal degradation [47]. The same work ruled out ubiquitin- and phosphoryl-group additions as potential modifications resulting in this isoform [47]. In light of the peptide-bond forming activity predicted for the R2K clade ATP-grasp proteins, we posit that the observed isoform perhaps results from automodification via serial ligation of amino acids to a particular sidechain or the C-terminus comparable to the modifications catalyzed by RimK on the ribosomal protein S6 or the TTLs on tubulins. RimK has been shown to ligate up to fifteen glutamate residues to S6 [48,25]; auto-ligation of a comparable number of amino acid residues would be sufficient to explain the observed larger isoform of CDC123. The interaction networks for various CDC123 eukaryotic orthologs inferred from high-throughput interactome studies show an enrichment for multiple proteosomal components [49]. This, together with heat-sensitivity of the CDC123 mutants, suggests that one consequence of this modification might be to regulate the stability of proteins via the proteasome. However, it is likely that the CDC123-catalyzed modification has a distinct role in the context of translation initiation. Physical interaction of CDC123 with GCD11/eIF2γ and the marked decrease in eIF2 complex formation without changes in concentration of individual eIF2 complex components in the CDC123 null mutants [10] suggest that the modification of particular components might facilitate assembly of this key translation initiation complex. Similarly, the cell-cycle checkpoint proteins Chf1/Chf2 [8] might also be other targets for modification catalyzed by CDC123.

We then examined the contextual information in the form of conserved gene neighborhoods and gene fusions of the prokaryotic versions as this has proved to be a useful tool for deciphering the function of uncharacterized gene products [50,51]. Consequently, we observed that across several phylogenetically distant bacteria, genes coding for members of the CDC123 (R2K.1) family are embedded within the recently-described polymorphic toxin loci (Figure 2). Polymorphic toxin systems have been implicated in intra-specific conflicts between bacteria, acting as the arbiters of “self versus non-self” distinctions between closely-related organisms [18,52,19,21]. The toxin proteins from these systems are delivered to target cells via a wide range of secretory systems, which are often genomically-linked to the core loci coding for the toxin and its cognate immunity protein [18]. Among these secretory systems is T6SS, which utilizes caudate bacteriophage tail-derived components to inject toxins into target cells [53]. We observed that CDC123 occurs specifically in polymorphic toxin loci with genes coding for the SUKH domain immunity protein [19] and diagnostic components of the T6SS system including the VgtG, Hcp1, and proteins with PAAR motifs [18] (Figure 2). As only a subset of polymorphic toxins delivered by T6SS encode a CDC123-like protein, it is likely to function in a supplementary role, perhaps as a secondary toxin injected into the target organism or as an auxiliary protein that regulates either the toxin, the immunity protein, or the secretory apparatus.

The CDC123 family protein found in Legionellae contains extended C-terminal and N-terminal regions not observed in other CDC123-like proteins (Figure 2, Additional file 1). *Legionella* secretes several toxins/effectors into its eukaryotic host cell using the Type IV secretion system (T4SS). The C-terminal region of CDC123 from Legionellae harbors several of the characteristics known to be important for T4SS delivery such as: 1) a largely unstructured C-terminal region [54], 2) a conserved hydrophobic residue very close to the C-terminus [54], and 3) a preponderance of both small and polar residues in the ~15 residues upstream of the hydrophobic residue [55] (Additional file 1). Hence, it is conceivable the *Legionella* CDC123 is secreted via the T4SS as an effector into the host eukaryotic cell. Thus, the evidence from the two distinct sets of bacterial members of the CDC123 family point in the direction of functioning as a secreted toxin or auxiliary factors of toxin systems, which might modify proteins with peptide tags by means of their peptide ligase activity. Given the second set of eukaryotic and NCLDV CDC123 homologs are specifically related to the *Legionella* versions, it is likely that these perform functions similar to the former and different from the classical CDC123 translation regulators referred to above. Their presence, often as multiple paralogous copies (unlike the single-copy classical CDC123 versions) across phylogenetically distant amoeboid eukaryotes (Additional File 1), raises the possibility that they modify cytoskeletal proteins associated with the amoeboid cellular morphology, such as components of the actin-based cytoskeleton. This might parallel the extensive modification of tubulin by peptide tags, ranging from a single tyrosine to long polyglutamyl or polyglycinyl chains, catalyzed by multiple ATP-grasp ligases [37,38,56,57]. Thus, such cytoskeletal modifications could be utilized by both the amoeboid organisms and their symbionts/parasites in facilitating formation of intracellular structures conducive to their lifestyle.

We observed operonic connections between genes of the R2K.3 family and those coding for multiple GCN5-like acetyltransferase (GNAT) domains in several actinobacteria of the *Streptomyces* lineage, the chloroflexi *Herpetosiphon*, and the cyanobacterium *Acaryochloris* (Figure 2). In certain firmicutes and the actinobacteria, genes for the R2K.3 and R2K.2 families were linked together in the same operon (Figure 2). The operonic linkage of genes for distinct ATP-grasp peptide ligases or unrelated ligase domains, such as those of the COOH-NH2 ligase or GNAT superfamilies, have been previously observed in multiple instances [16]. Such linked peptide ligases often catalyze successive peptide ligations with distinct moieties in the biosynthesis of peptide-derived secondary metabolites like antibiotics and siderophores, storage polypeptides like cyanophycin, peptidoglycan, teichuronopeptides, the O-antigen, and cofactors like glutathione [58,25]. Hence, we posit that the R2K.2 and R2K.3 families catalyze peptide ligation, which might be further followed by action of the second ligase or capped by an acyl group added by the associated GNAT protein. In certain firmicutes, the linked genes for the R2K.2 and R2K.3 family proteins sandwich a third gene coding for an ADP-ribosylglycohydrolase (ARG) (Figure 2). ARGs catalyze the hydrolysis of glycosidic bonds to remove ADP-ribose moieties conjugated to side-chains of particular residues in proteins by ADP-ribosyltranferases [59,60]. This linkage suggests that, like the ARG, the peptide ligase action of R2K.2 and R2K.3 enzymes is likely to target proteins. As there are no other linked genes in these neighborhoods, the identity of their target proteins remain elusive. Nevertheless, given that at least the R2K.2 family is found in several caudate bacteriophages infecting phylogenetically-distant bacteria (Additional File 1), it might modify specific host proteins, analogous to the ADP-ribose modification of the same by phage enzymes [61-63]. Conversely, even as phage-derived proteins are occasionally redeployed by the host against other viruses [64], it is possible that the bacterial versions are deployed against proteins encoded by invasive operons. This proposal is also consistent with the sporadic distribution of these families indicative of HGT and gene-loss, which is similar to that of other families of proteins implicated in providing specific selective advantage in biological conflicts [65,66].

### General conclusions

We present the discovery of a novel clade of ATP-grasp enzymes, the R2K clade, which includes the conserved eukaryotic protein CDC123. We show that this clade displays certain aberrant features hitherto not encountered in other members of the ATP-grasp superfamily. Nevertheless, the weight of the evidence suggests that they belong to the vast assemblage of ligases catalyzing formation of peptide bonds or similar linkages in the biosynthesis of a variety of compounds and also in the peptide-tag-modification of target proteins. We propose that the classical CDC123 family is likely to modify proteins, including possibly components of the eukaryotic eIF2 translation initiation complex. Importantly, we show that the CDC123 family had its origins in bacteria where it appears to have diversified first along with the two other families of the R2K clade. The bacterial CDC123 proteins are of two distinct types, one specifically associated with T6SS-delivered polymorphic toxin systems and the other probably functioning as effectors directed at amoeboid eukaryotic hosts. Similarly, the R2K.2 and R2K.3 families are also proposed to participate in biological conflicts, probably between bacteriophages and their hosts. Thus, our findings not only help predict an unexpected biochemical function for a poorly understood translation initiation factor but also help trace its origin back to bacterial conflict systems, where it might have been deployed as a toxin in intergenomic/interorganismal conflicts [22,21].

Previously, several key components of the eukaryotic protein modification and signaling systems, such as the ADP-ribosyltransferases, DOT1-like protein methyltransferases, and Fic/Doc-like protein AMPylating enzymes, have been traced to polymorphic toxin- or related host targeting effector-systems of endosymbiotic bacteria [67,18]. CDC123 joins these as a potential protein modification system that was recruited from a bacterial effector. This observation adds one more piece of evidence to the recently proposed hypothesis that effectors from the bacterial endosymbionts of the stem eukaryotes played a fundamental role in the emergence of the characteristically eukaryotic regulatory systems and sub-cellular structures [21]. Moreover, the diversification of the R2K clade in bacteria and their phages also adds support to the hypothesis that the exchange of a common set of protein- and nucleic-acid-modifying enzymatic effectors between disparate bacterial conflict systems helped in their extensive diversification. Representatives of this pool of enzymes were repeatedly taken up by eukaryotes and used as components of novel regulatory systems.

### Methods

Iterative sequence-profile and HMM searches were performed using the PSI-BLAST [68] and JACKHMMER web utilities (http://hmmer.janelia.org/search/jackhmmer), respectively. Queries were run against the non-redundant (nr) protein database of the National Center for Biotechnology Information (NCBI). Profile-profile comparisons were performed using the HHpred program [69]. Multiple sequence alignments were constructed using the MUSCLE alignment program [70] followed by manual adjustment as determined by high-scoring pairs detailed in homology search results and alignment with experimentally-elucidated protein structures. Alignment secondary structure predictions were performed with the JPred program [71]. Gene neighborhoods were extracted from PTT and GenBank files (downloadable from the NCBI ftp server) using Perl scripts. Sequence-based homology clustering of all proteins determined to belong to the R2K assemblage and proteins encoded in the recovered gene neighborhoods was performed with the BLASTCLUST program (http://ftp.ncbi.nih.gov/blast/documents/blastclust.html) using empirically-determined scoring and length threshold values. Visualization and manipulation of protein structure was accomplished using the PyMol program (http://www.pymol.org), structure similarity searches were performed using DaliLite [72]. Phylogenetic trees were constructed using the maximum likelihood method as implemented by the PhyML program [73].

## Reviewer reports

### Reviewer number 1: Dr. Michael Galperin, NCBI, NLM, NIH, United States of America

The work by Burroughs and colleagues is an important contribution that expands the diversity of the members of the ATP-grasp superfamily and proposes an enzymatic function for a widespread eukaryotic translational regulator CDC123. I have only some minor comments that might improve the presentation of the results.

1. Members the ATP-grasp superfamily are primarily ATP-dependent carboxylate-amine ligases [17], although certain members are known to function as carboxylate-thiol ligases, carboxylate-hydroxyl ligases, or phosphotransferases (kinases) [74]. It would make sense to explicitly state in the Abstract that CDC123 is predicted to function as an ATP-dependent protein-peptide ligase (or a protein-amino acid ligase) and indicate that future experiments would be required to uncover the exact nature of the CDC123-catalyzed reaction.

Author response: *We have made the suggested change to the abstract.*

2. Sequence alignment on Figure 1 would benefit from inclusion of sequences of several ATP-grasp superfamily members of known 3D structure, such as RimK (4iwx), LysX (1uc9), glutathione synthase (1gsh), and/or biotin carboxylase (1dv1). This would help illustrate the common and distinct features of CDC123-like and typical ATP-grasp enzymes.

Author response: *We agree this change increases the information conveyed by the figure; we accordingly have altered the figure and its legend according to the reviewer’s recommendation.*

3. While sequences of human and yeast CDC123 are indicated on Figure 1, it would make sense to explicitly list in the text the predicted active site residues of the human protein that should be targeted by future experimental efforts.

Author response: *The information has been added to the text.*

4. In the Figure 2 legend, the D123 label needs to be explained, and it should be stated that all other labels are specified in the main text. Since RefSeq gi numbers 159900810, 158338501, 493650113, and 492493562 are now obsolete, these need to be replaced with the respective GenBank codes. Also, gi 504297789 (Bifidobacterium longum) is repeated twice; the second instance appears to be incorrect.

Author response: *We thank the reviewer for identifying these issues. We have replaced the D123 label with R2K.1 and updated the gi labels.*

5. The suggested name R2K clade is hardly ideal, as this name is widely used in other contexts, such as the “R2K theology”. There could be better acronyms for “RAGNYA-containing 2 lysines” (e.g., R2Lys) that would not have such connotations.

Author response: *We thank the reviewer for cross-checking the R2K name and drawing this to our attention; however, given the relative obscurity of the R2K theological doctrine within Christianity and the relative independence between the respective fields (molecular biology and theology), we believe it should not be a notable source of confusion. We admit that the growth of the annotated domain space makes simple yet meaningful names for domains that might not overlap with nomenclature used elsewhere increasingly difficult to assign. However, we respectfully suggest that is better to retain “R2K”. Its primary advantage is its simplicity and it remains a proper descriptive moniker for this clade of the ATP-grasp superfamily.*

### Reviewer number 2: Professor Sandor Pongor, International Centre for Genetic Engineering and biotechnology (ICGEB), Italy

It is often argued that the growing body of sequence data will eventually provide answers to many important questions, eukaryogenesis remains one of the fundamental problems of evolution which is still very difficult to tackle. The main problem is the complexity of genetic and biochemical systems which have to be taken into consideration when interpreting distant structural and functional similarities. This paper presents such an interesting prediction, the authors conjecture that the eukaryotic translation initiation regulator CDC123 defines a novel clade of ATP-grasp enzymes which may have role in novel protein modifications. The finding is based on combining the available biochemical and genetic data on CDC123 with the inferred enzymatic function. The authors present a clear case and do not over interpret the data.

Author response: *We appreciate the reviewer’s kind comments.*

## Additional file

Additional File 1:
**Provides an updated tree showing the relationship of the ATP grasp modules, a more extensive alignment of the R2K clade, its phyletic distribution, and its gene neighborhoods.**

## Abbreviations

R2K: RAGNYA-containing lysine
DUF: Domain of Unknown Function
HMM: Hidden Markov Model
LECA: Last Eukaryotic Common Ancestor
GNAT: GCN5-like acetyltransferase
ARG: ADP-ribosylglycohydrolase
