# Supplementary material for: The eukaryotic translation initiation regulator CDC123 defines a divergent clade of ATP-grasp enzymes with a predicted role in novel protein modifications
Source: Biol Direct. 2015 May 15;10:21. doi: 10.1186/s13062-015-0053-x (PMC4431377; doi:10.1186/s13062-015-0053-x)
Supplement: Additional File 1: — Provides an updated tree showing the relationship of the ATP grasp modules, a more extensive alignment of the R2K clade, its phyletic distribution, and its gene neighborhoods. [file 13062_2015_53_MOESM1_ESM.html]

 

```
The CDC123 protein defines a divergent clade of active ATP-grasp fold members evincing novel structural features and a complex evolutionary history

					SUPPLEMENTARY MATERIAL

				A Maxwell Burroughs, Dapeng Zhang, L Aravind

					     NCBI/NLM/NIH

Abstract

Deciphering the origin of uniquely eukaryotic features of sub-cellular systems such as the translation apparatus is critical in reconstructing eukaryogenesis.
One of these is the highly conserved, but poorly understood, eukaryotic protein CDC123 which regulates the abundance of the eukaryotic translation initiation
eIF2 complex, and binds one of its components eIF2gamma. We show that the eukaryotic protein CDC123 defines a novel clade of ATP-grasp enzymes distinguished from
all other members of the superfamily by a RAGNYA domain with two conserved lysines (henceforth the R2K clade). Combining the available biochemical and genetic
data on CDC123 with the inferred enzymatic function, we propose that the eukaryotic CDC123 proteins are likely to modify proteins by ribosome-independent
addition of an oligopeptide tag. We also show that the CDC123 family emerged first in bacteria where it appears to have diversified first along with the two
other families of the R2K clade. The bacterial CDC123 family members are of two distinct types, one found as part of type VI secretion system-delivered
polymorphic toxins and the other functioning as potential effectors delivered to amoeboid eukaryotic hosts. Representatives of the latter type have also been
independently transferred to phylogenetically unrelated amoeboid eukaryotes and their nucleo-cytoplasmic large DNA viruses. Similarly, the two other prokaryotic
R2K clade families are also proposed to possibly participate in biological conflicts between bacteriophages and their hosts. These findings add further evidence
to the recently proposed hypothesis that the horizontal transfer of enzymatic effectors from the bacterial endosymbionts of the stem eukaryotes played a
fundamental role in the emergence of the characteristically eukaryotic regulatory systems and sub-cellular structures.

-------------------------------------------------------------------------------------------------------

Table of Contents

I. Superalignment of all R2K-like ATP-grasp families

II. Individual family alignments
	A. R2K.1 family (cdc123-containing)
		1. bacterial representatives only
		2. all representatives
	B. R2K.2 family
	C. R2K.3 family

III. Sequences, domain architectures, and phyletic distributions for domain families
	A. R2K.1 family (cdc123-containing)
	B. R2K.2 family
	C. R2K.3 family

IV. Conserved gene neighborhoods
	A. R2K.1 family
	B. R2K.3 family

V. Updated evolutionary history of the ATP-grasp fold

-------------------------------------------------------------------------------------------------------

I. Superalignment of all R2K-like ATP-grasp families

Sequences labeled to the left with ncbi gene identifier (gi) number.

ALIGN	   --------------------------------------------HHHHHHHHHHH------------------EEEE---------------------------------------------------------------------HHHHHHHHH--------H--HHHHH----------------------------------------HHEHEEH-----------------------------------------EEEEEE--------------------EEEE-------------------------------HHHHHHHHH-HHHH-HH---------------------------EEEEE-EEEE-----------------------EEEEEE-------------------------------HHHHHHHHH-H----
HMM	   --------------------------------------------HHHHHHHHHHH------------------EEEE-----HHHHHHH-------------------------------------------------E---H---HHHHHHHHH-HHHHHHHH--HHHH------------------------------------------EEEEEE-----------------------------------------EEEEEEE-------------------EEEEE--------------------------------HHHHHHH-HHHH-HH----------------------------EEEE-EEEE----------------------EEEEEEE--------------------------------HHHHHHHH------
FREQ	   -------------------------------------------H-HHHHHHHHHH------------------E----------HHHHH-----------------------------------------------------------EEEEE------E---------EE--------------------------------------HHHHHHHHHHH--------------------------------------HHHHHHHHH------------------EEEE----HHHHHHHH-------------------HHHHHHHHH-HHHH-HHHE--------------------------EEEE-EEE-----------------------EEEEEE-------------------------HHHH---HHHHHHHH-------
PSSM	   -------EE----------------------------------HHHHHHHHHHHH------------------EEE------------------------------------------------------------------H---HHHHHHHHH--HHHH---------------------------------------------------EEEEEE-----------------------------------------EEEEEEE-------------------EEEE-------------------------------HHHHHHHHH-HHHH-H-----------------------------EEEE-EEE------------------------EEEEEE---------------------------------HHHHHHH------
FINAL	   -------EE----------------------------------HHHHHHHHHHHH------------------EEE--------HHHHH-----------------------------------------------------H---HHHHHHHH---------------------------------------------------------EEEEEEEE----------------------------------------EEEEEEE-------------------EEEEE----HHHHHHH-------------------HHHHHHHHH-HHHH-HHH---------------------------EEEE-EEE-----------------------EEEEEEE--------------------------HH---HHHHHHHHH------
CDC123 (R2K.1)
655450018  GAAATKKSFP---------------------------------GFEAAILKAIEKL--------------GGKVFVKTNWSSPRDAVWVS---------------------------------------------GTLQCQSV---GEIILLLKSSDFVSFDLE--HAYDECREESTE---------GESV-----------RNASKRPEHFCLVLRKWCNLF---------------------------------PSQEFRCFVKD----------------RQLIATSQRDPTKFYNFLPSN---------------VSTYSPLVSE-FFDT-SIRPVFP--------------------DDNFSFD-VYIDR-------------------KHRVWLQDFNVF----------------SRATNALLF---TWAELLAVD-TQEAD
406938528  ---LTKEDVK---------------------------------LLEKLRRNIQLHL---------NKSKSTHGFFVRMSNRSPKDGTPLKNKSMVDIYKE--------------IYSNPNDDWNNKMIKICDAQMKMLCCQNA---DEVMNLLLSSERIYMDLI--EALDCHLYSKS------------------------------DLWKTSVILREWIPDLK--------------------------------QDFEFRIFVSN----------------NHVTATSQYNHYCCFESLMIL-------------NQHNELMKLNQR-LIDY-AMKIHPLIN------------------KSQYVLD-VALIN--------------------NELYVIELNPF----------------DKSTGPCLF---SWEKDSELL-TGNGS
517993611  ---INNIVPN---------------------------------NLIKKISKAMSKI--------------SCSCFIRTDAYSPKDLLHRE-------------------------------------------IVDTLEVTNA---ITALKLITQSERCSSKLF--GGDN-------------------------------------KIISKNIVIREYVNYD---------------------------------TNYEFRCFVYN----------------WRLTAISQSGFEYNPILHSKK----------------NIIYQSIIK-FWDK-FSRICP---------------------YSECTMD-IVYNDKWIDNTL-----------NHSGVVIIEFN---------------------------------------------
494490064  ---AQGKSLA---------------------------------SLEERITRAIASL--------------PGSCVVGLDSCMPTDSVSFQ---------------------------------------------KSKSLKNG---KLALKILNESEKVKSAVS--------------------------------------------SGDKTITVRPYRRMD---------------------------------KTREFRLFVKD----------------GELKAMTQRNLERHFKRLEAR---------------RELYWQKGVS-FVDE-IKKYIN---------------------EKDYVVD-IYFTS-------------------TADIMIVDFNSW----------------GEPTLPLLMK--SWERDWSSV-DGLKL
521962559  ---NDGEACT---------------------------------RQVECGRRALAEF--------------PDGVFFKLDSRSPKDSDI-----------------------------------------------GKYTAENL---DQLPNAFFGSERVFDDIC--LQRH-------------------------------------HRDRIVLCFRKWVEFG-----------------------------------EEYRVFVKE----------------RQIQGISRYDYLSASKVEHTP--------------EVVAAVQGQAEGYLAT-INEHYP---------------------PSDYVFD-IGHTP--------------------DGPVMIEINPY-----------------GLSDPCLF------------------
640615598  ---ARDFLSK---------------------------------ALLTEIDSALLKY--------------PEGVMPRIGYCSWKASLI-----------------------------------------------EKRAAKTR---NDVMQIITTDDPRVGNAL--SVLVG------------------------------------SDDPVVLHLRAWRKIP---------------------------------DWSEVRLFFKN----------------GRFVGASQYAYRRSFPEITSN---------------AKEIETMVAR-AASV-IVEKLH---------------------LSDVIVD-LALLPSDGKSPV-----------SGLKPLLIELNPF----------------SPLTDACLF---SWEKGGNFD-GRFR-
504056239  LAANTPEFCE---------------------------------QFGIFNHYGLSEE--------------FRDTISKGL-EKFKDGAFPR------------------------------------------LDYCSWKTSCL---LNAPAKSLGEVEAIVLQP--NQRVASALMDP----------------------------VINNTGANFYLRKWVDIP---------------------------------RWSEFRIFMRD----------------RKIIGVSQYYTDEQFPALQEN---------------LDKIREALIE-FCLF-FYKESH---------------------LDTVVAD-VFLANQNEK----------------LQAQLIELNPF----------------LNRTDPCLY---NWEK-----------
518854330  --SRTAAFRE---------------------------------LFDIDELTQLSDE--------------FRIGIDAKT-ALFKDGAHFR------------------------------------------LGGCSFKQPGR---YQDGPIFNSAQLMPHVLR--DNPRVAGLLASS---------------------------LQDKFDVCMFIRPWENIP---------------------------------KWSEFRLFMKN----------------REFIGASQYFHTAFFPEIEAK---------------ARSIAVALVE-FADR-FRQVAH---------------------IDDAIVD-IYLRPDDAGG---------------FEGVLLDLNPL----------------ILRSDPCLF---QWKNGGDFD-RGLR-
492493562  ---RRIGFGP---------------------------------DFEKTIAKALETF--------------PEGLMPRIGMCSWKAST-----------------------------------------------VVHAPCHSV---ADVMRVITANDPRVAQAI--LDHRI------------------------------------SKRPVVLHLRAWRDIP---------------------------------DWAEFRLFVKR----------------RGLLGVSQYAWQETFPQIAAQ---------------HSAIVTAVNA-LLKD-IWEDLH---------------------MDDVVID-VCVLPEGDG----------------LKAWLIELNPL----------------DPRSDACLY---SWENGGDFD-GSFRY
573470149  ---RFLNISQ---------------------------------PVSLPSRGFGKKM--------------EGDAFPKIGPVSWKEISAFI---------------------------------------------SVPLAAID---ELMPVMLRGVTDRMAFIL--HAFVC------------------------------------RQVSTKLHVFPFVDLS---------------------------------KAFEVRFHIED----------------GEPVHAKWMNRSDRYVPPPGS--------GEKLSNFAANIAERVG----------------------------------IGYALLD-LLLIKGADGE----------------AIKVVEVNPI---------------LERSASGRLF------------------
667804119  -VASDPEGLH---------------------------------SLAARLQKALEGV--------------EQGVFVRLGSGSPKDSALFR---------------------------------------------EQGGCART---PMMALKFLQTSPRTRAHL--SRFLE------------------------------------LGHPVHLFVRHWVRIP---------------------------------PWQEFRCFMRN----------------RRLVGISQLAHRGDTPEYSLA-------------PRAEELGRTLQD-FFVG-VARASH---------------------VGSAVFD-VWCDTGAGDGAP-------------ARVWLLDANPW----------------GPASDACLF---DWSQPEGFD-GSFRY
488698896  -DAVGTAPLT---------------------------------ALQAQLDGLLQQF--------------PAGAFVRLGSRSPKDTERFV--------------------------------------------LSGGRADSG---AEAIALLSAGSRRMFVDY--RRCMQ------------------------------------NHWTPSIFLREWQPMS---------------------------------SAQEWRCFVHE----------------RQLLGITQYWHAQALDSGACA-------------QLQRTGIASLLQ-LAAQ-LLQRLP---------------------LPSFVFD-ACLPLPSPLA---------------ARAVLIEINPF----------------GATTDAGLFD--ACDERLDRS-IRWRS
654515672  ---VDECEVY---------------------------------HIVNRIAHAQQAF--------------FGSKFVFADTIAPTDTTRFI--------------------------------------------SKRGSIHSA---ASAWKNLASSEKVRTAAK-------------------------------------------NREFECICVRPFRSMN---------------------------------RSREFRLFINE----------------GNLILMSQYWLDRHYHKLESK---------------KDFYWTKAEE-LLDE-ISWLLP---------------------KETIVLD-LYFTS-------------------KDQIILIDFNPW----------------GPPTLPLLAE--SWNIDWTEE-IGIKI
667801279  ---SEHIPID---------------------------------SLMWRLNVALRQF--------------PEGAFVRLGSRSSKDSAYAL--------------------------------------------QHGLRVRTG---EAAVRMLTEGSERTAFDL--RLAAQ------------------------------------VHYAPHIFVRKWMDIP---------------------------------RWTEFRCFMKE----------------RRLVGISQYDCLTLGIRPEIT-------------RNAESLHTAIRE-FSQR-FAEASH---------------------LDNVVFD-VFVEGMDTSGP--------------LNVRLLELNPF----------------FPKTDPCLF---DWSVPADFD-GSFR-
496403701  -----SFKSE---------------------------------SIELHERDVIAIG-------------ASTDEFMSARGLQEKPFFSAQLH----------------------------------------DDIEYALSVLN---KPAFVRFGGVSYHDASLS--RLDTV--DGIV----------KQLSVS-NRCVASYLWDCLQSSTPVWLFLREWRDIP---------------------------------RWGEFRCFIRD----------------AKVIGVSQYHCLEYFPFLKEK---------------ENEIRLQLIM-FLQK-LLPVLH---------------------MNSVVAD-IAIDYQDGK----------------FTTTLIELNPF----------------IQRTDACLF---SWVNGGDFN-GRIRV
654084322  -----SFQSE---------------------------------GIELHERDVIAIG-------------ACTSEFMDAKRLLEKPPFSEQLR----------------------------------------DDIEYILSKFT---TPVFVRFGGVSYHEATIP--RTNNV--DDVI----------RQLSVS-SHRVASYLWDCLQSSTPAWLFLRGWHDIP---------------------------------RWGEFRCFIKD----------------GNVVGVSQYHCLEYFPFLTEH---------------ADEIRQQIIQ-FLQK-LIPLLH---------------------MDSVVAD-IAITHQNGQ----------------YDTMLIELNPF----------------IQRTDACLF---SWLNGGDFN-GRIRV
489732119  GIGSSEYQST---------------------------------NLEQKFDKLLSQF-------------NNQEVFMRLSTRSPKDSRHLFDEAATLMSNDY-------------FYWKDTDNKNQQLVSFVASMAKSMKITNG---KKIIQMIQESPRVQNDLF--ALLSS---------------------------------ESPSDCKTNIVLREWHNIR---------------------------------PDHEFRLFVSRRC-----------REESIVTAISQYFHFLHFDKAPGD---------CFNFLDESIKKSLILK-FQNYVLKSIDPAVARFLNFS-SEQDDDNSINCIREYIVD-LALVPISEYHGEVTDENKIEIGENTYILTVIELNPF---------------APSATGCGLF---NWQKDLDIL-WGKAP
654927668  GNILPDYDST---------------------------------KLESMFNNIISEF-------------NNKEVFMRLSTRSPKDSKHLFEEAATIMSKDF-------------VYWSENDNKHQQLVSFVASMLKSMKIKNG---RKIIETIAQSPRVYNDLI--ALVSS---------------------------------VDQSDCTTNIILREWHDIR---------------------------------PDHEFRVFVSRRH-----------RKESIVTAISQYFHFLYFDKNPAD---------CFNFLDEEDKKTVIKK-FENYVLKSVDPDVAKFLNFS-SEQDNDESADCIREYIVD-LALIPIHQYHGEVTDENKIAIGENIYVMVVIELNPF---------------APAATGCGLF---NWKNDLMML-WGKTS
504656984  GNIPPDYDST---------------------------------KLESIFNKILSEF-------------NNKEVFMRLSTRSPKDSKFLFEEASTIMSKDF-------------VYWNENDNKHQQLVSFVASMLKAMKIKSG---RKIIETIAQSPRVYNDLL--ALVSS---------------------------------SDKLDCTTNVILREWHDIR---------------------------------PDHEFRVFVSRRH-----------RKESIVTAISQYFHFLYFDKSPTD---------CFNFLDEEDKKAVIKK-FQNYVLKSVDPDVAKFLNFS-SEQNDDESSDCIREYIVD-LALIPVSQYHGEITDENIIQIGTNTYVMVVIELNPF---------------APAATGSGLF---NWKNDLMML-WGKAS
671590708  GNGSPEYDST---------------------------------LLEHSFDHFLSQL-------------RNQEAFMRLSSRSPKDSKALFDEAATIMSKDF-------------SNWNEFDNKNQQLVAFVASMTKAMKITSG---RKIIETIQQSPRVHNDLI--ALMST---------------------------------ASRSNCTTNIVLREWYSIR---------------------------------PDHEFRAFVSRRC-----------RKESIVTAIAQYFHFLYFDKAPTD---------CFNFLEEDTKKNLILK-FQNYILKSIDPAVANFLNFA-SEQDDDNSIDCIREYIVD-IALIPINQYHGEMTDDNTIHIGGSTYIIMVIELNPF---------------APSATGCALF---NWKTDLDML-WGKAS
519004236  ---ASTQPLI---------------------------------HLAHRLDAVIAKQ--------------NRACFIRLSSRSPKDSIYAL-------------------------------------------RNGLCIRDGA---QALAIILEGSERCAADLR--MALDY-------------------------------------HHPMAIIVRNWIDFP---------------------------------PWAEFRCFMVG----------------RCWVGASQARHLERIAYPPI-----------------ADYKSKILE-VLNASMKKIAAASP------------------IDNAAFD-LVFDSLQK----------------SNHAILLDANPL----------------LVSTDTALF---SSIADLDST-FRFRN
497965255  ---------------------------------------------------------------------------------------------------------------------------------------IEYALSVL---NKPA-FLRFGGVSYHDYA--RPRLETVDGVI----------EQLAVS-NRRVASYLWDCLQSSTPVWLYLREWREIL---------------------------------RWGEFRCFIKE----------------GKVIGVSQYHCLEYFPFIKEK---------------ENEIRLQLIA-FLQK-LLPVLH---------------------VDSVVAD-VAITYQDS----------------KFATTLIELNPF----------------IQRTDACLF---SWINGGDFN-GRIR-
557952578  ----SVLLPF---------------------------------SDFDKVDDAIEKL--------------GGSVFVRLSSLSPK---------------------------------------------------FFEPVQTK---EQVLSVLQSSERTRDELE--G--------------------------------------------STLFLRKYYDFP---------------------------------KDKEFRLFVRK----------------GKLRAISRYDPEAECQLSP------------------EFVRDKLTR-WFRCLCLEGLLS--------------------FEDCTLD-VVLWEEKK-----------EMSLFDDGIFLIEYNSYG--------------EDSVSGSCLF---DWEADWEIL-T----
327409947  ----SVLLPF---------------------------------SNFEKVDDAIQKL--------------GGSVFVRLSSLSPK---------------------------------------------------FFEPVQTK---EEVLSVLTESERTRDNLE--N--------------------------------------------SVLFLRKYFDFP---------------------------------KNKEFRLFVRK----------------GKLRAISKYDPEADCGLPK------------------EGVQQKVSK-WFRCLCLEGLLS--------------------FEDCTLD-IVLWEERR-----------ELSLFDDGIFLIEYNSYG--------------EDSISGSCLF---DWEKDWEIL-T----
585299522  -----VLIPFDNS----------------------------PFEIVEKIDEVIKKL--------------GGSCFIRLNSLSPK---------------------------------------------------HFEPVTSG---YEAATILYESERTRQTFG--LFR------------------------------------------NLVMVRKFERFP---------------------------------KEMEFRLFVRK----------------GKLRAISRYDPYCLAPLKNSA----------------EELQRIFQR-FFRCLQVESLVL--------------------FDLCTID-CVYWPEQI-----------NRSYFLDGVFLIEFNTFG--------------PDSISGSCLF---DWEADKQIL-YHGKG
284504410  ----SVLVPL---------------------------------SEKRKLGEAIERL--------------GGSVFVRLGSLSPK---------------------------------------------------FFEPVETP---EQVLQVLQESERTRDCLK--DGE------------------------------------------EVFFLRRYEDIP---------------------------------KNKEFRLFVCK----------------GKLRAVSKYDPEADCFMAS------------------EEVRDIISR-WFRNICLDGLLS--------------------FENCCLD-VVVWEERK-----------EESLYDDGVFLIEYNSFG--------------EDSVSGSCLF---HWEEDWETL-T----
311978298  ---TYGIIPE---------------------------------SLLKIIDDSITEI--------------NNLCFVRTDAYSPKDLVFEN-------------------------------------------KIDNLKVSDA---LTAIKLITDSERCCQKLF--SND--------------------------------------QIISKYLAIREYVNLD---------------------------------TNYEFRCFIYN----------------WNLRAICQSGFEYNSELHAKK----------------KIIRDSILK-FWNK-FESICP---------------------YSECTMD-IIYDNNFK------------NTLNDSCIMVIEFNSFG--------------PHMNADSGLY---DWDRDYILL-TKSN-
363540335  ---INNIIPD---------------------------------SLAKKITKTISKM--------------SCSCFIRTDAYSPKDLLHRE-------------------------------------------IIDTLEVTDA---ITALKLITQSERCSSKLF--GDD--------------------------------------KVISRYIVVREYVNYD---------------------------------INYEFRCFVYN----------------WRLTAISQSGFEYNPVLHSKK----------------NIIYQSIIK-FWDK-FSRICP---------------------YSECTMD-IIYNDKWI-----------DNTLNNSGVIIIEFNSFG--------------EHMNASSGLY---DWTKDHNIL-TRSK-
441432004  ---DKNIIPF---------------------------------NLVKEISNIILEL--------------GGSAFIRTDAYSPKDLLYNH-------------------------------------------TVSTLKVTNA---IDALQLVTKSTRCCSKLF--DINN-------------------------------------KIISKYLVLRQYIDYD---------------------------------TNYEFRCFIYG----------------WKLRAISQAGFEYNPQLHEKK----------------KKIYDLILK-FWDK-FSRICP---------------------FSECTMD-IVYNEKWI-----------DNTLGNSGIIIIEFNSFG--------------PHMNACSGLY---NWVRDYYIL-TQSE-
451927830  ---ANNIIPV---------------------------------NIVNKLSNIILEL--------------GGSSFIRTDAYSPKDLLYNH-------------------------------------------TVSSLKVNNA---IDALQLVIKSPRCCTKLF--DINN-------------------------------------KIMSEYLVLRQYIDFD---------------------------------TNYEFRCFIYG----------------WKLRAVSQAGFEYNSELYDKK----------------KIIYDLVLK-FWDK-FSRICP---------------------FSECTMD-IVYNEKWI-----------ENTLGNSGIMIIEFNSFG--------------PHMNACSGLY---NWVRDYYIL-TQSE-
441432002  --YPKIYNDE---------------------------------LKDIIDKLSSCWK--------------SGSWFMRLDACSTKDS------------------------------------------------VVELPFESP---ETIIASIVTSRRAINALT--DNIN-------------------------------------RNLNTRIYFTHYDKKWN--------------------------------SSRELRCFIR----------------KNKLTAISQYCWTNRDFFCEWT---------EEDLINLAKKINKLVN-NIIEELSNRIG---------------------TKDMVMD-IYLDD-------------------QNNLQIIELNNFG--------------YWLACGSALF---HWIKDYDKL-YNTD-
629674745  NRLYVEDVAS---------------------------------CIVEPVLSELQFP--------------PEGLFVRLAACSPKDG------------------------------------------------MKKDALHSP---DDVVLKIVTSIRARNAIR--QALDEGEEKEAD-----------------------------RPFELPLSFLPFDGRMG--------------------------------SVNEYRVFCPPG--------------GKKISGISQYQWHKPWKFASLPKHPFYPKGADNMTAKISERAEDIRK-LVMEELHERGEGDNDD-----------DVLFMKQGFTFD-ILYDD------------------LQDTCELVELNVFG--------------ARSPCGSCLF---HWVKDREVL-WGVEG
225681182  TRIAHIAAHFP--------------------------------KTMSDMPIDFVFR-------------PGKKWFCRLDLTNPTDE-----------------------------------------------LEQKLPVTSL---KELVWRLCTSSLAHGALL--IDVEQ---------------------------------------QPKLFLIPFDDTFR--------------------------------QSLEFRAFCPPG--------------SPHVTAVSQRHWLTPFPNGTIE----------NMIWLAKVVFQCANR-LRYEILEYSMKIPDRTVS----------HDIHTRGFVFD-FLLTP-------------------GMEGRLVDINGFG--------------AMGGTGSCLF---NWIRDAHVL-YGLK-
598011243  LHEREDFLIK---------------------------------ELEGPLQAALDRL--------------GGGCIVKTSSRSPKDAAARTGALPCILRESL------------AASPQALQDENEMLRVVCEAEGAALRFSSA---RAVIRAFVLSERIWQDMT--LALRH-----------------------------------PESFEQNIVVRRWEDIR---------------------------------IDMEFRCFVHA----------------GKLTAISQYAYQLYSSTLVSS---------------LDQAKQAIAD-YFESRLRAVLVQGG------------------FETCVVD-IALVP------------------PSWGAWVIEINPF----------------LPTTDAGLF---SWENERAVL-EGKA-
470375844  HAQSDALALA---------------------------------DLERKIDGLMGAF------------ARDGGAFVRLSTRSPKDAVISSERTLHEWQAELA-----------RQGPEAERDENARLVALIKASTAALKVRSG---REALALAKASERSNEDLL--LALEF-----------------------------------PHQWDMKIITRQWVEMH---------------------------------PAMEFRGFVCG----------------KKLTALSQYFHIVHFPALAAH---------------KDEIARRIQA-FFAERIVDLIP---------------------LDNYVID-FGIASATDDIVAD---------GGGGEVLVIELNPF----------------ND-------------------------
470392261  ---DDEAQLA---------------------------------QLEAELAHAMEEMKSSSARRELQGEEEEMSFFVRLSSRSPKDAGLNEGHPRIMEYLTEEL---------DRFGGLAAADPNQKTVAMQRAAGRILRVTDA---KQALWLIVNSERTFTDMV--HALGH------E----------------------------DGEWTMKIVLRHWMDGVD--------------------------------LANEFRGFVCQ----------------GNLTALSQYNDGNYYPELMGK---------------EELIVGKISA-YWQK-VKHQVK---------------------YQACIVD-FVLVN------------------NMQDVYIVEINPFVHLPPLDSIPSRPFMYGPVTGGALF---DWGTERPLL-QGECD
470526872  PTPEDASVLK---------------------------------ELESRLDALVAAV-----------GGQDKGCFVKLSSRSPKDVTTKGDKFAAIYSSLLDQRR----RSDSSSASSEEADKNARLGLLYRASTEAMRVHSA---AEGIALLLQSRRIYWDLT--YFLAL----NDG---------LGAA----DDDDEDRERDNVDVGDLAIVVRPWENIA---------------------------------LQNEFRGFVHG----------------GQFTALSQYFTQLHFPELVER---------------RSELEQRIVA-FWHRIQHAFAA---------------------YDKYVID-FAIVEDDQ---------------GNEKMVVLELNPF----------------NISTGAALF---SWESEDDSD-TLEGK
470392564  DARSAQRTLE---------------------------------PAHATVEEAIEAL--------------GGAAFVRISTLSPKDAVKWQSEKLKGLLEAE-------------LEGAAAGDEDAEIIAINMACCLACRVTNG---AEAMDLLIRSDRVDRHLA--TRREE------E----------------------------GDELSVNIVIRKWLDFR---------------------------------PELEFRSFVYD----------------RQLTAVTHYYKFCFVREAVEK---------------KEAIAQQIRS-FYEEKLRDTIP---------------------ASTYAID-FALLP-------------------DGQLIVVELNPF----------------APNTSPGLF---DWTKDEDVL-KGVK-
470426125  -----PEVIN---------------------------------ELERTIQNVIDTD------------FNGGAVFVRLSTQSPKDVGRGWTEHPRVVPIVREE---------LAATESEGRTLNDRIRALFAASLRVMKVESG---RGALDLLLKSERIQSSVL--HALAN----AA-----------------------------EARWDLCVVVRAWEESMR--------------------------------LDREFRTFVVR----------------DRVVAITQYNEYCHYPAWADQ---------------HQLIADKIHHLFVHQGLRDKVPRAY-----------------REWAYVAD-FVLLGEPEERAD-------------LRVQLVEINPF----------------GPGTGASLF---DW-------------
470440496  LLDEHSRVLE---------------------------------ELADKLQSVIQSP------------EFGGRAFVKISTRSPKDVVFHLPKFELTLRQVTEEWT-------ALYNLPDPMPESVEQAIMQRCGALCLSVTSG---AEAVDMLKTSKRIWVDLK--EALDT----PS------------------------------DDFAIKLVVREWLDIP---------------------------------NVFELRGFVRN----------------RRLTALSQYFSMCYFPWVSPA--------------QWADVKTRVLS-FFETTVAELLP---------------------VEDCVCD-FVVCE--------------------DRVMVIELNLF----------------GRTAGAALF---SWDTDQQLL-YGNR-
470519872  --------------------------------------------------------------------------------------------------------------------------------ALYTALHRAMRVESA---EEALKLLLISFRTLEDVK--KRLEY------R----------------------------ERTWSLCVAVRQWVDFA---------------------------------PAMQFRGFVYQ----------------GRLNALSQYFYDCYFPVLQRH---------------KDKIEASLVS-FWQS-FREKVP---------------------YKSYVVD-LAILPCDLDQAD------------PLPVRIIEFNPF----------------DYYTDAAMF---NWLADKQTF-REGP-
471191192  -YDADDLVIQ---------------------------------GIKTKLDEVVSQH--------------PPPYFIRLGTRSAKDAFSPDMKLRFEKILTDIYKESNDINSSSKNGTTLEKPTMKDVQNYYIGRFKLLKCTTT---DEMMDMLLHSERIRTDLT--RLLAL----PTI----------------------------DTKNFEVLALREWCDAVN--------------------------------PVLEFRGFVHK----------------GILTAVSQYNPIFYSDYLVKN---------------KALIQKVLRT-FFDEKFKPTFEEKKKTLP----------DVSRLTSYIVD-FAVTD-----------------VERGEVKVVELNSF----------------STFAGASMF---SWKKDIETL-FGTK-
471197419  -QEFDDAHLA---------------------------------VVKQKIDAEISQA-------------HWNGYFVRLGTRSPKDAFTEKMFFDFSKNLKMLFTARIESYHGQIPENWAKTFSVYDYMDYSKARFSCWKCKTS---EEALSLFTNSDRVLNDIE--RILKL-----EI----------------------------DPQKFEVLALREWCDELD--------------------------------PWYEFRAVVYK----------------DNLTALTQYDSRFVLDNVIQN---------------PKEVEDVVKT-FFTKEFKRNFCEKRDKLQE--------EERKRLESYVID-FAYLQ------------------KTKTVKVVEINSF----------------CSLCGVSLF---KWEKDILVL-FGKT-
290999655  ----RRGILL---------------------------------NLCKRVDECAVDL-----------PNREDGFFCRMATMSPKDAATNRLGFISLVWKHYNELL----KLEKEMNIDFKEQMNRNVYALYKASTSALKLNNG---MDAVQLLVESERAQQELN--KIASGVYG--------------------------------DATKTNELILREWCTFD---------------------------------VAHEFRAFICN----------------KKLTGITQYNPFVYFPQLIKQ---------------KEDLQQLMRN-FLEKDIINNPAIQ-------------------VSNFIVD-IIIVQDR---------------NGEKQVKIVELNPF----------------AEFAGTCLF---TWENDRDIL-EGKS-
290984195  ------KILN---------------------------------NLADRISSKMKEM---------EELSGRSGVFIRLSTLSPKDAAINRKGFVKLIHEEYLNILKE-SELLDDKSLDKSEKTNIIMYALYRASISILKIYNG---HEAIQLLIESKRAQQEFK--AMLDS-----------------------------------PTKSPLDLIIREVCDFD---------------------------------VAHEFRAFVYN----------------RKITGLTQYNPVVYFPTLFKQ---------------KDQIKHLIIS-FILENIVNNAELP-------------------ISNYAID-IILVKDMT--------------NGNLIVKIVELNPL----------------AEFTGTVLF---SWEEDREIL-MGDCN
290982785  --VVLLENVE---------------------------------KRIDECLAAHTEF--------------NDGAFVKLNTRSPKDVPWKCHNDESYQNQLNK-----------EVERVSDRTPNNICVAFLKAMNKSMKINSG---KSAMELLGRSQRIYEDLQ--KNTGFG----------------------------------EKLYESKIILREWNEEMI------------------------------ELPQFEFRCFVHE----------------KKLNAISQYFCDFKFDDLIAQ---------------KEEILKKINE-FFNGFCIERIP---------------------HPSFVVD-FFVSP-------------------TKGVTIIEINPF----------------HNGAGPPNA---DYDFFIIVS-NELYE
290973730  NSDTRRTVLE---------------------------------TLEIDVNREMKEL----------SCCNDEGCFIKLSCRSPKDAFAVCAKMKELFNDKIEK--------IVMEKKGVLATPNERLIAVNESFIQSMKVKDF---AEEYTYFTKSARVLEDLL--LFLKYDKKQR-------------------------------EENPIKIIIREWVDIP---------------------------------SKYEFRSFVKN----------------KQLTAISQYFDT----------------------------------------FAHRVP---------------------LNDYICD-FAIDS-------------------NGRVYIVELNPF----------------STTTDACLF---SWTKDGEIL-NGIQI
290974067  ESLKIELKLD---------------------------------ENIQKLMHEIDECSL------WKDEERREPLFFKLSSRSPKDVTKLNGKYVMELFHKDLK--------EHYPSEEAQLDNSNRVVSFTRAATEFLKISSS---DEVLELLLRSSRVNMDLI--ETYVEFWN--------------------------------EKSPLNKIALRRWDTTID--------------------------------IRLEFRTFIVN----------------GKLAAISQYNHFVHVEEIEQY---------------HNQIQQLIEK-SVKYLKENLDKHMIFK--------HMVADFVIYPRFFSD-IEKTNQWNEDLI----------PTGDYIKLIELNPY----------------EESTGACLF---SWKTDQQII-PMPPP
290995536  SPKDAAFNSQ---------------------------------KIRQILQRKLYEKN-------------AMFSFGGENAKIQSDQDKQNNEFIAF----------------------------------FESQVEVMKFESG---QEAIEMMTSSTRVYDDLN--IALKYRND--------------------------------DSLWNVFFVLRKWIPNHN--------------------------------IQYEFRTFVYN----------------RKLCAISQYNDALFFEDLCNH---------------KDLYLKAMLN-FFEK-IKDEIP---------------------FDNSVMD-LVIYPLSSDEEKLH-------DMDNLNVQVLEFNPF----------------NQYTGSAFF---SWIKDTEIL-KGEKP
291000324  ------KYLH---------------------------------NLKERLDQALLNE--------------TISYFVRCGPRSPKDSRGKLK----------------------------------HKPSLMVFTGGIPEISFS---TQVLNLLIESERVFKDIH--LYLENRTELLER-----------------------------NKYNFFVHLIPWRNFK---------------------------------KENELRCFFFK----------------KNLVAITQYDVQLNYPFKGKE-------------NICVKIIQKLMN-MHHGILKSVIP---------------------YENFVMD-VEIST------------------DTNSIYIIEFNPYG--------------KDGTTGPVHF---NWKQDENIL-FPETF
167385292  KKENWKSEIE---------------------------------WFNQIIERNVHLY---------------GGVFLKINGKALVDAEWMN---------------------------------------------GSLKVCNG---NEGMMLLQGSERAQELIE--KYRQE-------------------------------------GKVNELEIRKFEEIR---------------------------------ISDEFRCFVVH----------------RELIIISQRYNDAYEVKIQER---------------KKEIIKKVNE-LFEIIKSHHF----------------------SDCYTFD-VVINN-------------------KIKVIGFDEMNE-----------------RSFEGMTF---NKEELMSNA-KDINV
471191846  DDETFEVGHL---------------------------------WFDELIKKAIEKY---------------GEVFLRLNKVALLDSEWMN---------------------------------------------GSLSIHNS---RDALTLLQASERANISLD-------------------------------------------SHSPNELEIVQYVSIN---------------------------------PNQEFRCFVIQ----------------NTLCAIIQRYTDIFTSSIEKQ---------------KTQIVTAICS-LYDNMHQTNLN---------------------IENYTFD-VMVKG------------------DKATLIDADELDEY---------------HTKNTLEGF---STLDEIKEA-K----
330843241  KRVIKQTDFP---------------------------------ELLDKIKTAIEKM--------------GGTVIPKLNWSAPKDAIWMN-------------------------------------------TYNSLKCTTP---TDIFLLLKSSDYINHDLL--QYKIKEEEDDN------------------------------TTTPFVLVLRKWQNLH---------------------------------PSMEFRCYVKD----------------NKLIGISQRDTSTYFNFLKDK---------------KDKILNAIIN-FYDNSIKEKFN---------------------SSSFTFD-CYVTK-------------------DDKVWVIDFNPI----------------HPSTESLLF---LWDELFPEL-IEDDE
66819179   KRIVNETEFK---------------------------------ELSNQIIKSIEKL--------------GGNIFPKLNWSSPKDASWMN-------------------------------------------VYNSLKCTNT---TDIYLLLKSSDFINHDLM--QFSINQDDKDD------------------------------SLTPYVLVLRKWQNLQ---------------------------------PSMEFRCFVKD----------------NQLLGISQRDISTYFKFLKDK---------------KQKIQDAIVK-FYNESICGKFS---------------------NNSFTFD-CYVTK-------------------DEQVWLIDFNPI----------------HPSTEALLF---VWDELIPEL-IEQDQ
470261556  NRKISEKDFP---------------------------------ELIKEIEDAIAKL--------------GGEVVPKLNWSSPKDATWMN-------------------------------------------IHSSLKCLTP---TDVLLLLKSSDFINHDLC--QFQIEKDQEEI----------LKDD----------------SISPFTLVLRKYHNLF---------------------------------HSMEFRCFVKN----------------NQLIAISQRDTSTYYKFLQEK---------------KQHLQDLIQQ-FFNTIVKDKFD---------------------DINYTFD-VYITR-------------------DDKVYLMDFNPI----------------HPSTDALLF---DW-------------
281208675  EVKIDPQQLK---------------------------------SFTDKIDEAIKKL--------------GGECVPKLNWSSPKDATFMN-------------------------------------------IHASLRCSSS---SDILLLLKSSDFINHDLA--QFDNDIKDLQPD-----------------------------DITPLTLVLRRWANVN---------------------------------IALEFRCFIKD----------------NQLIAISQRDTSAFFDFLPAK---------------KELIQSKIKS-FAEQHIINKFN---------------------DVSYCFD-VCFLD-----------------TNLNTVTLMDFNPI----------------HPSTDSLLF---DWYELFPEE-LEHQE
158297128  DDDQEQPAFP---------------------------------EFSQLLTDAIQSL--------------GGNAFLKSDWHCPKDAQWIT-------------------------------------------LGQSLCVRDI---TDVYQLLKASSFCKEDFR--ERSEV------------------------------------NGSGYHVVLKKWRDIH---------------------------------PGSEFRCFVRN----------------RSLVAISPRHWPSYHEHIARE---------------RSDIVNDIVS-LFKEKIKETFP---------------------LKDYVFD-VYRPA-------------------KDNVIIMDFSLY---------------GKGHSDSLAF---DYDQLDDEA-QVATI
646693182  PSEVKQPSFP---------------------------------AFSKALKDGINQL--------------GGEVFMKLNWSAPVDAAWIT-------------------------------------------PTKSLKCTTI---EDIYLLLKSSDLLVKDLM--CSMQQQPP---------------------------------RSVNSCIVLRKWQNMD---------------------------------PGNEFRCFVCK----------------QQLIAISQRDYKVYYVHLAQS---------------KYQIMEEIAK-FFKEKISGNFP---------------------VPNYTFD-VVRHS-------------------SNKIQIIDFGLF---------------NPKRTMPLLF---TWGDLCAKE-RNLNA
665792272  AEESAQPTFP---------------------------------EFSKKIQDVIDDF---------------GAVFIKSNWRTPSDAMWVA-------------------------------------------VTKSLKCTSL---EEVYLLLKSSDRISRDLT--AVKELTSDT--------------------------------KVIPPCLVLKKWRDIN---------------------------------PCTEFRCFVVN----------------RELVGICQRDVTQYHQYIENE---------------KYSIQTDIKS-LFRERIKNRFQ---------------------LDNYTFD-VIRYK-------------------KDKVKILDFGPL---------------DESTTKGTLF---TYQELISSI-VEP--
607352919  EETEHQPTFP---------------------------------EFSQKIQNVLDEY---------------GAVFVKTNWSSPADATWVA-------------------------------------------PTKTLKCNTL---EEIYLLLKSSDRTARDIN--AVKSLRDHK--------------------------------NPLPFCLVLKQWQDIN---------------------------------PCTEFRCFVVD----------------NELIAISQRDISQYHSSNESE---------------KYNIQTDIKS-LFSERIKGRFP---------------------LRSYSFD-VVRRK-------------------KDKVKIIDFGLM---------------DESSAKSTLF---TYEELQNHI-DDT--
332373438  SDGEETPLPG---------------------------------EFKEKLKNAFNTL--------------GKTVFVKNNWHAPIDAKMFS-------------------------------------------TGNTLKAESA---DDIKLFFTTSTVIQKDLF--NIKGI---------------------------------------PFCLALRKWISIH---------------------------------PAAEFRCIVVN----------------DTLRGITSRDWPTFYAHFKEE---------------GSEIVENISD-FFLENIKAKFP---------------------RKNYVVD-IVLSY-------------------PDKPFILDFGPL----------------NSRTNLYAF---SWKEIGPLL-NKDFQ
556102499  GDDGQVPAFP---------------------------------EFEAQIQENIDKL--------------GGKVFPKLNWSSPQDATWIS-------------------------------------------FDKTLKCTCP---CDLYLLLKSSDFIAHDLT--EPFIHCEDCGNQ-----------------------------EPIQYELVLRKWMDIQ---------------------------------TGMEFRCFVRD----------------KNLIAISQRHHTQFYEFIGKD---------------SKDIVTDIQS-FYKENIQGKFK---------------------DDSFVFD-VYRQD-------------------KGKVILLDFNPF----------------GHVTDSQLF---KWKELTSET-VPDSS
524897036  -EGAKVPDFG---------------------------------DFDESVKEAIKSL--------------GGKVFPKLNWSSPKDANWIS-------------------------------------------FDKTLMCTCP---SDIYLLLKSSEFIAHDLD--QPFVHCDDAGDD---------SAEN---------------SPSISYCLVLKKWQPPD---------------------------------PSTEFRCFVHD----------------KKLIALCQRQATKFFSHINHE---------------RESIISDISK-FHQQKIAQRFS---------------------ETSYVFD-VVRPE-------------------QGKVILVDFNPF----------------GLVTDSLLY---SWEDIEGLL-KNMDK
221316620  TATLTAPEFP---------------------------------EFATKVQEAINSL--------------GGSVFPKLNWSAPRDAYWIA-------------------------------------------MNSSLKCKTL---SDIFLLFKSSDFITRDFT--QPFIHCTDDSPD-----------------------------PCIEYELVLRKWCELI---------------------------------PGAEFRCFVKE----------------NKLIGISQRDYTQYYDHISKQ---------------KEEIRRCIQD-FFKKHIQYKFL---------------------DEDFVFD-IYRDS-------------------RGKVWLIDFNPF----------------GEVTDSLLF---TWEELISEN-NLNGD
41152285   TTTVTAPEFP---------------------------------EFNVKVQEAINVL--------------GGCIFPKLNWSAPRDANWIA-------------------------------------------LNSSLQCQSL---SEIFLLFKSSDFITHDLT--QPFLHCSDDSPD-----------------------------PTINYELVLRKWSELI---------------------------------PGGEFRCFVKE----------------NKLIAICQRDYTQHYQHIGKQ---------------EASISTSILQ-FFRDNIQYQFP---------------------DEDFVLD-VYRDS-------------------SGRVWLIDFNPF----------------GEVTDSLLF---TWEELTSGK-NLTAN
225710208  SEEGPRPSFP---------------------------------EFSTALQEVLKQG--------------TGKFFIKLNWSSPRDAHWVS--------------------------------------------SSGLLCKTL---TDVYLLLKSSHFILHDLT--NPFKDCQKNSEE---------EKNA--------------ALQGSQYVLALREWSSIN---------------------------------PGHEFRCFVRG----------------GNLIGISQRDPTSFYDYILRQ---------------ETSIKRSILN-FFESHLWDFP----------------------LQSFVFD-VVSSS-------------------KGGITLMDFNPF----------------GPTTDGLLF---EWEELLNYE-EGTEL
321463239  TPALEAPHFE---------------------------------DIDKEITTAIAEF--------------GGKAFIKLNWSSPKDAAWIA-------------------------------------------LNNSLQCHMS---ADVHLLLKSSDFILHDLT--EPFKECEDQNVN----------------------------QTPVKYNLVLRKWVEIN---------------------------------PVNEFRCFVQN----------------KNLIGISQRDDTQFSPFIEKE---------------KEDIVRDIVS-FFKEQIRPKFH---------------------LDNYVMD-VFRQR-------------------KDKIVLIDFNPY----------------GVITDSLLF---DWNEDLLQI-SDTSE
115920254  GSSKVRPSFP---------------------------------DLQAKVERAINQL--------------GGEVFPKLNWSAPRDASWIA-------------------------------------------CGNSLKCHTF---NDIILLLKSSNFISHDLT--EPFTLCDETEQA-----------------------------GQVQYELVLRRWTEIP---------------------------------PSMEFRVFVGN----------------QEVIAISQRDCSSFFPCVPPL---------------VDDITYEINN-FHDRYVAGLFK---------------------EQHYVLD-VFRKD-------------------KAEFLIVDFNPF----------------NEVTDSSLF---TWTELYRMR-DGDEE
555694651  TDSETKTSLN---------------------------------RFVTDVDLAIKQL--------------GGQVIPKFTWSVPKDATWIS-------------------------------------------HDKSLKCINS---DEVLLLLKSSNQITHDID--YALGDGAAPEK------------------------------STINHVLALRKWVNVN---------------------------------PCLEFRCFVRN----------------NCLIAISQRNVQQFFPEIIEK---------------SHKIKSLIID-FINSEIKNKFF---------------------VENFTLD-VFLTD--------------------HIVKLLDFNPY----------------GHMSHSLLY---TYEELDKVN-ILNDR
443686486  EEDAERPHFP---------------------------------VFHESLLSAISAL--------------GGTVFPKLTWSAPQDATWVS-------------------------------------------FNNSMECKSP---SDIYLLLKSSTFVSHDLT--EAFRYCDDGP-------------------------------VPVSHSLVLRKWTDVD---------------------------------PSSEFRCFVFD----------------QTLIAISQRHTKAYFSHLAPL---------------NHQIIADIGN-FYRSEICDKFP---------------------LQQYIFD-VIWKN-------------------NGVVTLVDFNPF----------------GRTTDALLF---SWDSDLAQG-QISQL
156408387  QATVEAPEFN---------------------------------DIDTKIKEAIQEL--------------GGEVFPKLNWSAPRDASWIS-------------------------------------------HDNTLRCKSP---GDIYLLLKSSDTIDRVLC--DAFIHCEDNSTQ-----------------------------THDSFELILRKWQNIY---------------------------------PAMEFRCFVRN----------------NELVAISQRDISNYYHFLAEN---------------EDEICADILN-FYESKIAEKFP---------------------DTSYVFD-VYKYA-------------------DQKCTLIDFSPY----------------GVPTNPLLF---TWSELDTEV-VPD--
449671755  QDHTLAPCFS---------------------------------LIKNKVDKVIDYF--------------GGSVFPKLNWSSPKDAVWIT-------------------------------------------MDGTLKCSSF---NDICLLLKSSDFISHDLN--DAYSHCIESTLP----------------------------HSDDAFELVLREWVDLI---------------------------------PSMEFRVFVKE----------------RIIIGISQRHSSGYYSYLHTQ---------------KDILLQEIIR-FFNLKIKSKFL---------------------DSNFVFD-IVKLE-------------------NGCYKLLDFNPF----------------GEVTDGLLF---SWSELRSLS-PQSID
340372881  SSDAMRPSFP---------------------------------QLVKTIFESIQEL--------------GGSVFPKLNWSSPKDAAWIN-------------------------------------------CDNTLRCTVP---AEVVLLLKSSDIISDTIA--SLIDKSGQDS----------------------------------AFTLCLRKWINVH---------------------------------PSGEFRCFIKD----------------KRLIGVSQRQISNCYKHLLDS---------------IESIMTDISC-FFEDNHISNKFP--------------------LRNFVMD-VYRPC-------------------KGSVLLLDFNPF---------------DKAVTNPLLF---SWDELNRVA-PVLP-
260798456  -APTEAPEFP---------------------------------ELEAQIKQAIAHL--------------GGKVFPKLNWSAPKDASWIA-------------------------------------------LNNSLQCTCP---EDVYLLLKSSDFVTHDLT--QPFDRCEDEDTD-----------------------------VSVHYELVLRRWTNVH---------------------------------PGMEFRCFVKN----------------DQLIAISQRHHSSFFQYIHDQ---------------HDGIQADIVD-FYHTDIEKKFP---------------------DSNYVFD-VYRKK-------------------AGKLMLVDFNPF----------------CEVTDPLLF---TWEELTYRV-PED--
391345987  EGETAVPKFE---------------------------------ELSRKVMKKIRKL--------------GGRCFPKLDWSSPKDASWIA-------------------------------------------LNRTLSCSTF---IDICLLLKSSSITTHDLV--DPFEYAHPADPG---------KGDS--------------ENQSLKHHLILRKWVEIE---------------------------------PSFEFRCFVNN----------------RKLIGISQRETSQVYSHIAEQ---------------KDQIVTDIVS-FFSEYIENRFP---------------------LRNYAFD-VFRTK-------------------KDYVRLLDFNPF----------------CQQTDALLF---TWQELHGMG-AQEAP
209867682  -------SVS---------------------------------DFSSSLLDEFDA------------------VFPKLNWSSPKDARWVV-------------------------------------------VDNRLKCTNL---AEIFLLLKSSDFITHDLS--EPFKFCHDDRPD---------------------------LLPTIPYVLVLRKWSALQ---------------------------------PAKEFRCFVKN----------------NRIIAISQRDCENFYSHIGSA---------------AEEIRTNLCE-FFTEKIQKKFF---------------------SSDFIVD-IYRKD-------------------ADRLYIIDFNPW----------------GPMTDALLF---GWAELMDLA-AEVKW
290999565  EDDYQVPEFT---------------------------------SLQMEIASAIEEY---------------EEVFPKMNWSAPKDAQWML------------------------------------------SDSKLLRCQTV---SDVFLVLKSSSFVTHDVQ--QAYNNCTDYTPE-----------------------------KEVRRVLALRKWYDVN---------------------------------PSMEFRCFVKN----------------RNLIGISQRDISNFYSFLVSD---------------KDMYREKLVG-FWEKFIKNSFP---------------------LENYTVD-LYISR-------------------TGPVFIVDFNVY----------------GPPTSPLLF---ASFKKGHLA-SDSTE
15235584   DEPLNRPSFP---------------------------------ELEIEIRESIETL--------------GGTIIPKLNWSSPKDAAWIS-------------------------------------------PSQNLSCTCF---NEIALLFRSSDSLTHDLF--NAYDSCSDKVSS-----------------------------RPESFYLALRKWYPSLK--------------------------------PEMEFRCFVKS----------------NELVGICQREVTTFYPVLLNE---------------KDLLKGLIEE-FFDDKIRFEFE---------------------SENYTFD-VYVTK-------------------ERRVKLIDFNTW----------------CGSTLPLMY---TWEELEKIH-GECDE
302784068  EQQQQQPSFP---------------------------------ELEAEVEASIRRL--------------GGAVMPKLNWSAPKDATWIS-------------------------------------------SSKNLKCQSF---GDVSLLLKASDSIVHDLC--HAFDNCDDKPSS---------STG-----------------RPEELVLALRKWYDLR---------------------------------PEMEFRAFVRR----------------GALLGICQREVTGFYSSLVSS---------------KDSLRTAISG-FFENSLLGKFE---------------------LESYTFD-VYVTK-------------------DLRVKLLDFNPW----------------EGSTLPLLF---SWEELEAIG-DGSDE
612394185  TNDTNKSPSE---------------------------------SLLTAIAKAIKQL--------------GGSAHPKLTWSAPTDAIWLT--------------------------------------------QFSTKCLNA---DEVMLLLQSSDRVAHDLD-GSAYACCREDEDE----------DED--------EDEDKERHQQEFHSLTLRKHSVSLE--------------------------------LSREFRVFVVN----------------GTITGVSQRDVTSFYPFLVNE---------------KSKIGWTIER-FWKDEIRPSAWHRKICGDGSVASTEKRNAFSNKNGYCMD-VVLSN-----------------DNEKVRFIIDFNPF----------------GGATLPLLF---SYDELSNDD-GNEIA
552837040  AAPPPPPELA---------------------------------TLRQQLQAAIESL--------------GGRVVPKLSWSCPKDAVWMS-------------------------------------------PSASLCCANA---EEVLLLLRSSDRVAHDIC--HALQQAAGGAADGGADGGAEAGPSGGA--AASAAAGGGAAVPAVQHCLALRRWHDLQ---------------------------------PGREFRCFVRG----------------GELVGASQRDVTHCYSFLRDE---------------RRELAAALQA-FHARHIQGRFP---------------------HPHYTYD-AYVAA-------------------SGAVRLLDFNPL----------------RGTTSPLLF---SWHEL----------
545366653  DEPTNLPEFP---------------------------------ELLAKIQAAIDDL--------------GGCVAPKLNWSAPHDAIWVS-------------------------------------------SNKSLACTHA---DEVLLLLKSSDRIAHDIC--HAFDACADAP------------------------------AALPGFHLALRKWVPLR---------------------------------PEREFRCFVKG----------------RDLAAISQRNIMENAASLEAQ---------------KEDLLEAMLE-FFEEHVQQRFP---------------------KADYTFD-VYITT-------------------AGRVRLIDFNPI----------------GGTTSPLLF---DWAELPYAL-PNSAD
145345498  RDGARRATFA---------------------------------AFERAIEDAIEAL--------------GGEVAPKFAWSAPKDAAWVA-------------------------------------------AGNTMKCRNA---DEVVLLLKASDAVAHDLT--EAYGACEDYARG---------DGSE-------ESEEDRAVREHAASVLTLREWYDLN---------------------------------PSMEFRCFVKN----------------RNLVAASQRHVNDFYEFLVRD---------------KDAIEDAIAL-FWESNVSCTSWHDD------------------QVDYVFD-VYVTP------------------KTKKVKIIDFNVW----------------GGTTLPLLF---EWHELEAMN-RDRAE
308802928  ADATAREAFE---------------------------------NFTREIGAAIERL--------------GGEVAPKFAWSAPKDATWVT-------------------------------------------AGNTMKCRNA---DEVVLLLKASDSVTHDLT--EAYRACADYVVD---------EMED---------EEDRAVREHANTALALREWYDLN---------------------------------PSMEFRCFVKT----------------YNLVAVSQRHVNDFYEFLLRE---------------KEEIEEAIAE-FFENEISKHYT---------------------GRDYVFD-VYVTP------------------KTHKVKIMDFNVW----------------GGTTLPLLF---DWNELESRG-SDQDC
511010991  -DDSKIPNFP---------------------------------EVEKEIREAIYEF--------------EGAVFPKLNWTSPRDAAWIS-------------------------------------------ATQSLKCTSP---FDVFLLLKSSDFINHDLN--HAYEHCTDIEYR---------------------------ENQKIQFNLVLRKWYDLQ---------------------------------PSMEFRCFVKN----------------KEIIGITQRDM-NFYPFLLET---------------KQDIEQSIYE-FFEDVVRDGFE---------------------STHYVFD-VYIQR------------------SNSKVYLVDFNPF----------------SPTTDALLY---NWKELMSFD-VDKDQ
671694366  -QEHNIPKFP---------------------------------EIEQFIRDSVRQL--------------GGDVFPKLNWSSPRDAAWIS-------------------------------------------ATQSLKCNSP---FDVFLLLKSSDFINHDLN--HAFDECVDPPTS------------------------------QQQQHLVLREWHVLQ---------------------------------PSMEFRVFVKD----------------KEIAGISQRDM-TFYDYMAGI---------------KDDIEEMIYA-FFQDHIQSKFP---------------------TTSYVMD-VYVDR------------------PRHKVWLIDFNPF----------------SPTTDGLLF---DWTELIEFH-PLNPE
672824691  -EEAALPYFP---------------------------------ELEDEIAQTIAEF--------------GGEVFPKLNWSSPRDASWIA-------------------------------------------TTNTLKCHNA---ADIFLLLKSSDFIAHDLA--HAYEDCSDEAGE----------------------VGSGIRSRPENVVLILRKWFDLA---------------------------------PSMEFRCFVRD----------------NKLIGISQRDM-TFYDFLKDI---------------REELEEKIVD-FFEERIQGKFT---------------------DSDYTFD-VYITR------------------NRERIYLIDFNPF----------------AQKTDSLLY---QWEELL---------
6323244    QEVEPLIDFP---------------------------------ELHQKLKDALNEL---------------GAVAPKLNWSAPRDATWIL-------------------------------------------PNNTMKCNEV---NELYLLLNASNYIMHDLQ--RAFKGCVDGDDI-----------------------------KGLKFDLVLRQWCDMN---------------------------------PALEFRVFVKN----------------AHIVGATQRDL-NYYDYLDEL---------------SDTFKDLIDE-IVHDVVLPKFP---------------------DKSFVLD-VYIPR------------------PFNKIFIVDINPF----------------ARKTDSLLF---SWNEIAAIA-PPKND
629657643  PKLPPNQRFP---------------------------------ELHQAIKDKIAEL--------------GGAVAPKLNWSSPKDATWIS------------------------------------------PHQNTIKCTSP---NDIYLLLKSSNFITHDLE--HAFDDCTPPPPSPS-----SASASTANNTNNSATSSSSSSPPSFKPVLVLRSYFNPH---------------------------------TAMEFRCFVKQ----------------RNLVAISQRDL-RHFDFLRSL---------------RADVVARASQ-LFNHRLRFTFP---------------------EGSFVFD-VYVPEKDGSDDDDSG-----GGGPLGRARLIDINPW----------------APHTDSLLF---GWQELLDME-VPGP-
68470928   HNDNVFSQFQ---------------------------------DIQDKIDASIQEM--------------GGAVFTKLNWSSPKDAKWIM-------------------------------------------PGNTIKCQNV---SDVYLLLNSSDHIGDDLD--NPFSEVQKKKTI----------------------------PEKVDYELVLTKWQEIN---------------------------------PAYEFRVFVKD----------------HRIIGISQRDN-NKYEFLQGL---------------KSELNEKITQ-FVEDHVIPKLKSD-----------------TQLSKYIVD-VYVSK--------------------NDIYIIDINPF----------------SRKSDSCLF---TWVELLDKK-DKHDN
226293274  -EVDPAAEWQ---------------------------------EIHAQIKATIAEL--------------DGKVAPKLNWSAPKDATWIS-------------------------------------------ATNDMQCRTP---NDIYLLLKSSDFITHDLE--HAFDGCVSEEEQ----------QKQQEKTEITTQPGAQDSPPRIPYHLVLRKYVTLN---------------------------------PALEFRCFVRD----------------RKLLCLCQRDL-NHFDFLFGL---------------RDNLRDKIQT-FFDIRLHDTFP---------------------DRDFVFD-VYVPP------------------PHNRVWLMDINPW----------------APRTDPLLF---SWLEILQMK-GPGCE
575476564  -EDTPNPSFP---------------------------------TLEAHIISSITRL--------------GGRIFPKLNWSSPKDAAWIT-------------------------------------------FATTLQCTTP---ADIFLLLKSSDFIAHDLS--HAYEECVDFEPG-------------------------HDQDRPKEFELVLREWFDLA---------------------------------PSMQFRCFVHH----------------GDLVGMCQRDSGNYFEFLKLN---------------RNTIELDLCR-FFDSKISGKFP---------------------DPSYVFD-VYMNA------------------RTRNIWLMDFNPF----------------GPTTDALLY---TWQEILE--------
552934656  DENQPLPSFP---------------------------------DLEQQIWDIIDEF--------------DGSVFPKLNWSSPRDATWIS-------------------------------------------ATNTLKCNSP---SDIFLLLKSSDFIAHDLD--HAFDDCYYDNQS------------------------DSRRHRPNEFELVLRKWYDVA---------------------------------PSMEFRCFVKE----------------EELVAISQRDV-NYYSFLNDI---------------KEELETKIIQ-FFETHVQNKFF---------------------NRDYVFD-VYVTR------------------NRERVWLIDFNPF----------------GPMTDGLMY---TWEEILTAT-GPPSF
406698079  DEAAPEYHLP---------------------------------ALNNAINGVLRKY--------------PQ-VFPKLNLTAPRDAAFVLQ-----------------------------------------TSSGPLVCQRA---SDVYLFLKSSNLIHHDLEPERLYEGCEDAPNP-----------------WTATEEEKVQQKAAVPLELVLRKYISDLN--------------------------------PALEFRCFVRD----------------GVLVGITQRDL-NFYDHLQDE-------------KTQDKLRRGIRE-FFEDELASNPPIAAGSSSST------STTPPDNASYVFDIYVDTKGALGY---------GYQYEGDRFVLMDFQPY----------------RSTTDALLF---TYEDIRDIL-QKSRE
169858039  GSPPPTYSFP---------------------------------ELDTQIRACIQKY--------------DA-VFPKLNFTSPRDAAWVL------------------------------------------PASSPLKCTSP---ADVYMLLKSSDFISHDLEPSSVFEGCRKYEHG--------------------PEGKDEGSWPHYDLELVLRKWYAVN---------------------------------PSRELRCFVRD----------------GRLIGISQRDT-NHYDFLNEP-------------STKDRILTAVKT-FFETKVKPRIPN--------------------QLDYTFD-FLLTK------------------DLSRGHIIDFNPY----------------SPRTDSLLF---SYEDLRDIA-SSSPP
598038401  PEPATRFAFP---------------------------------ELDQRIREVIKAY--------------DGGVFPKLNWSAPKDARWIA-------------------------------------------PSPPLRCLTP---ADVYLLLKSSDFISHDVDPSQAYDGCLGEDSA------------------------------GLPLELVLKKYYPIQ---------------------------------ESREVRCFVRR----------------SVLIAISQRDP-NYYEFWNDS-------------ATQENIRQAVIR-FFDQHIQQCWEG--------------------PPDYVVD-LLLTR------------------DLARAHIVDFNPY----------------APRTDSLLF---TYPELLALA-ESNPP
635371235  HSDSRIQAIQ---------------------------------QIQYQVENQFKEL--------------KGKSFIKLNWSSPRDAKWIL---------------------------------------------STLQCRTF---DDIILLLKSSDFTTHDLL--YPYRACTDAASP---------ETCG---------------IANKAPCLVMKKWCNLH---------------------------------DSMLFRGFVLH----------------HALVAISQRHCEACYPFLALK---------------KDSLRALIKS-FFLKHLQPLTVAPAH----------------LDSDFVFD-IYIDK-------------------NDRVYLVDINVF----------------GEVTDPLLF---SYNELQSFN-RQEAT
325179898  DSNARIRAIQ---------------------------------QVQVQVEKQFKEL--------------KGKSFIKLNWSSPRDAKWIL---------------------------------------------STLQCHTF---DDIVLLLKSSDFITHDLL--YPYRFCKDATSS---------ETRG---------------IANTSPCLVMKKWCNLH---------------------------------DSMLFRGFVRH----------------HALIAMSQRHCEACYPFLSLR---------------KRSLRSLIEI-FFRKHLEPLTLEPA----------------HLDSDFSFD-VYIDK-------------------NDRVYLVDINVY----------------GEITNPLLF---SYKELQSL---QETT
574109612  -----EDLYPS--------------------------------SFVAAVKDAIHRL--------------GGRVFAKLDWSSAKDAKWIL--------------------------------------------ANSLCCRSF---ADILMLLKASDFITHDLT--QAYDGCSDVGTK----------------------------RRPDTFHLVLKKWCHLF---------------------------------DSMHFRCFVRA----------------KKLLGISQRNCTERYDFLASE-------------ATQDTLCDAIAA-FFESHLTTSQALP-------------------DPNYVFD-VYVDK-------------------DHKVHLIDINVF----------------GAVTDPLLF---SWDELKQPA-TAEDE
574465217  -----EDLYPA--------------------------------TFVRAVKDGIDRL--------------GGRVFVKLDWSSPKDAKWIM--------------------------------------------GNSLCCTSF---EDVLTLLKASDFIVHDLT--LAYDGCYDKDVV---------GKT-----------------RPDTFHLVLKKWCNFF---------------------------------DSMHFRCFVGR---------------HSKLLGICQRNCTQVYDFLASD-------------STQDAVCDAIET-FFEAHLSTSSVLP-------------------DPNYVFD-VYVDK-------------------DHNVYLLDVNVF----------------GAVTDPLLF---TWDELHRAL-VADED
299115353  -DEQDRPDFP---------------------------------DLHALLSDAIASL--------------GGAVFPKLNWSCPKDAAWVN--------------------------------------------GGSLKCKLP---GDVLCLIKSSTFISHDLN--HAFDACTGSSIS-----------------------------RPETFTLVLRKWCNLH---------------------------------PSMLFRCFVRE----------------RRLVGVCQRDCTSYYGFLEEE---------------ADRLSTLLEE-FFAAEVCKKFA---------------------DPDCVAD-VYVDN-------------------RSRVWLLDMNPF----------------SGVTDSLLF---DWSED----------
585104546  HSDSSPSFLY---------------------------------AFELALTEALAEL--------------GGQAVPKMNWSVPRDAAWIN--------------------------------------------GGTLKCETP---GDIMLLLKSSDRVQEDLN--RLLKTSAPPDIP-----------------------------FRFSPTLTLRKWATLL---------------------------------PFMLFRCFIYR----------------SSLCAISQREPSAFYPFLAAQ---------------ESRLSTLLYD-FFQHEGREGLTGFQRLFEHGEEETDMEMGGKEGLAVCVD-VYVDR-------------------RDKVWVLDVAPW----------------GPPTDSLLF---EWVE-----------
219115585  EVSNELFHFS---------------------------------ALNSAIDAAIRRL--------------GGLVAPKLNWSSPKDAIWVN--------------------------------------------GGTLQCKTA---GDVYLLLKSSDFCAFDIQ--HSWKEVRDGDDT---------SDET-----ATDCHG------AIPLQLVLRKWCNLY---------------------------------PSQEFRCFVRE----------------QELVAVSQRQHSQHFEHLVRD---------------QYLIRSLVVE-FFDEIIKPHSQS--------------------LNNYTFD-VYLDK-------------------KERVWLVDFNVW----------------GRRTDPLLF---TWDEL----------
567996516  LTDDQLAVIS---------------------------------SVKRKVEKVLQDF--------------GGKLFPKTNWSAPRDAAWML---------------------------------------------GSLKCTNF---EDIFLLLQASDFVVHDLT--QPYIGCSGENTE----------------------------NSPTESYLVLKKWCNFL---------------------------------DSMLFRCFVVG----------------RRLVAVSQRNCDEFYEFLPDQ---------------QDELCELLYE-FYKKNFRKAEGEFLF----------------PDPNYSFD-VYVDK-------------------RHRVYLLDINVF----------------GAVTDTLLF---SWEELLEFQ-TESPA
348667590  LTDEQLSIIS---------------------------------TVREQVERVLEDF--------------SGKLFPKTNWSAPRDAAWML---------------------------------------------GSLKCTSF---EDVFLLLQASDFVVHDLT--QPYIGCSGEKAE----------------------------SAPSESYLVLKKWCNFL---------------------------------DSMLFRCFVVG----------------HRLVAVSQRNCDEFYEFLPDQ---------------QDELCELLYE-FYKTNFKKDDGGEF---------------VFPDPNYSFD-VYVDK-------------------RRRVYLLDINVF----------------GAVTDTLLF---SWEELLELQ-KDGLA
669157540  --------------------------------------------FVADVKAAIASL--------------GGKVFVKLDWSCPKDAKWIL--------------------------------------------GNSLACVCL---EDMVLALKASDFILHDLT--AAYDECVDVSDD---------------------------RRRPETFHLVLKKWANCY---------------------------------DSMHFRCFVKD----------------RRLVGITQRNCGDYYAFLTDE-------------TTQDELCNAIGA-FYSRYLLHAEVV--------------------DASFVFD-VYVDK-------------------SHRVFLLDINVF----------------GAVTDPLLF---TWDELATWR-HDEDA
397566776  -DETTSYSFP---------------------------------SLTAQIQTALDSL----------NGDRDLGCMPKMNWSSPRDATWMN--------------------------------------------QGSLKCTKV---GDVYLLLKSSEFVTFDVE--RAFDDLAEDESI---------TGGY---------------PDCFSHELVLRKWCNLH---------------------------------PSMEFRCFVRG----------------HKLVAISQRHPSKFYGHLQPP-----------ADGSAHPSAEMISS-FFETYVRRRFADGR------------------VSCYVLD-VYLDS-------------------QERVWVVDFN---------------------------------------------
223995977  TEELKKYSFP---------------------------------SLTAEIQSALSVL----------GGTVNKGCMPKLNWSSPKDATWMN--------------------------------------------CGSLKCTKV---GDVYLLLKSSEFVSFDLE--SAWEDLAVESEDETSHDTKKNGMNNLAMHDRNNASNTNRIPHDFQYELVLRKWCNLH---------------------------------PSMEFRCYVYD----------------HELVGISQRHPSKYYPYLQPP-----------SDETSHPIVNIIQQ-FFDIYVRNRFAQGA------------------VHS-----------------------------QERTWIIDVNVW----------------GSRTDALLF---DWKELAEL-------
656186323  DDSFDWSAVE---------------------------------PFVEKLKALIAKY---------------QSVVPSINGVFLDDALWVA--------------------------------------------NMNVECTSA---REVLLLLKSSTCWQDLEG---------------------------------------------SQCHLTLYPGIYFR---------------------------------GRLQLRCFLFE----------------NELVCVEQIFVNENFGFLAKD---------------AQSLVGSLKS-YSPSVMGVLNKGG-------------------VRSAIFD-VTVST------------------KNGEIEMLNIYQF----------------GSLPDGLL-------------------
156088905  MEQFNWEVIK---------------------------------TFTDELRTSIDNY---------------GSVVPCVNGTYLDDALWVT--------------------------------------------NYTIECSNV---RELLMQLKSSTYWHDRAQ---------------------------------------------GSSHLTLLPGRHFN---------------------------------NHYQLRCFVLE----------------HELVCIEQLFVHENFEFMAKD---------------AHRLVTLLKQ-FSVPILNIDTP-----------------------NAIFD-VTISR------------------ELTDPRLVDIYPF----------------GAVADGLLH----LEDVYHFY-YTRVK
510904826  NFDFQDHKLS---------------------------------SGISQLKSAIVEF--------------NKGSLPCINGSYLLDGVWIL--------------------------------------------NNTNICRDV---RDLLLLIKSSTEWQTMEL---------------------------------------------DKIKLFLYRAKMFS---------------------------------KSTQFRVYIYD----------------FEIVVIAQLFLNQVYDYLLNA-------------DQRKAFQDRIVH-FYREHILGQIPP--------------------VRNFVLD-LYIVE--------------------EHVYLLDIKPW-----------------KYHPLILF---TWQNLYEFA-TCRKS
399218561  VISLSDPELN---------------------------------HPIIAISGVLRKH---------------AGCMVKYRNIFVLDGCWII--------------------------------------------NNSYTVTNE---RDVILLLKSSCKWRDCSY---------------------------------------------QEGELYLVERRKID---------------------------------PSNEFRLYYID----------------NSLVAISQLHSHIKLPYLQNP---------------NNRIEVVTNI-IQLNNEIQNIIREL-----------------RLQYCAVD-VALGS---------------------DEFIIDILPW-----------------GWNGPL--------------------
67607916   ----SRKCLE---------------------------------DINKSIRENTEFF--------------SMGFSPKFTWSAPTDATWIN-------------------------------------------SNRSICCRTL---EELFILLKASTKVSEDID--RAKE-------------------------------------GNISNVLLLREYIPTLN--------------------------------EMFEFRVFIGGCSL----------HSEYKILGISQRHICYYYKELSEN------------FKLRTNIKSCIME-FFLYSKKELISELFDIF--------------NSMCIAFD-VYISN-YKDKL---------------SILIIDVQPL-----------------LHASPLLF---NLFELKLNL-LTEEG
209878444  ----SKESKI---------------------------------FLERLSLAVNKYS--------------KSGCVPKFTWSTPTDSCWIL-------------------------------------------PSSTIRCENY---SDVMILLKASTKVSEDLE--RSQN------------------------------------QKDFQHAILLREYIPTWD--------------------------------SSMEFRIFLSKASQ----------NASFRINGISQRHISLYFEELVSN------------KELQNGILSSINS-IVKCDERELINELFEAL--------------KTKKLAID-VYLIKTSTKYKF--------------RSLIVDVSLM-----------------YNIDTLLF---SFSELK---------
401407811  --------------------------------------------------------------------------------------------------------------------------------------SEDEGNCGR---SSLSSESKVFGKRGVDTQ--STGQKGGNPVC---------------CSAPLPVSAMAWRERVTVLDELTLVETKRLE---------------------------------EGMEFRCFING----------------GQVVGVSQRYLQDYFPFLVNK------------PQLQARVKRAIAA-FVECAVLGSEASGASKNTL--------KKQPFLRRFVID-VYVQRRQKREEGRTAG---EELPGRFKCWLLNVLPW----------------GSKTEPLLF---TWDELRTLS-YTSP-
237837343  --------------------------------------------------------------------------------------------------------------------------------------PVEGCAGSE---GEKTSVSRAFKISNEKDE--AEEQSIVYPHV-------------------AFGAASEWRERVRVLDELSLVETKRLE---------------------------------EGMEFRCFING----------------GQLLGISQRYLQDYFPFLVNK------------PQLQVQVKRSIAA-FVARAVLGAEAN-SGKNPV--------QKKPFLRRFVVD-VYVQRKQKREAKSAS----EELPGRFKCWLLNVLPW----------------GQKTESLLF---SWEELRILA-YTSRV
221055373  QNLFKSEQFR---------------------------------SQLEEIQEAIESL--------------NGSVFLRVNNKNLRKGSFVN--------------------------------------------NFSLEVNTL---YDALLMLKSSTDVYKVLK--QKE--------------------------------------TNRDNYIILSKYVNLN---------------------------------LCFLFDVYVYS----------------NSIVAVSQKCLNYYFDFLSKP-------------DVIEEIIQTIRI-FFEKHIKESFP---------------------QDHYILQ-LYIHT-FKRTKK-------------KKVLLINAKRW----------------LFKNKHPVL---TNKIFTNYL-FTDGV
457870279  RNLFKSEQFR---------------------------------SQLEEIQQAIEAL--------------NGSAFLRVNYKNMRKGSFVN--------------------------------------------SFSLEVNTL---YDALLMLKSCTDVYKTLK--KKE--------------------------------------TNRDNYLSLSKYVNLN---------------------------------LCFLFDVYVYC----------------NSVVAVSQKCLNYYFDFLSKP-------------DVIEDIIHTINF-FFKKHIKDTFP---------------------QDHYILQ-LYIHS-LKKTNK-------------KKILLINAKSW----------------LFKNKHPIF---TNKFLTNYL-FTDGV
71029258   RFDFNSPEFL---------------------------------DFFSAISESIKSF--------------GGLVLPYLNGSYLQDGSWII--------------------------------------------NNTTVCTDL---RDVILLLKSSTKWHGDSD--S-------------------------------------------ILKLFLYKVINQN---------------------------------HSTQFKLFYYD----------------REIIIISQLFLNHVYDSLISN-------------KHSHKLIRRILE-FSNHKLIHLIPEN-------------------LSNFVVD-LYILN------------------RTDEIYIMNIVPF-----------------NFNSEIIF---TWDQIYEYA-TSRTP
403223783  VFDFSSGSFA---------------------------------TFFNKVTETIKEF--------------GGLALPYLNGSYLQDGSWII--------------------------------------------NNSTVCNDL---RDVVLLLKGSTAWQNELV--NTPVASEVRDKH--------------------------------ELKLYLYKIPHFV---------------------------------KSVQFRLFVHE----------------AQILLISQLFFNNVYDYLLKE-------------EAFSELYEQIVK-FYNHNLVRRLPKD-------------------VSKVVVD-LYIIN------------------RTDEIYVTNVSPW-----------------YLNNENIF---TWEEIQEYV-TSERP
71756087   GIDGNPPRFD---------------------------------EMVQWVIDALKDIE-------GCGGAASDGVVLCGRWVVADDCSWVV-------------------------------------------PSRTPILHSP---RDVFLAMRNSPKFLRDVH--HQILSGDTSSAG-----------------------------MGSTVELTLAKAVGKN---------------------------------PANDFRVFVPYRLVRAEDTFRVTIWEGRVFAGVCQRSTDVCFPSLMSW---------------DLRTHSENYD-HVLRHIE----------------------------------------------------------------------------------------------------------------
342185981  NDGGEPLCFD---------------------------------DVVQWVVDALRTVG-------GCGDGAASGVVLCGRRVVADDCCWAV-------------------------------------------PSRTPALYSP---RDVFLAMRNSTKFLRDIH--HQTDCVRVGD-------------------------------GEATVEIALARVVGEN---------------------------------PANEFRVFVPYRLVKTGDTFSITKWEETAYIGVCQRATDVCFPSLMAW---------------DSTVHSANYD-YF---------------------------------------------------------------------------------------------------------------------
554940128  DSPDPVPCFD---------------------------------DVVQWVTDTLHEI-----------GGHGDGVVLCGRSVVADDCGWAV-------------------------------------------FSRTPTLLSS---REVFIAMRNSSKFIQDVH--HQLRGGASAAS------------------------------GPVKVEVTLAKALGRN---------------------------------QALEFRAFLPYRLHTSAGEVRAEAWEDHMYAGISQRATDVCFPILMSW---------------DEATHDENY-------------------------------------------------------------------------------------------------------------------------
657139967  GDTDAVPRFD---------------------------------DVVQWVIEALSAV-----------GGDGAGVVLCGRCVVADDCGWAV-------------------------------------------LSRTPALHSP---RDVFVTMRNSSKFLRDVH--HQLRGEKSGEEA------------------------EEAASAPVQVELTLAKSMGGD---------------------------------PVKEFRVFLPYRLYTTDGEVQVELWDDHVYAGISQRATDVCFPALMAW---------------DVERHDESYA-VVM--------------------------------------------------------------------------------------------------------------------
340059086  CGVASSLCFD---------------------------------NEVQWVIDAIRDL-----------DGDASGVVLCGSRVVADDCGWAV-------------------------------------------SSRTPALRTP---REVFIVMRNSSKFMRDVY--FQLREVGDADAG-----------------------------GKGGVMVTLAKALCEC---------------------------------AANEFRVFIPYRLRVSAGAPQLPMWEGLAFAGVSQRATDVCFPSLMAW---------------DSDTH-----------------------------------------------------------------------------------------------------------------------------
123414063  -FIIEDGKFK---------------------------------DLEKRVNDAIDEL--------------GGVAFPKLNFTAPTDSNWIG-------------------------------------------FGKSIEVRSF---KDLVYVFKASTRMLIDIT--CPFNVQN----------------------------------VEIKPIIVLKKWFNYK---------------------------------VNREFRIFMRD----------------SEHFFVSSRNISVPYYL-------------------TQEEVREGAQ-TMVNLISQKIH---------------------PYRIIID-FYISP-------------------KMRPHVIDIAPW----------------TDVTSPLLY---SWSELELLA-QTEVR
559180405  ---VMTSVNA---------------------------------PALHKFCENLDLL---------------NGKFLKVDWAAPTDCSQI---------------------------------------------VETLMCIVP---SNAFILLKMSEKIRNAIL--NFHNEDAPKVFD-----------------------------PDVPHYLYVMPYERLH---------------------------------KIFEYRVFVKD----------------LRIVLISPRYQCMITDV-------------------TTDIVRFVKV-FLLEHIIQHMD---------------------VTDLIID-LYCDVDEEICS---------------SILLLDMQPL----------------PAAESTL--------------------

R2K.2
521964427  -LLNQQYVMM----------------------------------PGVEAIRQRDWLFS--------VFGREEQVFVRPTSCLKL--------------------------------------------------FVGRCVDQA-----AFATALAPTRYD-------------------------------------------------PATLVVIAAPQPI-----------------------------------DREWRLVVVG----------------DRVISGGQYAVNGAR-------------------SITPDCPAAVKS-FAESMLAEVK-------------------WRPDPVFMLD-VCESAG--------------------QLWLVELNSF-------------------SGSWLY---RCDLPAVVA-ATSEL
551312310  -LLNQDVIFT----------------------------------TISEVLNNPSIIKQ--------L--KTDKLFARPDSHLKE--------------------------------------------------FSGRIIPTE-----GLTPVHFDYGFYHEN----------------------------------------------INLPIVLAPLKRV-----------------------------------EKEFRFICVD----------------HKIVTGCEYVADGRK----------------GGRTINIENKEPAYL-FAQKIADDN--------------------SYYHIAYVID-VCISNG--------------------KFYLVEMNPF-------------------SGADFY---NCDAKKIIE-AIEAF
656023071  -LNGADALFL----------------------------------TIRDIPEQLADDGR--------------SWFIRPVDDSKE--------------------------------------------------EPGNVKSTD-----EIRRMAENVIALKEDEIPNGS--------------------------------------LRHDTLLMLTEPVRI-----------------------------------LREWRLWAIN----------------GKIVTWSLYKEGSRV-------------------VYRHEIDEDALQ-FAQDMVDCN--------------------AGYSPAFVLD-ICRTGQ--------------------GLKLLETNCI-------------------NAAGFY---AANLLKLAS-AVDGL
495944056  -LLHRDWKCL----------------------------------PANELIAHAADVAA--------EIGCKDRMFVRPNSPLKP--------------------------------------------------FSGRVLDVP-----SISLKTLDHGFYYE-----------------------------------------------DETLPVVVAPVVD----------------------------------VGREWRFVVVE----------------GHVVVGSGYDAETRL-------------------GAASGRDVEARE-LAVEVASSM--------------------TPPCPVYVLD-ICQVDD--------------------EHRLIELNPF-------------------GGADLY---NCDSKAIVE-AVSEY
501005901  -LLNNNGLII----------------------------------NSSKLTNLNIKQ----------------EMFVRPITDTKE--------------------------------------------------FTGGVFSLS-----EICKLAD------------------------------------------------------KNIEILISKPIKN----------------------------------IEKEYRCFIID----------------KKVISATLYKMDNKL------------------FTLNNDKNIELLK-FAESIINI---------------------WIPNNSCVMD-IAYVND--------------------CYKIVEFNNL-------------------NGSGFY---DCKIENILT-ELEIL
488791998  -MLSSEGEVL----------------------------------IFKQLRERDYPQDT--------------RFFIRPNADSKA--------------------------------------------------FAGMVLTFA-----EIKSWYDKVVQNEAETVN-------------------------------------------ATTEIFFSTAYHI-----------------------------------EKEWRNIVVN----------------GKVVTSTAYRKSFEL------------------YKSATDVPPEMIR-FVEERCRE---------------------YQPHDIFAMD-VAKCSG------------------EHEYYIIECGCA-------------------NSIGFY---HCDIFAYVK-HIGEF
494035823  -MLNFDSVVS----------------------------------SVRDAAFTTE------------------SMFVRPVNDSKY--------------------------------------------------FSGRIFTAQ-----EFGAWQRSICQPEADRRTSLA----------------------------------------PQTRIQLAPLVTI-----------------------------------HAEYRFWIVK----------------NEIITQSLYRLGGKA-------------------TPAREVDEKFAS-FVNERIRQ---------------------WTPHETFVID-VCDTPG--------------------GIKIVEVNTL-------------------NCSASY---AADVQRLVL-ALEQA
414087296  -FLNSDAVFS----------------------------------TLSTLQKPEWK-----------------NIFIRPVYDTKK--------------------------------------------------MIARVIPNV-----NFDFAIQLYSAYNK-----------------------------------------------AELEVMIADAKDI-----------------------------------IAEYRLFIVH----------------GKVVAHSLYKLRGEV-------------------RMQPKVPESIIN-IAESLCNQ---------------------WVPAAAFVMD-FAETSE--------------------GFKVLEVNNI-------------------NCAGFY---ACDIPSIVE-AFRD-
489365992  -LLNKDFMVG----------------------------------DLWELNPIDE------------------EFFIRPTGNTKL--------------------------------------------------FTGQTVTRE-----EFLAWQKRECKEDSP----------------------------------------------YLGQKLMISPIQE----------------------------------IQAEYRFFVIN----------------QNIVTSSCYKENKVM-------------------STVRKPSDELLS-YTMKMINQ---------------------FPLAQAFVID-VAETNN--------------------GFKVVEYNNM-------------------NTSGLY---DCDEILLVQ-AINEI
601089719  -MLNQHVRQM----------------------------------SVRECMAAMEHEPD------------DSVWFVRPLHDLKH--------------------------------------------------FDGTVTVAR-----EIRRWMTSVDSGNFSFD--------------------------------------------GDTEVIVAPPQKI-----------------------------------HAEWRYFIVD----------------GTVVDGSSYRIAGQR-------------------MANAVARPELYE-QAQALADG---------------------WLPHRTCVMD-VAQTDD--------------------GFKVIEFNTF-------------------NSSGFY---AHDIEKIVA-AVTAH
494031937  -MLNVDARFL----------------------------------SLEELAADAELANS------------EALRFCRPSADSKL--------------------------------------------------IAGQVDSGA-----ALVAWARRVLALEDASMD-------------------------------------------PDTKLMLAEPVGL-----------------------------------SDEWRTFVVD----------------GRVVAGSHYRSYQRL-------------------EVSPDLPGEVVA-FTEALLAR---------------------GSPAPVVVVD-IARSGS--------------------GLYVVEFNGF-------------------NSSGFY---AAELSAIVA-AVSER
492085198  -LLNPRPVFV----------------------------------PAGLFTRSVDHYFD---------LLGADQIFMRPDSGAKV--------------------------------------------------FSGLVVTRR-----CFEQEIGALDQLTGIT---------------------------------------------PQTMVMLSPVRPI-----------------------------------ESEYRFVIVR----------------GEVVAGSRYMMNGEI-------------------SLSGEIDDRCLT-LASEIAGL--------------------PRQVDLAYTCD-VCISQD--------------------RARIVELNAF-------------------STSGLY---ACDRNAVFQ-AVAKA
654643796  -LLNADAKMY----------------------------------PIESNLAQVFDR----------------DMFIKPSKDEKA--------------------------------------------------FDAGILAAG-----QTIEGFIASQPQARNW---------------------------------------------REEMAVIAPCKTI-----------------------------------IAEYRFFVVN----------------REVVTGSLYRFAGNA-------------------NFSAHIPGNVMA-AATEFAPL---------------------YQPHAVFTMD-LAETPD--------------------GIRIVEYNCW-------------------NASRMY---AADVATIFH-TVHEF
495144986  -ALNSSAICT----------------------------------SLAELPRTWD------------------RFFIRPTLDSKS--------------------------------------------------FAGTVMTWQ-----ELEEFRDGVARVSNAPDVTLR----------------------------------------PGDRVVMAPLTEI-----------------------------------EAEFRLFAID----------------GVIVTGSRYKVGEHL-------------------SVSGEVPSAVKE-FAQACVDD---------------------WMPNKAFTID-VAITGG--------------------QMKIIELNSA-------------------NSAGVY---ACDVGVFVD-AVNSR
414088380  -LLNDDMERM----------------------------------PLSAAGAYFAALTP------------GEKRFIKPNLDTKE--------------------------------------------------FAGQVITAD-----DFDVWLAGMIDTGYLTKD-------------------------------------------SDFDVVIAAPKDL-----------------------------------GVEWRAVVVD----------------GKVSSCCIYRQWQRV-------------------MPELHILPEVED-LILKAHAK---------------------FAPGDVYVID-VAQQYQMING------------ERDYVFKIIEYNTF-------------------NSAGLY---ACDVVKIID-DINAF
652981718  -ALNSHSSLM----------------------------------CLREAREMVLAG---------------ESLFLRPDRADKT--------------------------------------------------FAGRVFTRK-----DLDLLPDD-----------------------------------------------------PDLAVIASAPREI-----------------------------------FAEYRFVVVD----------------REVVTGSQYARAGKL-------------------DVRVDVDPACRA-VAKQAACL---------------------YAPVPAYVCD-VAETPE--------------------GPRVVEYNSF-------------------SAAGLY---ACDASAIAD-ALAQH
189427115  -LLNYWAEVR----------------------------------PFSQMKDRVWDV----------------PMFVKPTDDGKA--------------------------------------------------FAGLVLEGE-----SLSQALEKQTHQVIA----------------------------------------------DDQPILVSDCKTL-----------------------------------GREFRLFVVD----------------GYVVDISEYRNRGQI----------------QHKDVAPRLKADLRD-YFQSVCHP----------------------SAPRAYVMD-VGEVWKN----------------GEWRWCIIELNCF-------------------NCSGAY---TVDRAAVYG-AVANA
654660594  -LLNYHTEVK----------------------------------ELSAILDWQDKE----------------QKFIKPYKFAKL--------------------------------------------------FTGSIFTKI-----KWENFVENSLQNPNNPLLD------------------------------------------SKSLVQISIPRDI-----------------------------------IKEARLWIVG----------------GKVIEAVYYKILKDI-------------------PFEENVEKEGIE-FAERMISI---------------------FNVAEAFVMD-ICLTDI--------------------GWKIVEINCI-------------------NSAGFFP--NADVKKVFQ-ALNEF
493683809  -LLNNKYFIL----------------------------------SLGELVRRKTEILEY--------FKSGGDLFIRPDSNMKS--------------------------------------------------FRSGVFNLN----ILNTMQSLGSELKR------------------------------------------------DETTLVLVSGKRG----------------------------------ITKEWRFFVYK----------------NQIITGSLYLVGEER-------------------VDETIRGGYLEN-YLSEVFKQVS-------------------WYPESVYTID-ICESEG--------------------ELYVLELGSF-------------------SCAGEY---ACDLSAIVEFGAKAA
669404879  -FLHREIRSL----------------------------------SAMELVNLIQSSKK-------------SDVFAKPKEL-KL--------------------------------------------------FTGKVFSFN-----SNIDELLNLP---------------------------------------------------PDTPLLVSPVKKF-----------------------------------LCEYRIYIIN----------------HQIVASCRYDDNPDD--------------------DLAIDMGVVSS-AIDELQRNS---------------------FDPVAYCLD-FGLLSN-------------------GKTALVEVTDG-------------------YALGRYR--GINDSDYFK-LLKLR
663682964  -YLRRQVKLL----------------------------------PAGSVIGH---------------------WFIKPTTT-KA--------------------------------------------------FTGFVVDTL-----GNPEHLNGYDRIQYQAFLSLP----------------------------------------PDTPVWVGEPVSW-----------------------------------LSEYRYYVLD----------------GQVRGEGRYDDAPDD--------------------MPAPDLDLVRE-MAALMASGP---------------------NAPAAFSLD-VGVLNS-------------------GETALVECNDA-------------------WALGLYK-GTMTRADYIE-LLWRR
544728994  -LLYRKVSKL----------------------------------PSLNDAKLLVRNGK--------------KLFIKPAGW-KR--------------------------------------------------FTGFVVEFE----DDIRFNGAS-----------------------------------------------------GRQPVWIADPVRF-----------------------------------VSEWRAYVAG----------------GQLLALCLADHGGDV--------------------NAIPNRTIIEH-AIQRLTATL---------------------GTPAGYVID-FGVLDS-------------------GETALIEMNDG-------------------FSFGAYA--GVTPEAYWA-VTVSR
492083916  -LLHRKVAKL----------------------------------PLREVLNDIERGT---------------VCFVKPADRPKL--------------------------------------------------FTGYVLRPG----DDYPIKHVP-----------------------------------------------------LREMVWRSEVVKW-----------------------------------VSEWRFYVVC----------------GQIQAKACYSGDAC----------------------VVPDEAVVVA-ALDRLAATG---------------------TYPRHHAID-FGVLDN-------------------GCTALVECNEG-------------------FSIGAYE--GVPASVYFE-MLHGR
502624283  -YLGRKIKKI----------------------------------AINNAEIFMNENS-------------QQRYFIKPVID-KQ--------------------------------------------------FDAFIAEYL----QDLARLKHFD----------------------------------------------------KKTKIWISEYMKF-----------------------------------ESEFRCYIYN----------------KKPLAICKYRGYAV----------------------GEIDYSIVLE-MAETYVSA------------------------PVAYTLD-VGLFES--------------------KLYLVEVNDA-------------------YSVQNY---GLAVDKYVA-WLMLR
333798257  -YLGRKVRLD----------------------------------YAFNAKPT---------------------DYIKPRGT-KE--------------------------------------------------FTCDVKSEV-----EKTRDL-------------------------------------------------------TIEMVWISEPISI-----------------------------------NQEWRLYILN----------------GELVGWSRYDDNEGD--------------------DIQPDFSVVSD-MIKDYKN-----------------------NAPISYTLD-LGISDE--------------------KTILIEVNDM-------------------WGTGFYPWGTMTGEKYLE-CIQAR
496027364  -FLGRRIWKD----------------------------------TINSISRNEEKWSA--------------GYFVKPRRN-KA--------------------------------------------------FTGKIISGI----SDLVGCGNYS----------------------------------------------------EDYEVLVSEPLDI-----------------------------------CAEWRCFILY----------------DEIIDVRPYGLLLDRNRKS---------------YNYHYDSNILNS-MMEAFVSWE---------------------ERPVACSMD-ICVTKD-------------------GKTLLVELNDS-------------------YALGCY---GLASIFYAK-LISAR
516256638  --SNRVSQCM----------------------------------TLGEALEKRERENK--------------PLFIKPKNM-KV--------------------------------------------------FTGFVHEGY-----SYRCLEDVP----------------------------------------------------LDVRVLVYPVFSS---------------------------------PIVSEWRAYIHR----------------YEILDVRNYSGDVF----------------------VFPDKDYLDR-VVRENKS-----------------------DFPSTYCID-IGVLES-------------------QEQVVIEFNDM-------------------WAIGNY---GIPNWLYVK-ALRDR
488720645  -WIGRDFWIS----------------------------------TLEQVRQPFENEEE------------TRAVHVKPLHEHKK--------------------------------------------------FKGTVFHKF----SDLIPSAVVA----------------------------------------------------GETEVLVQEVVEF-----------------------------------VSEWRAYIFR----------------GRIKFVANYRGDPL----------------------VFPDPSRMQA-ALDAFENR------------------------PVGCSMD-WGITST-------------------GDTLLVEVNDG-------------------YALGNY---GLDGYVYTA-IIEAR
494527233  -FLKRTVRLC----------------------------------RGRDLTSTDT------------------PAFVKLADA-KVAE------------------------------------------------LPAGSGTED-----GLRQSLVALDA--------------------------------------------------LDSWTIVSELIDL-----------------------------------VEEWRVLIVD----------------GTAIDSSQYVRASRSIE------------------TKLPTFRDIEE-FASAAAQSDP-------------------GHLPASYMLD-VGITTT-------------------GDLTIIEANPI-------------------WCANWY---SLSTENVKQ-ALQCE
503987592  -WTKRFIQLK----------------------------------QIDQLKESDF------------------PVFIKPVIP-KM--------------------------------------------------FIAGIFQTL----HDFKQAVSGLT---------------------------------------------------DREEILVSTIVNN----------------------------------ITAEARCYIKG----------------NTVKDLALYEG--------------------------AADLSTGRQ-FVSAFLEENK-------------------AGLPEVVVID-IAHSED-------------------TGWFILEFNAC-------------------WGAGLN---SCEAENVID-CIIGA
494155548  -YTLRNITYD----------------------------------RFKNIKNVQN-------------------KFIKPVDF-KF--------------------------------------------------FPAGVYTSV----EEIQGYSTID----------------------------------------------------QNIEVFVSDVVSW-----------------------------------AIEVRCFVID----------------NTIQTWSTYIYNGEI---------------QLKNSMEKHEENAMLN-FLEEFLSDES-------------------IHLPEAVVID-VGYIPE-------------------KGWALIEANPV-------------------WSSGVY---ACDPKKVTQ-TIVRS
491091253  -FRRRNTVLT----------------------------------TLGAARRLDR------------------PAFVKPPSD-KS--------------------------------------------------FRAAVHADG----GGLPAAADLP----------------------------------------------------DGTPVQVADVVDW-----------------------------------AVEFRLFVLD----------------GEVRTGSQYAVHGRL---------------ESAPLDGHRHRAAVLS-FARRLLGACG-------------------DTLPSAVVVD-VGLLRTERA--------------PDAEWAVVEANMA-------------------WFSNCY---AADPDRALD-VVLRA
296280712  -HRLRDVRLT----------------------------------TLAEAVTLRER------------------TFIKPTDE-KL--------------------------------------------------FPARVYEGG----AALDPDPKLP----------------------------------------------------PDLPVLVSEPVVF-----------------------------------EVEFRFFILE----------------RRVATFSPYIRGGEIARSA-----------DGAWEADPQESAAATA-TVQALLADPD-------------------VALPPAVVVD-IGHMAG-------------------RGWGVVEANPA-------------------WASGLC---GSDPALVLP-VLRRC
203454815  -YTGRTVSTM----------------------------------TVRESIEWFEKLFG--------GTSESIDFFVKLPEA-KLDN------------------------------------------------FPARKHTIN----RHWATTIGQYHLP-------------------------------------------------DDALIQLQGLMDF-----------------------------------TTEWRFWIAE----------------GRIKAHSLYRAHKLGMEMVWGS--EGFPGGLDGDLAFNDELNMCAD-LVEQMLDDPD-------------------VTYPQGFVLD-VGTIGD------------------GAGAFVVEANAA-------------------WSSGPY---DAEVRGVYE-TIVAS
601044157  -HLRRRVWAG----------------------------------PLQDVPASPLW-------------ATGGPLFAKPAEI-KVRG------------------------------------------------LDAAAYPSA----EAVTRALRDRGLP-------------------------------------------------SSSTVVLSEVVRF-----------------------------------VEEFRCFVAPGP-----------GGRPRVVAASAYLVDGLTWD-------------AWEDGTAAPDPSEAVA-FAGGVVATV---------------------DGPAGYVLD-VGRLAD-------------------GAWAVVEANAS-------------------WSSSPY---HCDPAGVVA-SVLAA
501714260  -FLGRDVWAC----------------------------------ELGDLPYRGTD-----------------PKFYKLAEH-KHAG------------------------------------------------IPAGLRIGR----GIFQRTAGAAFDFATGY---------------------------------------------ETLHVIGSDPMEY-----------------------------------VREYRCFIAR----------------GKVTAASFYLATVPGIHDSTV-----TITWDAYEPARSPDASAAAT-FAQRVVHAMG-------------------ADQPPGYSLD-VGEDKD-------------------GNWSVIEANAA-------------------WSSNIY---HAPVAGVIE-SVLAA

R2K.3
547963574  -RTPNYLAFP---------------------------MNDGAVNVDADLINVTF-----------------DRFMIKDFVKSAKDT------------------------------------------------AFPSSVKTPIS-QDDLNDLVTKFIEIRGSLFV---------------------------------------------GGIVVKEYVDLK-----------------------------RYGGVTNEWRNFCFAN---------------GVNLGLNRNSNQ---------------------PSNCSVPPVELLE-ACNAFD--------------------------SPFCTID-YAELED-------------------GSWTIIETGDG-------------------QVSGLA--ASDSPTRFYE-ALRD-
653157870  EDSARILCFK------------------------------LHEPIQISAVLKEF-----------------PRFMVKDFVKSVKGT------------------------------------------------DFPKFFDSSFS-QEDFEHWMEVFYKYRGELLT---------------------------------------------GGICIKEYLELK-----------------------------KYDGKTNEYRAFYAN----------------HEMISLEPNSGQ---------------------ETFAPTPPEQMLK-KYQTLE--------------------------SPFYTAD-YAELAE-------------------GSWRVIETGDG-------------------SVSGLS--DRQDAKAFFR-ALYL-
488671896  DYTPGIRVYP------------------------------EGEAIDWREVKHAF-----------------PRFMMKDYVKSVKGT------------------------------------------------DFPACFDSSYD-DRQLDEYARRFQAMRGDLYV---------------------------------------------RGIVLKEYVELK-----------------------------RRDSMTNEYRAFYLN----------------GELLTLSPNSNQ---------------------KEGWPAVPIELVN-AIPVLD--------------------------SHFYTVD-FAELEN-------------------GSWTVIETGDG-------------------QVSGLS--PNQYVFKFYD-EIRW-
586958103  NLTFSSWFLK---------------------------------ANQPPDFSAGQ------------------RYFVKGLVKS----------------------------------------------------FGEDSVVASV---AQWQQLYRRHELLPE-------------------------------------------------ELLFVRQHAALL---------------------------------PDSERRFFVVA----------------GEAYGANGTML----------------------PPSLQPAVEALQP-RL--------------------------------FYSLD-VVLTQA-------------------GTPVIVEVGDG-------------------QVSDLK---EWMLPDFGS-TVLR-
504992857  NFTAETYFWA------------------------------VDEELEAKVEELGW-----------------DRYFVKDFVKSNTAK------------------------------------------------LGSIANSPSE--VRSIIEQIATYRGEIE-------------------------------------------------GGIAIRRFENYA---------------------------------HSTERRYFIVN----------------GTPYSSDGE------------------------------VPFMVQE-IASIIN--------------------------APFYSVD-VVENLE-------------------GKLRLVELGDG-------------------QVSDKK---AWSIFKFVE-VIAA-
504973111  DLTPETHCFD---------------------------------VDDRLESKLTE--------------LGWSQFFVKDYVKSLKTS------------------------------------------------TGSIINSPSA--INSLIADMQKFRGTIE-------------------------------------------------GGICVRKVEDFI---------------------------------TETERRYFVIN----------------GRVFAASPD----------------------------LEIPKIVTE-CARRID--------------------------SKFFSID-CIARRD-------------------GRIRIVEIGDG-------------------QVSGLV---GWTVDRFAN-LWTE-
663398345  GATPQSVWLPLAGAGSGSGAAGGGGEASDGADGSGGGDGAGIAPPGPERLAAAVARL-----------GGRGPAIVKDYVKSRKDE------------------------------------------------WGEACFVPELADLPAVARVVARFVELQGPYLA---------------------------------------------GGVVLRRFEEFTRVVWDADDAPSGGSGRLRAQDPGLPDDGRGGRRRAVESRVWWLD----------------GEPVLVGPHPDLPGC--------------------PSAPDLSGVRP-PVRTLG--------------------------CRFVTTD-LAERADG------------------SGWRVVEVGDG-------------------QVSELP--RGIDPSRLLI-ALLA-
503706355  EFTPRSVWTE---------------------------------GPDLGAFEACCSRL------------GPGPAVLRDYVKSLKHY------------------------------------------------WAEASYIPDVANIPAARAIASRFLELRAEDFA---------------------------------------------GGLVVRAFEQFT----------------------------------TTEARAWWVQ----------------GQCVLVTAHPDTPGE-------------------PPTGADLQSIAP-TVAALG--------------------------LPFVTVD-LVLRAD-------------------GVWRIVELGDG-------------------QVSDRP--KSTPAETFIA-AIAG-
498174610  ANTLKSAWTT--------------------------------DLTDNALIKLLDQ--------------FNGPVTVKDFVKSRKHE------------------------------------------------WYSAFYIPNPQNHYQALAIIHNFIQRQAENLV---------------------------------------------GGIVIREFVPFV----------------------QTGVHLKNETPIYEEYRVFYWH----------------SEPFAMIDYWHHNF-------------------DSLGDGDLIFVRK-SAQRIR--------------------------SPFFTID-FARKTD-------------------GTLIIVEIGDG-------------------QVSGLQ---DYDTRKFYH-QWFQ-
501142355  DQTPASLVFP------------------------------QSQGLDAASIAAQAAVY-----------FNQQALIIKDYVKSRKHE------------------------------------------------WYEACFIPNASDQAQSARVIQTFIERQAEALV---------------------------------------------GGVVLRAWHPLQ----------------------IWQTHQQSGLALAYEYRSFWLD----------------GTLLWATPYWDHQ--------------------PSQPTPDWSRYTA-LAQQIQ--------------------------SRFWTLD-LALTQT-------------------GDWLIIELGDA-------------------QVSGLP--DHADAQELLQ-ALAQ-
501191470  EHTARSIWIP------------------------------GSTFDLEAVVVHVREA------------FGAGAVILKDYVKSRKHE------------------------------------------------WFDACFIPAADDVANVRRVVGNFLRLQDDHVV---------------------------------------------GGLVFREFVELR----------------------RIGLHPKSRLPLVNEHRFFVLD----------------GRPFYAAPYWADGD-------------------YPGEPPSADVLAP-VLGRVR--------------------------SRFYAAD-VAEKEG-------------------GGWMLVELNDG-------------------GSAGIP--EGGAADAFYR-SLRA-
495786987  AHTPKTIFES------------------------------ASSESFINTLIAEAKA------------FGSQPIIIKDYVKSEKHH------------------------------------------------WQTACFVSQASNTAKLKETIDNLIRLRGDYLN---------------------------------------------EGIVIREFLELS----------------------DLTIHSKSGMPLKEEYRLFFCH----------------KKLIGIYNYWEEGE-------------------YLASKPDTEGFEV-LAQGIT--------------------------SNFFSMD-IARQKN-------------------GNLIIIELGDG-------------------QVSGIP--DHTDQNEFYK-NL---
502837742  GEKPKSNWTK---------------------------------DLSNANIIELTNE------------FGDQPIIVKEFVKSEKHN------------------------------------------------WNDACFILNAADKIKVKKIVHRFLELRSDYLN---------------------------------------------EEIVFREFEDLE----------------------FLTDHSKSTMPLTKEFRIVYLN----------------KHVVQIFNYWEEGS-------------------YDAEKPDIDFFKK-TAKRID--------------------------SDFFTMD-VAKKKN-------------------GGWIIMELGDG-------------------QVAGLP--EDGNSNEYYQ-QIKN-
554670782  NHTPRTVWLP------------------------------LTGELDVDALMARLAE------------FGHSPLVLKDYVKSAKHA------------------------------------------------WHEACFIPAASDRSAVERVVRRFLALQGKDLS---------------------------------------------VGLVFREYVELA----------------------------GTDPARPWEWRIFFLD----------------NAPLMVTDYWNTGI-------------------AVPEQAALAPFIE-LARAIP--------------------------SRFFTLD-IAQKRS-------------------GEWVIIEMGDG-------------------QVSGLP--QGVSEVEFYQ-KLSE-
498033291  EFTPRSVCLE--------------------------------RDQLTTAYIQEAAKS-----------FGTSALMVKDFVKSRKHD------------------------------------------------WLEACYIPDASDQEHVERVVRRFVELQGAEMN---------------------------------------------GGIVLREFVNLE----------------------FLANHPKSGMPLSKEYRIFFMF----------------GEPIFMVNYWDEVA-------------------YEEVRSDLEDFIK-IAGQVR--------------------------SPFFTMD-IARTQA-------------------GQWIIIELGDG-------------------QVSGLP--EHTDLEAFYT-SL---
501006087  GVTPKTKWSN---------------------------------SVPNKEEIIDMLKC-----------FGSKPVIVKDYVKSRKHE------------------------------------------------WYKSCYIENAEEKEKSVQVINNFIKGQVEDLN---------------------------------------------QGIVLREFVNLE----------------------SIGFHEKSRMPISNELRLFIYN----------------YKVICTIGYWDGKGL------------------NEYPKFVDEVLEK-L-KKVQ--------------------------SNFFTVD-IAKKAN-------------------GEWIIMELGDG-------------------QVSGLQ---DYEVKRFYK-DLIN-
488722731  EQTSRTTWIS----------------------------------GDDIESAWQLYSN-----------FADSDVIIKDWVKSAKNR------------------------------------------------WNDGCFIPAGTDEKRFKRIYKVFREERGNLFN---------------------------------------------RGIVLREFMPLI-----------------------SHGNDMRGMPLVEETRLFFWN----------------GKPVVLPLPNS-----------------------ALPPDGIKRWED-IAGQFK--------------------------SPFITID-VAALTD-------------------GTYKIVETGDG-------------------GVSGLP--VDLDPMLFYR-SLSE-
635314068  PETPKSYWTT----------------------------------DNSIEEAVKILK------------EVKKDAIIKDYVKSRKYE------------------------------------------------WKDSFFIPFHQNMEYKRKIIENFIHRQGESLI---------------------------------------------GGIVFREFISLK----------------------SLGLHQKSAIPISEEYRIVILL----------------GKIISISSYWIRKEK------------------INKEKIEN-FVTK-YIHKIK--------------------------SNFFTID-LGVLEN-------------------GELIIIELGDG-------------------QVSGLQ---EEKVENYYR-NIDY-
494039289  GLTPESVIVS--------------------------------SEGECAKAVAEVGL----------------PIFVKGAVQSRKAR------------------------------------------------GWKACVAETL---DELRRLTRTLLELENRSR----------------------------------------------GRVVLRKVVALR-----------------------HSRCSAEGFPFGREYRLFIYA----------------GEVLGMGYYWEGDDPLR-------------ALSADEEAHVRALAQQ-AAARMK--------------------------VPYAAID-IGQLED-------------------GHWIVIEAGDA-------------------QFSGVS---QIPLLQLWN-RISG-
499872176  RYTARSVWTE---------------------------------GMDAAEAWHAAQA------------LGPQPWLVKDHVKSAKER------------------------------------------------WDEACFIPEGTTQARFEQICANLVDERGDRFE---------------------------------------------RGLVVRKFLPFK------------------------TYGRTPAGPAHLEFRLFFGG----------------GRLLAAEPYHE----------------------FDVDVPDFTGFEP-LARRID--------------------------SPFITLD-VAMLQD-------------------GGWAVVEVNDG-------------------GVSGLP--PGLDPRTLFE-ALFA-
498168243  EFTPRSVCFE------------------------------SFPPLEEISDIFSF------------------PIFIKGA-RQTSQH------------------------------------------------NPELSIIRTP---ADYANISQHYQNDPILHW----------------------------------------------QKIVLREFIDLA----------------------PAPENVPGKIQPSLEFRSFWWN----------------GHCVGWGQYWYQLP----------------PYSTQGIDKGLSIAQE-AASRLK--------------------------VPFLVID-IAKTID-------------------GQWIIIECNDA-------------------QESGYS---SISPQVLWK-NILD-
551026029  ERTPYTQIYD------------------------------ELPPLENVTDDFSF------------------PFFVKGN-RQTNHH------------------------------------------------KKSQCIIENA---DQYYTLRNEWGNDKILNW----------------------------------------------QKVAIREYVPLK---------------------VLDSTSFPDSVPISYEFRFFYFE----------------GRCMAYGPYWNIGPQYS--------------LSEGELPEVLELTDW-AAHRLG--------------------------VSFPAID-VAKTVE-------------------GDWIIIEVNDA-------------------QESGFV---GVNPLKLWN-CTID-
664375985  GLTPVSGWLP----------------------------TAPGAVPDPEGLGALAAGL------------PPGAAVVKDYVKSRKHE------------------------------------------------WDEACYVPDLADPAALHRVVARFVALQGEFLT---------------------------------------------GGVVLRAFEHFV-----------------------------TPEGAAAEIRVWWRD----------------GVPRLVTAHPDSPVAEV----------------PQAFEPVLEPVRA-AVEALG--------------------------CPFVTTD-LALRAD-------------------GVWRVVEVGDG-------------------QVSDLH--REVDRAAFCT-LLTAD
496027362  GLTPFSVWNK------------------------------SRDIGDALELTEGLEG----------------AFVVKDYVKSRKHE------------------------------------------------WYDACFIKDIKDEKESGHILRNFINRQGENFE---------------------------------------------GGVVLRKYESLK----------------------SIGIHKESGMPISEEYRIFVFK----------------GEILVADNYWNENKE------------------VNISEDEYMWIES-IASRIE--------------------------SNFVTID-LARKAD-------------------GSLIIMEMGDG-------------------QVSGLQ---QIDAYEFYR-AFQNQ
493346610  HNPRLSKYLL-------------------------------DKTNVGALFGSCGGDY-----------EPATVYVIRDDDGSLFNF------------------------------------------------EGEYFLIGEQF-FSTYKQRVNDFIQEKGNTFT---------------------------------------------GEAFFELYAPRV-----------------------------CYEGRPVVYRVFYYY----------------GEPFFQSSLAPIRERENDNNE-------DRTEPARKVPTPPQQMVD-AFRNIGH-------------------------NTFYACD-FTLIAG-------------------GGWVCTRIFDG-------------------QMSQVF--RRHHLKEFYA-AFSQ-
583251313  GYSFVGDYEI-------------------------------ENINVGACFGSSGHGS-----------YPPTVYVVRDADGPLSDL-------------------------------------------------NESLAIGYFS-GDEGKELAKKFEEAKGTYFT---------------------------------------------GGAFFEEYVTKA-----------------------------TCNEIPVIWRVFYFE----------------GKAFFKSPLEPDALTI-----------------CPNLPKPPKKLIG-AFQDVTW-------------------------NLFYACD-FILLKE-------------------GIWKCNRFFDG-------------------QMSQVP--IGEETEKFYT-TLAR-
545611084  DYRPESLRAY-------------------------------KPPWDKTHYRSSSYNV-----------STLGPFIVRDAASVWTEN------------------------------------------------GKLKIFTPPIT-QEELDHIIDGFKSLGVNWAT----------------------------------------------QQWFDDIWLEP------------------------AERYWQNENAHACWRAIFFD----------------NRLVCLGVLDSED--------------------EAGIPRPPESLIA-KACEDS--------------------------KPFKAVD-FALTVS-------------------GIWRVTRVFDA-------------------QFTDLP--MSVNPCEYYE-KLG--

II. Individual family alignments

Sequences are labeled by gene name, organism abbreviation, and unique ncbi gene identifier number (gi number), demarcated by underscores. For sequences from GenBank records
	lacking gene names, gene name is not included.

A. R2K.1 family (cdc123-containing)

1. bacterial representatives only

SECONDARY_STRUCTURE         ------------------------------------------------------------------------------------------------------------------------------------------------------------------------------HHHH----------HHHH----------EEEE---HHHHHHHH----------------------------------------------------------HHHHHH------------EEEE-------HHHHHH---------------------------------------HHHHHHHHH-----------HHHHHH--------------------EEEEEEEE------EEEEEEE--------EEEEHHHHHHHH--HHHHH----------HHHHHHHHH-HHH----------------------------EEEEEEE-----------------------EEEEEEE---------------
RLEG3_07720_Rleg_573470149  --------------------------------------------------------------------------------------------------------------------------------------------------MMDFARIEP------------------WADDVIASSFENWPKELRV----HALAEPLDAIPISAGDMRAVL------------SQNAQYRRFL--------------------------NISQPVSLPSRGFGKKM--------EGDAFPKIGPVSWKEISAFISV---------------------------------PLAAIDELMPVMLRG------VTDRMAFILH------AFVCRQVS----TKLHVFPFVDLSKAFEVRFHIEDGE-----PVHAKWMNRSDRYVPPPGS----------GEKLSNFAA-NIAERVGIG---------------------YALLDLLLIK----------------GADGEAIKVVEVNPILERSASGRLFLS----------------------------------------------------------------------------------------------------
_Ecal_515585312             ---------------------------------------------------------------------------------------------------------------------------------------------MFDINRKGFASVET------------------HAQPIVSTEFECLPSVLLN----QFYAKPIEVLPVSHDQLLAVL------------SNNAHYRERF---------------------------GLTQLPVHFTALPRAF--------SQSLFPKLGVVSWKDVVGMQTIPDALLN---------------------------TADYSPVLECWLNAI-------SDRMALTLH------AYRCSSNT----PKLYLFPNQDFRERSEYRLSVSHGE-----IQGVNCYCSRRDY----------HEEY--LEEIKTWWL-SLEPFETSP------------------NLTHIFVDIAWCK------------------DRRAYVIIDVNPNLYLLDQE------------------------------------------VERRCV---------------------------------------------------------
_Ecal_515579254             ---------------------------------------------------------------------------------------------------------------------------------------------MFDINRKGFASVET------------------HAQPIVSTEFECLPSVLLN----QFYAKPIEVLPVSHDQLLAVL------------SNNAHYRERF---------------------------GLTQLPVHFPALPRAF--------SQSLFPKLGVVSWKDVVGMQTIPDALLN---------------------------TADYSPVLECWLNAI-------SDRMALTLH------AYRCSSNT----PKLYLFPNQDFRERSEYRLSVSHGE-----IQGVNCYCSRRDY----------HEEY--LEEIKTWWS-SLEPFETSP------------------NLTHIFVDIAWCK------------------ARGAYVIIDVNPNLYLLDQE------------------------------------------VERRCV---------------------------------------------------------
_Ecal_515582425             ---------------------------------------------------------------------------------------------------------------------------------------------MFDINRKGFASVET------------------HAQPIVSTEFECLPSVLLN----QFYAKPIEVLPVSHDQLLAVL------------SNNAHYRERF---------------------------GLTQLPVHFPALPRAF--------SQSLFPKLGVVSWKDVVGMQTIPDALLN---------------------------TADYSPVLECWLNAI-------SDRMALTLH------AYRCSSNT----PKLYLFPNQDFRERSEYRLSVSHGE-----IQGVNCYCSRRDY----------HEEY--LEEIKTWWL-SLEPFETSP------------------NLTHIFVDIAWCK------------------ARRAYVIIDVNPNLYLLDQE------------------------------------------VERRCV---------------------------------------------------------
_Ecal_515585173             ------------------------------------------------------------------------------------------------------------------------------------------------MDTSALIRIEQ------------------HAAAVVATEYRHLPSSIVS----YYLTPPAVAIALNKQQLTAVL------------SRNLRYRRQY---------------------------GLSPRNVPLSTQPSIQ--------QQDYLPKLGVVSWKDCIGMDMLPKALLL---------------------------PSAQNTTLTCWLNNV-------SDRMAMVLH------AYRVTEET----PTFYLFPYLDFSKRSEYRLAVSYGE-----LTHVRCYRRRNDFQAQH------------IEVIAAWWR-NIKDWPPTD------------------VLAHLFVDVVAGS------------------DPGQFFIIDVNPNLSAYH-----------------------------------------------------------------------------------------------------------
_Ecal_515579285             ------------------------------------------------------------------------------------------------------------------------------------------------MDTSALIRIEQ------------------HAAAVVATEYRHLPSSIVS----YYLTPPAVAIALNKQQLTAVL------------SRNLRYRRQY---------------------------GLSPRNVSLSTQPSIQ--------QQDYLPKLGVVSWKDCIGMDMLPKALLL---------------------------PNAQNTTLTCWLNNV-------SDRMAMVLH------AYRVTEET----PTFYLFPYLDFSKRSEYRLAVSYGE-----LTHVRCYRRRNDFQTKH------------TDAIAAWWR-NIKDWPPTD------------------VLAHLFVDVVAGS------------------DPGQFCIIDVNPNLSAYH-----------------------------------------------------------------------------------------------------------
_Ecal_515580012             ------------------------------------------------------------------------------------------------------------------------------------------------MDTSALIRIEQ------------------HAAAVVATEYRHLPSSIVS----YYLTPPAVAIALNKQQLTAVL------------SRNLRYRRQY---------------------------GLSPRNVSLSTQPSIQ--------QQDYLPKLGVVSWKDCIGMDMLPKALLL---------------------------PNAQNTTLTCWLNNV-------SDRMAMVLH------AYRVTEET----PTFYLFPYLDFSKRSEYRLAVSYGE-----LTHVRCYRRRNDFQTKH------------TDAIAAWWR-NIKDWPPTD------------------VLAHLFVDVVAGS------------------DPGQFFIIDVNPNLSAYH-----------------------------------------------------------------------------------------------------------
_Pbac_655450018             ----------------------------------------------------------------------------------------------------------------------------------------MASLVASAGGSSTNGLLVK-----------------PTRKHVDNCQFTSWFSLHGI----KKVTFRSITIPIPEEFVEYLNEDGVILPKVPDGVSVNPFDPRYVRETKDDDWADEDYEDYDDSDESDEEGGAAATKKSFPGFEAAILKAIEKL-GGKVFVKTNWSSPRDAVWVSGT---------------------------------LQCQSVGEIILLLKSSDFVSFDLEHAYDECREESTEGESVRNASKRPEHFCLVLRKWCNLFPSQEFRCFVKDRQ-----LIATSQRDPTKFYNF----LPSNVSTY--SPLVSEFFDTSIRPVFPDD---------------------NFSFDVYIDR-------------------KHRVWLQDFNVFSRATNAL-LFTWAELLAVDTQE------------------------ADAVANEFRVVQDPSGVQSSQLAAHKVPADVVNDPNNFIKEFVESCRNGTLNVQSNGNVDDEGGQ-
_Rsp._517993611             ------------------------------------------------------------------------------------------------------MDIIISPYVKISEHDSYTVYQYPKNYLELFLDELFYDNSIEVTDNYTFKMSINGFILNEVNIESLIDFEYLRQKQIATYWVSKWYDYL------IINVPDT--MITFKSKLIYLT-------------------------------------------------DNDIADLYEYKINNIV--------PNNLIKKISKAMSKISCSCFIRTDAYSPKDLLHREIVDTL---------------EVTNAITALKLI-TQ-------SERCSSKLF------GGDNKIIS----KNIVIREYVNYDTNYEFRCFVYNWR-----LTAISQ----SGFEYNP-ILHSKKNII--YQSIIKFWD-KFSRICPYS---------------------ECTMDIVYND-----------KWIDNTLNHSGVVIIEFNLLANI-------------------------------------------------------------------------------------------------------------
_Dpro_654515672             ---------------------------------------------------------------------------------------------------------------------------------------------------------------------------MLKKRTLKQYGVSKWYSTL------ADYTFPTTFVKLKETELDLLA-------------------------------------------------QGIVDECEVYHIVNRIAHAQ----QAFFGSKFVFADTIAPTDTTRFISKRG----------------------------SIHSAASAWKNL-AS-------SEKVRTAA-------KNREFE-------CICVRPFRSMNRSREFRLFINEGN-----LILMSQYWLDRHYHK----LESKKDFY--WTKAEELLD-EISWLLPKE---------------------TIVLDLYFTS-------------------KDQIILIDFNPWGPPTLPLLAESWNI--------------------------------DWTEEIGIKIVQPEDQTII-----------------------------------------------
_Lara_494490064             -----------------------------------------------------------------------------------------------------------------------------------------------------------------------------------MNKISEWYPFL------GHLTFPTVFLALSSEECDIVA-------------------------------------------------------------------------QGKSLASLEERITRAIASLPGSCVVGLDSCMPTDSVSFQKSK-------------SLKNGKLALKIL-NE-------SEKVKSAVS------SGD---------KTITVRPYRRMDKTREFRLFVKDGE-----LKAMTQRNLERHF----KRLEARRELY--WQKGVSFVD-EIKKYINEK---------------------DYVVDIYFT-------------------STADIMIVDFNSWGEPTLPLLMKSWER--------------------------------DWSSVDGLKLMAEPIQLGGDIKVSF-----------------------------------------
_Ssp._640625317             ----------------------------------------------------------------------------------------------------------------------------------------------------------------------------------MFLEYREWPPAL------RALGVSEL---LGRVDLDYE---------------------------------------------------LTDEGLAIAFDPAAIPVLDPPIFAKLGPKALPRGLLRD-----------------------------------------MGHLPGLIETL-TP-------NARAVVGLV------AEQGAL------LALYLFRWTDFSQVSEFKLSITPDR------VQLDAYVWRGARSEPL------TDGL--RQSLEAFGA-GMREHVSAG---------------------RFRADVALMP-------------------NDDLRLIELNPAKSALLRG---------------------------------------PKDLAITCTLP-------------------------------------------------------
llo2267_Llon_289165596      MNFFEKLDLLLKDVGIEDHNLFIHWVMKQLQLELDENAITAEQAPHKVLLNVSSLSATDLKKLFLFFNLPESTTPIENRIELSDSFLSEVVLPQFEEYVRTFAPNKTEIQSSRQKSALFDEGDEDLTDIDDFILVDSNDDQGEEIDYESYPWLNPAHPLYFPSTQDANPTSLTRWEAVDKFSVENWYPHL------KKYTFESCFFTLDHADIEFLL-------------------------------------------------GIGSSEYQSTNLEQKFDKLLSQFNNQEVFMRLSTRSPKDSRHLFDEAATLMSNDYFYWKDTDNKNQQLVSFVASMAKSMKITNGKKIIQMI-QE-------SPRVQNDLF------ALLSSESPSDCKTNIVLREWHNIRPDHEFRLFVSRRCREESIVTAISQYFHFLHFDKAPGDCFNFLDESIKKSLILKFQN-YVLKSIDPAVARFLNFSSEQDDDNSINCIREYIVDLALVPISEYHGEVTDENKIEIGENTYILTVIELNPFAPSATGCGLFNWQKDLDILWGKAPCEYPIYRFRTEPRENFSSVTLLPSNYEQVIDRA-LVKRLVTSLPSVDSQASPSMSTASHSSRFFTSPPQQINETSSLGPVSDDMKCSL
_Lsai_653018179             ------------------------------------------------------------------------------------------MFPQFEEYVRNLAANKTEVESSEQITKSLDEVEEEFTDIDDFILVDSNGDQEEEIDYESYPWLNPSHPLYFPATQDTNPTSLTRWEAVDKFSVENWYPHL------KKYTFESCFFTLDHADIQFLL-------------------------------------------------GNGSSEYQSTKLEQKFDKLLSQFNNQEVFMRLSTRSPKDSRYLFDEAATMMSHDFSYWKDTDNKNQQLVSFVASMAKSMKINNGKKIIQMI-QE-------SPRVQNDLF------ALLNSESPSDCKTNIVLREWHNIRPDHEFRLFVSRRCREESIVTAVSQYFHFLYFDKAPGDCFNFLDESIKKSLILKFQN-YVLKSIDPAVAKFLNFSKEQDDDNSINCIREYIVDLALVPISEYHGEVTDENKIELGENTYILMVIELNPFAPSATGCGLFNWEKNLDILWGKAPCEYPIYSFRTKPRENLSSVTLLPSNYEQVIERA-LDKRLAASLPSLDPQVSPSMSTASHSNRFFTAPHQQIDDTSSLGLVHDGMRCFP
_Lpne_652968979             -------------------------------------------------------------------------------------------------MKTVDEIDSLLQSDKIDEKSISAPVISPNEAIEDDFYLIDDSDYVEDHDFSDFPWLDPEHPLYFPATQAHNPKGSTRWDAVNKFSVENWYPHL------KKHTFKSRFITLNYNDIQYLM-------------------------------------------------GNILPDYDSTKLESMFNNIISEFNNKEVFMRLSTRSPKDSKHLFEEAATIMSKDFVYWSENDNKHQQLVSFVASMLKSMKIKNGKKIIETI-AQ-------SPRVYNDLI------ALVSSVDQSDCTTNIILREWHDIRPDHEFRVFVSRRHRKESIVTAISQYFHFLYFDKNPADCFNFLDEEDKKAVIKKFEN-YVLKSVDPDVAKFLNFSSEQDNDESADCIREYIVDLALIPIHQYHGEVTNENKIEIGKSIYVMVVIELNPFAPAATGSGLFNWKNDLMMLWGKTSCDYPVFKYRTTPREDLQSVSLLPSNYESVIQSA-LIKRLGNSYSSSVSQTARFFSSAQPEKGSSGSTLFDMDKKPW------------
_Lpne_653015156             -------------------------------------------------------------------------------------------------MKTVDEIDSLLQSDKIDEKSISAPVISPNEAIEDDFYLIDDIDYVEDHDFSDFPWLDPEHPLYFPATQAHNPKGSTRWDAVNKFSVENWYPHL------KKHTFKSRFITLNYNDIQYLM-------------------------------------------------GNILPDYDSTKLESMFNNIISEFNNKEVFMRLSTRSPKDSKHLFEEAATIMSKDFVYWSENDNKHQQLVSFVASMLKSMKIKNGRKIIETI-AQ-------SPRVYNDLI------ALVSSVDQSDCTTNIILREWYDIRPDHEFRVFVSRRHRKESIVTAISQYFHFLYFDKNPTDCFNFLDEEDKKTVIKKFEN-YVLKLVDPDVAKFLNFASEQDNDESADCIREYIVDLALIPIHQYHGEVTDENKIEIGKNIYVMVVIELNPFAPAATGCGLFNWKNDLMMLWGKTSCDYPIFKYRTTPREDLQSVSLLPSNYESVIQSA-LIKRLENSYSSSVNQAARFFSSAQPEKESSGSTLFDMGTKPW------------
_Lpne_52841912              -------------------------------------------------------------------------------------------------MKSVDEIDSLLQSAKTSEKDSSEQIVPPNDEIEDGFYLIDDSNHDEDLDFSDFPWLDPDHPLFFPATQANNSKGSTRWDAVNKFSVENWYPHL------RKHTFKSHFITLNYNDIHYLM-------------------------------------------------GNIPPDYDSSKLESIFNKILSEFNNKEVFMRLSTRSPKDSKFLFEEASTIMSKDFVYWNENDNKHQQLVSFVASMLKAMKIKSGRKIIETI-AQ-------SPRVYNDLL------ALVSSSNQSDCTTNVILREWHDIRPDHEFRVFVSRRHRKESIVTAISQYFHFLYFDKSPADCFNFLDEEDKKAVIKKFEN-YVLKSVDPDVAKFLNFSSEQDDDESSDCIREYIVDLALIPVSQYHGEITDENIIEIGANTYVMVVIELNPFAPAATGSGLFNWKNDLMMLWGKASCDYPVFKYRTTPREDLQSVSLLPSNYESVIKSA-LIKRLETSYSNSASQKERFFSSTKPVTESSGSALLDMVTKP-------------
_Lpne_397667414             -------------------------------------------------------------------------------------------------MKSVDEIDSLLQSAKKNEKDSSGQIIPPNEAIEDDFYLIDDSDYVEDHDFSDFPWLNPEHPLYFPATQAHNPKGSTRWDAVNKFSVENWYSHL------KKHTFKSHFVTLNYNDIQYLM-------------------------------------------------GNIPPDYDSTKLESIFNKILSEFNNKEVFMRLSTRSPKDSKFLFEEASTIMSKDFVYWNENDNKHQQLVSFVASMLKAMKIKSGRKIIETI-AQ-------SPRVYNDLL------ALVSSSDKLDCTTNVILREWHDIRPDHEFRVFVSRRHRKESIVTAISQYFHFLYFDKSPTDCFNFLDEEDKKAVIKKFQN-YVLKSVDPDVAKFLNFSSEQNDDESSDCIREYIVDLALIPVSQYHGEITDENIIQIGTNTYVMVVIELNPFAPAATGSGLFNWKNDLMMLWGKASCDYPVFKYRTTPREDFQSVSLLPSNYESVIKSA-LIKRLETSYSISASQKERFFSSTKPVTESSESALLDMVTKP-------------
_Zfor_521962559             -------------------------------------------------------------------------------------------------------------------------------------MTFTLPPIQAPTDEEVKQWTTA------------------KWKAQESALLSGLHPAI------MAITIPTMFFAIPMQEVKEKW------------------LPVF---------------------------DGKNDGEACTRQVECGRRALAEF-PDGVFFKLDSRSPKDSDIGK-----------------------------------YTAENLDQLPNAFFG-------SERVFDDIC------LQRHHRDR----IVLCFRKWVEFGE--EYRVFVKERQ-----IQGISRYDYLSASKVEH--TPEVVAAV--QGQAEGYLA-TINEHYPPS---------------------DYVFDIGHTP--------------------DGPVMIEINPYG-LSDPC-LF-------------------------------------GSYANIRGFVA------------------------------------------------------
D187_000664_Cfus_528060458  ---------------------------------------------------------------------------------------------------------------------------------------------------------------------------------------------M------QALSFRQQGIELTRAQLHALA------------RRNGIAASLF---------------------------DA-VGTAPLTALQAQLDGLLQQF-PAGAFVRLGSRSPKDTERFVLSGG-------------------------------RADSGAEAIALLSAG-------SRRMFVDYR------RCMQNHWT----PSIFLREWQPMSSAQEWRCFVHERQ-----LLGITQYWHAQALDSGA--CAQLQRTG--IASLLQLAA-QLLQRLPLP---------------------SFVFDACLPL-----------PSP----LAARAVLIEINPFGATTDAG-LFD-AC--------------------------------DERLDRSIRWR-SEAGVQQRPLADCCQPASGAPSQPGQAQNLLGSPP-------------------
_Mmar_519004236             -----------------------------------------------------------------------------------------------------------------------------------------------MANNQRVAWLPP-----------------YDFSAAKTTFIDAWPKAW------RDLAPGYDIVPVDRIEMNALG------------AQIMGFRHWF---------------------------TP-ASTQPLIHLAHRLDAVIAKQ-NRACFIRLSSRSPKDSIYALRNGL-------------------------------CIRDGAQALAIILEG-------SERCAADLR------MALDYHHP----MAIIVRNWIDFPPWAEFRCFMVGRC-----WVGASQARHLERIAYPP--IADYKSKI--LEVLNASMK-KIAAASPID---------------------NAAFDLVFDS----------------LQKSNHAILLDANPLLVSTDTA-LFSS----------------------------------IADLDSTFRFRNSKDNSIRKISF-------------------------------------------
Q664_25730_Cvio_667801279   -------------------------------------------------------------------------------------------------------------------------------------------------------MSKA------------------YFDRVSPTFIENWAPAL------HDLSIPQADVPLSRLETQALE------------ART--WHQKS---------------------------TDRSEHIPIDSLMWRLNVALRQF-PEGAFVRLGSRSSKDSAYALQHGL-------------------------------RVRTGEAAVRMLTEG-------SERTAFDLR------LAAQVHYA----PHIFVRKWMDIPRWTEFRCFMKERR-----LVGISQYDCLTLGIRPE--ITRNAESL--HTAIREFSQ-RFAEASHLD---------------------NVVFDVFVEG--------------MDTSGPLNVRLLELNPFFPKTDPC-LFDWSV--------------------------------PADFDGSFRFLGASTLKGRDHLGHGRGG--------------------------------------
Q664_08520_Cvio_667804119   --------------------------------------------------------------------------------------------------------------------------------------------------MSPVSWSRA------------------YFERIRPTFLECWAEEL------RALAVSHVHLPLTPAEARALS------------VTPPLWRERL---------------------------VA-SDPEGLHSLAARLQKALEGV-EQGVFVRLGSGSPKDSALFREQGG-------------------------------CARTPMMALKFL-QT-------SPRTRAHLS------RFLELGHP----VHLFVRHWVRIPPWQEFRCFMRNRR-----LVGISQLAHRGD--TPEYSLAPRAEEL--GRTLQDFFV-GVARASHVG---------------------SAVFDVWCDT-------------GAGDGAPARVWLLDANPWGPASDAC-LFDWSQ--------------------------------PEGFDGSFRYLK------------------------------------------------------
_Arhi_518854330             ----------------------------------------------------------------------------------------------------------------------------------------------------MLG----------------------LHEKWRATFIESWPDEV------CELGLPFEQLLISEQDRAAIG------------SRTAAFRELF---------------------------DIDELTQLSDEFRIGIDAKTALF-KDGAHFRLGGCSFKQPGRYQDG---------------------------------PIFNSAQLMPHVLRD-------NPRVAGLLA------SSLQDKFD----VCMFIRPWENIPKWSEFRLFMKNRE-----FIGASQYFHTAFF--PE--IEAKARSI--AVALVEFAD-RFRQVAHID---------------------DAIVDIYLRP-----------DDA----GGFEGVLLDLNPLILRSDPC-LFQWKN--------------------------------GGDFDRGLRFRGRDNRVLAITPLPFAYAA-------------------------------------
_Ssp._640615598             ---------------------------------------------------------------------------------------------------------------------------------------------------------------------------MSFPFAISQTFPENWPAPL------KNLAAPMTSIVLTPEDASALG------------AFSTAFRQQF---------------------------DVRARDFLSKALLTEIDSALLKY-PEGVMPRIGYCSWKASLIEKR----------------------------------AAKTRNDVMQIITTD-------DPRVGNALS------VLVGSDDP----VVLHLRAWRKIPDWSEVRLFFKNGR-----FVGASQYAYRRSF--PE--ITSNAKEI--ETMVARAAS-VIVEKLHLS---------------------DVIVDLALLP-----------SDGKSPVSGLKPLLIELNPFSPLTDAC-LFSWEK--------------------------------GGNFDGRFRWTQA-----------------------------------------------------
_Sste_492493562             --------------------------------------------------------------------------------------------------------------------------------------------------------------------------------MLALTFPENWPPTL------TLASLPAVSVTLSDGDAETLG------------SQSALYREVA---------------------------GDARRIGFGPDFEKTIAKALETF-PEGLMPRIGMCSWKASTVVHA----------------------------------PCHSVADVMRVITAN-------DPRVAQAIL------DHRISKRP----VVLHLRAWRDIPDWAEFRLFVKRRG-----LLGVSQYAWQETF--PQ--IAAQHSAI--VTAVNALLK-DIWEDLHMD---------------------DVVIDVCVLP-----------EG-----DGLKAWLIELNPLDPRSDAC-LYSWEN--------------------------------GGDFDGSFRYN-RPYRAEAFG---------------------------------------------
cdc123_Psp._374333538       ---------------------------------------------------------------------------------------------------------------------------------------------------------MA-----------------Y-PKAVSDTFFASWPDAL------KQASVPHEEIPISPEDVLALA------------ANTPEFCEQF---------------------------GIFNHYGLSEEFRDTISKGLEKF-KDGAFPRLDYCSWKTSCLLNA----------------------------------PAKSLGEVEAIVLQP-------NQRVASALM------DPVINNTG----ANFYLRKWVDIPRWSEFRIFMRDRK-----IIGVSQYYTDEQF--PA--LQENLDKI--REALIEFCL-FFYKESHLD---------------------TVVADVFLAN-----------QN-----EKLQAQLIELNPFLNRTDPC-LYNW-E--------------------------------KSNFDGKLRFI-AGDRVLAAPMAN------------------------------------------
_Dchr_654084322             -----------------------------------------------------------------------------------------------------------------------------------------------------------------------------MYTEHKQTFIENWPETL------LTLSFQSEGIELHERDVIAIG------------ACTSEFMDAK---------------------------RLLEKPPFSEQLRDDIEYILSKF-TTPVFVRFGGVSYHEATIP------------------------------------RTNNVDDVIRQLSVS-------SHRVASYLW------DCLQSSTP----AWLFLRGWHDIPRWGEFRCFIKDGN-----VVGVSQYHCLEYF--PF--LTEHADEI--RQQIIQFLQ-KLIPLLHMD---------------------SVVADIAITH-----------QN-----GQYDTMLIELNPFIQRTDAC-LFSWLN--------------------------------GGDFNGRIRVN-LSQADASAEKRRRPYLL-------------------------------------
_Pcar_497965255             -----------------------------------------------------------------------------------------------------------------------------------------------------------------------------MYSEHKATFIENWPKEL------LDLSFLSEGFELHERDVIAIG------------ANTHDFMNAR---------------------------GLLEKPLYSAQLREDIEYALSVL-NKPAFLRFGGVSYHDYARP------------------------------------RLETVDGVIEQLAVS-------NRRVASYLW------DCLQSSTP----VWLYLREWREILRWGEFRCFIKEGK-----VIGVSQYHCLEYF--PF--IKEKENEI--RLQLIAFLQ-KLLPVLHVD---------------------SVVADVAITY-----------QD-----SKFATTLIELNPFIQRTDAC-LFSWIN--------------------------------GGDFNGRIRIN-LSDADAQAEKQRRPYLL-------------------------------------
_Bsp._496403701             -----------------------------------------------------------------------------------------------------------------------------------------------------------------------------MYSEYKKTFIENWSEDI------LAQSFKSESIELHERDVIAIG------------ASTDEFMSAR---------------------------GLQEKPFFSAQLHDDIEYALSVL-NKPAFVRFGGVSYHDASLS------------------------------------RLDTVDGIVKQLSVS-------NRCVASYLW------DCLQSSTP----VWLFLREWRDIPRWGEFRCFIRDAK-----VIGVSQYHCLEYF--PF--LKEKENEI--RLQLIMFLQ-KLLPVLHMN---------------------SVVADIAIDY-----------QD-----GKFTTTLIELNPFIQRTDAC-LFSWVN--------------------------------GGDFNGRIRVN-HSIADAHAEKRKRPYLL-------------------------------------
_Bsp._496403708             -----------------------------------------------------------------------------------------------------------------------------------------------------------------------------MYSEHKATFIDNWPQDL------LALSFLSEGFELHERDVIAIG------------ASTDEFMTAR---------------------------ELQEKPVFSVQLHDDIEYALSVF-NRPVFVRFGGVSYHDASLS------------------------------------RLDTVDGVVKQLSVS-------SRRVASYLW------DCLQSSTP----VWLFLREWRDIPRWGEFRCFIRDAK-----VIGVSQYHCLEYF--PF--LKEKENEI--RLQLIMFLQ-KLLPVLHLD---------------------SVVADVAIDY-----------QD-----GKFTTTLIELNPFIQRTDAC-LFSWVN--------------------------------GGDFNGRIRVN-QSIADAQAEKRKRPYLL-------------------------------------
consensus/100%              .......................................................................................................................................................................................................s.......l.....................................................................b...............h....s.........................................................h............p....h.....................h.hb....h....Eh+h.h.............p...................................h.............................h.hDh............................h.-hN..................................................................................................................
consensus/95%               .........................................................................................................................................................................................h...h.........s.....h.l...p.................................................................b...b...........h..+.s..s......................................................h..........s.+....h.....................h.lb...ph....EhRh.h...p........hp...........................h..h....h............................ph.hDlhh..........................ll-hNs.................................................................................................................
consensus/90%               ......................................................................................................................................................................................hpph...l.........s.....h.lp..ph.hl.............................................................b...b...........hh.+hs..S.+ps..............................................h...l..........s.+h...l.....................h.lb.a.ph....EaRh.hp..p.....h..hpph.....b...................l..h....h......s.....................phhhDlhh..........................ll-hNP.................................................................................................................
consensus/85%               ......................................................................................................................................................................................hpph...l.........s.....h.lp..ph.hl.............................................................h...h...........hh.+hs..S.+ss..........................................ps..h...l..........s.Rh...l.....................hhlb.a.ph....EaRh.hp..p.....h..hsph.....h...................l..h....h......s.....................phhhDlhh..........................ll-hNP.................................................................................................................
consensus/80%               ......................................................................................................................................................................................hpph...l........hs.....l.ls..ph.hl.............................................................h.p.h.........p.hh.+hus.S.+-s........................................h.ps..hb..l..........s.Rh...L...........p.........lhlb.a.sh....EaRhhlpp.p.....l.uhspY.....a...........p.......l..a....l......s.....................phhhDlhh..........................ll-lNPh....p................................................h..........................................................
consensus/75%               .................................................................................................................................................................................h....hcph...l......b.hs.....l.ls..ph.hl..................................................s..........h.p.h........pp.ha.+lus.S.+-s........................................hpps..hhp.l..........s.Rhh..L.......s...sp......s.lhlb.a.ch....EaRhalpp.p.....l.ulspY..b..a...........p.......l..a....l......s.....................phhhDlhh....................s...h.lI-lNPh....s............................................h...h..........................................................
consensus/70%               ...............................................................................................................................................................................p.h...bhcsW.s.L......b.hoh....lsLspp-l.hl..................................................s....sh..spl.p.h..hh..h.spshF.+lus.S.KDs..h.....................................hpss..lhp.l..........S.RhhssL.......sh.pspps....splhLR.a.ch.p.pEaRhFlpc.c.....l.uloQY.pbp.a......h....p.b..b..l..ahp..lb.....s.....................phhhDlhh....................s...hhlI-lNPhh..sss..hb......................................shp..hp.........................................................

2. all representatives

SECONDARY STRUCTURE                  ---HHHHH------------------------------------------------------------------------------------------------------------------EEEEE------------------------HH-------HHH---------------------------------HHH-----E-----------------------------------------------------------------------------------------------------------------------------------------------------------------H-------------------------------------HHHHHHHHHHHH---------------H--------------EEEE------HHH---------------------------------------------------------------HHHH---------------------------------------------HHHHH------------------HHH----------E---------------------------------E------------------------------------------------EEEEEEEEE-------------------------------E---EEEE-------EE----------------E--------------------EEEEEE----H--------HHHHH--HHH----------------------------------------------------HHHHHHHHHHHHHHH---------------------------------------------------HHH--------------------------------------------EEEEEEE-----------------------------------------------------------------------EEEEEEE-------------------------H---------HH-HHHHH-----------------------------------------------------------------------------------------------------HH-------------------------------------------------------------------------EEEE-----------------------------------------------------------------------------------------------------------------HHHHH-------------------------------------------------------------
CYME_CMB007C_Cmer_544209437          MKLVPDAFH------PGYWGPL----------------------------------------------------------------------------------------------LSAHMPRTAYL---------------------PVSES-------FIS---------------------------------ALQRDSLNEADA---------------------------------------------------------------ERLQ----------------------------------------------------------------------------------------SDFS-------------------------------------ELIQGLSHAMDY---------------LRTDHSTSE-----GFCPKLNGKLPYDA---------------------------------------------------------------AWIL--GRHA----------------------------------PCCYTWNEAM------------------MVLR-A---SSR-I---------------------------------HEPT--------------RTSWQGS------------------------FCLVVQRWE--------------------------QIHPAN---EFRC-------FI----------------SSCR-----------------VIAICQRHPDT--------PYPFL--ARE----------------------------------------------------RDRLVNAMVSFYETH---------------------------------------------------VRPLHPA-------------------------------------YAECILDCYL------------------------------------------DDS-------------------------QSVRTLGIDAW-S----------------DDALL---------LF-EVSEM-----------------------------------------------------------------------------------------------------EALRAQAAAAFPGQHSDPDGHRQSEAPGEDATGAT--------------------------------------VSFRSVAQGFA-----------------------------------AVP--------------------------------------------------------------WGWSEKLPLELGD-----------------D----------------------------------------FLR
EDI_157500_Edis_167385292            MKPSTVQSL-----------------------------------------------------------------------------------------------------------FQSILIEGEVV---------------------ELDEE-------CIK---------------------------------EIEGDVK--------------------------------------------------------------------SNFTWS-----------------------------------------------GIEEIKDEEEERAFEKREKEEKQKK--------------ENWK-------------------------------------SEIEWFNQIIERNVH------------LYG-----------GVFLKINGKALVDA---------------------------------------------------------------EWMN--GS------------------------------------LKVCNGNEGM------------------MLLQ-G---SER-AQELI----------------------EK-----YR----------------QEGKV--------------------------NELEIRKFE--------------------------EIRISD---EFRC-------FV----------------VHRE-----------------LIIISQRYNDA--------YEVKI--QER----------------------------------------------------KKEIIKKVNEL-FEI---------------------------------------------------IKSHHF--------------------------------------SDCYTFDVVI------------------------------------------NNK-------------------------I--KVIGFDEMNER---------------SFEGM---------TF-NKEELMSNA-------------------------------------------------------------------------------------------------KDINVTK------------------------------------------------------------------PLLKYVEDSQHI----------------------------------IPT--------------------------------------------------------------VKQFYGIPEEFYD-----------------E----------------------------------------KVL
ENU1_182130_Enut_672809402           TVQSLFHSI---------------------------------------------------------------------------------------------------------------LIEGEVV---------------------ELDEE-------CIT---------------------------------EIEGDV---------------------------------------------------------------------KSNFTWSG---------------------------------------------IEEIKEEEEKE----------------------------KVFEKK-----------------------------------EKEEKQKKENWKSEIEWFNQIIERNVQLYG-----------GVFLKINGKALVDA---------------------------------------------------------------EWMN--GS------------------------------------LKVCNGNEGM------------------MLLQ-G---SER-AQELI----------------------EK-----HR----------------QEGKT--------------------------NELEIRKFE--------------------------EIRISD---EFRC-------FV----------------VHKE-----------------LIIISQRYNDA--------YEVKI--QER----------------------------------------------------KKEIIKKVNEL-FEI---------------------------------------------------IKNHHF--------------------------------------SDSYTFDVVI------------------------------------------NNK-------------------------I--KVIGFDEMNER---------------SFEGM---------TF-NKEELMLNA-------------------------------------------------------------------------------------------------KDINVVK------------------------------------------------------------------PLLKFVEDSQHI----------------------------------IPT--------------------------------------------------------------VKQFYGIPEEFYDEKVLNTFLTNPSKLTSFYYLG---------------------LNCEFLKLNNSKINFEFLI
EHI_128440_Ehis_183234918            MKPSTVQSL-----------------------------------------------------------------------------------------------------------FQSILIEGEVV---------------------ELDEE-------CIK---------------------------------EIEGDVKSNFT----------------------------------------------------------------WSGIEE-----------------------------------------------IKEEEEEKIFEKKEKEEKQK-------------------KENW-------------------------------------KSEIEWFNQIIERNVQ-----------LYG-----------GVFLKINGKALVDA---------------------------------------------------------------EWMN--GS------------------------------------LKVCNGNEGM------------------MLLQ-G---SER-VQELI----------------------EK-----HR----------------QEGKT--------------------------NELEIRKFE--------------------------EIRISD---EFRC-------FV----------------VHKE-----------------LIIISQRYNDA--------YEVKI--QER----------------------------------------------------KKEIIKKVNEL-FEI---------------------------------------------------IKNHHF--------------------------------------SDSYTFDVVI------------------------------------------NNK-------------------------I--KVIGFDEMNER---------------SFEGM---------TF-NKEELMLNA-------------------------------------------------------------------------------------------------KDINVVK------------------------------------------------------------------PLLKYVEDSKHI----------------------------------IPT--------------------------------------------------------------VKQFYGIPEEFYDEKVLNTFLTNPSK----LTSFY--------------------YLCSNCEFLKLNNSKINFE
DHA2_7593_Gint_559172713             CKSVYDKFR------YVNWPGP----------------------------------------------------------------------------------------------LMKYTIQRKTV---------------------LIPPE-------VCK---------------------------------EIVSDD---------------------------------------------------------------------MVMT-----------------------------------------------------------------------------------------SVNA------------------------------------SALHKFCESLDL---------------VN------------GKFLKVDWAAPTDC---------------------------------------------------------------SQIV--ET------------------------------------LMCIVPSNAF------------------ILLK-M---SEK-IRNAL----------------------LNFHNEEIPGKF-------------DPSIP--------------------------HYLYVMPYE--------------------------RLHKIF---EYRI-------FI----------------KDLR-----------------IVLIAPR------------YQCMI--TDV----------------------------------------------------TTDIVRFVKVFLLEH---------------------------------------------------ITQHMG--------------------------------------VTDLIIDLYC------------------------------------------DVDEDIC---------------------SSILLLDMQPLPTAESAFEDLCV------LGAAV---------LK-ERLKP-----------------------------------------------------------------------------------------------------DAILDPLFVNP--------------------------------------------------------------TVERISAVG-------------------------------------RAT--------------------------------------------------------------TNNTY--PSELTSG----------------MISG---------------------THTEIIELVRQATRKTEIP
Chro.60220_Chom_67607916             DKNKLRNMG------ISKWYERLSNY------------------------------------------------------------------------------------------KSKVTIHGILF---------------------NIPDS-------LILE--------------------------------YFESDDL--------------------------------------------------------------------DINYEA-----------------------------------------------LSES------------------------------------SRK-------------------------------------CLED-INKSIRENTEF-----------FSM-----------GFSPKFTWSAPTDA---------------------------------------------------------------TWIN--SNRS----------------------------------ICCRTLEELF------------------ILLK-A---STK-VSEDI----------------------DR-----AK----------------EGNIS--------------------------NVLLLREYIP-------------------------TLNEMF---EFRV-------FIGGCSLH----------SEYK-----------------ILGISQRHICY--------YYKEL--SENFKL-------------------------------------------------RTNIKSCIMEFFLYS---------------------------------------------------KKELISELFDIFN-------------------------------SMCIAFDVYISNY---------------------------------------KDK-------------------------LSILIIDVQPL-L----------------HASPL---------LF-NLFELK----------------------------------------------------------------------------------------------------LNLLTEEGTELGF------------------------------------------------------------DILRTTENNSTKNM--------------------------------INN--------------------------------------------------------------SYLKGFVPDELLS-----------------VSNS---------------------DFYNNETLDK------LEW
GL50803_7593_Glam_159110284          CKSVYDKFR------YVNWPGP----------------------------------------------------------------------------------------------LMKYTIQRKTV---------------------LIPPE-------VCK---------------------------------EIVSDD---------------------------------------------------------------------MAMT-----------------------------------------------------------------------------------------SVNA------------------------------------PALHKFCESLDL---------------VN------------GKFLKVDWAAPTDC---------------------------------------------------------------SQIV--ET------------------------------------LMCIVPSNAF------------------ILLK-M---SEK-IRNAL----------------------LNFHNEEIPGKF-------------DPSIP--------------------------HYLYVMPYE--------------------------RLHKIF---EYRI-------FI----------------KDLR-----------------IVLIAPR-------------YQCM--ITDV---------------------------------------------------TTDIVRFVKVFLLEH---------------------------------------------------ITQHMG--------------------------------------VTDLIIDLYC------------------------------------------DVDEDIC---------------------SSILLLDMQPLPTAESAFEDLCV------LGAAV---------LK-ERLKP-----------------------------------------------------------------------------------------------------DAILDPL------------------------------------------------------------------FVNPTVERISAVG---------------------------------RAT--------------------------------------------------------------TNNTY--PSELTSG----------------MISG---------------------THTEIIELVRQATRKTEIP
CMU_008050_Cmur_209878444            VNIKVYLKF-----RVSFWYPVEAKSLLLVPNKVDTVRLVNEAAVENKEAVSSREFYFNLIKLLADLGTPRDDIDINNFLIESILNFFMNIWGPNLDVYNGNDFGCMSWFPIVNSQKKMITIPSSLI---------------------PIPKS-------IINE--------------------------------YMNIDG---------------------------------------------------------------------IYNISK-----------------------------------------------LNLS-----------------------------------KESK-------------------------------------IFLERLSLAVNK---------------YSKS----------GCVPKFTWSTPTDS---------------------------------------------------------------CWIL--PSST----------------------------------IRCENYSDVM------------------ILLK-A---STK-VSEDLE---------------------RS-----QN----------------QKDFQ--------------------------HAILLREYIP-------------------------TWDSSM---EFRI-------FLSKASQN----------ASFR-----------------INGISQRHISL--------YFEEL--VSNKEL-------------------------------------------------QNGILSSINSIVKCDEREL-----------------------------------------------INELFEALK-----------------------------------TKKLAIDVYLIKTST-------------------------------------KYK-------------------------FRSLIVDVS-LMY----------------NIDTL---------LF-SFSELKFNQLRQ----------------------------------------------------------------------------------------------IDELNLI------------------------------------------------------------------DLYRVVEDPQSTIY--------------------------------QIK--------------------------------------------------------------NDLKGKVPKEMLN-----------------MCDG---------------------NDIEELLSQF------SLK
GSB_7593_Gint_559180405              CKSIYDKFR------YVNWPGP----------------------------------------------------------------------------------------------LMKYTIQRKTI---------------------LIPPE-------VCK---------------------------------EIVSDD---------------------------------------------------------------------MVMT-----------------------------------------------------------------------------------------SVNA------------------------------------PALHKFCENLDL---------------LN------------GKFLKVDWAAPTDC---------------------------------------------------------------SQIV--ET------------------------------------LMCIVPSNAF------------------ILLK-M---SEK-IRNAI----------------------LNFHNEDAPKVF-------------DPDVP--------------------------HYLYVMPYE--------------------------RLHKIF---EYRV-------FV----------------KDLR-----------------IVLISPR-------------YQCM--ITDV---------------------------------------------------TTDIVRFVKVFLLEH---------------------------------------------------IIQHMD--------------------------------------VTDLIIDLYC------------------------------------------DVDEEIC---------------------SSILLLDMQPLPAAESTLEDLCV------LGADV---------LK-ERLRP-----------------------------------------------------------------------------------------------------DAVLEPL------------------------------------------------------------------FVNPTVERISAVG---------------------------------RAT--------------------------------------------------------------TNNTY--PAELTSG----------------MISG---------------------THTEIIELIRQATRKTEIP
GLP15_1394_Glam_308162018            CKSVYDKFR------YVNWPGP----------------------------------------------------------------------------------------------LMKYTIQRKTI---------------------LIPPE-------VCK---------------------------------EIVSDD---------------------------------------------------------------------MIMT-----------------------------------------------------------------------------------------SINA------------------------------------PALHKFCESLDL---------------VN------------GKFLKVDWAAPTDC---------------------------------------------------------------SQIV--ET------------------------------------LMCIVPSNAF------------------ILLK-M---SEK-IRAAL----------------------MNFHNEEKPDKF-------------DPSVP--------------------------HYLYVMPYE--------------------------RLHKIF---EYRI-------FI----------------KDLR-----------------IVLIAPR------------YQCMI--TDV----------------------------------------------------TTDIVRFVKVFLFEH---------------------------------------------------ITQQMG--------------------------------------VTDLIIDLYC------------------------------------------DVDEDIC---------------------SSILLLDMQPLPTAESAFEDLCV------LGAAA---------LK-ERLKP-----------------------------------------------------------------------------------------------------DAVLDPL------------------------------------------------------------------FVNPTIERISAVG---------------------------------RAT--------------------------------------------------------------TNNTY--PSELTSG----------------MISG---------------------THTEIIELVRQATRKTEIP
GL50581_2165_Gint_253744363          CKSIYDKFR------YVNWPGP----------------------------------------------------------------------------------------------LMKYTIQRKTI---------------------LIPPE-------VCK---------------------------------EIVSDD---------------------------------------------------------------------MVMT-----------------------------------------------------------------------------------------SVNA------------------------------------PALHKFCENLDL---------------LN------------GKFLKVDWAAPTDC---------------------------------------------------------------SQIV--ET------------------------------------LMCIVPSNAF------------------ILLK-M---SEK-IRNAI----------------------LNFHNEDAPKVF-------------DPDVP--------------------------HYLYVMPYE--------------------------RLHKIF---EYRV-------FV----------------KDLR-----------------IVLISPR------------YQCMI--TDV----------------------------------------------------TTDIVRFVKVFLLEH---------------------------------------------------IIQHMD--------------------------------------VTDLIIDLYC------------------------------------------DVDEEIC---------------------SSILLLDMQPL-P----------------AAEST---------LE-DLCVLGADVLKERLRP------------------------------------------------------------------------------------------DAVLEPL------------------------------------------------------------------FVNPTVERISAVG---------------------------------RAT--------------------------------------------------------------TNNTY--PAELTSG----------------MISG---------------------THTEIIELIRQATRKTEIP
TVAG_384640_Tvag_123414063           MEEIIQKCS------FDKWYPI----------------------------------------------------------------------------------------------LRDDTIRSKII---------------------ELPDD-------FKEFLLFHE---------------------------FIIEDG------------------------------------------------------------------------------------------------------------------------------------------------------------------KFK-------------------------------------DLEKRVNDAIDE---------------LGG-----------VAFPKLNFTAPTDS---------------------------------------------------------------NWIG--FGKS----------------------------------IEVRSFKDLV------------------YVFK-A---STR-MLIDI----------------------TCPFN--VQ----------------NVEIK--------------------------PIIVLKKWF--------------------------NYKVNR---EFRI-------FMRD--------------SEHF-----------------FV--SSRNISVP-------YYLTQ--EEV----------------------------------------------------REGAQT-----MVNL---------------------------------------------------ISQKIH--------------------------------------PYRIIIDFYI------------------------------------------SPK-------------------------MRPHVIDIAPWTD----------------VTSPL---------LY-SWSEL-----------------------------------------------------------------------------------------------------ELLAQTEVRLTTDLNVQP-------------------------------------------------------PEDAAVPSD-------------------------------------MADGR------------------------------------------------------------KLEEI---------------------------------------------------MASLKEFKDEFNDNVHDK
cgd6_1830_Cpar_66475464              DKNKLCDMG------ISKWYERLSNY------------------------------------------------------------------------------------------KSKVTIDGILF---------------------NIPDS-------LILE--------------------------------YFELDG---------------------------------------------------------------------FDINYE-----------------------------------------------ALSE------------------------------------SSR-------------------------------------KCLEDINKSIRE---------------NTEFFSM-------GFSPKFTWSAPTDA---------------------------------------------------------------TWIN--SNRS----------------------------------ICCRTLEELF------------------ILLK-A---STK-VSEDI----------------------DR-----AK----------------EGNIS--------------------------NVLLLREYVP-------------------------TLNEMF---EFRV-------FIGGCSLH----------SEYK-----------------ILGISQRHICY--------YYKEL--SENFKL-------------------------------------------------RTDIKSCIMEFFLYSKKELILE--------------------------------------------LFDIFN--------------------------------------SMCIAFDIYISNY---------------------------------------KDK-------------------------LSILIIDVQPL-L----------------HASPL---------LF-NLFELK----------------------------------------------------------------------------------------------------LNLLTEEGTELGF------------------------------------------------------------DILRTTENN-------------------------------------STKNMINN---------------------------------------------------------SYLKGFVPDELLS-----------------VSNS---------------------DFYNNETLDK------LEW
_Dpro_654515672                      MLKKRTLKQ----YGVSKWYST----------------------------------------------------------------------------------------------LADYTFPTTFV---------------------KLKET-------ELD---------------------------------LLAQGI---------------------------------------------------------------------VDEC-----------------------------------------------------------------------------------------EVY-------------------------------------HIVNRIAHAQQA---------------FFG-----------SKFVFADTIAPTDT---------------------------------------------------------------TRFI--SK-R----------------------------------GSIHSAASAW------------------KNLA-S---SEK-V---------------------------------RT----------------AAKNREF------------------------ECICVRPFR--------------------------SMNRSR---EFRL-------FI----------------NEGN-----------------LILMSQYWLDR--------HYHKL--ESK----------------------------------------------------KDFYWTKAEEL-LDE---------------------------------------------------ISWLLP--------------------------------------KETIVLDLYF------------------------------------------TSK-------------------------DQIILIDFNPWGP----------------PTLPL---------LAESWNIDWTEEIG-----------------------------------------------------------------------------------------------IKIVQPE--------------------------------------------------------------------------------------------------------------------------------------------------------------------------------------------------------------------------------------------------------DQT
_Lara_494490064                      ---------------ISEWYPF----------------------------------------------------------------------------------------------LGHLTFPTVFL---------------------ALSSE-------ECD---------------------------------IVAQGK------------------------------------------------------------------------------------------------------------------------------------------------------------------SLA-------------------------------------SLEERITRAIAS---------------LPG-----------SCVVGLDSCMPTDS---------------------------------------------------------------V--S--FQKS----------------------------------KSLKNGKLAL------------------KILN-E---SEK-V---------------------------------KS----------------AVSSGD-------------------------KTITVRPYR--------------------------RMDKTR---EFRL-------FV----------------KDGE-----------------LKAMTQRNLER--------HFKRL--EAR----------------------------------------------------RELYWQKGVSFVDE----------------------------------------------------IKKYIN--------------------------------------EKDYVVDIYF------------------------------------------TST-------------------------ADIMIVDFNSWGE----------------PTLPLL--------MK-SWERDWSSVDGLKLMA------------------------------------------------------------------------------------------EPIQ-----------------------------------------------------------------------------------------------------------------------------------------------------------------------------------------------------------------------------------------------------------LGG
GSOID_T00020807001_Odio_313240620    ---KFEKKDVL-WCQLQNWYPI----------------------------------------------------------------------------------------------FKRFCPKTEFF---------------------QLDKC-------ALN---------------------------------YLKSDGS--------------------------------------------------------------------IILPEEEATPASPQPDIFGQVD-------------------------------SDNAFSDWSTD----------------------------EESESVPHINPVP----------------------------DLCRNIRRVIGER--------------ARK-----------SVVPKLNWSCPSDA---------------------------------------------------------------LHFS--PDQS----------------------------------LKCMSPNEVF------------------LLLK-S---SDK-INHDL----------------------TYPFENCHNTTEA------------DESPV--------------------------YTLALQEFA--------------------------KLAPSR---EFRC-------FI----------------REKQ-----------------PVAICSRSTT---------FSDDE--EDF----------------------------------------------------FDEVYEALLEFIDLQ---------------------------------------------------IIPKVE--------------------------------------LSSFVVDVYK------------------------------------------KEA-------------------------DGFLIFGFSPFGL----------------PTWSM---------YF-SWN-----------------------------------------------------------------------------------------------------------YTM------------------------------------------------------------------KILELRSQD-------------------------------------GGA--------------------------------------------------------------RGAAQ------------------------------------------------------------------HEK
GSOID_T00020813001_Odio_313240625    -TYKFEKKDVL-WCQLQNWYPI----------------------------------------------------------------------------------------------FKRFCPKTEFF---------------------QLDKC-------ALN---------------------------------YLKSDGS--------------------------------------------------------------------IILPEEEATPASPQPDIFGQVDSDNAFS-------------------------DWSTDEESESV----------------------------PHINPVP----------------------------------DLCRNIRRVIGER--------------ARK-----------SVVPKLNWSCPSDA---------------------------------------------------------------LHFS--PDQS----------------------------------LKCMSPNEVF------------------LLLK-S---SDK-INHDL----------------------TYPFENCHNTTEA------------DESPV--------------------------YTLALQEFA--------------------------KLAPSR---EFRC-------FI----------------REKQ-----------------PVAICSRSTT---------FSDDE--EDF----------------------------------------------------FDEVYEALLEFIDLQ---------------------------------------------------IIPKVE--------------------------------------LSSFVVDVYK------------------------------------------KEA-------------------------DGFLIFGFSPFGL----------------PTWSM---------YF-SWKEL-----------------------------------------------------------------------------------------------------YDENFGVEE----------------------------------------------------------------PVYRFNEGE-------------------------------------QVIG-------------------------------------------------------------LSQDNRVPLDAIH-----------------ISQGL--------------------DSQKLVDYMK------LVK
GSOID_T00008885001_Odio_313225989    -KKDVLWCQ------LQNWYPI----------------------------------------------------------------------------------------------FKRFCPKTEFF---------------------QLDKC-------ALN---------------------------------YLKSDGS--------------------------------------------------------------------IILPEEEATPAGPQPDIFGQVD-------------------------------SDNAFSDWSTD----------------------------EESEFVPHINPVP----------------------------DLCRNIRRVIGER--------------ARK-----------SVVPKLNWSCPSDA---------------------------------------------------------------LHFS--PDQS----------------------------------LKCMSPNEVF------------------LLLK-S---SDK-INHDL----------------------TYPFENCHNTTEA------------DESPV--------------------------YTLALQEFA--------------------------KLAPSR---EFRC-------FI----------------REKQ-----------------PVAICSRSTT---------FSDDE--EDF----------------------------------------------------FDEVYEAILEFIDLQ---------------------------------------------------IIPKVE--------------------------------------LSSFVVDVYK------------------------------------------KEA-------------------------DGFLIFGFSPFGL----------------PTWSM---------YF-SWKEL-----------------------------------------------------------------------------------------------------YDENFGVEE----------------------------------------------------------------PVYRFNEGE-------------------------------------QVIG-------------------------------------------------------------LSQDNRVPLDAIH-----------------ISQGLDSQKLV--------------DYMKLVKEGN------LDG
LOC100115146_Nvit_156550406          MKTENSQVQ----CSFCNWYPL----------------------------------------------------------------------------------------------FSKNALEAVTL---------------------PLPVE-------VCK---------------------------------YLEHDAFLLPVEATSSG----------------------------------------------------------LPSNSEWSDGSAVN---------------------------------------HDESDQESEEQ----------------------------PTFP-------------------------------------DFSRQIQEVIDD---------------FG------------AVFVKSNWSTPSDA---------------------------------------------------------------TWVA--ATKT----------------------------------LKCNSLEDVY------------------LLLK-S---SDR-ISHDL----------------------SSIKNSNNK----------------DSPLT--------------------------PCLVLKRWR--------------------------DIDPCT---EFRC-------FV----------------VNKE-----------------LVGICQRDISQ--------YYRHI--ENE----------------------------------------------------KYDIQRDIKSLFMEK---------------------------------------------------IKDKFS--------------------------------------VDNYSFDVIR------------------------------------------YKK-------------------------DKVKIVDFGLLNES---------------VTKGT---------LF-TLEEL-----------------------------------------------------------------------------------------------------QSEVPEA------------------------------------------------------------------PEFRFIAEDMGI----------------------------------QPK--------------------------------------------------------------TTRHFCIPQEVNEFFQAT------------GGMS---------------------VIDAIRKEVEEQGRLSNDD
EMIHUDRAFT_115862_Ehux_551582952     -SSDLLSCG------IDRWYPD----------------------------------------------------------------------------------------------LRRLTIRSVLV---------------------PLPSD-------FAA---------------------------------YLVADG---------------------------------------------------------------------VVLPGG-----------------------------------------------DDEAGASPAGEEADDESADGSATLALP------------ASFE-------------------------------------AVRRAIDDGIER---------------LGG-----------AVFVKLNWSAPRDA---------------------------------------------------------------AWVL--GG-S----------------------------------LKCTSSEDVL------------------LLLQ-S---SDR-AAHDL----------------------TEARRMCEDAAAAGEGGAGVD----AAGEPHG------------------------FVLALRKWC--------------------------ELQPSR---EFRC-------FRG---------------AGGR-----------------LLAACQRDRFS--------HFPEL--GAR----------------------------------------------------RSHDLGLLEAFGRE-----------------------------------------------------LPSAP--------------------------------------ARV-VWDAYV------------------------------------------DRN-------------------------DKVHLVDLAPFHE----------------STDPL---------LF-EWEEL-----------------------------------------------------------------------------------------------------VALDDAAASP---------------------------------------------------------------PQLRLVAEGHI-----------------------------------APS--------------------------------------------------------------ASIYNGWPQELQQ-----------------LGQQ---------------------DLESLIQTAQ------KAA
EMIHUDRAFT_106575_Ehux_551543656     LSSDLLSCG------IDRWYPD----------------------------------------------------------------------------------------------LRRLTIRSVLV---------------------PLPPD-------FAA---------------------------------YLVADG---------------------------------------------------------------------VVLPGG-----------------------------------------------DDEAGASPAGEEADDESADGSAALALP------------ASFE-------------------------------------AVRRAIDDGIER---------------LGG-----------AVFVKLNWSAPRDA---------------------------------------------------------------AWVL--GG-S----------------------------------LKCTSSEDVL------------------LLLQ-S---SDR-AAHDL----------------------TEARRMCEDAAAAGEGGAGVDAA--GEPHG--------------------------FVLALRKWC--------------------------ELQPSR---EFRC-------FRG---------------AGGR-----------------LLAACQRDRFS--------HFPEL--GAR----------------------------------------------------RSRDLGLLEAF-------------------------------------------------------GRELPSA-------------------------------------PARVVWDAYV------------------------------------------DRN-------------------------DKVHLVDLAPFHE----------------STDPL---------LF-EWEEL-----------------------------------------------------------------------------------------------------VALDDAAASP---------------------------------------------------------------PQLRLVAEGHI-----------------------------------APS--------------------------------------------------------------ASIYNGWPQELQQ-----------------LGQQ---------------------DLESLIQTAQKAA---KSA
CDC123_Scer_74644913                 ----VTRAQVE-HCSYSFWSSL----------------------------------------------------------------------------------------------YPKYVPKSIVLK--------------------SLPKK-------FIQ---------------------------------YLEQDG---------------------------------------------------------------------IKLPQEENSRSVYTEEIIRN---------------------------------EDNDYSDWEDDEDTATEFVQEVEPL--------------IDFP-------------------------------------ELHQKLKDALNE---------------LG------------AVAPKLNWSAPRDA---------------------------------------------------------------TWIL--PNNT----------------------------------MKCNEVNELY------------------LLLN-A---SNY-IMHDL----------------------QRAFKGCVD----------------GDDIKGLK-----------------------FDLVLRQWC--------------------------DMNPAL---EFRV-------FV----------------KNAH-----------------IVGATQRDLN---------YYDYL--DEL----------------------------------------------------SDTFKDLIDEIVHDV---------------------------------------------------VLPKFP--------------------------------------DKSFVLDVYI------------------------------------------PRPF------------------------NKIFIVDINPFAR----------------KTDSL---------LF-SWNEIAAIAP------------------------------------------------------------------------------------------------PKNDVED------------------------------------------------------------------YELRLVTRHNTGRF--------------------------------ASK--------------------------------------------------------------EHSENHVPQDLVE-----------------ASLNPE-------------------AIRELTQKWKELLSQQAKE
CDC123_Scer_6323244                  ---PVTRAQVE-HCSYSFWSSL----------------------------------------------------------------------------------------------YPKYVPKSIVLK--------------------SLPKK-------FIQ---------------------------------YLEQDG---------------------------------------------------------------------IKLPQEENSRSVYTEEIIRNEDNDYSDWE------------------------DDEDTATEFVQEVEPL-----------------------IDFP-------------------------------------ELHQKLKDALNE---------------LG------------AVAPKLNWSAPRDA---------------------------------------------------------------TWIL--PNNT----------------------------------MKCNEVNELY------------------LLLN-A---SNY-IMHDL----------------------QRAFKGCVD----------------GDDIKGLK-----------------------FDLVLRQWC--------------------------DMNPAL---EFRV-------FV----------------KNAH-----------------IVGATQRDLN---------YYDYL--DEL----------------------------------------------------SDTFKDLIDEIVHDV---------------------------------------------------VLPKFP--------------------------------------DKSFVLDVYI------------------------------------------PRPF------------------------NKIFIVDINPFAR----------------KTDSL---------LF-SWNEI-----------------------------------------------------------------------------------------------------AAIAPPKNDVED-------------------------------------------------------------YELRLVTRHNTGRF--------------------------------ASK--------------------------------------------------------------EHSENHVPQDLVE-----------------ASLNPE-------------------AIRELTQKWKELLSQQAKE
_Ssp._640615598                      -PFAISQTF------PENWPAP----------------------------------------------------------------------------------------------LKNLAAPMTSI---------------------VLTPE-------DAS---------------------------------ALGAFS---------------------------------------------------------------------TAFRQQ-----------------------------------------------FDVR------------------------------------ARDFLSK---------------------------------ALLTEIDSALLK---------------YPE-----------GVMPRIGYCSWKASLIEKR----------------------------------------------------------AAKT--RNDV----------------------------------MQIITTDD------------------------------PR-VGNAL----------------------SV-----LV----------------GSDDP--------------------------VVLHLRAWR--------------------------KIPDWS---EVRL-------FF----------------KNGR-----------------FVGASQYAYRR--------SFPEI--TSN----------------------------------------------------AKEIETMVARA-ASV---------------------------------------------------IVEKLH--------------------------------------LSDVIVDLALL-----------------------------------------PSDGKSPVSG------------------LKPLLIELNPFSP----------------LTDAC---------LF-SWEKG-----------------------------------------------------------------------------------------------------GNFDGR---------------------------------------------------------------------------------------------------------------------------------------------------------------------------------------------------------------------------------------------------------FRW
_Bsp._496403701                      MYSEYKKTF------IENWSED----------------------------------------------------------------------------------------------ILAQSFKSESI---------------------ELHER-------DVI---------------------------------AIGASTDEFM-----------------------------------------------------------------SARGLQ-----------------------------------------------EKPF------------------------------------FSA-------------------------------------QLHDDIEYALSV---------------LNK-----------PAFVRFGGVSYHDA---------------------------------------------------------------SLSR--LD----------------------------------------TVDGIV------------------KQLS-V---SNR-C--------------------------VASYLWDCL----------------QSSTP--------------------------VWLFLREWR--------------------------DIPRWG---EFRC-------FI----------------RDAK-----------------VIGVSQYHCLE--------YFPFL--KEK----------------------------------------------------ENEIRLQLIMF-LQK---------------------------------------------------LLPVLH--------------------------------------MNSVVADIAIDYQ---------------------------------------DGK-------------------------FTTTLIELNPFIQ----------------RTDAC---------LF-SW----------------------------------------------------------------------------------------------------------VNGGD------------------------------------------------------------------FNGRIRVNHS------------------------------------IAD--------------------------------------------------------------AHAEK------------------------------------------------------------------RKR
Q664_25730_Cvio_667801279            YFDRVSPTF------IENWAPA----------------------------------------------------------------------------------------------LHDLSIPQADV---------------------PLSRL-------ETQ---------------------------------ALEART---------------------------------------------------------------------WHQKST-----------------------------------------------DRSE------------------------------------HIPID-----------------------------------SLMWRLNVALRQ---------------FPE-----------GAFVRLGSRSSKDS--------------------------------------------------------------AYALQ--HG------------------------------------LRVRTGEAAV------------------RMLT-EG--SER-TAFDL----------------------RL-----AA----------------QVHYA--------------------------PHIFVRKWM--------------------------DIPRWT---EFRC-------FM----------------KERR-----------------LVGISQYDCLTLG------IRPEI--TRN----------------------------------------------------AESLHTAIREF-SQR---------------------------------------------------FAEASH--------------------------------------LDNVVFDVFVE-----------------------------------------GMDTSGP---------------------LNVRLLELNPFFP----------------KTDPC---------LF-DWSVP-----------------------------------------------------------------------------------------------------ADFDGS---------------------------------------------------------------------FRFLGASTL-----------------------------------KGR--------------------------------------------------------------DHLGH------------------------------------------------------------------GRG
LACBIDRAFT_309209_Lbic_170089331     LFPNQTPSYIL-AFQFSSWYPK----------------------------------------------------------------------------------------------FSNQSIKSTIVR--------------------PLSQD-------FIN---------------------------------YLNSES---------------------------------------------------------------------VFVPEGSEDLPAESTL-------------------------------------SDESEDEEEQDDDEPAPRR--------------------YAFP-------------------------------------ELDKQIRNCVKE---------------YG------------AIFPKLNFSSPKDA---------------------------------------------------------------SWIL--PSSSP---------------------------------LKCTSPADIY------------------LLLK-S---SDF-ISHDLGV--------------------ENVFNGCLPAT--------------SSSSPHPSWPEYQ------------------LELVLRKWY--------------------------PVDTSR---ELRC-------FV----------------RQNR-----------------LLAISQRDTN---------YYDFF--NDLPT--------------------------------------------------QAKIIHTVETFWQSI---------------------------------------------------RSKWYT--------------------------------------QEDYIFDFLL------------------------------------------TRDL------------------------SRGHILDFNPYAP----------------RTDPL---------LF-TYQELC----------------------------------------------------------------------------------------------------DLLLSDD------------------------------------------------------------------TRPKLRVID-------------------------------------SPSHPAAMSKAP-----------------------------------------------------AHQHNMIPFEALS-----------------LGNGR--------------------NIEEFAEIWK-ETVKESRI
CDC123_Hsap_221316620                KKEHVLHCQ------FSAWYPF----------------------------------------------------------------------------------------------FRGVTIKSVIL---------------------PLPQN-------VKD---------------------------------YLLDDG---------------------------------------------------------------------TLVVSGRDDPPTHSQPDSDDEA-------------------------------EEIQWSDDENTATLTA-----------------------PEFP-------------------------------------EFATKVQEAINS---------------LGG-----------SVFPKLNWSAPRDA---------------------------------------------------------------YWIA--MNSS----------------------------------LKCKTLSDIF------------------LLFK-S---SDF-ITRDF----------------------TQPFIHCTD----------------DSPDPCIE-----------------------YELVLRKWC--------------------------ELIPGA---EFRC-------FV----------------KENK-----------------LIGISQRDYTQ--------YYDHI--SKQ----------------------------------------------------KEEIRRCIQDFFKKH---------------------------------------------------IQYKFL--------------------------------------DEDFVFDIYR------------------------------------------DSR-------------------------GKVWLIDFNPFGE----------------VTDSL---------LF-TWEELIS---------------------------------------------------------------------------------------------------ENNLNGDFSEVDAQEQDS-------------------------------------------------------PAFRCTNSEVTV----------------------------------QPS--------------------------------------------------------------PYLSYRLPKDFVD-----------------LSTGE--------------------DAHKLIDFLKLKRNQQEDD
Pmar_PMAR022513_Pmar_294938762       APDVVLACT-------EVWAPL----------------------------------------------------------------------------------------------LEGLAIECSVI---------------------DIDQD-------FVD---------------------------------YLNEDGLDGGG----------------------------------------------------------------LVMGRR-----------------------------------------------DSDSDSDWEYDGPDSYPTGSTLA----------------ERFP-------------------------------------VLNTRVEEAIGG---------------FGG-----------FAFPKLSWSAPTDA---------------------------------------------------------------EWVR--GPDG----------------------------------MKCRNLDEIT------------------VLLK-A---SDF-AQFDL----------------------QRLAIPESQ----------------RQLVPL-------------------------PKLILKPFIE-------------------------PFHETL---EFRC-------FV----------------RGGT-----------------LRALCPRHLTG--------HLPFL--YDMMYG-------------------------------------------------QEQGLEELRRFIDDQ---------------------------------------------------VMRRLSSGD-----------------------------------YDKVVIDVY-------------------------------------------KHR-------------------------KRYVVVDLAPWDEE---------------LTDPL---------LY-TWEEL-----------------------------------------------------------------------------------------------------EEFDGDV------------------------------------------------------------------EWREVPKGA-------------------------------------MPTNPSAV---------------------------------------------------------RKRASRMPVEVAQQLL--------------LHGG---------------------DVEKLVD---------SLR
TTHERM_00059240_Tthe_586745569       KEGHDKKQDMW-KYNQVNWPEQ----------------------------------------------------------------------------------------------LKKLTYKTKYI---------------------KVDQN-------LYD---------------------------------YLEEDGIYIHPRYQKNSSQNYLLEKNKDFHLQQQKNEEEQNKQD-------------------------------DSFNQD-----------------------------------------------YDDEDHDFKFR----------------------------IFSK-------------------------------------EFEEQVKEAFDD---------------FE------------SVFVKLNWRAPRDT---------------------------------------------------------------ENWL--HG------------------------------------LQIDNLEDLL------------------TVLK-S---SGI-LAELLEEYKDQFGLCSTINTENQFEQVQQ-----QI----------------DSQMG--------------------------LYLILKKWY--------------------------NLHKHM---MVRC-------FV----------------RNKK-----------------LIAISQRHCSS--------VNRTI--EEF----------------------------------------------------GDLFSQKISSYFYEK---------------------------------------------------IAPFFE--------------------------------------ENNYVFDLYIGI----------------------------------------APN-------------------------YKLRLLDINPWRG----------------HTNPL---------LL-DWDQL-----------------------------------------------------------------------------------------------------EKIDTEEFGQDKKIF----------------------------------------------------------IQYVRTESDV------------------------------------IIE--------------------------------------------------------------DLSAYRVPEDMLF-----------------DGENAQE------------------SMEKVFDYLN------IHN
BN9_014190_Acan_635371235            MPPSYSRGQVD-RCAFDHWYSK----------------------------------------------------------------------------------------------LKHVAIKGYAI---------------------SLPPE-------FVE---------------------------------YLLEDG---------------------------------------------------------------------IQLPKDY----------------------------------------------LASLSHSDSRI----------------------------QAIQ-------------------------------------QIQYQVENQFKE---------------LKG-----------KSFIKLNWSSPRDA---------------------------------------------------------------KWIL--ST------------------------------------LQCRTFDDII------------------LLLK-S---SDF-TTHDL----------------------LYPYRACTD----------------AASPETCGIANKA------------------PCLVMKKWC--------------------------NLHDSM---LFRG-------FV----------------LHHA-----------------LVAISQRHCEA--------CYPFL--ALK----------------------------------------------------KDSLRALIKSFFLKH---------------------------------------------------LQPLTVAPAHL---------------------------------DSDFVFDIYI------------------------------------------DKN-------------------------DRVYLVDINVFGE----------------VTDPL---------LF-SYNELQSFN-------------------------------------------------------------------------------------------------RQEATSS------------------------------------------------------------------IAFRIVENEQSI----------------------------------QPN--------------------------------------------------------------SLATYRVPVDFMQ-----------------HLST---------------------DNAMEAFYRQVERDNASSD
AlNc14C3G408_Alai_325179898          SAPSYSRGHVD-HCAFDHWYPK----------------------------------------------------------------------------------------------LKHVAIKGCII---------------------SLPPP-------FVQ---------------------------------YLLEDG---------------------------------------------------------------------IHLPTDCIASLP-----------------------------------------QSED--SNARI----------------------------RAIQ-------------------------------------QVQVQVEKQFKE---------------LKG-----------KSFIKLNWSSPRDA---------------------------------------------------------------KWIL--ST------------------------------------LQCHTFDDIV------------------LLLK-S---SDF-ITHDL----------------------LYPYRFCKD----------------ATSSETRGIANTS------------------PCLVMKKWC--------------------------NLHDSM---LFRG-------FV----------------RHHA-----------------LIAMSQRHCEA--------CYPFL--SLR----------------------------------------------------KRSLRSLIEIFFRKH---------------------------------------------------LEPLTLEPAHL---------------------------------DSDFSFDVYI------------------------------------------DKN-------------------------DRVYLVDINVYGE----------------ITNPL---------LF-SYKELQS---------------------------------------------------------------------------------------------------LQETTSS------------------------------------------------------------------IAFRIVETKRSI----------------------------------QPN--------------------------------------------------------------SLAKYRVPIDFMQ-----------------HLST---------------------DNAMEAFYQQVKRDNASDE
F443_03685_Ppar_566030451            TGPSYSAAQVN-NCAFAAWYPQ----------------------------------------------------------------------------------------------LKRVCIKGEVV---------------------PLPTE-------FVS---------------------------------LLLADG---------------------------------------------------------------------VTLPSAPVASGIV----------------------------------------DEDDDEEVDAA----------------------------SALT-------------------------------------DDQLAVISSVKRKVEKVLQD-------FGG-----------KLFPKTNWSAPRDA---------------------------------------------------------------AWML--GS------------------------------------LKCTNFEDIF------------------LLLQ-A---SDF-VVHDL----------------------TQPYIGCSGENTE------------NSPTE--------------------------SYLVLKKWC--------------------------NFLDSM---LFRC-------FV----------------VGRR-----------------LVAVSQRNCDE--------FYEFL--PDQ----------------------------------------------------QDELCELLYEFYKKNFRKAE----------------------------------------------GEFLFP--------------------------------------DPNYSFDVYV------------------------------------------DKR-------------------------HRVYLLDINVFGA----------------VTDTL---------LF-SWEEL-----------------------------------------------------------------------------------------------------LEFQTESPATPQDTEDEHHV-----------------------------------------------------VDFRVVESKKGI----------------------------------RAN--------------------------------------------------------------PLSGYRAPTDLVD-----------------HLAG---------------------GAGFEAFIEQVKRDNAADS
PHYSODRAFT_528425_Psoj_348667590     ----YTAAQVD-NCAFAAWYPQ----------------------------------------------------------------------------------------------LKRVSIKGEVV---------------------PLPAA-------FVS---------------------------------LLLSDG---------------------------------------------------------------------VTLPSAPVASGI-----------------------------------------EDEDGDDDEED----------------------------AASALTDEQLSIIS---------------------------TVREQVERVLED---------------FSG-----------KLFPKTNWSAPRDA---------------------------------------------------------------AWML--GS------------------------------------LKCTSFEDVF------------------LLLQ-A---SDF-VVHDL----------------------TQPYIGCSGEKAE------------SAPSE--------------------------SYLVLKKWC--------------------------NFLDSM---LFRC-------FV----------------VGHR-----------------LVAVSQRNCDE--------FYEFL--PDQ----------------------------------------------------QDELCELLYEFYKTNFKKDDG---------------------------------------------GEFVFP--------------------------------------DPNYSFDVYV------------------------------------------DKR-------------------------RRVYLLDINVFGA----------------VTDTL---------LF-SWEELLELQKDGLATPQ-----------------------------------------------------------------------------------------DAEDEGHM-----------------------------------------------------------------IDFRVVESKKGI----------------------------------RAN--------------------------------------------------------------PLSGYRAPTDLVD-----------------HLAG---------------------GAGFDAFIEQVKRDNAAGG
OXYTRI_10769_Otri_403338747          YRLRIIKTQ------FQEWYPY----------------------------------------------------------------------------------------------FKKHSFKAEII---------------------ELSPR-------FID---------------------------------YLNQDG---------------------------------------------------------------------VFLPEQ-----------------------------------------------EPEQDSDSDDSQEEDSKQSKDSQSINKIQDPEVRQAFL-QEVS-------------------------------------EIRKRIGRIIEK---------------WEN-----------GVFVKLNWSAPRDA---------------------------------------------------------------EWLN--PT------------------------------------FQSISADEIF------------------TLLK-S---SQF-VAHDY----------------------SCPYHEAIPGF--------------TLPNP--------------------------HYLIIKRWH--------------------------NLNLSM---EFRV-------FV----------------KNHT-----------------LVGISQRDCTA--------FYQFL--YDEVN--------------------------------------------------NRQIARNISEFFNNS---------------------------------------------------IKQSMMDYCSE---------------------------------MSDYVIDVYVDV----------------------------------------PPK-------------------------SRVWLIDINPWIPE---------------SVDSL---------LF-QWDEL-----------------------------------------------------------------------------------------------------NQKDLSK------------------------------------------------------------------IQFDGRIRV-------------------------------------VENNGQIMPSDV-----------------------------------------------------NQFKV--PADFED-----------------M------------------------------DRMQEFMKNMNDK
CDC123_Amis_564264160                KKEQVLHCQ------FSAWYPR----------------------------------------------------------------------------------------------FRALTIRSAIL---------------------PLPQN-------VQE---------------------------------YLLDDG---------------------------------------------------------------------TLVVSGREDPPTHTQEDSGEDEA------------------------------EEIQWSDDENTTTLKA-----------------------PEFP-------------------------------------EFALKVQEAISS---------------LGG-----------SVFPKLNWSAPRDA---------------------------------------------------------------YWIA--MNSS----------------------------------LKCKTLSDIF------------------LLLK-S---SDF-ITRDF----------------------TQPFIHCTD----------------DSPDPSLD-----------------------YELVLRKWC--------------------------ELIPGA---EFRC-------FV----------------KENK-----------------LIGISQRDYTQ--------YYEHI--SKQ----------------------------------------------------HEEISRSIQEFFQKH---------------------------------------------------IQYKFL--------------------------------------DEDFVFDVYR------------------------------------------DSK-------------------------GKVWLIDFNPFGE----------------VTDSL---------LF-TWEELTS---------------------------------------------------------------------------------------------------GQNVKGDHGEGEATEQDY-------------------------------------------------------PAFRCTNSGVTV----------------------------------QPS--------------------------------------------------------------PYLSYGLPKDFVD-----------------LSTGE--------------------DVHKLIDLLKLKRNQQDDD
_Pbac_655450018                      LLVKPTRKHVD-NCQFTSWFSLHG--------------------------------------------------------------------------------------------IKKVTFRSITI---------------------PIPEE-------FVE---------------------------------YLNEDG---------------------------------------------------------------------VILPKVPDGVSVNPFDPRYVRETKDDDWAD-----------------------EDYEDYDDSDESDEEGGAAATK-----------------KSFP-------------------------------------GFEAAILKAIEK---------------LGG-----------KVFVKTNWSSPRDA---------------------------------------------------------------VWVS--GT------------------------------------LQCQSVGEII------------------LLLK-S---SDF-VSFDL----------------------EHAYDECRE----------------ESTEGESVRNASKRPEH--------------FCLVLRKWC--------------------------NLFPSQ---EFRC-------FV----------------KDRQ-----------------LIATSQRDPTK--------FYNFL--PSN----------------------------------------------------VSTYSPLVSEFFDTS---------------------------------------------------IRPVFP--------------------------------------DDNFSFDVYI------------------------------------------DRK-------------------------HRVWLQDFNVFSR----------------ATNAL---------LF-TWAEL-----------------------------------------------------------------------------------------------------LAVDTQEADAVA-------------------------------------------------------------NEFRVVQDPSGV----------------------------------QSS--------------------------------------------------------------QLAAHKVPADVVN-----------------DPNN---------------------FIKEFVESCR------NGT
GLOINDRAFT_321036_Rirr_552934656     PFPPLTEQHIL-NCTFSSWYNN----------------------------------------------------------------------------------------------FRRVSIKSKIIK--------------------PLPEE-------FVE---------------------------------YLHADG---------------------------------------------------------------------VFLPEQNYLEGSHLREISDNDDDDFN---------------------------ANETDIDEDDENQPL------------------------PSFP-------------------------------------DLEQQIWDIIDE---------------FDG-----------SVFPKLNWSSPRDA---------------------------------------------------------------TWIS--ATNT----------------------------------LKCNSPSDIF------------------LLLK-S---SDF-IAHDL----------------------DHAFDDCYYDN--------------QSDSRRHRPNE--------------------FELVLRKWY--------------------------DVAPSM---EFRC-------FV----------------KEEE-----------------LVAISQRDVN---------YYSFL--NDI----------------------------------------------------KEELETKIIQFFETH---------------------------------------------------VQNKFF--------------------------------------NRDYVFDVYV------------------------------------------TRNR------------------------ERVWLIDFNPFGP----------------MTDGL---------MY-TWEEIL----------------------------------------------------------------------------------------------------TATGPPS------------------------------------------------------------------FRLITSQTEASQS---------------------------------RSR--------------------------------------------------------------PFAVNRYPREIFD-----------------LSQGQ--------------------TIAEFAEQFQRELAIAVSS
NEMVEDRAFT_v1g233898_Nvec_156408387  KQQHVENCN------FSSWYPR----------------------------------------------------------------------------------------------FKNVTIRSKII---------------------PLSKE-------FVD---------------------------------YLKTDG---------------------------------------------------------------------VVLPGKPSSLPR-----------------------------------------HEDDESDSEEWQNLEEDPEQATVEA--------------PEFN-------------------------------------DIDTKIKEAIQE---------------LGG-----------EVFPKLNWSAPRDA---------------------------------------------------------------SWIS--HDNT----------------------------------LRCKSPGDIY------------------LLLK-S---SDT-IDRVL----------------------CDAFIHCEDNST-------------QTHDS--------------------------FELILRKWQ--------------------------NIYPAM---EFRC-------FV----------------RNNE-----------------LVAISQRDISN--------YYHFL--AEN----------------------------------------------------EDEICADILNFYESK---------------------------------------------------IAEKFP--------------------------------------DTSYVFDVYK------------------------------------------YAD-------------------------QKCTLIDFSPYGV----------------PTNPL---------LF-TWSELDT---------------------------------------------------------------------------------------------------EVVPDL--------------------------------------------------------------------LFKVVPSAIGV----------------------------------QPG--------------------------------------------------------------PFACSRLPQDMVD-----------------LTSGA--------------------DVNKLVDFLNVGNLIRRPG
CDC123_Phod_556753625                KKEHVLHCQ------FSAWYPL----------------------------------------------------------------------------------------------FRSLTIKSVIL---------------------PLPQN-------VKD---------------------------------YLLDDG---------------------------------------------------------------------TLVVSGREDPPARSQPDSDDEA-------------------------------EEIQWSDDENTATLTA-----------------------PEFP-------------------------------------EFTTKVQEAINS---------------LGG-----------SVFPKLNWSAPRDA---------------------------------------------------------------YWIA--MNSS----------------------------------LKCKTLSDIF------------------LLFK-S---SDF-ITRDF----------------------TQPFIHCTD----------------DSPDPCME-----------------------YELVLRKWC--------------------------ELIPGA---EFRC-------FV----------------KENK-----------------LIGISQRDYTQ--------YYDHI--SKQ----------------------------------------------------KEEICRCIQDFFRKH---------------------------------------------------IQYKFL--------------------------------------DEDFVFDIYR------------------------------------------DSR-------------------------GKVWLIDFNPFGE----------------VTDSL---------LF-TWDELLS---------------------------------------------------------------------------------------------------GTNLKGDFSEGEALEQDA-------------------------------------------------------PAFRCTNSEVTV----------------------------------QPS--------------------------------------------------------------PYLSYRLPKDFVD-----------------LSTGE--------------------DAHKLIDFLKLKRNQQEDD
LOC577449_Spur_115920254             NRQHILNCS------FSSWYAN----------------------------------------------------------------------------------------------FVEHTIESEVI---------------------KLPQS-------FVN---------------------------------YLLEDG---------------------------------------------------------------------IYLPLSKDEIAE-----------------------------------------QNIQDVGSSKVR---------------------------PSFP-------------------------------------DLQAKVERAINQ---------------LGG-----------EVFPKLNWSAPRDA---------------------------------------------------------------SWIA--CGNS----------------------------------LKCHTFNDII------------------LLLK-S---SNF-ISHDL----------------------TEPFTLCDETEQ-------------AGQVQ--------------------------YELVLRRWT--------------------------EIPPSM---EFRV-------FV----------------GNQE-----------------VIAISQRDCSS--------FFPCV--PPL----------------------------------------------------VDDITYEINNFHDRY---------------------------------------------------VAGLFK--------------------------------------EQHYVLDVFR------------------------------------------KDK-------------------------AEFLIVDFNPFNE----------------VTDSS---------LF-TWTELYR---------------------------------------------------------------------------------------------------MRDGDEE------------------------------------------------------------------CDLRCIKEEAGV----------------------------------QPS--------------------------------------------------------------PYLAYSMPSDFLD-----------------LRSGT--------------------DANKLVDFLKMEVQRQDDS
cdc123_Drer_41152285                 KKEQVVNCQ------FSVWYPL----------------------------------------------------------------------------------------------FKKHTIKSLIL---------------------PIPQN-------VID---------------------------------YLLDDG---------------------------------------------------------------------TLVVSGSENNNSQTQANNSDSDE------------------------------EDIQWTDDETTTTVTA-----------------------PEFP-------------------------------------EFNVKVQEAINV---------------LGG-----------CIFPKLNWSAPRDA---------------------------------------------------------------NWIA--LNSS----------------------------------LQCQSLSEIF------------------LLFK-S---SDF-ITHDL----------------------TQPFLHCSD----------------DSPDPTIN-----------------------YELVLRKWS--------------------------ELIPGG---EFRC-------FV----------------KENK-----------------LIAICQRDYTQ--------HYQHI--GKQ----------------------------------------------------EASISTSILQFFRDN---------------------------------------------------IQYQFP--------------------------------------DEDFVLDVYR------------------------------------------DSS-------------------------GRVWLIDFNPFGE----------------VTDSL---------LF-TWEELTS---------------------------------------------------------------------------------------------------GKNLTANQTQEETALPDG-------------------------------------------------------PAFRCTNSEVTV----------------------------------QPS--------------------------------------------------------------PCLSYRIPRDFLD-----------------LTTGE--------------------DAYKLIDFLKLKRGQQEEE
TTHERM_00059240_Tthe_118348362       KKQDMWKYN------QVNWPEQ----------------------------------------------------------------------------------------------LKKLTYKTKYI---------------------KVDQN-------LYD---------------------------------YLEEDGIYIHPRYQKNSSQNYLLEKNKDFHLQQQKNEEEQNKQD-------------------------------DSFNQD-----------------------------------------------YDDEDHDFKFR----------------------------IFSK-------------------------------------EFEEQVKEAFDD---------------FE------------SVFVKLNWRAPRDT---------------------------------------------------------------ENWL--HG------------------------------------LQIDNLEDLL------------------TVLK-S---SGI-LAELLEEYKDQFGLCSTIN--------TE---NQFE----------------QVQQQIDSQMG--------------------LYLILKKWY--------------------------NLHKHM---MVRC-------FV----------------RNKK-----------------LIAISQRHCSS--------VNRTI--EEF----------------------------------------------------GDLFSQKISSYFYEK---------------------------------------------------IAPFFE--------------------------------------ENNYVFDLYIGI----------------------------------------APN-------------------------YKLRLLDINPWRG----------------HTNPL---------LL-DWDQL-----------------------------------------------------------------------------------------------------EKIDTEEFGQDKKIF----------------------------------------------------------IQYVRTESDV------------------------------------IIE--------------------------------------------------------------DLSAYRVPEDMLF-----------------DGENAQE------------------SMEKVFDYLNIHN---KDL
CDC123_Ggal_118081986                KKEQVAHCQ------FSVWYPL----------------------------------------------------------------------------------------------FRAVTIRSVIL---------------------PLPEN-------VKE---------------------------------YLLDDG---------------------------------------------------------------------TLVVSGREDPPTQTPEGSDDA--------------------------------EEIQWSDDENTATLKA-----------------------PEFP-------------------------------------EFTAKVEEAISS---------------LGG-----------SVFPKLNWSAPRDA---------------------------------------------------------------YWIA--MNSS----------------------------------LKCKALSDIF------------------LLFK-S---SDF-ITRDL----------------------TQPFIHCTD----------------DSPDPSLN-----------------------YELVLRKWC--------------------------ELIPGA---EFRC-------FV----------------KENK-----------------LIGISQRDYTQ--------YYDHI--SKQ----------------------------------------------------HEEICRSIQEFFKKH---------------------------------------------------IQYKFL--------------------------------------DEDFVFDVYR------------------------------------------DSR-------------------------GKIWLIDFNPFGE----------------VTDSL---------LF-TWDELTS---------------------------------------------------------------------------------------------------GRNLKGDQSEVEATEQDY-------------------------------------------------------PVFRCTNSQVTV----------------------------------QPS--------------------------------------------------------------PYLSYRLPKDFVD-----------------LSTGE--------------------DVHKLIDFLKLKRNQQDDD
GSTEN:00013253:G:001_Tnig_47218245   KKEQVVNCQ------FSVWYPI----------------------------------------------------------------------------------------------FKKHTIKSLIL---------------------PLPQN-------VIE---------------------------------YLLDDG---------------------------------------------------------------------TLVVAGSDHNTYTHCNSDLNAE-------------------------------EDIQWSDDETTATVTA-----------------------PEFP-------------------------------------EFSSHVLEAINA---------------LGG-----------SVFPKLNWSAPRDA---------------------------------------------------------------NWIA--LNSS----------------------------------LQCHSLSDIF------------------LLFK-S---SDF-ITHDL----------------------TQPFLLCSDQ---------------DSPDPVIH-----------------------YELVLRKWS--------------------------ELIPGG---EFRC-------FV----------------KENK-----------------LIAVSQRDHTQ--------FYQHI--PKQ----------------------------------------------------EEQICQAIQLFFSQN---------------------------------------------------IQYNFL--------------------------------------DEDFVFDVYR------------------------------------------DSQ-------------------------GRVWLIDLNPFGE----------------VTDAL---------LF-SWEELTS---------------------------------------------------------------------------------------------------GELTQQQEG----------------------------------------------------------------PAFRYATSEVTV----------------------------------QPS--------------------------------------------------------------PCLSYRIPRDFVD-----------------LSTGE--------------------DAYKLIDFLKLKKRQQEDS
cdc123_Xlae_148236867                KKEQVLNCQ------FGQWYPT----------------------------------------------------------------------------------------------FKKFSIRSVII---------------------PLPEN-------VKD---------------------------------YLLDDG---------------------------------------------------------------------TLVVSGREESPGCSQRDLNCTEE------------------------------DEVQWSDDESTATLTA-----------------------PEFP-------------------------------------EFSIKVQEAINS---------------LGG-----------SVFPKLNWSSPRDA---------------------------------------------------------------YWIA--LNSS----------------------------------LKCTTLSDIF------------------LLFK-S---SDF-VTHDF----------------------TQPFIYCAD----------------DSPDPNIK-----------------------YELVLRKWC--------------------------ELIPGA---EFRC-------FV----------------RENK-----------------LIGISQRDYTQ--------YYDHI--SKQ----------------------------------------------------KEEIRKSIQYFFQEH---------------------------------------------------IQYNFP--------------------------------------DEDFVFDIYK------------------------------------------DSQ-------------------------GKIWLIDFNPFGE----------------VTDSL---------LF-TWEELRRNLCDVPGD-------------------------------------------------------------------------------------------VENEDQDC-----------------------------------------------------------------PTFRYTNREVTV----------------------------------QPS--------------------------------------------------------------PYLSYRLPKDFVD-----------------LSTGE--------------------DAHKLIDFLKLNRNRQDED
BBOV_III007300_Bbov_527101724        ----------------------------------------------------------------------------------------------------------------------------------------------------PPGTD-------NIS---------------------------------YASIES---------------------------------------------------------------------DTDTSSYP---------------------------------------------SD------------------------------------------------------------------------------NELTDTEADME----------------QFN-----------WEVIKTFTDELRTSIDNYGSVVPCVNGTYLDDA--------------------------------------------LWVT--NY-T----------------------------------VECSNVREPL------------------MQLK-S---STY-WHDRA-------------------------------------------------QDS--------------------------F-LTLFPGR--------------------------HFNNHY---QIRC-------FV----------------LEHE-----------------LVCIEQLFVHE--------NFEFM--AKD----------------------------------------------------AHGLVTLLKQF-------------------------------------------------------SVPIVNLD------------------------------------TPKAIFDVTI------------------------------------------SRELT-----------------------D-PRLVDIYPFGA----------------VADGLIHLEDVYHFYY-TRVKD-----------------------------------------------------------------------------------------------------GIEPRDK------------------------------------------------------------------VDMDIIDGVLVVFVGCN-----------------------------ASR--------------------------------------------------------------LKKHAWCPKDVGN-----------------ISFS---------------------TPDDLIDYLR------FQT
BBOV_III007300_Bbov_156088905        PPGVTAWDH------FSQWAHGTGFGSLKVDRDARTQLLELIQAHSFNETSAYYIAPLESRLYILWHWVSASYTVEFGLPLALPQGHQSASETLGTTS------------------LKHMRIPSHRM---------------------LLDKE-------VLR---------------------------------YLTSDR---------------------------------------------------------------------MVFPPGTDNISYSSIESDTDTSSYPS----------------------------D------------------------------------------------------------------------------NELTDSEADME----------------QFN-----------WEVIKTFTDELRTSIDNYGSVVPCVNGTYLDDA--------------------------------------------LWVT--NY-T----------------------------------IECSNVRELL------------------MQLK-S---STY-WHDRA-------------------------------------------------QGS--------------------------SHLTLLPGR--------------------------HFNNHY---QLRC-------FV----------------LEHE-----------------LVCIEQLFVHE--------NFEFM--AKD----------------------------------------------------AHRLVTLLKQF-------------------------------------------------------SVPILNID------------------------------------TPNAIFDVTI------------------------------------------SREL------------------------TDPRLVDIYPFGA----------------VADGLLHLEDVY--HF-YYTRV-----------------------------------------------------------------------------------------------------KDGIEPSDK----------------------------------------------------------------VDMEIIDGVLVVFVGCN-----------------------------ASR--------------------------------------------------------------LKKHAWCPKDVGN-----------------LSFS---------------------TPDDLIDYLR------FQT
Cdc123_Mmus_19527052                 KKEHVSHCQ------FSAWYPL----------------------------------------------------------------------------------------------FRSLTIKSVIL---------------------PLPQN-------VKD---------------------------------YLLDDG---------------------------------------------------------------------TLVVSGREDPPTCSQSDSGNEA-------------------------------EETQWSDDESTATLTA-----------------------PEFP-------------------------------------EFNTQVQEAINS---------------LGG-----------SVFPKLNWSAPRDA---------------------------------------------------------------YWIA--MNSS----------------------------------LKCKTLSDIF------------------LLFK-S---SDF-ITHDF----------------------TQPFIHCTD----------------DSPDPCIE-----------------------YELVLRKWC--------------------------ELIPGA---EFRC-------FV----------------KENK-----------------LIGISQRDYTQ--------YYDHI--SKQ----------------------------------------------------KEEICRCIQDFFKEH---------------------------------------------------LQYKFL--------------------------------------DEDFVFDIYR------------------------------------------DSR-------------------------GKVWLIDFNPFGE----------------VTDSL---------LF-TWEELTS---------------------------------------------------------------------------------------------------ENNLRGEVTEGDAQEQDS-------------------------------------------------------PAFRCTNSEVTV----------------------------------QPS--------------------------------------------------------------PYLSFGLPKDFVD-----------------LSTGE--------------------DAHKLIDFLKLKRNEQEDD
GSHART1_T00005550001_Psp._594147520  PHATFTAAS---------------------------------------------------------------------------------------------------------------------------------------------TSP-------FFC---------------------------------YLDSND---------------------------------------------------------------------TRLPAHPTLLPYAHPAEDYNTYVGEEVWDRPSGDGT-----------------DKAATDEAPADD---------------------------LRFDAVVAWVWQVWEREDWAKD------GVVLCG-------RYCVANDCAWAVSSNSPVIFTP-----REL-----------FMVMRNSVKFLRDI------------------------------------------------------------------------------------------------------------------HTQ------------------LHSS-S---SGN-AE---------------------------------------------------------------------------------AEFTIAKAL--------------------------AGEGAK---EMRV-------FL----------------PYRLTTNENNELHLSPPLEH-YAAVCQRLTDV--------CFPSL--MDWSEEEHHENFKVILK--------------------------------------RIQQAQLLERAYATNGK-------------------------------------------------LLPKLKEAFIRRVSRTTGSTKHQSGTAQRGESQNLILMLAVDILFEGSSFPIYILSAKMRLFFVKLADSLNQEVGSDKDAVVGDLPYIKELLNSDSDDN-------------------------DICDTNDLNINDDV---------------SSFSS---------LS-SISEN-----------------------------------------------------------------------------------------------------DETNESL------------------------------------------------------------------LEFFRLIRD-------------------------------------PSNWNRYVRVMAERYASSFTSLQQTCNIQDEAGSPCLIPLEEGNPMVNHYCIVASDPMDLITCRGDLRMRGLPLEFID-----------------PQLLQANP-----------------EAMEFLRNMRDALVNNQSE
STCU_08515_Scul_528219429            -PKSLSPFFLL-PSSVSSLSGPQLGCTCWYLMSSSNSACLSSYLFFFFFFKVVHKYIYIPIRLHRRPMLHRIPLTTVLSAP-----------------------------------GSRHRVPSRTYHA-------------------DVSNP-------FFR---------------------------------YLDSNDTRVVRHPLVDVS---------------------------------------------------------AAAPEDY----------------------------------------------ETYVGEDVWQRGERRDDDGDEATDDPP---PRFDDVVEWVAATMEEPW-------ARD---------GVVLCG-------RYAVADDAGAWALPSQTASIHHP----HEV-----------FVLMKSSIKFLRDV---------------------------------------------------------------------------------------------------------------RAQFRH------------------AAAA-A---SAG-TKEPS------------------------------------------------------------------------------LQFTLAKAM--------------------------SGEEAR---EMRV-------YV----------------PYAVHPCGDGAAPLQLAPLYDYAAICQRCTDV--------CLPPL--MAWTEAQ------------------------------------------------HDAHFRAMLARVRTAAVLERH---------------------------------------------LAADPSFLPRLLPPGAATAAAADPWAPF----------------LLLLAVDVLF------------------------------------------DGPGHPIYVLSAKVRVVARRAPADAASDAPYLLEDAASDAP----------------AVAER---------AE-LEEESAARFFRLFRDVAHWNTYIAEVERRRRAAGEDGGEVARQ---------------------------------------------------------------LRRDTDADAKAVAEAGDGAPPRESAATG---------------------------------------------LHYVVVASEFGDLVH-------------------------------TGE--------------------------------------------------------------AVEKMGLPLEFFQPE---------------LLQG---------------------NPELLAIIEQMKRDMEKAK
Tb11.01.0660_Tbru_71756087           MDFRLPLCS------VLNSEV-----------------------------------------------------------------------------------------------GRRHKIPHRTL---------------------SLPKTSL-----FVR---------------------------------YLDSDD---------------------------------------------------------------------TRLPVHSLLNIVPQPAEDY----------------------------------ETYTGEDDWEKKVDDAAGVVGDCGGIDGNPPRFDEMVQWVIDALKD---------IEGCGGAASD--GVVLCG-------RWVVADDCSWVVPSRTPILHSP-----RDV-----------FLAMRNSPKFLRDV---------------------------------------------------------------------------------------------------------------HHQILS-------------------GDT-S---SAG-MGST-------------------------------------------------------------------------------VELTLAKAV--------------------------GKNPAN---DFRV-------FV----------------PYRLVRAEDTFRVTIWEGRV-FAGVCQRSTDV--------CFPSL--MSWDLRTHSENYDHVLR--------------------------------------HIEGARLLERSIEADPYLRTFLLRKVEANEVEGAASPCSVLLLSVDVVFEGVSLPLYIVSAKVRCFKHDRLR--------------------------------------DPLCVSPFIK------------------------------------------EED-------------------------NNGSEEGVNAVGG----------------GVDGD---------RS-DGIDT-----------------------------------------------------------------------------------------------------KEMLDDSVIFFRLFRDVNNWNFHVTLCLHPDGGEVSGDVDKGVNIVPSPQRD---------------------RRFFVIASE-------------------------------------RSDLVLTKE--------------------------------------------------------PILKRGMPLEFLRP----------------ELLG---------------------DIGEGLPQVW------KDR
Tc00.1047053508601.70_Tcru_71421453  MDLRVPLSY------VLDTPL-----------------------------------------------------------------------------------------------AQRHAIPHRTLFL-------------------DATHP-------FIQ---------------------------------YLNSDD---------------------------------------------------------------------TRLPQHPTLTVEARPVDEYETYVGEDDWRR-----------------------EKAVDSDNTDPA------------------PRFDDVVQWVSETLNE---------IGGNGD------GVVLCG-------RRVVADDCGWAVFSRTPTLLSS-----RDV-----------FIAMRNSSKFLRDL---------------------------------------------------------------------------------------------------------------HHQLQG------------------VALA-G---AEG-PVK--------------------------------------------------------------------------------VEVTLAKAL--------------------------SGNNAR---EFRA-------FL----------------PYRLHTSAGDGCVEAWAGHM-YAGISQRATDV--------CFPSL--MAWDEATHDENYAIVMRHIEHAKLLERALENDALLRTALFRQANKPSVADPAPKKKSSVVLLAVDVLFESPSLPVYLLSAAVRCF------------------------------------HHDDVE--------------------------------------DPACAFPFIL------------------------------------------DDDAKLGRGNDGN---------------TTTTTTNIANNNN----------------NNNSK---------AD-GWSGE-----------------------------------------------------------------------------------------------------SEDDDDDLLSDSVSFFRLFRDAANWNRHVEVWRRRGSTAGVGERSDTTRTQKE--------------------RRFFVVASERGDLAS-------------------------------TKE--------------------------------------------------------------YMAKRGLPLEFFR-----------------PDLFKGDGN----------------FLREWRERLMQRISMAEQS
CAPTEDRAFT_169051_Ctel_443686486     KNNEVVRCM------FSEWFPT----------------------------------------------------------------------------------------------FKTLTISSRVI---------------------SLPDD-------VRE---------------------------------YLLADG---------------------------------------------------------------------FVLPKECEADSSVDV--------------------------------------RDGWTDDDEEDAER-------------------------PHFP-------------------------------------VFHESLLSAISA---------------LGG-----------TVFPKLTWSAPQDA---------------------------------------------------------------TWVS--FNNS----------------------------------MECKSPSDIY------------------LLLK-S---STF-VSHDL----------------------TEAFRYCDDG---------------PVPVS--------------------------HSLVLRKWT--------------------------DVDPSS---EFRC-------FV----------------FDQT-----------------LIAISQRHTKA--------YFSHL--APL----------------------------------------------------NHQIIADIGNFYRSE---------------------------------------------------ICDKFP--------------------------------------LQQYIFDVIW------------------------------------------KNN-------------------------GVVTLVDFNPFGR----------------TTDAL---------LF-SWDSDLAQGQISQLLS------------------------------------------------------------------------------------------AALDKGE------------------------------------------------------------------PTLRCLESEADA----------------------------------SVD--------------------------------------------------------------PFQCYRVPQDFVH-----------------LASGE--------------------DPEKLLDLLNLKIQQQNEE
TOT_040000293_Tori_403223783         -WSSVGTSP------YHEWASLNSEEADKS--------------------------------------------------------------------------------------YKSLYLTNKGI---------------------ELTGS-------VLE---------------------------------YLSSDV---------------------------------------------------------------------MVLPNHLKSAKFSHV--------------------------------------EEYSELSEYSDSDSLEGSESVFDFSS-------------GSFA-------------------------------------TFFNKVTETIKE---------------FGG-----------LALPYLNGSYLQDG---------------------------------------------------------------SWII--NN-S----------------------------------TVCNDLRDVV------------------LLLK-G---STA-WQNEL----------------------VN-----TPV---------------ASEVRDK------------------------HELKLYLYKIP------------------------HFVKSV---QFRL-------FV----------------HEAQ-----------------ILLISQLFFNN--------VYDYL--LKEEA--------------------------------------------------FSELYEQIVKFYNHN---------------------------------------------------LVRRLPKD------------------------------------VSKVVVDLYII-----------------------------------------NRT-------------------------DEIYVTNVSPWYLNNENIFTWEEIQEY--VTSER---------PF-DHDESI----------------------------------------------------------------------------------------------------KVTYFGS------------------------------------------------------------------VAFAILLSNKLSR---------------------------------HKL--------------------------------------------------------------YSFCS------------------------------------------------------------------EDV
TA10770_Tann_84997113                ALSSFFHNI---------WAPIGLLTNRELYLFSLTIPFEK---------------------------------------------------------------------------YKKHWLNNYTI---------------------ELSCA-------VYE---------------------------------YLKSDI---------------------------------------------------------------------MVMPDDLKSTKFSNV--------------------------------------EDYSDFSEYSDYDECEVRFDFSS----------------TEFQ-------------------------------------DFFSAISESIKS---------------FGG-----------SVLPYLNGSYLQDG---------------------------------------------------------------SWIT--NN-T----------------------------------TLCTDLRDVI------------------LLLK-S---STN-W---------------------------------HG----------------DSDSI--------------------------LKLFLYKVI--------------------------NHNNST---QFKL-------FY----------------YDRE-----------------IIIISQLFLNH--------VYDSL--INNKQ--------------------------------------------------SHKLIRRILEFSNHK---------------------------------------------------LLHLIPEN------------------------------------LLNFVVDLYI------------------------------------------LNKS------------------------DEIYIMNIAPFNFNHEIIFPWDQIYEY--ATSRT---------PF-THDES-----------------------------------------------------------------------------------------------------VKVDYFG------------------------------------------------------------------NIAFVVMSSNK-----------------------------------LSR--------------------------------------------------------------HKLYS------------------------------------------------------------------FCS
TP04_0637_Tpar_71029258              ----LSSFI------HNIWAPIGLLTNRELYLFSLTVPFDK---------------------------------------------------------------------------YKKYWLNNYSI---------------------ELSQT-------VYE---------------------------------YLKSDV---------------------------------------------------------------------MVMPDDLKSTKFSNV--------------------------------------EEYSDFSEYSDYDECEIRFDFNS----------------PEFL-------------------------------------DFFSAISESIKS---------------FGG-----------LVLPYLNGSYLQDG---------------------------------------------------------------SWII--NN-T----------------------------------TVCTDLRDVI------------------LLLK-S---STK-W---------------------------------HG----------------DSDSI--------------------------LKLFLYKVI--------------------------NQNHST---QFKL-------FY----------------YDRE-----------------IIIISQLFLNH--------VYDSL--ISNKH--------------------------------------------------SHKLIRRILEFSNHK---------------------------------------------------LIHLIPEN------------------------------------LSNFVVDLYIL-----------------------------------------NRT-------------------------DEIYIMNIVPFNFNSEIIFTWDQIYEY--ATSRT---------PF-THDES-----------------------------------------------------------------------------------------------------IKIDYYG------------------------------------------------------------------NIAFVVMSSNK-----------------------------------LSR--------------------------------------------------------------HKLYSFCSEDVEE-----------------L----------------------------------------AKI
YALI0F20394g_Ylip_50556506           ----LTREHLL-NCQFSAWYKL----------------------------------------------------------------------------------------------YKSITPKTRIIK--------------------PLPED-------FVN---------------------------------YLSEDG---------------------------------------------------------------------VILPDEEKTSYGSDSGVF-----------------------------------EEYSDDEDELD----------------------------SYVSKLD----------------------------------DFHPKVQAVIDE---------------FG------------AVAPKLNWSSPQDA---------------------------------------------------------------IWIS--PSNS----------------------------------TRCVTVNDVY------------------LLLK-S---SDY-IAHDL----------------------TM-----LDK---------------LGGIPKDFS----------------------FELVLRKWI--------------------------NINPAL---EFRC-------FV----------------KDRE-----------------LIAVTQRDQN---------YYEFL--IKL----------------------------------------------------KERFLGEIELFFYEH---------------------------------------------------IKDTFD--------------------------------------PSSFVFDVYI------------------------------------------PEPY------------------------DKVWLMDFNVFYP----------------TTDSL---------I--EWSTL-----------------------------------------------------------------------------------------------------VNLDATS------------------------------------------------------------------PSFDLDLLL-------------------------------------IDKELRNASFACQ----------------------------------------------------RHSQNKVPMDIVN-----------------ASMDTE-------------------AMVEMLRSQQREMDRLDGD
BEWA_006780_Bequ_510904826           CGEWVHEFELG-LLEYENWYNI----------------------------------------------------------------------------------------------YKKWTLSHKMI---------------------EFEGK-------FVE---------------------------------YLKSDT---------------------------------------------------------------------MVLPEEFSHTTYAKV--------------------------------------DSDSDFSGYSDQGVIA-----------------------ENFDFQDHKLS------------------------------SGISQLKSAIVE---------------FNK-----------GSLPCINGSYLLDG---------------------------------------------------------------VWIL--NN-T----------------------------------NICRDVRDLL------------------LLIK-S---STE-W---------------------------------QT----------------MELDK--------------------------IKLFLYRAK--------------------------MFSKST---QFRV-------YI----------------YDFE-----------------IVVIAQLFLNQ--------VYDYLLNADQ----------------------------------------------------RKAFQDRIVHFYREH---------------------------------------------------ILGQIPP-------------------------------------VRNFVLDLYI------------------------------------------VEE-------------------------H-VYLLDIKPW-K----------------YHPLI---------LF-TWQNLYEFATCRKSFETCSSST------------------------------------------------------------------------------------QVSINDN------------------------------------------------------------------IAFVILTSD-------------------------------------NYS--------------------------------------------------------------KHLYN------------------------------------------------------------------LHP
EIN_114160_Einv_471191846            RLRELFGEY---------------------------------------------------------------------------------------------------------------MIPGEVH---------------------LLPDD-------MKT---------------------------------IILEEMGGSVIWDGVEE----------------------------------------------------------VSETTSI----------------------------------------------FGVCKGDDETF----------------------------EVGHL------------------------------------WFDELIKKAIEK---------------YG------------EVFLRLNKVALLDS---------------------------------------------------------------EWMN--GS------------------------------------LSIHNSRDAL------------------TLLQ-A---SER-ANISL-----------------------------------------------DSHSP--------------------------NELEIVQYV--------------------------SINPNQ---EFRC-------FV----------------IQNT-----------------LCAIIQRYTDI--------FTSSI--EKQ----------------------------------------------------KTQIVTAICSLYDNM---------------------------------------------------HQTNLN--------------------------------------IENYTFDVMV------------------------------------------KGD--------------------------KATLIDADELDEY---------------HTKNT---------LE-GFSTL-----------------------------------------------------------------------------------------------------DEIKEAKVK----------------------------------------------------------------PVFLYVKEQNQV----------------------------------QPS--------------------------------------------------------------VKLFQGIPQEFFN-----------------D---------------------------KVIETFN------KDP
UM04862.1_Umay_71021557              PNSALYWTS------YGTWYPI----------------------------------------------------------------------------------------------FRKHAPKSTVI---------------------DIGTV-------QPELIH------------------------------WLDSDT---------------------------------------------------------------------FILPEGSGPGTSSAQICSSSCSSSSCSSSAQA---------------------ESEEDAQIKEA----------------------------IQLS-------------------------------------ALDAEIRRVVSN---------------YDG-----------AVFPKLNWSAPLDS---------------------------------------------------------------GFML--PGNN----------------------------------LQCLHPEDVY------------------LLLK-S---SEF-VGRDLEQISHM----------------TAPCSGASDVQG-------------AQSVG--------------------------PQLVLKKWF--------------------------GLNKSY---EFRC-------FV----------------RSHS-----------------LIAITQRDVT---------FFDHL--QSRTL--------------------------------------------------QSQIKNAICTFWTDVLLAAK----------------------------------------------QHAKWQ--------------------------------------LADYAFDVYL------------------------------------------TKDL------------------------GRVWLVDINAWLP----------------RTDPL---------LF-DFQELDALHRAD----------------------------------------------------------------------------------------------KARKTTL------------------------------------------------------------------PSATLVHEQ-------------------------------------QQTLQTAQNLPF-----------------------------------------------------TRSNH--------------------------AAG---------------------QDRFQHVEFRILSDRRLQS
GSPATT00017041001_Ptet_145528546     NMNQFCRVN---------WPKG----------------------------------------------------------------------------------------------LKS--FKSKSI---------------------RLSQE-------VVS---------------------------------YLKEDG---------------------------------------------------------------------ILLNDV-----------------------------------------------WKRPPLQDNGT----------------------------KRFP-------------------------------------ELEQLVNEILDD---------------FE------------SVFIKLNWRAPLDC---------------------------------------------------------------QNTF--QD------------------------------------MCFQDLYDIM------------------MALK-Y---SGV-IMEMI----------------------ED-----YDEQVIDQNHPEKCQLVAQPSQG--------------------------YLLELKKYY--------------------------KLRPNA---EFRC-------FV----------------KNKK-----------------LIGISQK-------------NLYL--ITE----------------------------------------------------DESVKDKIQNY-FNK---------------------------------------------------IVDLIE--------------------------------------IDNYVLDVYIDI----------------------------------------PPK-------------------------ENIILVDLNPWQE----------------HTRPK---------LF-TYEEL-----------------------------------------------------------------------------------------------------DIFQECQ------------------------------------------------------------------LRLVKNKDMI------------------------------------VQE--------------------------------------------------------------DYSGYRESVDIET-----------------L-----------------------------IQQQQQEKQQLQQQ
RFI_02506_Rfil_569434815             LVASYRAFQYNEAYSISQWIGNDE--------------------------------------------------------------------------------------------LRKHTFDTCVL---------------------PVSLE-------ESK---------------------------------HLLIYFRYIQH----------------------------------------------------------------SQMSKR-----------------------------------------------TTSEWIEDYKKTQ--------------------------ESVDKYVS---------------------------------QVKSKIDTCLQTKQKV-----------WKD-----------GFFAKFNSRSPKDVFICEGQNPRLKQM--------------------------------------------------YWEK--LDDYLFSQ------------------------------LQQTTPKNDYVDSNEDKEEKKYHTDSKVWLFK-N---LEE-VIKEI----------------------IQ-----GQR---------------HLISS--------------------------TNAVLAYFHLITEEMCLHSSNDLINMFAKSFRCLEDLQKSV---SLGA-------YM----------------PDAENVISI------------VECLSQYYSSL--------YFPQL--CRD----------------------------------------------------KEQIGKEILEFFQTH---------------------------------------------------IESQLRAG------------------------------------HESYVIDFLRLETG--------------------------------------PNA-------------------------IKIYVIELNPFYA----------------QTGAA---------LF-NWKQHR----------------------------------------------------------------------------------------------------ELFMNGP------------------------------------------------------------------FEFRVRTEP-------------------------------------NEKCDEYLHQSWKISLDEYRNSEKTAK--------------------------------------PQSTD------------------------------------------------------------------NTN
ACA1_187390_Acas_470426125           KFNYLGAFD------IQRWYDAVAGEKELSG-------------------------------------------------------------------------------------GGGCTMRSRFV---------------------PLTVD-------QAR---------------------------------ALAKEH---------------------------------------------------------------------KRRRRL-----------------------------------------------GDEAEAEAGHAGGA-------------------------PEVIN------------------------------------ELERTIQNVIDTDF-------------NGG-----------AVFVRLSTQSPKDVGRGWTEHPRVVPIVREELA--------------------------------------------ATES--EGRTLNDRIRALFAASLRV-------------------MKVESGRGAL------------------DLLL-K---SER-IQSSV----------------------LH-----ALANAA------------EARWD--------------------------LCVVVRAWEE-------------------------SMRLDR---EFRT-------FV----------------VRDR-----------------VVAITQYNEYC--------HYPAW--ADQ----------------------------------------------------HQLIADKIHHLFVHQG--------------------------------------------------LRDKVPRAYR----------------------------------EWAYVADFVLLGEPEE------------------------------------RAD-------------------------LRVQLVEINPFGP----------------GTGAS---------LF-DWRTE-----------------------------------------------------------------------------------------------------RCLLQAG------------------------------------------------------------------HDLWGDLDSAAAAAAAA-----------------------------QPSDIDAWTKTVACRVGDGDDEQTVVVRMA-----------------------------------TRPNPRVDSSFLSSFGIDILV---------PALD---------------------DDHEEDDDDEDEDDDDEEE
ACA1_355720_Acas_470490276           KFNYLGAFD------IQRWYDAVAG-------------------------------------------------------------------------------------------EKELSGGGCTMRSRPGASPRQGAQAAAT----LGDEA-------EAE---------------------------------AGHAGG-----------------------------------------------------------------------------------------------------------------------------------AP----------------------------EVIN-------------------------------------ELERTIQNVIDTDF-------------NGG-----------AVFVRLSTQSPKDVGRGWTEHPRVVPIVREELA--------------------------------------------ATES--EGRTLNDRIRALFAASLRV-------------------MKVESGRGAL------------------DLLL-K---SER-IQSSV----------------------LHALANAAE----------------ARWDL--------------------------C-VVVRAWEE-------------------------SMRLDR---EFRT-------FV----------------VRDR-----------------VVAITQYNEYC--------HYPAW--ADQ----------------------------------------------------HQLIADKIHHLFVHQG--------------------------------------------------LRDKVPRAYR----------------------------------EWAYVADFVLLGE---------------------------------------PEERAD----------------------LRVQLVEINPFGP----------------GTGAS---------LF-DWHTERCLLQA-----------------------------------------------------------------------------------------------GHDLWGDLDSAAAAQPSDIDAWTKTVACRVGDGDDEQT-----------------------------------VVVRMATRP-------------------------------------NPR--------------------------------------------------------------VDSSF--LSSFGI-----------------DILVPALDD----------------DDEEDDDDED-EDDDDDEE
NAEGRDRAFT_50779_Ngru_290984195      RKTNTKSKQ---------------------------------------------------------------------------------------------------------------STNSRMI---------------------CSSDD-------DYQ---------------------------------HLLEETNFDTYFKVIEPFSFRSLQ---------------------------------------------------MTLERSEIK--------------------------------------------ALIDENEKFKK----------------------------CEFPQNEKDNVVEYDEWKVGENAKILN--------------NLADRISSKMKEMEE------------LSGRS---------GVFIRLSTLSPKDAAINRKGFVKLIHEEYLNILKESELLDDKSLDKSEK----------------------------TNII--MYALYRASISI---------------------------LKIYNGHEAI------------------QLLI-E---SKR-AQQEF----------------------KA-----ML----------------DSPTKSPLDLIIREVCLLVMNFQFVKV----LCNYFEKWN--------------------------DFDVAH---EFRA-------FV----------------YNRK-----------------ITGLTQYNPVV--------YFPTL--FKQ----------------------------------------------------KDQIKHLIISFILEN---------------------------------------------------IVNNAELP------------------------------------ISNYAIDIIL------------------------------------------VKDMTNGN--------------------LIVKIVELNPLAE----------------FTGTV---------LF-SWEED-----------------------------------------------------------------------------------------------------REILMGDCNANFE------------------------------------------------------------CEFRMLNGVAKFA---------------------------------AVN--------------------------------------------------------------CGPEW---------------------------------------------------------------QVALKK
NAEGRDRAFT_62166_Ngru_290999655      YNRLLSETS------FDHYYQA----------------------------------------------------------------------------------------------IKQFTFQSVSL---------------------KISVE-------EAK---------------------------------ALIAEFQDFSSNFR-------------------------------------------------------------RTQDTE-----------------------------------------------QANSEDNVIQY----------------------------EEWKNVNTDDHELNERRGILL--------------------NLCKRVDECAVD---------------LPNRED--------GFFCRMATMSPKDAATNRLGFISLVWKHYNELLKLEKEMNIDFKEQMNRNVYA------------------------LYKA--STSA----------------------------------LKLNNGMDAV------------------QLLV-E---SER-AQQEL----------------------NKIASGVYG----------------DATKT--------------------------NELILREWC--------------------------TFDVAH---EFRA-------FI----------------CNKK-----------------LTGITQYNPFV--------YFPQL--IKQ----------------------------------------------------KEDLQQLMRNFLEKD---------------------------------------------------IINNPAIQ------------------------------------VSNFIVDIIIVQ----------------------------------------DRNGE-----------------------KQVKIVELNPFAE----------------FAGTC---------LF-TWEND-----------------------------------------------------------------------------------------------------RDILEGKSPNHQ-------------------------------------------------------------IEFRLVTAC-------------------------------------VPNCEKE----------------------------------------------------------MGNEW------------------------------------------------------------------KDA
NAEGRDRAFT_57308_Ngru_290995536      NPSERYGFE----YDIDAWYEK----------------------------------------------------------------------------------------------CSKFTTYTKFI---------------------PITQS-------EAKV--------------------------------MIKQYEFHIKKTK--------------------------------------------------------------DQSPSG-----------------------------------------------DDYE------------------------------------ILK-------------------------------------KLEGKIDDELTN---------------NSEFYDEEMDVS--SAFIRLSSRSPKDAAFNSQKIRQILQRKLYEKNAMFSFGGENAKIQSDQDKQNN-----------------------EFIA--FFESQVEV------------------------------MKFESGQEAI------------------EMMT-S---STR-VYDDL----------------------NIALKYRND----------------DSLWN--------------------------VFFVLRKWIP-------------------------NHNIQY---EFRT-------FV----------------YNRK-----------------LCAISQYNDAL--------FFEDL--CNH----------------------------------------------------KDLYLKAMLNF-FEK---------------------------------------------------IKDEIP--------------------------------------FDNSVMDLVIYPLSS-------------------------------------DEEKLHDMDN------------------LNVQVLEFNPFNQ----------------YTGSA---------FF-SWIKD-----------------------------------------------------------------------------------------------------TEILKGEKP----------------------------------------------------------------FEFRIREDSLPVLRNIDFSE--------------------------SGS--------------------------------------------------------------TNKNNQLMFNLMDI----------------PDQSIIEENSELLEMVDDFLNNDLERIHSNFKYVVQKTTTESAS
ACA1_092760_Acas_470392261           TLPSFSLEK----WSYMSLFDPAYASVLVGRAAECWYED-----------------------------------------------------------------------------LKDFTFATAFM---------------------PVSKA-------QAT---------------------------------ALVHHY---------------------------------------------------------------------QQHQLSRTDALTP----------------------------------------DDEA------------------------------------QLA-------------------------------------QLEAELAHAMEEMKSSSARRELQGEE-EEM-----------SFFVRLSSRSPKDAGLNEGHPRIMEYLTEELDRFGGLAAADPNQKTVA-----------------------------MQRA--AGRI----------------------------------LRVTDAKQAL------------------WLIV-N---SER-TFTDM----------------------VH-----ALGHE-------------DGEWT--------------------------MKIVLRHWMD-------------------------GVDLAN---EFRG-------FV----------------CQGN-----------------LTALSQYNDGN--------YYPEL--MGK----------------------------------------------------EELIVGKISAY-WQK---------------------------------------------------VKHQVK--------------------------------------YQACIVDFVL------------------------------------------VNNM------------------------QDVYIVEINPFVHLPPLDSIPSRPFMYGPVTGGA---------LF-DWGTE-----------------------------------------------------------------------------------------------------RPLLQGECDVWGDLDEYQRKQKSLYGLGDADDGED--------------------------------------GEREVNEAPVRVQLRYQEYHHDDHDGEP------------------RPPV-------------------------------------------------------------LRLVHRVPSYLTD-----------------DYLMAF-------------------PILDLIGKWQQPGTSSAGA
NAEGRDRAFT_75417_Ngru_290973730      ------QEQ------ADKWFETNMDVWYDA--------------------------------------------------------------------------------------IVDLTFRTRMI---------------------SLSEE-------QIM---------------------------------AIILDN---------------------------------------------------------------------EYFNGECNQEVLETSGID-----------------------------------IVSSLNSDTRR----------------------------TVLE-------------------------------------TLEIDVNREMKE---------------LSCCNDE-------GCFIKLSCRSPKDAFAVCAKMKELFNDKIEKIVMEKKGVLATPNERLIA----------------------------VNES--FIQS----------------------------------MKVKDFAEEY------------------TYFT-K---SAR-VLEDL----------------------LLFLKYDKK----------------QREENP-------------------------IKIIIREWV--------------------------DIPSKY---EFRS-------FV----------------KNKQ-----------------LTAISQY-------------------------------------------------------------------------------------FDT----------------------------------------------------FAHRVP--------------------------------------LNDYICDFAI------------------------------------------DSN-------------------------GRVYIVELNPFST----------------TTDAC---------LF-SWTKD-----------------------------------------------------------------------------------------------------GEILNGIQIKNETNQLDQ-------------------------------------------------------IEFRLKKEVEKHLKH-------------------------------QII--------------------------------------------------------------SVWVP------------------------------------------------------------------FVI
LOC101849943_Acal_524893279          YQESVLDAN------MEEWLQY----------------------------------------------------------------------------------------------LKDFTFPSEFV---------------------PITID-------EAN---------------------------------MFVNVY---------------------------------------------------------------------RRLFANLDP--------------------------------------------SGISTISWKENLSAEEL----------------------VKVE-------------------------------------DISCRLQTAIDS---------------FTRKEEAK------FVFIKLSSRSPKDAPLAQDRFKSLYKHYLEKEQKEERFLENTQIKC-------------------------------LLKA--CFEA----------------------------------LRMSTADEAL------------------DACF-R---SER-TYQDL----------------------LL-----AL----------------AVPDRFR------------------------ENWVIRKFV--------------------------EIDVDM---EFRA-------FV----------------SNRR-----------------MTAISQYNFL---------YYSKR--LKEN---------------------------------------------------QQYYCDLIHRFYEQE---------------------------------------------------IAEKLQGF------------------------------------LHSFIIDFAVC-----------------------------------------GEG-------------------------SKIWVIEINPFLP----------------TTDSA---------LF-SWERE-----------------------------------------------------------------------------------------------------KQLLEGSHQD---------------------------------------------------------------LVFRVTEKP-------------------------------------RPG--------------------------------------------------------------AKSML------------------------------------------------------------------PAG
ACA1_171940_Acas_470524690           --------------------------------------------------------------------------------------------------------------------AKR--------------------------------EE-------ELR---------------------------------YWNSFS---------------------------------------------------------------------VHDIPDY----------------------------------------------DDCP------------------------------------LLM-------------------------------------GLEQRMNPIIEQYVK------------EGG-----------GAFVKLSDRSPKDAATERGRIYKFLELPEAKGGGALELSSNAVGLPA------------------------------VYRA--MMGS----------------------------------LKVKSGREAL------------------KLLL-I---SFR-TLEDVQIR-------------------LN-----YQ----------------DKLWE--------------------------LKLVIRQWV--------------------------DFDIAY---ELRG-------FV----------------CNNQ-----------------FTALSQYYYDC--------YFDVV--AQR----------------------------------------------------KDLIAASVQHYWENEARSVEPSI-------------------------------------------VKGKPP--------------------------------------YDSYVIDFTV------------------------------------------LPHRGDLAEGEL----------------LPVTIVELNPFDE----------------YTDSA---------LF-NWKLH-----------------------------------------------------------------------------------------------------RQTLHEGP-----------------------------------------------------------------FTFKIVDHPSVEAKRTWWKRILTTH---------------------AES--------------------------------------------------------------NAALQAAALPHEK-----------------DGETAEKTKAKEKTKTKETK-----KKRKEKKKEKKEKTDTKKK
NAEGRDRAFT_74519_Ngru_290975459      IKETYFGVNMD-KFITSFDEK-----------------------------------------------------------------------------------------------NET--MPTRTI---------------------PLTVQ-------QGK---------------------------------ALLHLC---------------------------------------------------------------------RHFKKG-----------------------------------------------TVMENEQDLMEL---------------------------KQVS-------------------------------------DNIEKIMKELNENN-------------YRD-----------GFIVKLTSRSPKDITPNHEKTMKKYRELLETPRFNPNQFTNVDLKRNAHWSA-------------------------LYFA--SLQC----------------------------------LRVYNAQEAI------------------SLLS-Q---SSR-IEYDL----------------------LL-----DLTF--------------ESDFS--------------------------LGLVVRPWDA-------------------------NMTLES---EFRG-------IV----------------YNGE-----------------LKCLSQYFTQC--------YLESL--EKD----------------------------------------------------KEEIEKHCKEFFSTT---------------------------------------------------IKPVLQEHAPE---------------------------------LTNYIVDFSL------------------------------------------TKNEANQ---------------------FSVKLLEINPYLT----------------TTGVG---------LF-DWEKD-----------------------------------------------------------------------------------------------------TEPLFENPKSQ--------------------------------------------------------------FEFRILTEP-------------------------------------IIS--------------------------------------------------------------QLLKD------------------------------------------------------------------PDS
ACA1_176070_Acas_470526872           --EAHFQVN------MDRWAPL----------------------------------------------------------------------------------------------IAEHTIPTLAL---------------------PLTPA-------DVR---------------------------------ALREHNTTHE-----------------------------------------------------------------QAQPTP-----------------------------------------------EDAS------------------------------------VLK-------------------------------------ELESRLDALVAA---------------VGGQDK--------GCFVKLSSRSPKDVTTKGDKFAAIYSSLLDQRRRSDSSSASSEEADKNARLGL------------------------LYRA--STEA----------------------------------MRVHSAAEGI------------------ALLL-Q---SRR-IYWDL----------------------TY-----FL----------------ALNDGLGAADDDDEDRERDNVDVGD------LAIVVRPWE--------------------------NIALQN---EFRG-------FV----------------HGGQ-----------------FTALSQYFTQL--------HFPEL--VER----------------------------------------------------RSELEQRIVAFWHRI---------------------------------------------------QHAFAA--------------------------------------YDKYVIDFAI------------------------------------------VEDDQGN---------------------EKMVVLELNPFNI----------------STGAA---------LF-SWESE-----------------------------------------------------------------------------------------------------DDSDTLEGKRP--------------------------------------------------------------FEFRVRQTPIAH----------------------------------TLR--------------------------------------------------------------NKLAP------------------------------------------------------------------EWR
ACA1_389340_Acas_470375829           CKDEVHAVN------IENWYSL----------------------------------------------------------------------------------------------LKEHTFATEFL---------------------DLSMD-------EARSD-------------------------------AL----------------------------------------------------------------------------------------------------------------------------------------------------------------------ALA-------------------------------------DLERKIDGLMGAFA-------------RDG-----------GAFVRLSTRSPKDAVISGGRLLHEWQVELARQGPEAERDENARLVA-------------------------------LIKA--STAA----------------------------------LKVRSGREAL------------------ALVK-A---SER-SNEDL----------------------LL-----ALEF--------------PHQWD--------------------------MKIIIRQWV--------------------------EMHPAM---EFRG-------FV----------------CGKK-----------------LTALSQYFHMA--------FFPAL--AAHKANTIIV---------------------------------------------QDEIARRIQAFFARH---------------------------------------------------IADLIP--------------------------------------LDNYVIDFGIASADDKEAVTALVAEGGGN-----------------------DHD-------------------------LGLLVIELNPFGA----------------GADPG---------LF-GWRQD-----------------------------------------------------------------------------------------------------RAVLEGDAP----------------------------------------------------------------FEFRVREAPFDV----------------------------------KPF--------------------------------------------------------------VICQW------------------------------------------------------------------REL
ACA1_389360_Acas_470375844           -TPPHIQEH---------------------------------------------------------------------------------------------------------------TFATEFL---------------------DLSMD-------EAR---------------------------------ALVREFDCQRGRQGQQ-----------------------------------------------------------QQQQHDQHAQ-------------------------------------------SDAL------------------------------------ALA-------------------------------------DLERKIDGLMGAFA-------------RDG-----------GAFVRLSTRSPKDA---------------------------------------------------------------VISS--ER-TLHEWQAELARQGPEAERDENARLVALIKASTAA-LKVRSGREAL------------------ALAK-A---SER-SNEDL----------------------LL-----ALEF--------------PHQWD--------------------------MKIITRQWV--------------------------EMHPAM---EFRG-------FV----------------CGKK-----------------LTALSQYFHIV--------HFPAL--AAH----------------------------------------------------KDEIARRIQAFFAER---------------------------------------------------IVDLIP--------------------------------------LDNYVIDFGIASA---------------------------------------TDDIVADGGG------------------GEVLVIELNPFND----------------YVGCGPVR------LA-ARP----------------------------------------------------------------------------------------------------------------------------------------------------------------------------------------------------------------------------------------------------------------------------------------------------------------------------------------------------------------------ARG
RFI_23982_Rfil_569376747             YRESYTLEK------WLSIPE-----------------------------------------------------------------------------------------------LEKHTFTTLFL---------------------QLTFE-------QADVFLASAR--------------------------YIQACISRDRSKNQT------------------------------------------------------------EFKSAA-----------------------------------------------IDDWNANKEAYE---------------------------KVLN-------------------------------------QLAQDIDTLLFETNKWD----------RSR-----------GFFAKFNSRSPKDVFNYQGGDKRLRELYFENLDELLFQTLNLPRRSRATPNEAVWA---------------------WYIT--TAKL----------------------------------LKMTCGKDVL------------------DTFS-A---SFR-SLEDL----------------------QA-----EI----------------ALGEKHMN-----------------------LSLAFRQWE--------------------------DKIPYTAHGEFRG-------FV----------------NNRQ-----------------LNAVTQYMSMIQF------PVHQT--LEK----------------------------------------------------REWYKEHITQFFESQ---------------------------------------------------VKEFVP--------------------------------------FDSYVIDFLV------------------------------------------FDD-------------------------GRVAIIEFNPFYT----------------KAGSG---------LF-AWKTDR----------------------------------------------------------------------------------------------------ELFLNGP------------------------------------------------------------------LEFRFLLQP-------------------------------------DPTVI------------------------------------------------------------NNLHN------------------------------------------------------------------SWK
Q664_08520_Cvio_667804119            SPVSWSRAY------FERIRPTFLECWAEE--------------------------------------------------------------------------------------LRALAVSHVHL---------------------PLTPA-------EAR---------------------------------AL----------------------------------------------------------------------------------------------------------------------------------------------------------------------SVTPPLWRERLVASDPEGLH--------------------SLAARLQKALEG---------------VEQ-----------GVFVRLGSGSPKDS---------------------------------------------------------------ALFR--EQ-G----------------------------------GCARTPMMAL------------------KFLQ-T---SPR-TRAHL----------------------SR-----FL----------------ELGHP--------------------------VHLFVRHWV--------------------------RIPPWQ---EFRC-------FM----------------RNRR-----------------LVGISQLAHRGDT------PEYSL--APR----------------------------------------------------AEELGRTLQDFFVG----------------------------------------------------VARASH--------------------------------------VGSAVFDVWCDTGAGD------------------------------------GAP-------------------------ARVWLLDANPWGP----------------ASDAC---------LF-DWSQP-----------------------------------------------------------------------------------------------------EGFDGSF--------------------------------------------------------------------------------------------------------------------------------------------------------------------------------------------------------------------------------------------------------RYL
_Mmar_519004236                      PPYDFSAAK---TTFIDAWPKA----------------------------------------------------------------------------------------------WRDLAPGYDIV---------------------PVDRI-------EMN---------------------------------AL---GAQIMGFRH-------------------------------------------------------------WFTP-----------------------------------------------------------------------------------------ASTQPLI---------------------------------HLAHRLDAVIAK---------------QNR-----------ACFIRLSSRSPKDS---------------------------------------------------------------IYALR-NG------------------------------------LCIRDGAQAL------------------AIIL-EG--SER-CAADL----------------------RM-----AL----------------DYHHP--------------------------MAIIVRNWI--------------------------DFPPWA---EFRC-------FM----------------VGRC-----------------WVGASQARHLERI------AYPPI--ADY----------------------------------------------------KSKILEVLNAS-MKK---------------------------------------------------IAAASP--------------------------------------IDNAAFDLVF------------------------------------------DSLQKS----------------------NHAILLDANPLLV----------------STDTA---------LF-SSIAD-----------------------------------------------------------------------------------------------------LDSTFRF------------------------------------------------------------------------------------------------------------------------------------------------------------------------------------------------------------------------------------------------------RNSKD
RLEG3_07720_Rleg_573470149           ---EPWADDVI-ASSFENWPKELR--------------------------------------------------------------------------------------------VHALAEPLDAI---------------------PISAG-------DMR---------------------------------AV----------------------------------------------------------------------------------------------------------------------------------------------------------------------LSQ-------------------------------------NAQYRRFLNISQPVSLPSRGFGKK---MEG-----------DAFPKIGPVSWKEI---------------------------------------------------------------SAFI--SV-P----------------------------------LAAIDELMPV------------------MLRG-V---TDR-MAFIL----------------------HA-----FV----------------CRQVS--------------------------TKLHVFPFV--------------------------DLSKAF---EVRF-------HI----------------EDGE-----------------PVHAKWMNRSD--------RYVPP--PGS----------------------------------------------------GEKLSN-----FAAN---------------------------------------------------IAERVG--------------------------------------IGYALLDLLLIK----------------------------------------GADG------------------------EAIKVVEVNPILE----------------RSASG---------R---------------------------------------------------------------------------------------------------------------------------------------------------------------------------------------------------------------------------------------------------------------------------------------------------------------------------------------------------------------------------LFL
crov369_CrVB_310831359               KKECNTNNH--------NEYFPVEILDENKIKIDKKW-------------------------------------------------------------------------------FNKLTYKTKKRLHAFISKYELDNLKFPHNNYKSLNES-------QLKLWYNIYHC-------------------------YLQQYD---------------------------------------------------------------------FTEYADFYN--------------------------------------------KDYVKIDISKNNLAILHKVCKVVISTNTSTSWIN-----TQFEDLSE---------------------------------EFILQIINGLKK---------------YDN-----------YCFIKTNKTSGKNE---------------------------------------------------------------ISLT--PK---------------------------------------STYLDVL------------------NHLT-N---CKE-YYIHF----------------------NR-----ALDKY-------------KLGDT--------------------------ENLIISKWRD-------------------------DFDKYR---EFRV-------II----------------LDGK-----------------IKGISQQ--L---------WYEKI--IYS----------------------------------------------------EEEILK-----ISNA---------------------------------------------------IIEFFNQNKFY---------------------------------VPDMTVDINV------------------------------------------NIK-------------------------YHVDLIECNPGGLYS--------------SSGSS---------LF-HWIND-----------------------------------------------------------------------------------------------------YDKLYDN--------------------------------------------------------------------------------------------------------------------------------------------------------------------------------------------------------------------------------------------------------TDI
_Ecal_515585312                      LPSVLLNQF-----------------------------------------------------------------------------------------------------------YAK---PIEVL---------------------PVSHD-------QLL---------------------------------AV----------------------------------------------------------------------------------------------------------------------------------------------------------------------LSN-------------------------------------NAHYRERFGLTQLPVHFTALPRA----FSQ-----------SLFPKLGVVSWKDV---------------------------------------------------------------VGMQTIPDAL----------------------------------LNTADYSPVL------------------ECWL-NAI-SDR-MALTL----------------------HA-----YR----------------CSSNT--------------------------PKLYLFPNQ--------------------------DFRERS---EYRL-------SV----------------SHGEIQG--------------VNCYCSR-------------RDYH--EEY----------------------------------------------------LEEIKTWWLSL--EP---------------------------------------------------FETSPN--------------------------------------LTHIFVDIAWC-----------------------------------------KDR-------------------------RAYVIIDVNPNLY----------------LLDQE---------V---------------------------------------------------------------------------------------------------------------------------------------------------------------------------------------------------------------------------------------------------------------------------------------------------------------------------------------------------------------------------ERR
_Ecal_515579254                      LPSVLLNQF-----------------------------------------------------------------------------------------------------------YAK---PIEVL---------------------PVSHD-------QLL---------------------------------AV----------------------------------------------------------------------------------------------------------------------------------------------------------------------LSN-------------------------------------NAHYRERFGLTQLPVHFPALPRA----FSQ-----------SLFPKLGVVSWKDV---------------------------------------------------------------VGMQTIPDAL----------------------------------LNTADYSPVLEC----------------WLNA-I---SDR-MALTL----------------------HA-----YR----------------CSSNT--------------------------PKLYLFPNQ--------------------------DFRERS---EYRL-------SV----------------SHGE-----------------IQGV-----N---------CYCSR--RDY----------------------------------------------------HEEYLEEIKTWWSSLEP-------------------------------------------------FETSPN--------------------------------------LTHIFVDIAWC-----------------------------------------KAR-------------------------GAYVIIDVNPNLY----------------LLDQE---------V---------------------------------------------------------------------------------------------------------------------------------------------------------------------------------------------------------------------------------------------------------------------------------------------------------------------------------------------------------------------------ERR
_Ecal_515582425                      -PSVLLNQF-----------------------------------------------------------------------------------------------------------YAK---PIEVL---------------------PVSHD-------QLL---------------------------------AV----------------------------------------------------------------------------------------------------------------------------------------------------------------------LSN-------------------------------------NAHYRERFGLTQ---------------LPVHFPALPRAFSQSLFPKLGVVSWKDVVGMQTIPDA------------------------------------------------------LLNT--AD---------------------------------------YSPVLEC------------------WLNA-I---SDR-M---------------------------------ALTLHAYR----------CSSNT--------------------------PKLYLFPNQ--------------------------DFRERS---EYRL-------SV----------------SHGE-----------------IQGV-----N---------CYCSR--RDY----------------------------------------------------HEEYLEEIKTWWLSL---------------------------------------------------EPFETSPN------------------------------------LTHIFVDIAW------------------------------------------CKAR------------------------RAYVIIDVNPNLY----------------LLDQE---------V---------------------------------------------------------------------------------------------------------------------------------------------------------------------------------------------------------------------------------------------------------------------------------------------------------------------------------------------------------------------------ERR
_Ecal_515585173                      PSSIVSYYL---------------------------------------------------------------------------------------------------------------TPPAVAI---------------------ALNKQ-------QLT---------------------------------AV----------------------------------------------------------------------------------------------------------------------------------------------------------------------LSR-------------------------------------NLRYRRQYGLSP---------------RNVPLSTQPSIQQQDYLPKLGVVSWKDCIG-------------------------------------------------------------MDML--PKALLL--------------------------------PSAQNTTLTC------------------WLNN-V---SDR-MAMVL----------------------HA-----YR----------------VTEET--------------------------PTFYLFPYL--------------------------DFSKRS---EYRL-------AV----------------SYGE-----------------LT-----HVR---------CYRRR--NDF----------------------------------------------------QAQHIEVIAAW-WRN---------------------------------------------------IKDWPPTDV-----------------------------------LAHLFVDVVA------------------------------------------GSDP------------------------GQFFIIDVNPNLS----------------------------------------------------------------------------------------------------------------------------------------------------------------------------------------------------------------------------------------------------------------------------------------------------------------------------------------------------------------------------------------------------------AYH
cdc123_Psp._374333538                YPKAVSDTF------FASWPDA----------------------------------------------------------------------------------------------LKQASVPHEEI---------------------PISPE-------DVL---------------------------------ALAANT-----------------------------------------------------------------------------------------------------------------------------------------------------------------PEFCEQFGIFNHYGLSE------------------------EFRDTISKGLEK---------------FKD-----------GAFPRLDYCSWKTSC--------------------------------------------------------------LLNA--PA---------------------------------------KSLGEVEA-----------------IVLQ-P---NQR-VASAL----------------------MD-----PV----------------INNTG--------------------------ANFYLRKWV--------------------------DIPRWS---EFRI-------FM----------------RDRK-----------------IIGVSQYYTDE--------QFPAL--QEN----------------------------------------------------LDKIREALIEFCLF----------------------------------------------------FYKESH--------------------------------------LDTVVADVFLANQ---------------------------------------NEK-------------------------LQAQLIELNPFLN----------------RTDPC---------LY-NWE----------------------------------------------------------------------------------------------------------------------------------------------------------------------------------------------------------------------------------------------------------------------------------------------------------------------------------------------------------------------KSN
_Psp._504056239                      YPKAVSDTF------FASWPDA----------------------------------------------------------------------------------------------LKQASVPHEEI---------------------PISPE-------DVL---------------------------------ALAANT-----------------------------------------------------------------------------------------------------------------------------------------------------------------PEFCEQFGIFNHYGLSE------------------------EFRDTISKGLEK---------------FKD-----------GAFPRLDYCSWKTSC--------------------------------------------------------------LLNA--PA---------------------------------------KSLGEVEA-----------------IVLQ-P---NQR-VASAL----------------------MD-----PV----------------INNTG--------------------------ANFYLRKWV--------------------------DIPRWS---EFRI-------FM----------------RDRK-----------------IIGVSQYYTDE--------QFPAL--QEN----------------------------------------------------LDKIREALIEF-CLF---------------------------------------------------FYKESH--------------------------------------LDTVVADVFLANQ---------------------------------------NEK-------------------------LQAQLIELNPFLN----------------RTDPC---------LY-NWE----------------------------------------------------------------------------------------------------------------------------------------------------------------------------------------------------------------------------------------------------------------------------------------------------------------------------------------------------------------------KSN
_Sste_492493562                      -------TF------PENWPPT----------------------------------------------------------------------------------------------LTLASLPAVSV---------------------TLSDG-------DAETLGSQS---------------------------ALYREV------------------------------------------------------------------------------------------------------------------------------------------------------------------AGDARRIGFGP-----------------------------DFEKTIAKALET---------------FPE-----------GLMPRIGMCSWKAS---------------------------------------------------------------TVVH--AP--------------------------------------CHSVADVM------------------RVIT-AN--DPR-VAQAI----------------------LD-----HR----------------ISKRP--------------------------VVLHLRAWR--------------------------DIPDWA---EFRL-------FV----------------KRRG-----------------LLGVSQYAWQE--------TFPQI--AAQ----------------------------------------------------HSAIVTAVNAL-LKD---------------------------------------------------IWEDLH--------------------------------------MDDVVIDVCV------------------------------------------LPEGDG----------------------LKAWLIELNPLDP----------------RSDAC---------LY-SW---------------------------------------------------------------------------------------------------------------------------------------------------------------------------------------ENGGDFDGSFR-----------------------------YNR--------------------------------------------------------------PYRAE------------------------------------------------------------------AFG
_Ecal_515579285                      PSSIVSYYL---------------------------------------------------------------------------------------------------------------TPPAVAI---------------------ALNKQ-------QLT---------------------------------AV----------------------------------------------------------------------------------------------------------------------------------------------------------------------LSR-------------------------------------NLRYRRQYGLSP---------------RNVSLSTQPSIQQQDYLPKLGVVSWKDCIG-------------------------------------------------------------MDML--PK-A----------------------------------LLLPNAQNTTLTC---------------WLNN-V---SDR-MAMVL----------------------HA-----YR----------------VTEET--------------------------PTFYLFPYL--------------------------DFSKRS---EYRL-------AV----------------SYGE-----------------LT--HVR------------CYRRR--NDF----------------------------------------------------QTKHTDAIAAW-WRN---------------------------------------------------IKDWPPTDV-----------------------------------LAHLFVDVVA------------------------------------------GSDP------------------------GQFCIIDVNPNL-----------------------------------------------------------------------------------------------------------------------------------------------------------------------------------------------------------------------------------------------------------------------------------------------------------------------------------------------------------------------------------------------------------SAY
_Arhi_518854330                      LHEKWRATF------IESWPDEVCELGLP---------------------------------------------------------------------------------------FEQ-------L---------------------LISEQ-------DRA---------------------------------AIGSRT---------------------------------------------------------------------AAFREL-----------------------------------------------FDID------------------------------------ELT-------------------------------------QLSDEFRIGIDAKTALF----------KDG-----------AHF-RLGGCSFKQ----------------------------------------------------------------------PGRYQDG-------------------------------PIFNSAQLMP------------------HVLR-D---NPR-VAGLL----------------------AS-----SL----------------QDKFD--------------------------VCMFIRPWE--------------------------NIPKWS---EFRL-------FM----------------KNRE-----------------FIGASQYFHTA--------FFPEI--EAK----------------------------------------------------ARSIAVALVEF-ADR---------------------------------------------------FRQVAH--------------------------------------IDDAIVDIYL------------------------------------------RPDDAGG---------------------FEGVLLDLNPLIL----------------RSDPC---------LF-QWKNG-----------------------------------------------------------------------------------------------------GDFDRGLR-----------------------------------------------------------------FRGRDNRVL-------------------------------------AIT--------------------------------------------------------------PLPFA------------------------------------------------------------------YAA
_Atum_489600129                      LHEKWRATF------IESWPDEVCELGLP---------------------------------------------------------------------------------------FEQ-------L---------------------LISEQ-------DRA---------------------------------AIGSRT---------------------------------------------------------------------AAFREL-----------------------------------------------FDID------------------------------------ELT-------------------------------------QLSDEFRIGIDAKTAL-----------FKD-----------GAHFRLGGCSFKQ----------------------------------------------------------------------PGRYQDG-------------------------------PIFNSAQLLP------------------HVLR-D---NPR-VAGLL----------------------AS-----SL----------------QDKFD--------------------------VCMFIRPWE--------------------------NIPKWS---EFRL-------FM----------------KNRE-----------------FIGASQYFHTA--------FFPEI--EAK----------------------------------------------------ARSIAVALVEF-ADR---------------------------------------------------FRQVAH--------------------------------------IDDAIVDIYLR-----------------------------------------PDDAAG----------------------FEAVLLDLNPLIL----------------RSDPC---------LF-QWKNG-----------------------------------------------------------------------------------------------------GDFDRGLR-----------------------------------------------------------------FRGRDNRVL-------------------------------------AIT--------------------------------------------------------------PLPFA------------------------------------------------------------------YAA
ACD_46C00111G0002_Ubac_406938528     KKELHFDFD------FDMWYDK----------------------------------------------------------------------------------------------LKDYSFKSDVV---------------------PITPE-------IAQAMV------------------------------NYYSNR---------------------------------------------------------------------FYKR-----------------------------------------------------------------------------------------NVLTKEDV--------------------------------KLLEKLRRNIQL---------------HLNKSKSTH-----GFFVRMSNRSPKDGTPLKNKSMVDIYKEIYSNPNDDWNNKMIKICD-------------------------------AQ-----MKM----------------------------------LCCQNADEVM------------------NLLL-S---SER-IYMDLIEALDCH---------------LY-----SK----------------SDLWK--------------------------TSVILREWIP-------------------------DLKQDF---EFRI-------FV----------------SNNH-----------------VTATSQYNHYC--------CFESL--MILNQ--------------------------------------------------HNELMKLNQRLIDYAMK-------------------------------------------------IHPLIN--------------------------------------KSQYVLDVAL------------------------------------------INN-------------------------E-LYVIELNPFDK----------------STGPC---------LF-SWEKD-----------------------------------------------------------------------------------------------------SELLTGNGSMQI-------------------------------------------------------------SELRINQAP-------------------------------------RNNVNEIIQHIIETETDLAKDSIEPYF--------------------------------------EYSEY------------------------------------------------------------------KKH
BBM_III06255_Bmic_399218561          PEWIYNINY-N-YWTHQHWANC----------------------------------------------------------------------------------------------CKSSTFKYHKV---------------------ELPEC-------FVK---------------------------------YLNSNT---------------------------------------------------------------------FYVPDNLACGKYIVKS-------------------------------------DSESELHYSDN----------------------------ELSPNDSVDSVIS----------------------------LSDPELNHPII----------------AIS-----------GVLRKHAGCMVKYRNIFVLDG--------------------------------------------------------CWII--NN-S----------------------------------YTVTNERDVI------------------LLLK-S---SCK-WRDCS-------------------------------------------------YQE--------------------------GELYLVERR--------------------------KIDPSN---EFRL-------YY----------------IDNS-----------------LVAISQLHSHIKL------PYLQN--PNN----------------------------------------------------RIEVVTNIIQLNNEIQN-------------------------------------------------IIRELR--------------------------------------LQYCAVDVAL------------------------------------------GSD-------------------------E--FIIDILPWGWNGP-------------LAHEP---------LL-DWRDLRL---------------------------------------------------------------------------------------------------LYIANYK------------------------------------------------------------------FELRQLFRCGVEAIKNVYFLY-------------------------IES--------------------------------------------------------------DNQVR------------------------------------------------------------------NGP
EMIHUDRAFT_461318_Ehux_551569108     AAPEDGGDVCN-SCNLHEWAPFRADCDGVRVDPSW---------------------------------------------------------------------------------WASLAPEHRALMSGAVVV--------------TVSEA-------PIDVPHAAEIRTHAGCGA------------------LYNAYNAGFAFHHWRHMQPAAHL----------------------------------------------------VALAPS-----------------------------------------------DRDVLESVAIRHAVYNGRKLNEEDL--------------ESLP-------------------------------------ALISALTEAIRA---------------VGG-----------QAFVKTAEKSAKND---------------------------------------------------------------VPLR--PH---------------------------------------ATAQSAL------------------TELT-S---SED-VLRQS-------------------------------------------------LSGGAAGRA--------------------RYLVVQPWEH-------------------------GISAHN---EWRL-------IV----------------CGGR-----------------VAGISQQTWRR--------AAGHT--EASA---------------------------------------------------RAAVPSLIRLW--QE---------------------------------------------------LAPLSP--------------------------------------YADCVVDAHV------------------------------------------DSSS------------------------GRAKLIEVNACGWWG--------------PSGSA---------LF-HYERD-----------------------------------------------------------------------------------------------------HELLRDPDRL---------------------------------------------------------------PVRVVVETADEH----------------------------------TVP--------------------------------------------------------------LPNVP------------------------------------------------------------------WRR
EMIHUDRAFT_247309_Ehux_551556040     -------CN------LHEWAPFRADCDGVRVDPSW---------------------------------------------------------------------------------WASLAPEHRALMSGAVVV--------------TVSEA-------PIDVPHAAEIRTHAGCGALYNAYNAGFAF-------HHWRHMQPAAHL---------------------------------------------------------------VALAPS-----------------------------------------------DRDVLESVAIRHAVYNGRKLNEEDL--------------EGLP-------------------------------------ALISALTEVIRA---------------VGG-----------QAFVKTAEKSAKND---------------------------------------------------------------VPLR--PHATAQSAL-----------------------------TELTSSEDVL------------------RQSL-S-----------------------------------------GG----------------AAGRA--------------------------RYLVVQPWEH-------------------------GISAHN---EWRL-------IV----------------CGGR-----------------VAGISQQTWRRA-------AGHTE--ASA----------------------------------------------------RAAVPSLIRLW--QE---------------------------------------------------LAPLSP--------------------------------------YADCVVDAHV------------------------------------------DSSS------------------------GRAKLIEVNACGWWG--------------PSGSA---------LF-HYERD-----------------------------------------------------------------------------------------------------HELLRDPDRL---------------------------------------------------------------PVRVVVETADEHT---------------------------------VPL--------------------------------------------------------------PTGTD------------------------------------------------------------------VGG
NAEGRDRAFT_74505_Ngru_290975738      SPSSHKKFY---DFRVEDWYDCCFD-------------------------------------------------------------------------------------------GNPLTFVTKFM---------------------PITIE-------QANAVVHLYEIFHKYPRQLIPELSNRTDLDEEKVYYYLKGAKVKLMKRFANCEPSNIVEN---------------------------------------------------FYLFES-----------------------------------------------VSNEGSEVDLE----------------------------NYEPNLRKQKIFTQKDA------------------------TLIQQLALNLHETFESMPSDCEY----RNN-----------GYFVRASTRSPKDG---------------------------------------------------------------CLKH--RESFMEHLKQELVKNSQDSNRELTYNEPLAVVRTLNAKLCVQNAMQAM------------------NLVI-H---SER-MYADLFR--------------------ALLFIDEKL----------------KKTPE--------------------------CELPGQKFVARLFKK--------------------MKRPEY---EFRV-------FV----------------KYFEKEMKHQ-----------VTGITQYFRT---------CYLED--IQHGTL-------------------------------------------------KEQIQKGIEELAQEV---------------------------------------------------SKRLST--------------------------------------TENFVLDVSVEYDK--------------------------------------NETGDCTV--------------------SKIWMIEINSFSK----------------QASPC---------MF-DWNDPVS---------------------------------------------------------------------------------------------------NEILMGNRP----------------------------------------------------------------MEFRVLTKPLTLKEGREELAKDIREML-------------------EEI--------------------------------------------------------------NGAVP-----------------------------------------------------PEAEESNDEDKKKNCS
_Lsai_653018179                      PTSLTRWEAVD-KFSVENWYPH----------------------------------------------------------------------------------------------LKKYTFESCFF---------------------TLDHA-------DIQ---------------------------------FLLGNG---------------------------------------------------------------------SSEY-----------------------------------------------------------------------------------------QST-------------------------------------KLEQKFDKLLSQ---------------FNNQ----------EVFMRLSTRSPKDSRYLFDEAATMMSHDFS-----------------------------------------------YWKD--TDNKNQQLVSFVASMAKS--------------------MKINNGKKII------------------QMIQ-E---SPR-VQNDL----------------------FA-----LLNSES------------PSDCK--------------------------TNIVLREWH--------------------------NIRPDH---EFRL-------FV----------------SRRCREESI------------VTAVSQYFHFLYFDKAPGDCFNFL--DESI---------------------------------------------------KKSLILKFQNYVLKS---------------------------------------------------IDPAVAKFLNFSKEQDDDNSINC---------------------IREYIVDLALVPISEYHGEVT-------------------------------DENKIELGENT-----------------YILMVIELNPFAPS---------------ATGCG---------LF-NWEKN-----------------------------------------------------------------------------------------------------LDILWGKAPCEY-------------------------------------------------------------PIYSFRTKP-------------------------------------REN--------------------------------------------------------------LSSVTLLPSNYEQ-----------------VIER---------------------ALDKRLAASLPSLDPQVSP
pv_193_PVs_585299522                 TPEELLRRKIS-FTHAENWYPDLEEISSD---------------------------------------------------------------------------------------GRILTWESVLI---------------------PFD---------------------------------------------------------------------------------------------------------------------------------------------------------------------------------------------------------------------NSPF------------------------------------EIVEKIDEVIKK---------------LGG-----------SCFIRLNSLSPKH-----------------------------------------------------------------------F------------------------------------EPVTSGYEAA------------------TILY-E---SER-T---------------------------------RQT---------------FGLFR--------------------------NLVMVRKFE--------------------------RFPKEM---EFRL-------FV----------------RKGK-----------------LRAISRYDPY---------CLAPL--KNS----------------------------------------------------AEELQRIFQRFFRCLQ--------------------------------------------------VESLVL--------------------------------------FDLCTIDCVY------------------------------------------WPEQINRSYFL-----------------DGVFLIEFNTFGPDS--------------ISGSC---------LF-DWEAD-----------------------------------------------------------------------------------------------------KQIL-----------------------------------------------------------------------------------------------------------------------------------------------------------------------------------------------------------------------------------------------------------YHG
MAR_ORF400_MVMV_284504410            KKVKFTNAK--------NWYPEAEHGVDG---------------------------------------------------------------------------------------KRFLTFPSVLV---------------------PLSEK--------------------------------------------------------------------------------------------------------------------------------------------------------------------------------------------------------------------------------------------------------------RKLGEAIER---------------LGG-----------SVFVRLGSLSPKF-----------------------------------------------------------------------F------------------------------------EPVETPEQVL------------------QVLQ-E---SER-T---------------------------------RDC---------------LKDGE--------------------------EVFFLRRYE--------------------------DIPKNK---EFRL-------FV----------------CKGK-----------------LRAVSKYDPEAD-------CFMAS--EEV----------------------------------------------------RDIISR-----WFRNIC-------------------------------------------------LDGLLS--------------------------------------FENCCLDVVV------------------------------------------WEERKEESLYD-----------------DGVFLIEYNSFGEDS--------------VSGSC---------LF-HWEED-----------------------------------------------------------------------------------------------------WETL-----------------------------------------------------------------------------------------------------------------------------------------------------------------------------------------------------------------------------------------------------------TKG
C8_423_C8V_539398937                 KKVKFTNAK--------NWYPEAEHGVDG---------------------------------------------------------------------------------------KRFLTFPSVLV---------------------PLS--------------------------------------------------------------------------------------------------------------------------------------------------------------------------------------------------------------------------------------------------------------EKRKLGEAIER---------------LGG-----------SVFVRLGSLSPKF------------------------------------------------------------------FE--PV---------------------------------------ETPEQVL------------------QVLQ-E---SER-T---------------------------------RDC---------------LKDGE--------------------------EVFFLRRYE--------------------------DIPKNK---EFRL-------FV----------------CKGK-----------------LRAVSKYDPEAD-------CFMAS--EDV----------------------------------------------------RDIISRWFRNI---C---------------------------------------------------LDGLLS--------------------------------------FENCCLDVVVWEERKEES----------------------------------LYD-------------------------DGVFLIEYNSFGEDS--------------VSGSC---------LF-HWEED-----------------------------------------------------------------------------------------------------WETL-----------------------------------------------------------------------------------------------------------------------------------------------------------------------------------------------------------------------------------------------------------TKG
LAU_0405_LV_327409947                REKKISFTH------AENWYPHAEEGRDG---------------------------------------------------------------------------------------ERFLTFESVLL---------------------PFS--------------------------------------------------------------------------------------------------------------------------------------------------------------------------------------------------------------------------------------------------------------NFEKVDDAIQK---------------LGG-----------SVFVRLSSLSPKF------------------------------------------------------------------FE--PV---------------------------------------QTKEEVL------------------SVLT-E---SER-----------------------------------TR----------------DNLEN--------------------------SVLFLRKYF--------------------------DFPKNK---EFRL-------FV----------------RKGK-----------------LRAISKYDPE---------ADCGL--PKE------------------------------------------------------GVQQKVSKWFRCLC--------------------------------------------------LEGLLS--------------------------------------FEDCTLDIVL------------------------------------------WEERRELSLFD-----------------DGIFLIEYNSYGEDS--------------ISGSC---------LF-DWEKD-----------------------------------------------------------------------------------------------------WEILTKG--------------------------------------------------------------------------------------------------------------------------------------------------------------------------------------------------------------------------------------------------------KAV
TNS_ORF328_TVf_566082636             TVEELFHKKIK-HTHAENWYPHASEGNED---------------------------------------------------------------------------------------GKILTFESVLL---------------------PFS--------------------------------------------------------------------------------------------------------------------------------------------------------------------------------------------------------------------------------------------------------------DFDKVDDAIEK---------------LGG-----------SVFVRLSSLSPKF-----------------------------------------------------------------------F------------------------------------EPVQTKEQVL------------------SVLQ-S---SER-----------------------------------TR----------------DELKG--------------------------STLFLRKYY--------------------------DFPKDK---EFRL-------FV----------------RRGK-----------------LRTISRYDPE---------ADCHL--SPEFI--------------------------------------------------RDKIAK-----WFRCLC-------------------------------------------------LEGLLS--------------------------------------FEDCTLDVVLWEE---------------------------------------KKEMSLFD--------------------DGIFLIEYNSYGEDS--------------VSGSC---------LF-DWEAD-----------------------------------------------------------------------------------------------------WEIL-----------------------------------------------------------------------------------------------------------------------------------------------------------------------------------------------------------------------------------------------------------TKG
ISTM_472_IV_557952576                FHKKIKHTH------AENWYPHASEGNED---------------------------------------------------------------------------------------GKILTFESVLM---------------------PFSD--------------------------------------------------------------------------------------------------------------------------------------------------------------------------------------------------------------------------------------------------------------FDKVDDAIEK---------------LGG-----------SVFVRLSSLSPKFF---------------------------------------------------------------EP-----V---------------------------------------QTKEQVL------------------SVLQ-S---SER-----------------------------------TR----------------DELKG--------------------------STLFLRKYY--------------------------DFPKDK---EFRL-------FV----------------RRGK-----------------LRAISRYDPEAD-------CHLSP--EFI----------------------------------------------------RDKIAK-----WFRCLC-------------------------------------------------LEGLLS--------------------------------------FEDCTLDVVL------------------------------------------WEEKKEMSLFD-----------------DGIFLIEYNSYGEDS--------------VSGSC---------LF-DWEADW----------------------------------------------------------------------------------------------------EILT-----------------------------------------------------------------------------------------------------------------------------------------------------------------------------------------------------------------------------------------------------------KGK
ISTM_474_IV_557952578                TVEELLHKKIK-HTHAENWYPHASEGNED---------------------------------------------------------------------------------------GKILTFESVLL---------------------PFS--------------------------------------------------------------------------------------------------------------------------------------------------------------------------------------------------------------------------------------------------------------DFDKVDDAIEK---------------LGG-----------SVFVRLSSLSPKF-----------------------------------------------------------------------F------------------------------------EPVQTKEQVL------------------SVLQ-S---SER-----------------------------------TR----------------DELEG--------------------------STLFLRKYY--------------------------DFPKDK---EFRL-------FV----------------RKGK-----------------LRAISRYDPE---------AECQL--SPEFV--------------------------------------------------RDKLTR-----WFRCLC-------------------------------------------------LEGLLS--------------------------------------FEDCTLDVVL------------------------------------------WEEKKEMSLFD-----------------DGIFLIEYNSYGEDS--------------VSGSC---------LF-DWEAD-----------------------------------------------------------------------------------------------------WEILT----------------------------------------------------------------------------------------------------------------------------------------------------------------------------------------------------------------------------------------------------------KGK
_Llon_489732119                      PTSLTRWEAVD-KFSVENWYPH----------------------------------------------------------------------------------------------LKKYTFESCFF---------------------TLDHA-------DIE---------------------------------FLLGIG---------------------------------------------------------------------SSEY-----------------------------------------------------------------------------------------QST-------------------------------------NLEQKFDKLLSQ---------------FNNQ----------EVFMRLSTRSPKDSRHLFDEAATLMSNDYF-----------------------------------------------YWKD--TDNKNQQLVSFVASMAKS--------------------MKITNGKKII------------------QMIQ-E---SPR-VQNDL----------------------FA-----LLSSES------------PSDCK--------------------------TNIVLREWH--------------------------NIRPDH---EFRL-------FV----------------SRRCREESI------------VTAISQYFHFLHFDKAPGDCFNFL--DESI---------------------------------------------------KKSLILKFQNYVLKS---------------------------------------------------IDPAVARFLNFSSEQDDDNSINC---------------------IREYIVDLALVPISEYHGEVT-------------------------------DENKIEIGENT-----------------YILTVIELNPFAPS---------------ATGCG---------LF-NWQKD-----------------------------------------------------------------------------------------------------LDILWGKAPCEY-------------------------------------------------------------PIYRFRTEP-------------------------------------REN--------------------------------------------------------------FSSVTLLPSNYEQ-----------------VIDR---------------------ALVKRLVTSLPSVDSQASP
LLO_2267_Llon_289165596              PTSLTRWEAVD-KFSVENWYPH----------------------------------------------------------------------------------------------LKKYTFESCFF---------------------TLDHA-------DIE---------------------------------FLLGIG---------------------------------------------------------------------SSEY-----------------------------------------------------------------------------------------QST-------------------------------------NLEQKFDKLLSQ---------------FNNQ----------EVFMRLSTRSPKDSRHLFDEAATLMSNDYF-----------------------------------------------YWKD--TDNKNQQLVSFVASMAKS--------------------MKITNGKKII------------------QMIQ-E---SPR-VQNDL----------------------FA-----LLSSES------------PSDCK--------------------------TNIVLREWH--------------------------NIRPDH---EFRL-------FV----------------SRRCREESI------------VTAISQYFHFLHFDKAPGDCFNFL--DESI---------------------------------------------------KKSLILKFQNYVLKS---------------------------------------------------IDPAVARFLNFSSEQDDDNSINC---------------------IREYIVDLALVPISEYHGEVT-------------------------------DENKIEIGENT-----------------YILTVIELNPFAPS---------------ATGCG---------LF-NWQKD-----------------------------------------------------------------------------------------------------LDILWGKAPCEY-------------------------------------------------------------PIYRFRTEP-------------------------------------REN--------------------------------------------------------------FSSVTLLPSNYEQ-----------------VIDR---------------------ALVKRLVTSLPSVDSQASP
_Lwad_671590708                      -TSLTRWEAVD-KFSVENWYPH----------------------------------------------------------------------------------------------LEKYTFVTTFI---------------------KLDYD-------DIQ---------------------------------FLMGNG---------------------------------------------------------------------SPEY-----------------------------------------------------------------------------------------DST-------------------------------------LLEHSFDHFLSQ---------------LRNQ----------EAFMRLSSRSPKDSKALFDEAATIMSKDFS-----------------------------------------------NWNE--FDNKNQQLVAFVASMTKA--------------------MKITSGRKII------------------ETIQ-Q---SPR-VHNDL----------------------IA-----LMSTAS------------RSNCT--------------------------TNIVLREWY--------------------------SIRPDH---EFRA-------FV----------------SRRCRKESI------------VTAIAQYFHFLYFDKAPTDCFNFL--EEDT---------------------------------------------------KKNLILKFQNYILKS---------------------------------------------------IDPAVANFLNFASEQDDDNSIDC---------------------IREYIVDIALIPINQYHGEMT-------------------------------DDNTIHIGGST-----------------YIIMVIELNPFAPS---------------ATGCA---------LF-NWKTD-----------------------------------------------------------------------------------------------------LDMLWGKASCDY-------------------------------------------------------------PVFSYRTQP-------------------------------------RED--------------------------------------------------------------LRSVTLLPSNHQE-----------------VIQK---------------------AREKRISHSTAAVITPVSP
_Lpne_504656984                      PKGSTRWDAVN-KFSVENWYSH----------------------------------------------------------------------------------------------LKKHTFKSHFV---------------------TLNYN-------DIQ---------------------------------YLMGNI---------------------------------------------------------------------PPDY-----------------------------------------------------------------------------------------DST-------------------------------------KLESIFNKILSE---------------FNNK----------EVFMRLSTRSPKDSKFLFEEASTIMSKDFV-----------------------------------------------YWNE--NDNKHQQLVSFVASMLKA--------------------MKIKSGRKII------------------ETIA-Q---SPR-VYNDL----------------------LA-----LVSSSD------------KLDCT--------------------------TNVILREWH--------------------------DIRPDH---EFRV-------FV----------------SRRHRKESI------------VTAISQYFHFLYFDKSPTDCFNFL--DEED---------------------------------------------------KKAVIKKFQNYVLKS---------------------------------------------------VDPDVAKFLNFSSEQNDDESSDC---------------------IREYIVDLALIPVSQYHGEIT-------------------------------DENIIQIGTNT-----------------YVMVVIELNPFAPA---------------ATGSG---------LF-NWKND-----------------------------------------------------------------------------------------------------LMMLWGKASCDY-------------------------------------------------------------PVFKYRTTP-------------------------------------RED--------------------------------------------------------------FQSVSLLPSNYES-----------------VIKS---------------------ALIKRLETSYSISASQKER
LPV_1947_Lpne_397667414              PKGSTRWDAVN-KFSVENWYSH----------------------------------------------------------------------------------------------LKKHTFKSHFV---------------------TLNYN-------DIQ---------------------------------YLMGNI---------------------------------------------------------------------PPDY-----------------------------------------------------------------------------------------DST-------------------------------------KLESIFNKILSE---------------FNNK----------EVFMRLSTRSPKDSKFLFEEASTIMSKDFV-----------------------------------------------YWNE--NDNKHQQLVSFVASMLKA--------------------MKIKSGRKII------------------ETIA-Q---SPR-VYNDL----------------------LA-----LVSSSD------------KLDCT--------------------------TNVILREWH--------------------------DIRPDH---EFRV-------FV----------------SRRHRKESI------------VTAISQYFHFLYFDKSPTDCFNFL--DEED---------------------------------------------------KKAVIKKFQNYVLKS---------------------------------------------------VDPDVAKFLNFSSEQNDDESSDC---------------------IREYIVDLALIPVSQYHGEIT-------------------------------DENIIQIGTNT-----------------YVMVVIELNPFAPA---------------ATGSG---------LF-NWKND-----------------------------------------------------------------------------------------------------LMMLWGKASCDY-------------------------------------------------------------PVFKYRTTP-------------------------------------RED--------------------------------------------------------------FQSVSLLPSNYES-----------------VIKS---------------------ALIKRLETSYSISASQKER
ACA1_093020_Acas_470392564           -GEEQQKFDKL-DWAVEGWYEA----------------------------------------------------------------------------------------------IKEFTFETRFV---------------------ELPPA-------AVQ---------------------------------ALLRAH---------------------------------------------------------------------DARSAQ-----------------------------------------------R---------------------------------------TLE-------------------------------------PAHATVEEAIEA---------------LGG-----------AAFVRISTLSPKDAVKWQSEKLKGLLEAELEGAAAGDEDAEIIA---------------------------------INMA--CCLA----------------------------------CRVTNGAEAM------------------DLLI-R---SDR-VDRHLA---------------------TR-----RE----------------EEGDELS------------------------VNIVIRKWL--------------------------DFRPEL---EFRSEFSIG--FV----------------YDRQ-----------------LTAVTHYYKFC--------FVREA--VEK----------------------------------------------------KEAIAQQIRSFYEEK---------------------------------------------------LRDTIP--------------------------------------ASTYAIDFAL------------------------------------------LPD-------------------------GQLIVVELNPFAP----------------NTSPG---------LF-DWTKD-----------------------------------------------------------------------------------------------------EDVLKGVKP----------------------------------------------------------------FEFRLLENR-------------------------------------VENAREL----------------------------------------------------------LAAPLRFLLDLVR-----------------PREAA--------------------DEKKGSEEEPAARSRDKNE
_Lpne_499249871                      SKGSTRWDAVN-KFSVENWYPH----------------------------------------------------------------------------------------------LRKHTFKSHFI---------------------TLNYN-------DIH---------------------------------YLMGNI---------------------------------------------------------------------PPDY-----------------------------------------------------------------------------------------DSS-------------------------------------KLESIFNKILSE---------------FNNK----------EVFMRLSTRSPKDSKFLFEEASTIMSKDFV-----------------------------------------------YWNE--NDNKHQQLVSFVASMLKA--------------------MKIKSGRKII------------------ETIA-Q---SPR-VYNDL----------------------LA-----LVSSSN------------QSDCT--------------------------TNVILREWH--------------------------DIRPDH---EFRV-------FV----------------SRRHRKESI------------VTAISQYFHFLYFDKSPADCFNFL--DEED---------------------------------------------------KKAVIKKFENYVLKS---------------------------------------------------VDPDVAKFLNFSSEQDDDESSDC---------------------IREYIVDLALIPVSQYHGEIT-------------------------------DENIIEIGANT-----------------YVMVVIELNPFAPA---------------ATGSG---------LF-NWKND-----------------------------------------------------------------------------------------------------LMMLWGKASCDY-------------------------------------------------------------PVFKYRTTP-------------------------------------RED--------------------------------------------------------------LQSVSLLPSNYES-----------------VIKS---------------------ALIKRLETSYSNSASQKER
lpg1684_Lpne_52841912                SKGSTRWDAVN-KFSVENWYPH----------------------------------------------------------------------------------------------LRKHTFKSHFI---------------------TLNYN-------DIH---------------------------------YLMGNI---------------------------------------------------------------------PPDY-----------------------------------------------------------------------------------------DSS-------------------------------------KLESIFNKILSE---------------FNNK----------EVFMRLSTRSPKDSKFLFEEASTIMSKDFV-----------------------------------------------YWNE--NDNKHQQLVSFVASMLKA--------------------MKIKSGRKII------------------ETIA-Q---SPR-VYNDL----------------------LA-----LVSSSN------------QSDCT--------------------------TNVILREWH--------------------------DIRPDH---EFRV-------FV----------------SRRHRKESI------------VTAISQYFHFLYFDKSPADCFNFL--DEED---------------------------------------------------KKAVIKKFENYVLKS---------------------------------------------------VDPDVAKFLNFSSEQDDDESSDC---------------------IREYIVDLALIPVSQYHGEIT-------------------------------DENIIEIGANT-----------------YVMVVIELNPFAPA---------------ATGSG---------LF-NWKND-----------------------------------------------------------------------------------------------------LMMLWGKASCDY-------------------------------------------------------------PVFKYRTTP-------------------------------------RED--------------------------------------------------------------LQSVSLLPSNYES-----------------VIKS---------------------ALIKRLETSYSNSASQKER
_Lpne_652968979                      PKGSTRWDAVN-KFSVENWYPH----------------------------------------------------------------------------------------------LKKHTFKSRFI---------------------TLNYN-------DIQ---------------------------------YLMGNI---------------------------------------------------------------------LPDY-----------------------------------------------------------------------------------------DST-------------------------------------KLESMFNNIISE---------------FNNK----------EVFMRLSTRSPKDSKHLFEEAATIMSKDFV-----------------------------------------------YWSE--NDNKHQQLVSFVASMLKS--------------------MKIKNGKKII------------------ETIA-Q---SPR-VYNDL----------------------IA-----LVSSVD------------QSDCT--------------------------TNIILREWH--------------------------DIRPDH---EFRV-------FV----------------SRRHRKESI------------VTAISQYFHFLYFDKNPADCFNFL--DEED---------------------------------------------------KKAVIKKFENYVLKS---------------------------------------------------VDPDVAKFLNFSSEQDNDESADC---------------------IREYIVDLALIPIHQYHGEVT-------------------------------NENKIEIGKSI-----------------YVMVVIELNPFAPA---------------ATGSG---------LF-NWKND-----------------------------------------------------------------------------------------------------LMMLWGKTSCDY-------------------------------------------------------------PVFKYRTTP-------------------------------------RED--------------------------------------------------------------LQSVSLLPSNYES-----------------VIQS---------------------ALIKRLGNSYSSSVSQTAR
_Lpne_654927668                      PKGSTRWDAVN-KFSVENWYPH----------------------------------------------------------------------------------------------LKKHTFKSRFI---------------------TLNYN-------DIQ---------------------------------YLMGNI---------------------------------------------------------------------LPDY-----------------------------------------------------------------------------------------DST-------------------------------------KLESMFNNIISE---------------FNNK----------EVFMRLSTRSPKDSKHLFEEAATIMSKDFV-----------------------------------------------YWSE--NDNKHQQLVSFVASMLKS--------------------MKIKNGRKII------------------ETIA-Q---SPR-VYNDL----------------------IA-----LVSSVD------------QSDCT--------------------------TNIILREWH--------------------------DIRPDH---EFRV-------FV----------------SRRHRKESI------------VTAISQYFHFLYFDKNPADCFNFL--DEED---------------------------------------------------KKTVIKKFENYVLKS---------------------------------------------------VDPDVAKFLNFSSEQDNDESADC---------------------IREYIVDLALIPIHQYHGEVT-------------------------------DENKIAIGENI-----------------YVMVVIELNPFAPA---------------ATGCG---------LF-NWKND-----------------------------------------------------------------------------------------------------LMMLWGKTSCDY-------------------------------------------------------------PVFKYRTTP-------------------------------------RED--------------------------------------------------------------LQSVTLLPSNYES-----------------VIQS---------------------ALIKRLENSYSSSISQAAR
_Lpne_653015156                      PKGSTRWDAVN-KFSVENWYPH----------------------------------------------------------------------------------------------LKKHTFKSRFI---------------------TLNYN-------DIQ---------------------------------YLMGNI---------------------------------------------------------------------LPDY-----------------------------------------------------------------------------------------DST-------------------------------------KLESMFNNIISE---------------FNNK----------EVFMRLSTRSPKDSKHLFEEAATIMSKDFV-----------------------------------------------YWSE--NDNKHQQLVSFVASMLKS--------------------MKIKNGRKII------------------ETIA-Q---SPR-VYNDL----------------------IA-----LVSSVD------------QSDCT--------------------------TNIILREWY--------------------------DIRPDH---EFRV-------FV----------------SRRHRKESI------------VTAISQYFHFLYFDKNPTDCFNFL--DEED---------------------------------------------------KKTVIKKFENYVLKL---------------------------------------------------VDPDVAKFLNFASEQDNDESADC---------------------IREYIVDLALIPIHQYHGEVT-------------------------------DENKIEIGKNI-----------------YVMVVIELNPFAPA---------------ATGCG---------LF-NWKND-----------------------------------------------------------------------------------------------------LMMLWGKTSCDY-------------------------------------------------------------PIFKYRTTP-------------------------------------RED--------------------------------------------------------------LQSVSLLPSNYES-----------------VIQS---------------------ALIKRLENSYSSSVNQAAR
H696_00181_Falb_627950801            -VPNLRRADVD-ACATGAWSVG----------------------------------------------------------------------------------------------LASLQVPSLVV---------------------PLSRV-------FIRG--------------------------------FLESHS---------------------------------------------------------------------LYVPSAGEMAEWPAPAPAFSAAD------------------------------LDNTDALWEADQAGRSTEELQLLVR--------------SHVR-------------------------------------AVFLAVQEAIAS---------------LGG-----------AVLPKLNWSSPKDS---------------------------------------------------------------TWML--PNQT----------------------------------LKCMTAEDVF------------------LLLG-A---SDY-VSHDI----------------------LHPYDACDD----------------FEGGPVRPLR---------------------LSLTLRAWR--------------------------VFHVAR---EFRC-------FV----------------KNRR-----------------LIGISQRDYAT--------HYDFL--GAE----------------------------------------------------RISIVEAIVRFFYSKLC-------------------------------------------------LAPAIT--------------------------------------IDDFVFDVFI------------------------------------------PESF------------------------KRCILVDIAPYAT----------------VTEPL---------LF-NWTELTQAVVPP----------------------------------------------------------------------------------------------KEDLEPF------------------------------------------------------------------IHPPLTRDD-------------------------------------DIPPELLEPVILRLSGTAVGVQPAR----------------------------------------DTFLSRVPKDFID-----------------ASMGSIDIN----------------ALADAYRQQLLESDSSTDE
ACA1_183310_Acas_470491859           NIEQFRRQR------YQAAFGIES--------------------------------------------------------------------------------------------WHDHTFYTKSV---------------------PLSYR-------EAE---------------------------------ALSRYH---------------------------------------------------------------------YSLSPN-----------------------------------------------RVSEPLSDEDK----------------------------KLVK-------------------------------------ELEERLDEAIKG---------------DGRLAH--------GAFIKLDTRSPKDVVLYDFENQQVKDLVKAELEELYRNKRKQQAGGKEAAEEDLVLSPSGKISVSRAERDDNEETSTFVVA--TSRA----------------------------------MKITSGSEAL------------------YLLA-K---SDR-ISEDL----------------------NK-----IL----------------PYGEQHFD-----------------------LNLILREWRDEV-----------------------IERPQY---EFRA-------FV----------------HQNQ-----------------LNAMSQYFCFC--------KYDDL--IAR----------------------------------------------------EEEVKRTILDF-HES---------------------------------------------------IKDKIS--------------------------------------HSSYVIDFYL------------------------------------------TRD-------------------------NRVLIIELNPFHN----------------GAGAA---------LF-SWARDRERFMHGPFELRI----------------------------------------------------------------------------------------TKELKESPKEILPP-----------------------------------------------------------FWVRFIDSCFDHPDDAPATP--------------------------AAS--------------------------------------------------------------AKQNT------------------------------------------------------------------ESS
EIN_135800_Einv_471191192            -MLEVWKDL-----------------------------------------------------------------------------------------------------------ISDFTFKTWTF---------------------YITRT-------EAE---------------------------------SLERMN---------------------------------------------------------------------SYLWGTTYTS-------------------------------------------RPKYTQYDADD----------------------------LVIQ-------------------------------------GIKTKLDEVVSQ---------------HPP-----------PYFIRLGTRSAKDAFSPDMKLRFEKILTDIYKESNDINSSSKNGTTLEKPTMKDVQN--------------------YYIG--RFKL----------------------------------LKCTTTDEMM------------------DMLL-H---SER-IRTDL----------------------TRLLALPTI----------------DTKNF--------------------------EVLALREWCD-------------------------AVNPVL---EFRG-------FV----------------HKGI-----------------LTAVSQYNPIF--------YSDYL--VKN----------------------------------------------------KALIQKVLRTFFDEK---------------------------------------------------FKPTFEEKKKTLPDVSR---------------------------LTSYIVDFAV------------------------------------------TDVER-----------------------GEVKVVELNSFST----------------FAGAS---------MF-SWKKD-----------------------------------------------------------------------------------------------------IETLFGTKE----------------------------------------------------------------FEFRINENCRDN----------------------------------ESG--------------------------------------------------------------DMELF--PEDIK-----------------------------------------------MIAKAKKEAVQCKDK
AgaP_AGAP008049_Agam_158297128       ---ELEKQA----CMHVNWYEL----------------------------------------------------------------------------------------------FRKNTIKSCII---------------------PVPDD-------VLA---------------------------------YLRQDM---------------------------------------------------------------------LILPKECSNFTDVSTGEGFQTTHYNAFDDQFDGTDSE----------------GEDGADDDQEQ----------------------------PAFP-------------------------------------EFSQLLTDAIQS---------------LGG-----------NAFLKSDWHCPKDA---------------------------------------------------------------QWIT--LGQS----------------------------------LCVRDITDVY------------------QLLK-A---SSF-CKEDFR---------------------ER-----SE----------------VNGSG--------------------------YHVVLKKWR--------------------------DIHPGS---EFRC-------FV----------------RNRS-----------------LVAISPRHWPS--------YHEHI--ARE----------------------------------------------------RSDIVNDIVSLFKEK---------------------------------------------------IKETFP--------------------------------------LKDYVFDVYR------------------------------------------PAK-------------------------DNVIIMDFSLYGKG---------------HSDSL---------AF-DYDQLDDEAQVA----------------------------------------------------------------------------------------------TIEEEDD------------------------------------------------------------------PEFRYLPNDCGV----------------------------------QPI--------------------------------------------------------------KRNVYGFPQDFCNFFQGAA-----------PSSDGGAAAEEASVEGDSNNLI---NRLIEQCNLQQLHDLDNQN
CpipJ_CPIJ002632_Cqui_170033673      -------------CMLVNWYDQ----------------------------------------------------------------------------------------------FRKNTIKAQIV---------------------PVPDD-------VLE---------------------------------YLRQDL---------------------------------------------------------------------VILPKECSVLSSSDTSKTKHFNTYN----------------------------DEFSDDEEDDEEEL-------------------------PAFP-------------------------------------EFSKKLSDSIEK---------------LGG-----------SAFLKSDWHSPKDA---------------------------------------------------------------QWIT--LGQS----------------------------------LKVKDITDAY------------------QLLK-A---SSF-CKEDFAE--------------------RS-----SP----------------ANGGG--------------------------FHVVLKKWC--------------------------DIHPGS---EFRC-------FV----------------KGRS-----------------LVAISPRHWPS--------YHEHI--ATE----------------------------------------------------RNDIVSDIVSLFKEK---------------------------------------------------IKEAFP--------------------------------------LKDYVFDVYR------------------------------------------PAK-------------------------DHVVIMDFNVYGKG---------------HSDSL---------AF-DYDQL-----------------------------------------------------------------------------------------------------DADAVTATIEEEDV-----------------------------------------------------------PEFRYLPEDCGI----------------------------------QPN--------------------------------------------------------------PRNNYGFPQDVLE-----------------MFQGASG------------------AAGQPAEGDGLIHRLMDEW
HELRODRAFT_85046_Hrob_555694651      ----VKGFA------IREWYGK----------------------------------------------------------------------------------------------FSKLTVKTVFI---------------------SVDDL-------ILN---------------------------------YLSSDG---------------------------------------------------------------------IYLPEDY----------------------------------------------QQLSSTDSETK----------------------------TSLN-------------------------------------RFVTDVDLAIKQ---------------LGG-----------QVIPKFTWSVPKDA---------------------------------------------------------------TWIS--HDKS----------------------------------LKCINSDEVL------------------LLLK-S---SNQ-ITHDI----------------------DY-----ALGDGAAPE---------KSTIN--------------------------HVLALRKWV--------------------------NVNPCL---EFRC-------FV----------------RNNC-----------------LIAISQRNVQQ--------FFPEI--IEK----------------------------------------------------SHKIKSLIIDFINSE---------------------------------------------------IKNKFF--------------------------------------VENFTLDVFL------------------------------------------TDH-------------------------I-VKLLDFNPYGH----------------MSHSL---------LY-TYEELDKV--------------------------------------------------------------------------------------------------NILNDRN------------------------------------------------------------------ILFRYITSQKDV----------------------------------KAN--------------------------------------------------------------LHIWDAYPEDFLH-----------------LHGGL--------------------DINKFIDFMN------LKE
EIN_475520_Einv_471197419            YDELDRHTN----GLLEIWSDL----------------------------------------------------------------------------------------------VTEYSFNTRTF---------------------YLTRD-------ECK---------------------------------SIYQRGIHNKVLRIVGENK--------------------------------------------------------QYKTQE-----------------------------------------------FDDA------------------------------------HLA-------------------------------------VVKQKIDAEISQAH-------------WN------------GYFVRLGTRSPKDAFTEKMFFDFSKNLKMLFTARIESYHGQIP----------------------------------ENWA--KTFSVYDYMDYSKARFSC--------------------WKCKTSEEAL------------------SLFT-N---SDR-VLNDI----------------------ER-----ILKLEI------------DPQKF--------------------------EVLALREWCD-------------------------ELDPWY---EFRA-------VV----------------YKDN-----------------LTALTQYDSRF--------VLDNV--IQN----------------------------------------------------PKEVEDVVKTFFTKE---------------------------------------------------FKRNFCEKRDKLQEEERKR-------------------------LESYVIDFAY------------------------------------------LQKT------------------------KTVKVVEINSFCS----------------LCGVS---------LF-KWEKD-----------------------------------------------------------------------------------------------------ILVLFGKTH----------------------------------------------------------------FEFRYNTTAPDINWNYEVSENSLKKCFEIKNE--------------VYN--------------------------------------------------------------QVNKH------------------------------------------------------------------CQV
OXYTRI_06365_Otri_403346478          -YENLFDFQ--------NYYEQ----------------------------------------------------------------------------------------------LKPLTIPSEII---------------------KISLE-------DSL---------------------------------DLINGK---------------------------------------------------------------------MNNQ---------------------------------------------------------------------------------------------------------------------------------FLKNQVDIAIEK---------------LGG-----------KVFFKMR-RSPKDAFKTVRELIQY-----------------------------------------------------DWES--KWNIKNNEEASKYY------------------------MRIQTFDQLQ------------------ILCQ-S---SDR-IREDI----------------------ID-----AQN---------------NNQDD--------------------------LFLIIREFQ--------------------------ELDGYF---EFRC-------FI----------------CNNQ-----------------LNAVTIQ--P---------NQPEL--TKNQ---------------------------------------------------QEMFRK-----FFNN---------------------------------------------------KDYVFSEID-----------------------------------YSHAVIDVVV------------------------------------------NLD-------------------------MEFLIIEINPFGK----------------MAQSG---------KF-SWVID-----------------------------------------------------------------------------------------------------KDILYYR--------------------------------------------------------------------------------------------------------------------------------------------------------------------------------------------------------------------------------------------------------FQK
_Rsp._517993611                      DFEYLRQKQIA-TYWVSKWYDYLIINVP----------------------------------------------------------------------------------------DTMITFKSKLI---------------------YLTDN-------DIA---------------------------------DLYEYK---------------------------------------------------------------------INNI-----------------------------------------------------------------------------------------VPN-------------------------------------NLIKKISKAMSK---------------ISC-----------SCFIRTDAYSPKDL---------------------------------------------------------------LHRE--IVDT----------------------------------LEVTNAITAL------------------KLIT-Q---SER-CSSKL----------------------FG-----GD----------------NKIIS--------------------------KNIVIREYV--------------------------NYDTNY---EFRC-------FV----------------YNWR-----------------LTAISQSGFE---------YNPIL--HSK----------------------------------------------------KNIIYQSIIKF-WDK---------------------------------------------------FSRICP--------------------------------------YSECTMDIVY------------------------------------------NDKWIDNTLNH-----------------SGVVIIEFNLL------------------------------------------------------------------------------------------------------------------------------------------------------------------------------------------------------------------------------------------------------------------------------------------------------------------------------------------------------------------------------------------------------------ANI
mg113_MVc_363540335                  -FEYLRQKQIA-TYWVSEWYNYLIENVP----------------------------------------------------------------------------------------NTMITFKSKLI---------------------RLTDN-------DIA---------------------------------DLSKFK---------------------------------------------------------------------INNI-----------------------------------------------------------------------------------------IPD-------------------------------------SLAKKITKTISK---------------MSC-----------SCFIRTDAYSPKDL---------------------------------------------------------------LHRE--IIDT----------------------------------LEVTDAITAL------------------KLIT-Q---SER-CSSKL----------------------F------GD----------------DKVIS--------------------------RYIVVREYV--------------------------NYDINY---EFRC-------FV----------------YNWR-----------------LTAISQSGFE---------YNPVL--HSK----------------------------------------------------KNIIYQSIIKF-WDK---------------------------------------------------FSRICP--------------------------------------YSECTMDIIY------------------------------------------NDKWIDNTLNN-----------------SGVIIIEFNSFGEHM--------------NASSG---------LY-DWTKD-----------------------------------------------------------------------------------------------------HNILTRS------------------------------------------------------------------------KTP-------------------------------------HFL--------------------------------------------------------------LAEKP------------------------------------------------------------------LNV
NAEGRDRAFT_30015_Ngru_290999565      -MDEVNQCS------ISNWYYLYDGKTNPQIT------------------------------------------------------------------------------------PKRITILSKLI---------------------YLNDD-------FIE---------------------------------YLKHDG---------------------------------------------------------------------VVLPESIDNIQKVPNFDSDSDEEEEKYKEVQEKN-------------------EQDSDEDDYQV----------------------------PEFT-------------------------------------SLQMEIASAIEE---------------Y-E-----------EVFPKMNWSAPKDA---------------------------------------------------------------QWML--SDSKL---------------------------------LRCQTVSDVF------------------LVLK-S---SSF-VTHDV----------------------QQAYNNCTD----------------YTPEKEVR-----------------------RVLALRKWY--------------------------DVNPSM---EFRC-------FV----------------KNRN-----------------LIGISQRDISN--------FYSFL--VSD----------------------------------------------------KDMYREKLVGFWEKF---------------------------------------------------IKNSFP--------------------------------------LENYTVDLYI------------------------------------------SRT-------------------------GPVFIVDFNVYGP----------------PTSPL---------LF-ASFKKGH---------------------------------------------------------------------------------------------------LASDSTE------------------------------------------------------------------LEFRIIETDAGVM---------------------------------RPS--------------------------------------------------------------FKMYTGVPMDFFS-----------------VENAQ--------------------SMQDFIEKQQKFQ---SNE
MONBRDRAFT_27954_Mbre_167533509      ----------------------------------------------------------------------------------------------------------------------------------------------------PLPHR-------EDE---------------------------------DMEDDS---------------------------------------------------------------------TAQQSTTKSNEPTGH--------------------------------------ENSDDSDEEEEARPL------------------------PTFP-------------------------------------ELQVAITKAIAE---------------LGG-----------EVFPKLNWSAPKDA---------------------------------------------------------------VWIS--QTNS----------------------------------LKCRTAGDVF------------------LLLK-S---STH-AQHDL----------------------LHAYRCCQP----------------PHEERRPEK----------------------VFLVLKNYW--------------------------SSAPSQ---EFRC-------FV----------------RQGQ-----------------LVAISQRHVDQ--------HFPNL--AQL----------------------------------------------------ATDIRFQCYHLYHDD---------------------------------------------------IKRVVS--------------------------------------QPHLSFDVIC------------------------------------------DGN--------------------------RAILLDINAWTG----------------DSDPL---------LY-TWQELEALPAATSNSERDPEPTTYCTDEELEQFMQAHQAQERAQTGDATLAAVEPTPMVERTQTDRLNAHLLSALMRQLDVNGSPGTVGDDQDAGDDQDEWAEAPTLQEDEESVGDDHAAKNDEDNADDTWARQAA-------------------------------------------IVFRVVDDP-------------------------------------QPLKGA-----------------------------------------------------------SLATHAYPRDVVEL----------------AANG---------------------DVEVMMQALVQAQQAQNED
H012_gp856_ApMV_441432004            -FEYLRQAQIS-VYWVSNWYDYLMDNVK----------------------------------------------------------------------------------------DVMITFKSHLI---------------------HLSEE-------DIF---------------------------------NLFNYKDKNI-----------------------------------------------------------------IPFN----------------------------------------------------------------------------------------------------------------------------------LVKEISNIILE---------------LGG-----------SAFIRTDAYSPKDL---------------------------------------------------------------LYNH--TVST----------------------------------LKVTNAIDAL------------------QLVT-K---STR-CCSKL----------------------FD-----IN----------------NKIIS--------------------------KYLVLRQYI--------------------------DYDTNY---EFRC-------FI----------------YGWK-----------------LRAISQAGFE---------YNPQL--HEK----------------------------------------------------KKKIYDLILKF-WDK---------------------------------------------------FSRICP--------------------------------------FSECTMDIVY------------------------------------------NEKWIDNTLGN-----------------SGIIIIEFNSFGPHM--------------NACSG---------LY-NWVRD-----------------------------------------------------------------------------------------------------YYILTQS-----------------------------------------------------------------------------------------------------------------EK--------------------------------------------------------------PHFVL------------------------------------------------------------------AEK
Piso0_000399_Mfar_448122862          EPIELSRKQVD-DSIFSRWYPS----------------------------------------------------------------------------------------------YKGDTIESRVIS--------------------PLPQA-------FID---------------------------------ALNGES---------------------------------------------------------------------IHMPHEMGNPV------------------------------------------EANSDNDYSDWSEDEAQDGRSEDSDDEEDENNRRRLDPAKDFP-------------------------------------ELHDQIKRHIEE---------------LGG-----------AVMPKLNWSAPKDA---------------------------------------------------------------RWIM--ADNT----------------------------------LRCTSPADVY------------------LLLQ-A---SDH-IAHDI----------------------DAPYGECTDGA--------------AEQSGGKSDAAAP------------------LELVLRRWT--------------------------NINPAL---EFRV-------FV----------------RSGG-----------------VVAAAQRDRN---------HFPFL--AGL----------------------------------------------------KPTLARLIWNFVDST---------------------------------------------------LVPRLT--------------------------------------HPDAVVDIYV------------------------------------------PQPY------------------------RRVVLVDINPWSR----------------TADPL---------LF-AWNEI-----------------------------------------------------------------------------------------------------LHLAPERS-----------------------------------------------------------------PQFAFRLVD-------------------------------------RPNNAAFAAK-------------------------------------------------------EYSESMLPLDVVH-----------------AAADPA-------------------ALVDLARASRASQDRLESR
CDC123_Calb_68470928                 TSEEVLQCS------YSNWSKLFP--------------------------------------------------------------------------------------------GKT--FPSKIIK--------------------PLPST-------FLD---------------------------------YLASES---------------------------------------------------------------------IRLPSNTNNKKITVLEAD-----------------------------------SDNEYSDWDDEEDQQQDAEHNDNVF--------------SQFQ-------------------------------------DIQDKIDASIQE---------------MGG-----------AVFTKLNWSSPKDA---------------------------------------------------------------KWIM--PGNT----------------------------------IKCQNVSDVY------------------LLLN-S---SDH-IGDDL----------------------DNPFSEVQK----------------KKTIPEKVD----------------------YELVLTKWQ--------------------------EINPAY---EFRV-------FV----------------KDHR-----------------IIGISQRDNN---------KYEFL--QGL----------------------------------------------------KSELNEKITQFVEDH---------------------------------------------------VIPKLKSDTQ----------------------------------LSKYIVDVYV------------------------------------------SKN-------------------------D-IYIIDINPFSR----------------KSDSC---------LF-TWVEL-----------------------------------------------------------------------------------------------------LDKKDKHDNH---------------------------------------------------------------HELRLVENQN------------------------------------FAK--------------------------------------------------------------EFSESQVPIEVVG-----------------ATMDTE-------------------AMVELAREWD---KLQAKE
glt_00905_MVg_451927830              -FEYLRQAQIS-VYWVSNWYDYLMDNVK----------------------------------------------------------------------------------------DVMITFKSHLI---------------------YLTEE-------DIS---------------------------------NL-----------------------------------------------------------------------------------------------------------------------------------------------------------------------FNYKANNIIPV----------------------------NIVNKLSNIILE---------------LGG-----------SSFIRTDAYSPKDL---------------------------------------------------------------LYNH--TVSS----------------------------------LKVNNAIDAL------------------QLVI-K---SPR-CCTKL----------------------FD-----IN----------------NKIMS--------------------------EYLVLRQYI--------------------------DFDTNY---EFRC-------FI----------------YGWK-----------------LRAVSQAGFE---------YNSEL--YDK----------------------------------------------------KKIIYDLVLKF-WDK---------------------------------------------------FSRICP--------------------------------------FSECTMDIVY------------------------------------------NEKWIENTLGN-----------------SGIMIIEFNSFGPHM--------------NACSG---------LY-NWVRD-----------------------------------------------------------------------------------------------------YYILTQS-----------------------------------------------------------------------------------------------------------------EK--------------------------------------------------------------PHIVL------------------------------------------------------------------AEK
L884_APMV_311978298                  DFEYLRQSQIA-VYWIHEWYDYLIKNSD----------------------------------------------------------------------------------------NKNITFKTKLI---------------------ELSNE-------DIE---------------------------------SLFNFK---------------------------------------------------------------------TYGI-----------------------------------------------------------------------------------------IPE-------------------------------------SLLKIIDDSITE---------------INN-----------LCFVRTDAYSPKDL---------------------------------------------------------------VFEN--KIDN----------------------------------LKVSDALTAI------------------KLIT-D---SER-CCQKL----------------------F------SN----------------DQIIS--------------------------KYLAIREYV--------------------------NLDTNY---EFRC-------FI----------------YNWN-----------------LRAICQSGFE---------YNSEL--HAK----------------------------------------------------KKIIRDSILKF-WNK---------------------------------------------------FESICP--------------------------------------YSECTMDIIY------------------------------------------DNNFKNTLND------------------SCIMVIEFNSFGPHM--------------NADSG---------LY-DWDRD-----------------------------------------------------------------------------------------------------YILLTKS--------------------------------------------------------------------------------------------------------------------------------------------------------------------------------------------------------------------------------------------------------NQP
H012_gp858_ApMV_441432002            YKTELWKTNLS-NYYDLINYPI----------------------------------------------------------------------------------------------FWTYDF--------------------------NLSETKILLKACQIG---------------------------------SISGRYPK-------------------------------------------------------------------IYND-----------------------------------------------------------------------------------------ELK-------------------------------------DIIDKLSSCWKS-----------------G-----------SWFMRLDACSTKDS---------------------------------------------------------------VVEL--PF---------------------------------------ESPETII------------------ASIV-T---SRR-A---------------------------------IN----------------ALTDNIN------------------------RNLNTRIYFTHYDK---------------------KWNSSR---ELRC-------FI----------------RKNK-----------------LTAISQYCWTNRD------FFCEW--TEED---------------------------------------------------LINLAKKINKLVNNI---------------------------------------------------IEELSNRIG-----------------------------------TKDMVMDIYL------------------------------------------DDQ-------------------------NNLQIIELNNFGYWL--------------ACGSA---------LF-HWIKD-----------------------------------------------------------------------------------------------------YDKLYNT--------------------------------------------------------------------------------------------------------------------------------------------------------------------------------------------------------------------------------------------------------DGD
CNN00310_Cneo_58262274               LFPTLTREQID-AARTSAWYDT----------------------------------------------------------------------------------------------FADLTFPAQFI---------------------DLKKL-------GEEEEFLRTVGRSQPPPI------------------STVSDS---------------------------------------------------------------------LPTPRS-----------------------------------------------RSNSGADDSSS----------------------------SSSVAPVYHLP------------------------------KLNAAVRQAIEQ---------------YGG-----------AVFPKLNWTSPKDA---------------------------------------------------------------AFIL--PQASSGP-------------------------------LYCSSPADMY------------------LLLK-S---SDF-ISHDIDH--------------------ERAYSGVQDPQV-------------EEKPK--------------------------IELVLKKFE--------------------------SLNPSR---EVRC-------FV----------------RNNV-----------------LVGITQRDMN---------FYDHL--QPEEV--------------------------------------------------RSKISRTVREFWEDE---------------------------------------------------IRENYEG-------------------------------------GDDYVFDLYL------------------------------------------SPSF------------------------DSATIIDFQPYRE----------------STDPL---------LF-TYEEL-----------------------------------------------------------------------------------------------------LAILQDSVSPSDSEFRPRL------------------------------------------------------PLFKIIDSQAHPAVTR------------------------------NAP--------------------------------------------------------------TYQSNMMPLEMIE-----------------FSEGR--------------------NMAEFKEAWDEAVRMGMTE
CHLNCDRAFT_21599_Cvar_552837040      --------------MISSWYPR----------------------------------------------------------------------------------------------FQRVTFKTVIL---------------------PLPQP-------VLD---------------------------------WLVSDG---------------------------------------------------------------------LHLPPDSQAFAKRSPL-------------------------------------EEYATEDDYQEWTSEDEASEGSAAPPPP-----------PELA-------------------------------------TLRQQLQAAIES---------------LGG-----------RVVPKLSWSCPKDA---------------------------------------------------------------VWMS--PSAS----------------------------------LCCANAEEVL------------------LLLR-S---SDR-VAHDI----------------------CH-----ALQQAAGGAADGGADGG-AEAGPSGGAAASAAAGGGAAVPAVQ------HCLALRRWH--------------------------DLQPGR---EFRC-------FV----------------RGGE-----------------LVGASQRDVTH--------CYSFL--RDE----------------------------------------------------RRELAAALQAFHARH---------------------------------------------------IQGRFP--------------------------------------HPHYTYDAYV------------------------------------------AAS-------------------------GAVRLLDFNPLRG----------------TTSPL---------LF-SW-----------------------------------------------------------------------------------------------------------------------------------------------------------------------------------------------------------------------------------------------------------------------------------------------------------------------------------------------------------------------HEL
ACA1_050010_Acas_470469110           AEYELKETQVL-NCMFASWYPN----------------------------------------------------------------------------------------------YSSHTFKSRVV---------------------PLHDE-------FVE---------------------------------YLQDEGTIYLPSSAYKERKREMVDYSVDEWAEDDPALQRRRAAAAKLKQIHEIGTQSESDSESSEEEEEEEEPKYVEFPEL-----------------------------------------------EEESDSESSEEEEEEEEPKY-------------------VEFP-------------------------------------ELEEEVERAIRE---------------LDG-----------AVVPKLNWSSPKDA---------------------------------------------------------------SWMS--VGGT----------------------------------LNCSSFADIC------------------LLLK-S---SDF-VSHDL----------------------SYAFSESVDEKGRPSQL--------KRPPQ--------------------------FTLVLRKWF--------------------------ALSPAL---EFRC-------FV----------------KRRT-----------------LVGISQRDPAK--------FYPFL--PRM----------------------------------------------------KAELLELIEDFFDAH---------------------------------------------------ILSSFP--------------------------------------DENVVVDVYV------------------------------------------EEAK------------------------KRVWLMDFNPFGP----------------VTDGM---------LF-SWAEIYTAPTPTATTTTQQRPRPAM---------------------------------------------------------------------------------EAENESKTTKEERSKTATTVVDVD-------------------------------------------------VDFRIVESEAEV----------------------------------RVS--------------------------------------------------------------TANSSRFPVDVARGMFDA------------ATAT---------------------DINKLVEMMQ------SQG
Ot04g02590_Otau_308802928            RIPAPTVAELD-ATRIASWRRALIDKV-----------------------------------------------------------------------------------------AKRASFRTELI---------------------DVPES-------FVR---------------------------------YLLADG---------------------------------------------------------------------VACGEE-----------------------------------------------DASLPKRIAQDAFDAKESAERFGEGCEEDDAGDADATAREAFE-------------------------------------NFTREIGAAIER---------------LGG-----------EVAPKFAWSAPKDA---------------------------------------------------------------TWVT--AGNT----------------------------------MKCRNADEVV------------------LLLK-A---SDS-VTHDL----------------------TEAYRACADYVVDEME---------DEEDRAVREHAN-------------------TALALREWY--------------------------DLNPSM---EFRC-------FV----------------KTYN-----------------LVAVSQRHVND--------FYEFL--LRE----------------------------------------------------KEEIEEAIAEFFENE---------------------------------------------------ISKHYT--------------------------------------GRDYVFDVYV------------------------------------------TPKT------------------------HKVKIMDFNVWGG----------------TTLPL---------LF-DWNELESRGSDQDCVP------------------------------------------------------------------------------------------EDERGWTDN----------------------------------------------------------------VEFRVIQSQG------------------------------------HIR--------------------------------------------------------------PGLQLGVPFELYD-----------------T----------------------------------------SEG
OSTLU_31179_Oluc_145345498           RVPAPTRAALD-ACRDARWRAT----------------------------------------------------------------------------------------------FAAHGFRAVAL---------------------EVPED-------FIR---------------------------------YVLADG---------------------------------------------------------------------VVARETDAAMPRRVAQDAFDAAESAARFARARATTTTME--------------EEEAEDASARDGARR------------------------ATFA-------------------------------------AFERAIEDAIEA---------------LGG-----------EVAPKFAWSAPKDA---------------------------------------------------------------AWVA--AGNT----------------------------------MKCRNADEVV------------------LLLK-A---SDA-VAHDL----------------------TEAYGACEDYARGDGSEE-------SEEDRAVREHAA-------------------SVLTLREWY--------------------------DLNPSM---EFRC-------FV----------------KNRN-----------------LVAASQRHVND--------FYEFL--VRD----------------------------------------------------KDAIEDAIALFWESN---------------------------------------------------VSCTSWHDD-----------------------------------QVDYVFDVYV------------------------------------------TPKT------------------------KKVKIIDFNVWGG----------------TTLPL---------LF-EWHEL-----------------------------------------------------------------------------------------------------EAMNRDRAEGDDARGYADE------------------------------------------------------IEFRIIESQG------------------------------------HIR--------------------------------------------------------------PGLQLGVPFDLYD-----------------TSEG---------------------GAISEFLEEQRRRQEQESC
MICPUN_70546_Msp._255085574          ----------------------------------------------------------------------------------------------------------------------------------------------------ALPPD-------FVA---------------------------------YLLEDGLSLAADSQAMPARIRPD----------------------------------------------------IAEQME-----------------------------------------------SAFTLSDEEDDAGVADA----------------------RHFP-------------------------------------ELEDAMREAIES---------------LGG-----------AVTPKLTWSSPKDA---------------------------------------------------------------VWMA--TTND----------------------------------TRCQNPAEVM------------------LLLK-A---SDA-VAYDL----------------------QDAYAQCVD----------------ASESSSAALTTG-------------------VVLTLRKWA--------------------------GLSPSM---EFRC-------FV----------------RRGN-----------------LRGVCQRDVAN--------FYPFL--PEQ----------------------------------------------------VGQIEEAIAVFWQEN---------------------------------------------------VHGVFP--------------------------------------VVDYVMDVYV------------------------------------------TSR-------------------------KKVKIVDFNPYGG----------------ATLPL---------LF-DW-----------------------------------------------------------------------------------------------------------------------------------------------------------------------------------------------------------------------------------------------------------------------------------------------------------------------------------------------------------------------NEL
PHYPADRAFT_146294_Ppat_168052311     KESDVLRCQ------IQEWYPR----------------------------------------------------------------------------------------------FRTHSIRTQVV---------------------QLPEE-------FVS---------------------------------FLLEDG---------------------------------------------------------------------IFLPADSDAMPSRTRVDMPQL--------------------------------QAEDYSHWEEEDDESGVPAV-------------------PTFP-------------------------------------ELAAEVQTAIES---------------LGG-----------TVVPKLNWSAPKDT---------------------------------------------------------------AWIA--TTGS----------------------------------MRCQNFGEVS------------------LLLK-A---SDN-VVHDL----------------------CHAFDSCDD----------------KSMERPFQ-----------------------VVLALRKWY--------------------------DLRPEL---EFRG-------FV----------------RGNL-----------------LVGVCQREVTG--------FYETL--LGR----------------------------------------------------EEELSDLILDFFLDN---------------------------------------------------LKEEFG--------------------------------------GGDYTFDVYV------------------------------------------TKD-------------------------KKVKLIDFNTWRG----------------STLPL---------LF-TWDELEERFSEAGTL-------------------------------------------------------------------------------------------GTALNPD------------------------------------------------------------------FDFRIVTSEGLV----------------------------------QLG--------------------------------------------------------------LRVGTGVPIDFV-----------------------------------------------------------NQG
SELMODRAFT_118326_Smoe_302803676     KEVEVLECQ------VQQWYPR----------------------------------------------------------------------------------------------FKSVSIATEIH---------------------RLPEA-------FVS---------------------------------HLLDGD---------------------------------------------------------------------FFVPSSDLPSRLVPEIPELQA--------------------------------DGYEKWDEEDEEDGGEQQQQ-------------------PSFP-------------------------------------ELEAEVEASIRR---------------LGG-----------AVLPKLNWSAPKDA---------------------------------------------------------------TWIS--SSKN----------------------------------LKCQSFGDVS------------------LLLK-A---SDS-IVHDL----------------------CHAFDNCDDKP--------------ASSTGRPEE----------------------LVLALRKWY--------------------------DLRPEM---EFRA-------FV----------------RRGA-----------------LLGVCQREVTG--------FYSSL--VSS----------------------------------------------------KDSLRTAISGFFENS---------------------------------------------------LLGKFE--------------------------------------LESYTFDVYV------------------------------------------TKD-------------------------LRVRLLDFNPWGG----------------STLPL---------LF-SWEEL-----------------------------------------------------------------------------------------------------EAMGDGSDELPAKSTP---------------------------------------------------------VEFRIIEREGLV----------------------------------QPN--------------------------------------------------------------LTATSGVPLDYLD-----------------TGPGSA-------------------WDVFFRKTEDELKNQASEE
SELMODRAFT_100293_Smoe_302784068     KEVEVLDCQ------VQQWYPR----------------------------------------------------------------------------------------------FKSVSIATEIH---------------------RLPEA-------FVS---------------------------------HLLDGD---------------------------------------------------------------------FFVPSSDLPSRLVPEIPELQA--------------------------------DGYEKWDEEDEEDGGEQQQQQ------------------PSFP-------------------------------------ELEAEVEASIRR---------------LGG-----------AVMPKLNWSAPKDA---------------------------------------------------------------TWIS--SSKN----------------------------------LKCQSFGDVS------------------LLLK-A---SDS-IVHDL----------------------CHAFDNCDD----------------KPSSSTGRPEE--------------------LVLALRKWY--------------------------DLRPEM---EFRA-------FV----------------RRGA-----------------LLGICQREVTG--------FYSSL--VSS----------------------------------------------------KDSLRTAISGFFENS---------------------------------------------------LLGKFE--------------------------------------LESYTFDVYV------------------------------------------TKD-------------------------LRVKLLDFNPWEG----------------STLPL---------LF-SWEEL-----------------------------------------------------------------------------------------------------EAIGDGSDELPAKPTS---------------------------------------------------------VEFRIIEREGLV----------------------------------QPN--------------------------------------------------------------LTATSGVPLDYLDTGPGSAWD---------VFFR---------------------RTEDELKNQA------SEE
EDA35_Atha_15235584                  KEDEVNRCQ------IQNWYPR----------------------------------------------------------------------------------------------FKSLTIKTKFH---------------------KLPES-------FIN---------------------------------YLIDDSGPFLLPHSVTNE---------------------------------------------------------DAMPNR-----------------------------------------------VHNFEEEDDFQVPEEASDDEPLNR---------------PSFP-------------------------------------ELEIEIRESIET---------------LGG-----------TIIPKLNWSSPKDA---------------------------------------------------------------AWIS--PSQN----------------------------------LSCTCFNEIA------------------LLFR-S---SDS-LTHDL----------------------FNAYDSCSDKVS-------------SRPES--------------------------FYLALRKWYP-------------------------SLKPEM---EFRC-------FV----------------KSNE-----------------LVGICQREVTT--------FYPVL--LNE----------------------------------------------------KDLLKGLIEEFFDDK---------------------------------------------------IRFEFE--------------------------------------SENYTFDVYV------------------------------------------TKE-------------------------RRVKLIDFNTWCG----------------STLPL---------MY-TWEEL-----------------------------------------------------------------------------------------------------EKIHGECDE----------------------------------------------------------------LELRIVESRL------------------------------------SVL--------------------------------------------------------------PGLKTAVPYDYLD-----------------VSAGSGWA-----------------QVLKIVEEEFQKDDQSSDE
EUGRSUZ_E02475_Egra_629108734        RQEEVDRCQ------IQEWYPR----------------------------------------------------------------------------------------------FKSASIKTHVH---------------------QLPEA-------FVQ---------------------------------YLLGDSGPFVLPISVTGEDALPNRVRNPVDE--------------------------------------------DDLAVS-----------------------------------------------EGSGDESEEFPP---------------------------PSFP-------------------------------------ELELEIKESIES---------------LGG-----------AVFPKLNWSSPKDA---------------------------------------------------------------AWIS--GSGN----------------------------------LRCTSLSEIA------------------LLLR-A---SDS-TTHDL----------------------CHAYDSCSD----------------KTSSRPQS-----------------------FFLALRKWQP-------------------------ALRLEM---EFRC-------FV----------------RNKL-----------------LVGICQREVTT--------FYPVL--LEK----------------------------------------------------KDNLKLLIEDFFENE---------------------------------------------------VCPNFE--------------------------------------LDNYTFDIYV------------------------------------------TKD-------------------------GRVKVLDFNPWGA----------------FTLPL---------LF-SWDEP-----------------------------------------------------------------------------------------------------SEGVDES------------------------------------------------------------------VDFRVVESRC------------------------------------AVR--------------------------------------------------------------PGLKTAVPYDYLD-----------------TSPGS--------------------SWDQFLRNADEEPRRQTEE
EUGRSUZ_K03201_Egra_629083251        RQEEVDQCQ------IQEWYPK----------------------------------------------------------------------------------------------FKSVSIKTHIH---------------------QLPES-------FVQ---------------------------------YLLGDSGP-------------------------------------------------------------------FVLPISITGEDALPNRVHNPVDEDDL---------------------------EVPEGSGDESEEFPP------------------------PSFP-------------------------------------ELELKVKESITS---------------LGG-----------AIFPKLNWSSPKDA---------------------------------------------------------------AWIS--GSGN----------------------------------LRCTSLSEIT------------------LLLR-A---SDS-STHDL----------------------CHAYDSCSD----------------KTSSRPQS-----------------------FFLALRKWQP-------------------------ALRPEM---EFRC-------FV----------------RNKL-----------------LVGICQREVTT--------FYPVL--LEK----------------------------------------------------KDHLKVLIEDFFESE---------------------------------------------------VCPKFE--------------------------------------SDNYTFDIYV------------------------------------------TKD-------------------------GRVKVLDFNPWGA----------------FTLPL---------LF-SWDEL-----------------------------------------------------------------------------------------------------SEGDDEV------------------------------------------------------------------VDFRTVESRC------------------------------------AVR--------------------------------------------------------------PGLKTAVPYDYLD-----------------TSPGS--------------------GWDQFLRNADEELRRQTEE
glt_00911_MVg_451927836              YKTELWKTN------LSNYYD-----------------------------------------------------------------------------------------------LINYTVS-WTY---------------------DFNLS-------ETK---------------------------------ILLKACQIGSISGKCPK----------------------------------------------------------IYDD-----------------------------------------------------------------------------------------ELG-------------------------------------EIINKLNSSWKS-----------------G-----------SWFMRLDASSTKDS---------------------------------------------------------------IINP--PF---------------------------------------KSAQSVI------------------TSIV-T---SRR-T---------------------------------IN----------------ALTDNIN------------------------RNLNTRIYFTPYDS---------------------KWNSSR---ELRC-------FI----------------RNNK-----------------LIAISQYCWTRCEF-----FCDFS--KDE----------------------------------------------------LISLADRINKLVNNI---------------------------------------------------IEDLSNRID-----------------------------------TKDMVMDIYL------------------------------------------DDN-------------------------DNLQIIELNSFGYWL--------------ASGSA---------LF-HWIKD-----------------------------------------------------------------------------------------------------YDKLYNT--------------------------------------------------------------------------------------------------------------------------------------------------------------------------------------------------------------------------------------------------------NGD
NCU06497_Ncra_85083526               TFPPVTRDHIL-HCSYDYWFPK----------------------------------------------------------------------------------------------YRTSCIRSRVI---------------------PLSRE-------FIS---------------------------------YIREDG---------------------------------------------------------------------IILADDEPGN-------------------------------------------ENDSDDDDDWE----------------------------PTVPSSEIPAPPRNPGDADNDSDSDDEDSTPAKLPPNKRFPDLHNAINAAIKA---------------LGG-----------AAAPKLNWSSPKDA---------------------------------------------------------------TWISRHPN-T----------------------------------VKCTSANDVY------------------ILLK-S---SSF-ISHDL----------------------DHAFDDCVPSTTTSTP---------QSPSSTAAAAAAQVQQQQQQPQGFT------PVLVLRSFF--------------------------SPLPSL---EFRC-------FV----------------KDRN-----------------LIAITQRDLN---------YYAFL--RSL----------------------------------------------------QPAIIARCRELFNTK---------------------------------------------------LKYTFP--------------------------------------DSSFVFDVYIPEAAYRSDSES-------------------------------DDDETSEARSRL----------------ARARLIDINPWAP----------------RTDTI---------LF-GWEELLEADVKMPVLGTAESTPEKKEETVRLRFSATGAAAAAAGGDDNLTEP------------------------------------------------------EEEDDNNEQNHFDDDETTTDDEHEEEYE---------------------------------------------VELRLVEQDDPAAYNF------------------------------SSP--------------------------------------------------------------QYSAHKMPKDVVD-----------------ASMAGEG------------------GMREFAREWQRLQDQRGGG
NFIA_050590_Nfis_119467610           PFPPVTYSHIL-HCSYHHWQPR----------------------------------------------------------------------------------------------YRTLTPKSRAI---------------------PLTPS-------FVS---------------------------------YLRADG---------------------------------------------------------------------IVLPPETTRPQGDDDLDT-----------------------------------FSDDGADEESD----------------------------PSVEWQ-----------------------------------EIHSQIKSTISE---------------FGG-----------KVTPKLNWSAPKDA---------------------------------------------------------------VWMS--ATND----------------------------------LQCRTPNDIY------------------LLLK-S---SDF-ITHDL----------------------EHPFDGCVP----------------DTDDSSEAPATQPDIP---------------YHLVLRKYV--------------------------NFNPSL---EFRC-------FV----------------RNRV-----------------LLCMCQRDQN---------HFDFL--FSL----------------------------------------------------RDTLRSRIQAFFDEK---------------------------------------------------LKDTFP--------------------------------------DPNFVFDVYI------------------------------------------PEPH------------------------QRVWLIDINPWAD----------------RTDPL---------LF-SWLEI-----------------------------------------------------------------------------------------------------LQMKDPIGIKEEDADGPEESFVRLSLNGANPTVVEVNGDEDCESEEEDVGSADDGDDLPFL------------PEFRLVKRDDPEAYSF------------------------------STP--------------------------------------------------------------QYSAHKLPREVVD-----------------ASLSGPG------------------GMSEFLGKWQDILAKQTQE
AOR_1_234114_Aory_169777089          PFPPVTHSHIL-HCSYHDWQPR----------------------------------------------------------------------------------------------YRALTPKSRLI---------------------PLTVP-------FIS---------------------------------YLRADG---------------------------------------------------------------------IVLPPENATPTDDDNL-------------------------------------DTYSDDEADEQPD--------------------------PSTEWE-----------------------------------EIHTQIKTTISE---------------LGG-----------IITPKLNWSAPKDA---------------------------------------------------------------TWMA--ATND----------------------------------MQCRTPNDIY------------------LLLK-S---SDF-ISHDL----------------------ELPFDDCVP----------------DMPDSTTTPDVP-------------------YHLVLRKYV--------------------------NFNPSL---EFRC-------FV----------------RDRV-----------------LLCICQRDQN---------HFDFL--FPL----------------------------------------------------RETLRSRIQAFFDEK---------------------------------------------------LKDTFP--------------------------------------DPSFVFDVYIP-----------------------------------------PPH-------------------------QRVWLIDINPWAV----------------RTDPL---------LF-SWLEILNMKDPIGIQEEDGAEEQFVRLSLNGNTVTGVVGAAEGS--------------------------------------------------------------ESSDTEDESADDVDEDSPFF-----------------------------------------------------PEFRLVKRD-------------------------------------DPEAYAFTTP-------------------------------------------------------QYSAHKLPKEVVD-----------------ASISGPG------------------GMSEFLGKWQDILAKQAQE
CE11_00885_MVc_425701749             YKTELRKTFLN-RYAHIINYPIN---------------------------------------------------------------------------------------------WTH-----------------------------ELTLE-------ESKI--------------------------------ILHASK---------------------------------------------------------------------IYQTAK-----------------------------------------------CSIE------------------------------------IFN-------------------------------------NVLNPIKSRLEEKW-------------ISG-----------RWFMRLDALSPKDS---------------------------------------------------------------NYKM--PY---------------------------------------TNPSDII------------------NSIV-T---SKR-TFNAL----------------------LD-----NI----------------SLNIN--------------------------TKLYFVTYNE-------------------------NWKPSH---ELRC-------FV----------------YNKK-----------------LTAISQYCWTKEEY-----YCDKT--NDE----------------------------------------------------LIEIATKVNYFITNI---------------------------------------------------IDNICAQID-----------------------------------TTNIIFDLYL------------------------------------------NDD-------------------------LSMNIIELNCFGYWL--------------ASGSA---------LF-HWIRD-----------------------------------------------------------------------------------------------------KDKL-------------------------------------------------------------------------YNTDG-------------------------------------NIYF-------------------------------------------------------------RILKN------------------------------------------------------------------LSN
PHATRDRAFT_11084_Ptri_219115585      ----VHACQ------FSSWYATFANLPPNELG------------------------------------------------------------------------------------RKNVTVPSEIL---------------------NLPET-------FRD---------------------------------YLLCDG---------------------------------------------------------------------VQLPAGARTSGMLAMSGVG----------------------------------DDESVWSSDSEAEEVSNEL--------------------FHFS-------------------------------------ALNSAIDAAIRR---------------LGG-----------LVAPKLNWSSPKDA---------------------------------------------------------------IWVN--GG-T----------------------------------LQCKTAGDVY------------------LLLK-S---SDF-CAFDI----------------------QHSWKEVRDGD--------------DTSDETATDCHGAIP----------------LQLVLRKWC--------------------------NLYPSQ---EFRC-------FV----------------REQE-----------------LVAVSQRQHSQ--------HFEHL--VRD----------------------------------------------------QYLIRSLVVEFFDEI---------------------------------------------------IKPHSQS-------------------------------------LNNYTFDVYL------------------------------------------DKK-------------------------ERVWLVDFNVWGR----------------RTDPL---------LF-TW-----------------------------------------------------------------------------------------------------------------------------------------------------------------------------------------------------------------------------------------------------------------------------------------------------------------------------------------------------------------------DEL
THAPSDRAFT_2157_Tpse_223995977       SRPKPTAAEVH-ACQFSSWYASFRNMKLTPKDNTTTQSTTDDTTNEEFELHAQPTKKL----------------------------------------------------------RKNVTIESIIIR--------------------PLPSD-------FIE---------------------------------YLLSDG---------------------------------------------------------------------VRLPDCATKVSSCMKDNNND---------------------------------DGRWDSDDDDEGNNSQSSTEELKK---------------YSFP-------------------------------------SLTAEIQSALSV---------------LGGTVNK-------GCMPKLNWSSPKDA---------------------------------------------------------------TWMN--CG-S----------------------------------LKCTKVGDVY------------------LLLK-S---SEF-VSFDLESAWED----------------LAVESEDET----------------SHDTKKNGMNNLAMHDRNNASNTNRIPHDFQYELVLRKWC--------------------------NLHPSM---EFRC-------YV----------------YDHE-----------------LVGISQRHPSK--------YYPYL--QPP---------------------------------------------------------------SDET---------------------------------------------------SHPIVN--------------------------------------IIQQFFDIYV------------------------------------------RNRFAQGAVHSQ----------------ERTWIIDVNVWGS----------------RTDAL---------LF-DWK--------------------------------------------------------------------------------------------------------------------------------------------------------------------------------------ELA-------------------------------------ELG--------------------------------------------------------------DSSHL------------------------------------------------------------------FQW
BATDEDRAFT_87016_Bden_575476564      AFPGFTIQHVN-NCCFSSWYSH----------------------------------------------------------------------------------------------FSQCTFKSIVIQ--------------------PLPET-------FID---------------------------------YLNADG---------------------------------------------------------------------IYLPSEVNHAPLAEYDVDSDTSSEVNQ--------------------------DVSDTEEDTPN----------------------------PSFP-------------------------------------TLEAHIISSITR---------------LGG-----------RIFPKLNWSSPKDA---------------------------------------------------------------AWIT--FATT----------------------------------LQCTTPADIF------------------LLLK-S---SDF-IAHDL----------------------SHAYEECVD----------------FEPGHDQDRPKE-------------------FELVLREWF--------------------------DLAPSM---QFRC-------FV----------------HHGD-----------------LVGMCQRDSGN--------YFEFL--KLN----------------------------------------------------RNTIELDLCRFFDSK---------------------------------------------------ISGKFP--------------------------------------DPSYVFDVYM------------------------------------------NART------------------------RNIWLMDFNPFGP----------------TTDAL---------LY-TWQEI-----------------------------------------------------------------------------------------------------LESTESKLR----------------------------------------------------------------IVESTAEAEHH-----------------------------------AKS--------------------------------------------------------------NFSHNRLPKEVFD-----------------LSNNAS-------------------IHEFSERFKQGLLHAQFDK
LBA_00843_MVl_448825806              YKTELRKTFLN-RYAHIINYPIN---------------------------------------------------------------------------------------------WTH-----------------------------ELTLE-------ESK---------------------------------IILHASK--------------------------------------------------------------------IYQTAK-----------------------------------------------CSIE------------------------------------IFN-------------------------------------NVLNPIKSRLEEKW-------------ISG-----------RWFMRLDALSPKDS---------------------------------------------------------------NYKM--PY---------------------------------------TNPSDII------------------NSIV-T---SKR-TFNAL----------------------LD-----NI----------------SLNIN--------------------------TKLYFVTYNE-------------------------NWKPSH---ELRC-------FV----------------YNKK-----------------LTAISQYCWTKEEY-----YCDKT--NDE----------------------------------------------------LIEIATKVNYFITNI---------------------------------------------------IDNICAQID-----------------------------------TTNIIFDLYL------------------------------------------NDD-------------------------LSMNIIELNCFGYWL--------------ASGSA---------LF-HWIRD-----------------------------------------------------------------------------------------------------KDKL-------------------------------------------------------------------------YNTDGN------------------------------------IYF--------------------------------------------------------------RILKN------------------------------------------------------------------LSN
cdc123_Ppal_281208675                SRDILFKERKD-KCQFARWYST----------------------------------------------------------------------------------------------FRRLTFESVII---------------------ELPKI-------VVD---------------------------------YLNSDHFSTETTSFPKYKLDDYEDDIGDDDEQWSTPVGKSKESSMFNKK--------------------------YYQSYD-----------------------------------------------SDDDTDDSDDDKVEDNNNNNRTNEVKIDP----------QQLK-------------------------------------SFTDKIDEAIKK---------------LGG-----------ECVPKLNWSSPKDA---------------------------------------------------------------TFMN--IHAS----------------------------------LRCSSSSDIL------------------LLLK-S---SDF-INHDL----------------------AQ-----FDNDIKDLQP--------DDITP--------------------------LTLVLRRWA--------------------------NVNIAL---EFRC-------FI----------------KDNQ-----------------LIAISQRDTSA--------FFDFL--PAK----------------------------------------------------KELIQSKIKSFAEQH---------------------------------------------------IINKFN--------------------------------------DVSYCFDVCFLD----------------------------------------TNL-------------------------NTVTLMDFNPIHP----------------STDSL---------LF-DWYELFPEELEHQE--------------------------------------------------------------------------------------------IDESVKPLEH---------------------------------------------------------------FEFRIVESNEGI----------------------------------RPN--------------------------------------------------------------LSMSSRLPSDLVN-----------------MQST---------------------SEINEMLSKF------KDH
cdc123_Dfas_470261556                NNDKYFENK-L-KCSFEQWYRV----------------------------------------------------------------------------------------------FKNNTFSSIVI---------------------PLPKI-------FID---------------------------------YLNSDH---------------------------------------------------------------------FTMSEESFPEFRTEDNGEDDEEWSSTTPSDMSTKLDKRYYQSYDSESESESDNEQDEDDEDEDEKEKDNNNNNNNNKTNRKISE--------KDFP-------------------------------------ELIKEIEDAIAK---------------LGG-----------EVVPKLNWSSPKDA---------------------------------------------------------------TWMN--IHSS----------------------------------LKCLTPTDVL------------------LLLK-S---SDF-INHDL----------------------CQ-----FQIEKDQEEILKD-----DSISP--------------------------FTLVLRKYH--------------------------NLFHSM---EFRC-------FV----------------KNNQ-----------------LIAISQRDTST--------YYKFL--QEK----------------------------------------------------KQHLQDLIQQFFNTI---------------------------------------------------VKDKFD--------------------------------------DINYTFDVYI------------------------------------------TRD-------------------------DKVYLMDFNPIHP----------------STDAL---------LF-DWEEL-----------------------------------------------------------------------------------------------------FEELMEE------------------------------------------------------------------KEEKETNNN-------------------------------------NNN--------------------------------------------------------------QNNNN------------------------------------------------------------------NNN
cdc123_Ddis_66819179                 YFENKKQCQ------FQEWYEK----------------------------------------------------------------------------------------------FKSVTFSSIVI---------------------PLPKI-------FID---------------------------------YLQSDQ---------------------------------------------------------------------FTTPHEGFPEFKVDEHDDLFDDNNWSTSKNHISTLDPKYYQGYS---------DEEDESSDDDDSNDNDKDKKPKRIVNE------------TEFK-------------------------------------ELSNQIIKSIEK---------------LGG-----------NIFPKLNWSSPKDA---------------------------------------------------------------SWMN--VYNS----------------------------------LKCTNTTDIY------------------LLLK-S---SDF-INHDL----------------------MQFSINQDDKD--------------DSLTP--------------------------YVLVLRKWQ--------------------------NLQPSM---EFRC-------FV----------------KDNQ-----------------LLGISQRDIST--------YFKFL--KDK----------------------------------------------------KQKIQDAIVKFYNES---------------------------------------------------ICGKFS--------------------------------------NNSFTFDCYV------------------------------------------TKD-------------------------EQVWLIDFNPIHP----------------STEAL---------LF-VWDELIPELI------------------------------------------------------------------------------------------------EQDQDEKENEETKQAKEELPIEPLTK-----------------------------------------------LEFRIIDDESGI----------------------------------KPN--------------------------------------------------------------LAMTSRLPLDLLQSSGG-------------TGQG---------------------NINDILLNFK------NQN
DICPUDRAFT_158438_Dpur_330843241     FFENKKQCQ------FQNWYDK----------------------------------------------------------------------------------------------FKAVTFSSVII---------------------PLPKI-------FID---------------------------------YLQSDDFSFGNGDFPEFRVEDNDLLDDNNWSTPTTITKPDPKYYQSNS---------------------------DNEEES-----------------------------------------------DDDDDDDEEEEENSSKNKRVIKQ----------------TDFP-------------------------------------ELLDKIKTAIEK---------------MGG-----------TVIPKLNWSAPKDA---------------------------------------------------------------IWMN--TYNS----------------------------------LKCTTPTDIF------------------LLLK-S---SDY-INHDLLQ--------------------YKIKEEEDD----------------NTTTP--------------------------FVLVLRKWQ--------------------------NLHPSM---EFRC-------YV----------------KDNK-----------------LIGISQRDTST--------YFNFL--KDK----------------------------------------------------KDKILNAIINFYDNS---------------------------------------------------IKEKFN--------------------------------------SSSFTFDCYV------------------------------------------TKD-------------------------DKVWVIDFNPIHP----------------STESL---------LF-LWDELFPE--------------------------------------------------------------------------------------------------LIEDDEESLQAKKEDPIKEITE---------------------------------------------------LEFRIVQDENSI----------------------------------KPN--------------------------------------------------------------LSMKSRLPLELMN-----------------LSNSD--------------------NINELLQQFN------DQN
SPAP27G11.03_Spom_19114319           MTLILTKNQVL-HCQFSSWYSL----------------------------------------------------------------------------------------------FRKLTPKAKVIK--------------------PIPAT-------VLK---------------------------------YLHEDS---------------------------------------------------------------------IYVEQPM----------------------------------------------NTVEEVDSEED----------------------------EESAPAYYPER------------------------------EAIQLIEKAIKE---------------LGG-----------AVVPKLNWSTPKDA---------------------------------------------------------------LWIT--TTGS----------------------------------LKCTTAEEVL------------------LLLK-S---SDF-VAHDL----------------------NHAFDDCKDFDNADGSV--------PKDFS--------------------------FELVLKEWF--------------------------PMHAST---EFRC-------FV----------------KSKR-----------------LIAFCQRDDN---------YYEFL--KEN----------------------------------------------------IDCYEKLISDL--LK---------------------------------------------------KLDTFP--------------------------------------DPDFVFDVYI------------------------------------------HKD--------------------------RAWLIDINPFYP----------------RTDGL---------LF-SWSEL-----------------------------------------------------------------------------------------------------ESMNSENMK----------------------------------------------------------------PEIRLIPKGS------------------------------------MPSTGSA----------------------------------------------------------KYYTNRVPFDMIA-----------------ASEGE--------------------NLLEFAQKWQDLT---NKS
Esi_0013_0099_Esil_299115353         VSPAPTQGHVL-NCQFGRWHPV----------------------------------------------------------------------------------------------FKHCTPRSVVL---------------------KLPED-------VVR---------------------------------YLQQDG---------------------------------------------------------------------VVLPKGFQMSCGEGVR-------------------------------------DDSDDEVDWGNEDEDEQDR--------------------PDFP-------------------------------------DLHALLSDAIAS---------------LGG-----------AVFPKLNWSCPKDA---------------------------------------------------------------AWVN--GG-S----------------------------------LKCKLPGDVL------------------CLIK-S---STF-ISHDL----------------------NHAFDACTGSSI-------------SRPET--------------------------FTLVLRKWC--------------------------NLHPSM---LFRC-------FV----------------RERR-----------------LVGVCQRDCTS--------YYGFL--EEE----------------------------------------------------ADRLSTLLEEFFAAE---------------------------------------------------VCKKFA--------------------------------------DPDCVADVYV------------------------------------------DNR-------------------------SRVWLLDMNPFSG----------------VTDSL---------LF-DWSEDSLAAPLPPRPPTLDRG-------------------------------------------------------------------------------------EETLPPGVAMRIHFDPSSTHGDQASSTPSGTVAARSNDPSDSQQGPPGGRTRATGVLERATGITLAGGGEGRDFEFRCVPSSLHM----------------------------------VPD--------------------------------------------------------------PMGRYRGPADVGMGTLA-------------CGTG---------------------GNGGLELEDLIESCRLAEK
LOC100215744_Hvul_449671755          SRQQCINCI------FSNWYNS----------------------------------------------------------------------------------------------FKDITFRSKVI---------------------RLPPD-------FIA---------------------------------YLKKDG---------------------------------------------------------------------VVLPNIEETRYVNDL--------------------------------------DGYSDSDNEDWGTSDQDHTLA------------------PCFS-------------------------------------LIKNKVDKVIDY---------------FGG-----------SVFPKLNWSSPKDA---------------------------------------------------------------VWIT--MDGT----------------------------------LKCSSFNDIC------------------LLLK-S---SDF-ISHDL----------------------NDAYSHCIESTLP------------HSDDA--------------------------FELVLREWV--------------------------DLIPSM---EFRV-------FV----------------KERI-----------------IIGISQRHSSG--------YYSYL--HTQ----------------------------------------------------KDILLQEIIRFFNLK---------------------------------------------------IKSKFL--------------------------------------DSNFVFDIVK------------------------------------------LEN-------------------------GCYKLLDFNPFGE----------------VTDGL---------LF-SWSELRSLS-------------------------------------------------------------------------------------------------PQSIDNS------------------------------------------------------------------KILRLVSELENI----------------------------------QPN--------------------------------------------------------------PYLSYRLPKDVVD-----------------LSCGE--------------------DVSKMIDFLNVHSLVAKPG
mg809_MVc_363539772                  YKTELRKTFLN-RYAHIINYPIN---------------------------------------------------------------------------------------------WTH-----------------------------ELTLE-------ESK---------------------------------IILHISK--------------------------------------------------------------------IYGTTK-----------------------------------------------CSIE------------------------------------IFN-------------------------------------NVLNPIKSRLEEKW-------------ISG-----------RWFMRLDALSPKDS---------------------------------------------------------------NYKM--PY---------------------------------------TNPSDII------------------NSIV-T---SKR-TFNAL----------------------LD-----NI----------------SLNIN--------------------------TKLYFVTYNE-------------------------NWKPSH---ELRC-------FV----------------YNKK-----------------LTAISQYCWTKEEY-----YCDKT--NKE----------------------------------------------------LVEIATKVNYFITNI---------------------------------------------------IDNICAQID-----------------------------------TTNIIFDLYL------------------------------------------NDD-------------------------LSMNIIELNCFGYWL--------------ASGSA---------LF-HWIRD-----------------------------------------------------------------------------------------------------KDKL---------------------------------------------------------------------------------------------------------------------------------------------------------------------------------------YN-----------------------------------------------------------------TDGN
LOC100175373_Cint_198424565          NKQEILNCL------FSNWYHL----------------------------------------------------------------------------------------------FRRHTIKSVVI---------------------PLPED-------FVK---------------------------------YLQDDG---------------------------------------------------------------------TVVLPRGVVLPGATREEWDNEEEEGDNGEERSE--------------------EEWEENENEEEGNN-------------------------PEFT-------------------------------------ELMEEIGKLIED---------------LGG-----------KVFPKLGWSSPKDA---------------------------------------------------------------AWIA---NNS----------------------------------LLCNTAGEVV------------------LLLK-S---SDF-IIYDL----------------------TSVFKLCTDVES-------------DANIP--------------------------HQLVLRKWA--------------------------NLVPGM---EFRC-------FI----------------RNKS-----------------LLGVSQRDYKQ--------HYDYI--IKD----------------------------------------------------KDKLAEEIHNFVSDE---------------------------------------------------IIDKFP--------------------------------------SDSFVVDIYK------------------------------------------KRD-------------------------GNFWVIDFNPYGE----------------MTEPL---------LF-TWGELSS---------------------------------------------------------------------------------------------------GSLSTCDFYVDMKTADDVTI-----------------------------------------------------GQFRYTPSDVTM----------------------------------QPS--------------------------------------------------------------EHLRYRMPQDFVD-----------------LSTGS--------------------DATKLIDFMRMKIQNDDVE
LOC101857350_Acal_524897036          SKSNVMMCS------FSSWYKA----------------------------------------------------------------------------------------------FEDVTVKSIVL---------------------PLPDF-------FVS---------------------------------YLHADG---------------------------------------------------------------------VVLPESSSHGIYTKSANTAMDGDEDDPDIRDDELMKMYE--------------DDWSTPDSSEGAKV-------------------------PDFG-------------------------------------DFDESVKEAIKS---------------LGG-----------KVFPKLNWSSPKDA---------------------------------------------------------------NWIS--FDKT----------------------------------LMCTCPSDIY------------------LLLK-S---SEF-IAHDL----------------------DQPFVHCDD----------------AGDDSAENSPSIS------------------YCLVLKKWQ--------------------------PPDPST---EFRC-------FV----------------HDKK-----------------LIALCQRQATK--------FFSHI--NHE----------------------------------------------------RESIISDISKFHQQK---------------------------------------------------IAQRFS--------------------------------------ETSYVFDVVR------------------------------------------PEQ-------------------------GKVILVDFNPFGL----------------VTDSL---------LY-SWEDIEGLLKNMD---------------------------------------------------------------------------------------------KRAADQT------------------------------------------------------------------PDFRCVESEGGV----------------------------------QTS--------------------------------------------------------------DYANYALPRDIQD-----------------LTSGE--------------------DPYKLMDLLKLRDNADSSS
_Zfor_521962559                      TDEEVKQWT------TAKWKAQESALLSGLHPA-----------------------------------------------------------------------------------IMAITIPTMFF---------------------AIPMQ-------EVKEKWLPVF--------------------------DGKNDG-----------------------------------------------------------------------------------------------------------------------------------------------------------------EACT-------------------------------------RQVECGRRALAE---------------FPD-----------GVFFKLDSRSPKDSD--------------------------------------------------------------IGKY--TA---------------------------------------ENLDQLP------------------NAFF-G---SER-VFDDI----------------------CL-----QR----------------HHRDR--------------------------IVLCFRKWV--------------------------EFGE-----EYRV-------FV----------------KERQ-----------------IQGISRYDYLSAS------KVEHT--PEV----------------------------------------------------VAAVQGQAEGY-LAT---------------------------------------------------INEHYP--------------------------------------PSDYVFDIGH------------------------------------------TPD-------------------------GPVM-IEINPY-G----------------LSDPC---------LF-GS-----------------------------------------------------------------------------------------------------------------------------------------------------------------------------------------------------------------------------------------------------------------------------------------------------------------------------------------------------------------------YAN
RFI_23039_Rfil_569378781             ---------------FDDWYPV----------------------------------------------------------------------------------------------VEKFTMRSELI---------------------PINIK-------EIR---------------------------------AIMNAHESK------------------------------------------------------------------RPQNVG-----------------------------------------------MTIS---KEDT----------------------------EQLE-------------------------------------VFKKRIEEFLKKKTSD-----------ENKEKEEKEEKKE-GIFLRFSNRSPKDSILRINKEKFEESVRKLVKEIYEAQKKSLE----------------------------------QYSN--EAQIVNAIIRALTILTSSQ-------------------LCILNGDMAM------------------DLVL-N---SER-LFTDFKMG-------------------EL-----IR----------------GDDYQ--------------------------TQLILREFNH-------------------------QIDFEC---EFRC-------FV----------------FKDR-----------------MTCISQYNSMA--------FVPRI--VEN----------------------------------------------------QLLIPTSIVKYWSSN---------------------------------------------------IHPLMQQAG-----------------------------------ITSYVLDLAL------------------------------------------VKDSM-----------------------D-IILIEINPFCT----------------KAGAL---------LF-DWKSD-----------------------------------------------------------------------------------------------------LQILLSADP----------------------------------------------------------------VVFRFCQQPHSQ----------------------------------QPEH-------------------------------------------------------------LTSEL---------------------------------------------------------------HSIFQS
BRAFLDRAFT_117626_Bflo_260798456     ----MKKRQVL-NCIFSAWYPR----------------------------------------------------------------------------------------------FEHLTFPSVVL---------------------PLPAQ-------FVS---------------------------------YLLADG---------------------------------------------------------------------IVLPNSGKGKSQKSSYKAHPDDDSDV---------------------------EDPDWSDEEEDAPTEA-----------------------PEFP-------------------------------------ELEAQIKQAIAH---------------LGG-----------KVFPKLNWSAPKDA---------------------------------------------------------------SWIA--LNNS----------------------------------LQCTCPEDVY------------------LLLK-S---SDF-VTHDL----------------------TQPFDRCEDEDT-------------DVSVH--------------------------YELVLRRWT--------------------------NVHPGM---EFRC-------FV----------------KNDQ-----------------LIAISQRHHSS--------FFQYI--HDQ----------------------------------------------------HDGIQADIVDFYHTD---------------------------------------------------IEKKFP--------------------------------------DSNYVFDVYR------------------------------------------KKA-------------------------GKLMLVDFNPFCE----------------VTDPL---------LF-TWEEL-----------------------------------------------------------------------------------------------------TYRVPED------------------------------------------------------------------IAHLTSGED-------------------------------------AYK--------------------------------------------------------------LIDFL-----------------------------------------------------------QMKCQSQEGE
_Ecal_515580012                      PSSIVSYYL---------------------------------------------------------------------------------------------------------------TPPAVAI---------------------ALNKQ-------QLT---------------------------------AV----------------------------------------------------------------------------------------------------------------------------------------------------------------------LSR-------------------------------------NLRYRRQYGLSP---------------RNVSLSTQPSIQQQDYLPKLGVVSWKDC---------------------------------------------------------------IGMD--MLPKA---------------------------------LLLPNAQNTT------------------LTCW-LNNVSDR-MAMVL----------------------HA-----YR----------------VTEET--------------------------PTFYLFPYL--------------------------DFSKRS---EYRL-------AV----------------SYGE-----------------LT--HVR------------CYRRR--NDF----------------------------------------------------QTKHTDAIAAW-WRN---------------------------------------------------IKDWPPTDV-----------------------------------LAHLFVDVVA------------------------------------------GSDP------------------------GQFFIIDVNPN------------------------------------------------------------------------------------------------------------------------------------------------------------------------------------------------------------------------------------------------------------------------------------------------------------------------------------------------------------------------------------------------------------LSA
BBBOND_0306250_Bbig_656186323        MKRLLREYA----FNEEKGSSMVAPQIGALDAVLYVLWNWVSESGEKAIGSHMYIMNNDEGVCFSHE-------------------------------------------------NVKRNFGSMLI---------------------PNSLQ-------ILEGSLLD----------------------------YLKSDN---------------------------------------------------------------------MSMPPNIDQIKYKDVDSGSDFDGY-----------------------------ES------------------------------------------------------------------------------MSDSDDSFDWS----------------AVE-----------PFVEKLKALIAKYQ-----SVVPSINGVFLDDA--------------------------------------------LWVA--NM-N----------------------------------VECTSAREVL------------------LLLK-S---STC-WQDLE-------------------------------------------------GSQ--------------------------CHLTLYPGI--------------------------YFRGRL---QLRC-------FL----------------FENE-----------------LVCVEQIFVNE--------NFGFL--AKD----------------------------------------------------AQSLVGSLKSY--SPSVMG-----------------------------------------------VLNKGG--------------------------------------VRSAIFDVTVSTKNGEIEMLNIYQFGS-------------------------LPD-------------------------GLLQLEDVYHFYYT---------------GKSDG---------LK-PLDLA-----------------------------------------------------------------------------------------------------DMAVVDG------------------------------------------------------------------VLVVFVGAN-------------------------------------SSR--------------------------------------------------------------LQKHAWCPNDIGN-----------------LSFN---------------------THDDLIDYLR------FQA
_Pcar_497965255                      MYSEHKATF------IENWPKE----------------------------------------------------------------------------------------------LLDLSFLSEGF---------------------ELHER-------DVI---------------------------------AIGANT---------------------------------------------------------------------HDFMNA-----------------------------------------------RGLL------------------------------------EKPLYSA---------------------------------QLREDIEYALSV---------------LNK-----------PAFLRFGGVSYHD-----------------------------------------------------------------------YAR----------------------------------PRLETVDGVI------------------EQLA-V---SNRRVASYL----------------------WD-----CL----------------QSSTP--------------------------VWLYLREWR--------------------------EILRWG---EFRC-------FI----------------KEGK-----------------VIGVSQYHCLE--------YFPFI--KEK----------------------------------------------------ENEIRLQLIAF-LQK---------------------------------------------------LLPVLH--------------------------------------VDSVVADVAITYQ---------------------------------------DSK-------------------------FATTLIELNPFIQ----------------RTDAC---------LF-SW----------------------------------------------------------------------------------------------------------INGGD------------------------------------------------------------------FNGRIRINL-------------------------------------SDA--------------------------------------------------------------DAQAE------------------------------------------------------------------KQR
IW01_15560_Pcar_671736394            MYSEHKATF------IENWPKE----------------------------------------------------------------------------------------------LLDLSFLSEGF---------------------ELHER-------DVI---------------------------------AIGANTH--------------------------------------------------------------------DFMNARGLL--------------------------------------------EKPL------------------------------------YSA-------------------------------------QLREDIEYALSV---------------LNK-----------PAFLRFGGVSYHD-----------------------------------------------------------------------DAR----------------------------------PRLEAVDGVI------------------EQLA-V---SNR-R---------------------------------VASYLWDCL---------QSSTP--------------------------VWLYLREWR--------------------------DIPRWG---EFRC-------FI----------------KEGK-----------------VIGVSQYHCLE--------YFPFI--KEK----------------------------------------------------ENEIRLQLIAF-LQK---------------------------------------------------LLPVLH--------------------------------------VDSVVADVAI------------------------------------------TYQDSE----------------------FATTLIELNPFIQ----------------RTDAC---------LF-SW----------------------------------------------------------------------------------------------------------VSGGD------------------------------------------------------------------FNGRIRINL-------------------------------------SDA--------------------------------------------------------------DAQAE------------------------------------------------------------------KQR
_Bsp._496403708                      MYSEHKATF------IDNWPQD----------------------------------------------------------------------------------------------LLALSFLSEGF---------------------ELHER-------DVI---------------------------------AIGAST---------------------------------------------------------------------DEFMTA-----------------------------------------------RELQ-----EK----------------------------PVFSV------------------------------------QLHDDIEYALSV---------------FNR-----------PVFVRFGGVSYHDA---------------------------------------------------------------SLSR--LD---------------------------------------TVDGVVK------------------QLSV-S---SRR-VASYL----------------------WD-----CL----------------QSSTP--------------------------VWLFLREWR--------------------------DIPRWG---EFRC-------FI----------------RDAK-----------------VIGVSQYHCLE--------YFPFL--KEK----------------------------------------------------ENEIRLQLIMF-LQK---------------------------------------------------LLPVLH--------------------------------------LDSVVADVAI------------------------------------------DYQDGK----------------------FTTTLIELNPFIQ----------------RTDAC---------LF-SW----------------------------------------------------------------------------------------------------------VNGGD------------------------------------------------------------------FNGRIRVNQ-------------------------------------SIA--------------------------------------------------------------DAQAE------------------------------------------------------------------KRK
_Dchr_654084322                      MYTEHKQTF------IENWPET----------------------------------------------------------------------------------------------LLTLSFQSEGI---------------------ELHER-------DVI---------------------------------AIGACT---------------------------------------------------------------------SEFMDAKRLL-------------------------------------------EKPP------------------------------------FSE-------------------------------------QLRDDIEYILSK---------------FTT-----------PVFVRFGGVSYHEA---------------------------------------------------------------TIPR--TN----------------------------------------NVDDVI------------------RQLSVS---SHR-VASYL----------------------WD-----CL----------------QSSTP--------------------------AWLFLRGWH--------------------------DIPRWG---EFRC-------FI----------------KDGN-----------------VVGVSQYHCLE--------YFPFL--TEH----------------------------------------------------ADEIRQQIIQF-LQK---------------------------------------------------LIPLLH--------------------------------------MDSVVADIAI------------------------------------------THQNGQ----------------------YDTMLIELNPFIQ----------------RTDAC---------LF-SW----------------------------------------------------------------------------------------------------------LNGGD------------------------------------------------------------------FNGRIRVNLS------------------------------------QAD--------------------------------------------------------------ASAEK------------------------------------------------------------------RRR
LBRM_28_2200_Lbra_154340585          PLSIVFSGE-----------------------------------------------------------------------------------------------------------GRRHAIPHRTFCVAAQSEDGSAGVSADT----DIAEE-------GVAFFR------------------------------YLDSND---------------------------------------------------------------------TRMPSHPHLRAYAHSAEEYETYV------------------------------GEEVWDDADGAPTSASRQPPERDTGKVQSGSGSCYATLDTVAPVPSFRSVVEW--VSGVFEEDWTSDGVVLCG-------RYVVADDGAWAVPSRSPTLYTT-----REV-----------FLLMRNSSKFLHDV-------------------------------------------------------------------------------------------------------------HTQVVDLK------------------AVAK-R---GAH-SGSLT------------------------------------------------------------------------------LEFTLAKSL--------------------------AGSGAS---EMRAILPYPLYFS----------------PQTRTWVVRDTAASLS-----FTGIGQRMTDV--------CFPSL--MAWTEAEHDNNFRFMQRRIEQAALLERTCAT------------------------DPGFISRLVSAFDKEKH-------------------------------------------------LAYASDGAAGQQHPYPRLALLLTVDLL-----------------FESSSLPIYL------------------------------------------LSS--------------------------KARVFEVASRTS----------------VTWGA---------IN-DASQWHLWTPLL----------------------------------------------------------------------------------------------VDADTSGADGALDASAAAPTVKAACGCDDGIHAA---------------------------------------PLGEDEESDETEEEEEDAATRSLNFFRMFRGIGHWNQYVSSMVARLQQS--------------------------------------------------------------RSDALGL-----D-----------------DSNKPSRDVQHYCV-----------IASESADLVNCADTLTKRG
consensus/100%                       ...............................................................................................................................................................................................................................................................................................................................................................................................................................................................................................................................................................................................................................................................................................................................................................h.....................................................................................................................................................................................................................................................sh...............................................................................h..................................................................................................................................................................................................................................................................................................................................................................................................................
consensus/95%                        .....................................................................................................................................................h.......................................................................................................................................................................................................................................................................h.................................h.+.s....p..................................................................................................................................................s........................................................................................h.h..h....................................bhR........hh.....................................h..h..............................................................................................................................................................................................h.hDh............................................................................lhph..................................................................................................................................................................................................................................................................................................................................................................................................................
consensus/90%                        ..............................................................................................................................h......................ls..............................................h...................................................................................................................................................................................................................h...h................................hh.+.s..s.ps................................................................................................................s...h.....................h......S........................................................................................l.lb.a............................h.......EhRh.......al.....................................h.shspb.............................................................................h...h..h.....................................................................................................ph.hDhh...........................................................................lh-hssh...................s.s..........hb..b..........................................................................................................................................................................................................................................................................................................................................................................
consensus/85%                        ..............................................................................................................................h......................ls..............................................l...............................................................................................................................................................................................................h...hp..l.................b..............hh.+hs..u.pDs.............................................................................................................h.s..phh....................h......S.b......................................................................................lhlb.a...........................ph.......EaRh.......Fl.................p.p.................l.slspb................h............................................................h...h..h..p....................................................h.............................................ph.hDhhh............................................p...........................h.ll-hssh...................s.s..........hh.ph..........................................................................................................................................................................................................................................................................................................................................................................
consensus/80%                        ..................a.....................................................................................................s..s..h......................ls..........hp.................................hl...............................................................................................................................................................................................................h...hp..lp................hs.............hh.+hs..us+Ds.............................................................................................................hps..phh...................hl......Spb.h...h................................................................................lhlbpa...........................ph.......EFRh.......Fl.................p.p.................l.ulsQb.............h..h..........................................................p.l.p.h..h..p....................................................l...h.........................................ph.hDlhh............................................p...........................h.ll-hssh...................sss..........La.sa..........................................................................................................................................................................................................................................................................................................................................................................
consensus/75%                        ..................W.................................................................................................hpp.sh.s..l......................ls.p........hp.................................hl..............................................................................................................................................................................................................ph..plppslpp...............hss............hh.+ls.puP+Ds...........................................................................................................h.hps.pphh..................bll......Spb.h...l................................................................................lhl+pa...........................ph.......EFRh.......Fl.................p.p.................l.ulsQb............ha..l...p.....................................................pp.l.p.l..a..pp...................................................l...h........................................pshhhDlhh..........................................spp...........................hbll-hNsa...................sss..........LF.sa.p........................................................................................................................................................................................................................................................................................................................................................................
consensus/70%                        ....h..........h.pW.s...............................................................................................hpp.oh.s.hl......................ls.p........hp.................................hl..p...........................................................................................................................................................................................................ph.pplppslpp...............hss............hF.+ls.puP+Du................................................................hb....s....................................hphps.pchh..................bllp.s...Spb.h.psl................................................................................LhL+ca...........................shp......EFRh.......FV................pp.p.................l.uloQb............has.l...pp....................................................pppl.p.l.pah.pp...................................................l...h........................................pshhhDlhl..........................................spp..........................phbll-hNsas..................ossh.........LF.sWpp......................................................................................................................................................................................................................................................................................................................................................................p.

B. R2K.2 family

SECONDARY_STRUCTURE                        ----------EEE----------HHHHHH-HHHH-------EE--EE----------------------------------------------------------------------------------------------------EEE---------------------------------------------------------EEE----HHHH-------HHHHH----------------H--HHHHH---EEEE-E-H---------------H--------HH----------------E-EEE---------E-------------EEE--------------------------------------------------------------EEEEEEE-E---E--------EEEEEEEE--------E----------------------EEE------------------------------------------------------------------------H----H---H-------HHHHHHH-------------------EEEEEE------------------E--EE-------------------EEEEEE-H-HH--HH------------H------HHHHHHHHHHHHH----------H--------------------------------------
AZ78_15305_Lcap_601089719                  VT-WA-----IQT-NFIAD--EQIRRVWN-AAED-AGAR--VQDVQV---IPF-SD-EL-GN----------HVP---------R----ID-----------------------------------------------GV--V----IPYGAT------K-----L-TR------L--A-MQR------G-WSG--LCFD-----PETFRV---D----------CW-NRNRD-------------------DMLNQHVRQ-MSV-----------R---E-CM-AAM-EHEPD------D-SVW----FVRPLHDLKHFDG------TVTVAR-EIRRWMTS-V---------DS-GNFSFD-------G--DT-------------------------EVIVAPP-Q-KIH--------AEWRYFIVDG-----TV----------------------VDGS----SYR-IAGQ------RMA-----------------------NAV--------------ARPEL-Y---E----Q---A--QAL--ADGWL---------------PH-R--TCVMDV------------------A--QTD------------DG----FKVIEFNT-FN--SSGFY-A-H-D---I------EKIVAAVTAHFAA--S---P-R-AA-T-A--VE--AD--APG-------------------------------------------------------------------------------------------
CcrColossus_gp324_BPCcrColossus_414088380  VA--------HLI-ASGIE--HHLVRVLP-MTSD-GGRK--APEIEG-K-----TP-VL-PA-------------------------------------------------------------------------GV-PV-------VVYGSV-G----V-QA--L-AR-----------QHG-------------------W--TPGVWT---DHEN-FNYE--TFRDKLGD-------------------LLLNDDMER-MPL-SAA-----------G----AYF-AALTP------G-EKR----FIKPNLDTKEFAG-Q-----------VITADDFD----VWLAG--MI-DTGYLT-------K--DS-------------DF----------DVVIAAP-K-DLG--------VEWRAVVVDG-----KV----------------------SSCC----IYR--QWQ------RVM-PE-----------------------------------------------L----H---I--LPE--VEDLILK-AHA-----K-FAPG-D--VYVIDV------------------A--QQY-QM-I---N---GERDYVFKIIEYNT-FN--SAGLY-A-C-D---V------VKIIDDINAFLER------S-----------------------------------------------------------------------------------------------------------------
_Cfla_494035823                            MH------W-VLQ-ASFLSE-PGRSALIA-ALER-FGIS--YSVHKV---VPK-EG-DL-IP-----------------EP---E-L--KH-----K-----------------------------------------NV-------ICFGSY------S-----L-RP------A--V-LRN----------------------QWSPGL-F-DVLD-Q-----DFETQREH-----------WGA-----HMLNFDSVV-SSV---------------R--------DAAFT-T----E-----SM-FVRPVNDSKYFSG-------------RIFTAQEF----GAWQRS-IC-QPEADR-RTSLA-P--QT-------------------------RIQLAPL-V-TIH--------AEYRFWIVKN-----EI----------------------ITQS----LYR--LGG------KAT-PA-------------------------------------REVDE-----K--------F--ASF--VNERIRQ-----------WTPH-E--TFVIDV------------------C--DTP------------GG----IKIVEVNT-LN--CSASY-A-A-D---V------QRLVLALEQAYSQ------------------------------------------------------------------------------------------------------------------------
_Msti_505162224                            MH------W-VIQ-DNLFNE-RGFRDLVQ-VLER-GDIP--HTRVKV---IPF-DG-GV-EP-----------------FV---D-V--PG-----------------------------------------------PV-------VIMGSL------T-----L-TR------Y--A-RKR----------------------GWTPGA-F-LNDQ--FDFR-VWREHLGE-------------------HLLNADAHV-CRF---------------A--------DVSER-E----G-----PF-FIRPCLDDKAFSG---MV--------TTWEDFHR-WR-EGVLA--VQ-EYPQLT-------A--ET-------------------------WVAVSEP-R-HIQ--------SEYRMVVVDG-----QV----------------------VTGT----RYK--LGA------RVF-AS--PE---------------------------------------V-E-P----V---V--WNF--AQRMADR-----------WGPD-R--AYALDI---------------------FMH--------Q---HQ----PYVGEINT-LN--AAGFY-A-Y-D---V------GKMVAAIEA--------------MT---F--------------------------------------------------------------------------------------------------------
IJ00_19815_Csp._668697069                  MH------W-VLQ-EGFVSE-AGWEELIA-TLER-FGIS--YSVHHV---VPR-VG-NI-VP-----------------DL---S-I--DH-----N-----------------------------------------NI-------ICIGSY------A-----M-RH------V--A-SRQ----------------------GWIPGV-F-DLFA-Y-----DFKQQRLH-----------WGE-----HLLNFHSTV-CTL---------------E--------HARFA-L----P-----KM-FVRPIHDSKHFSG-------------RVFNQEDF----VTWQRS-IC-ESTMNH-NTSLT-P--QT-------------------------KIQLSRP-I-SIY--------AEYRFWIVGD-----EI----------------------VTQS----LYK--RGG------QVY-YH-------------------------------------RDVDE-----P--------I--ASF--ARARVNE-----------WAPH-E--AFVIDI------------------C--NSE------------LG----VKIVEINT-IN--SSGFY-A-A-D---V------QRLVLKLEERFTQ------------------------------------------------------------------------------------------------------------------------
_Zfor_521964427                            MR----PVW-LIE-AGVYG--EEAAPLLG-EIRR-QGMV--AEIVPH---QAL-RK-GA-SP-----VIAGGQVL---------E----PG-----------------------------------------------AC--V----IGYGTY------P-----F-AQ-QI------L-LHH----------------------DWTLGA-W-CSAE-NLDCV-TYYARFGR-------------------YLLNQQYVM-MPG-----VEAIRQR---D----WLF-SVFGR-E----E-----QV-FVRPTSCLKLFVG-------------RCVDQAAF-AT--------AL-APTRYD-------P--AT-------------------------LVVIAAP-Q-PID--------REWRLVVVGD-----RV----------------------ISGG----QYA--VNG-AR---SIT-PD-------------------------------------------C---P----A-A-V--KSF--AESMLAE------VK---WRPD-P--VFMLDV------------------C--ESA------------GQ----LWLVELNS-----FSGSW---L-Y---R------CDLPAVVAATSEL--A---G-R-LW------HN---------------KH------N----------------------------------------------------------------------------
_Lcae_652981767                            MT-TL-----YYR-LSGTT--DPEERLED-ACLS-KAMH-DTYGSRI---TIL-PP-EA-SP---------------------------PA-----------------------------------------------DA-------TVFGRG---RQ-L--G--F--------------GTS----------------------GSGQAK-L-DYAR-DP----GFLWGISR-------------------SFKVCGLQE-AEL--------------------EVA-RLHAA------G-KDA----FVKAMQ-EKLMTE------RVPRGM-SLHEAIGD----MVYS---FI-----------------ER---------PD--------------CLMVQEH-V-AMR--------NERRLVFVDG-----TL---------VTHSPVAFHLTP-LDRS----RLA--AET------GRP-AE--EL-H-----Y-------------------------TDPES-R-T-P-VW-N------AEL--SRRMVRF-ANE--VA---ARSR-LR-TMTIDV------------------A--ELA--------D---GR----LEVIEFNS-GWPGSFGLF-A-C-D---P------YAIAKASSALLPA--------D----P----AG-----------E--------V--RCRKVEHLTAPAPREAAADTDADWCDEP-------------------------------------------------
[truncated: 853,177 more chars]
